# RTPhy‐ChatBot: A RAG‐Based intelligent assistant for radiotherapy physics using LLaMA3 and AAPM reports

**DOI:** 10.1002/acm2.70263

**Published:** 2025-10-09

**Authors:** Shuoyang Wei, Ankang Hu, Zhiqun Wang, Xiangyin Meng, Lang Yu, Bo Yang, Jie Qiu

**Affiliations:** ^1^ Department of Radiation Oncology Peking Union Medical College Hospital Beijing China; ^2^ Department of Engineering Physics Tsinghua University Beijing China; ^3^ Key Laboratory of Particle & Radiation Imaging (Tsinghua University) Ministry of Education Beijing China

**Keywords:** large language model, radiotherapy physics, retrieval‐augmented generation

## Abstract

**Background:**

Medical physics plays a crucial role in radiotherapy, with ongoing technological advancements aimed at improving treatment outcomes. However, the rapid pace of innovation presents challenges for medical physicists, who must continuously acquire and integrate complex information for effective decision‐making and communication.

**Purpose:**

To support efficient knowledge acquisition, we developed RTPhy‐ChatBot, an intelligent assistant tailored to radiotherapy physics. The objective was to create a reliable and precise tool to assist medical physicists in their daily work.

**Methods:**

The knowledge base for RTPhy‐ChatBot was constructed from publications by the American Association of Physicists in Medicine (AAPM), which were converted into markdown format, segmented, and embedded using the bge‐base‐en‐v1.5 model. RTPhy‐ChatBot employed the Meta‐LLaMA3‐8B‐Instruct model for response generation. We compared its performance with several commercial large language models (LLMs) across 20 template questions and evaluated the impact of zero‐shot chain‐of‐thought (CoT) reasoning. In addition to expert scoring by senior medical physicists, we conducted Rouge score analysis against synthesized reference answers.

**Results:**

RTPhy‐ChatBot demonstrated strong performance in answering radiotherapy physics questions. Across 20 questions, it achieved an average score of 4.0 ± 0.9, compared to 3.9 ± 1.1 for Gemini‐2.0‐Flash, 4.0 ± 1.4 for GPT‐4o, and 3.8 ± 1.2 for Moonshot‐v1. It excelled in questions involving specific quality assurance standards. Rouge analysis yielded scores of 0.5127 (Rouge‐1), 0.2119 (Rouge‐2), and 0.2748 (Rouge‐L), closely matching commercial LLMs.

**Conclusions:**

RTPhy‐ChatBot proved to be an effective intelligent assistant for radiotherapy physics, delivering accurate, referenced responses grounded in AAPM publications. Despite lacking online access, it matched or exceeded the performance of commercial LLMs in domain‐specific tasks. This pilot study highlights the potential of domain‐specific assistants in supporting clinical workflows.

## 1 INTRODUCTION

Medical physics plays an essential role in tumor radiotherapy, with new technologies continuously shaping clinical practice. Innovations such as respiratory motion management,^1^ dual‐energy computed tomography (CT),^2^ and proton/heavy ion radiotherapy^3^, ^4^ have become integral to treatment delivery. The American Association of Physicists in Medicine (AAPM) has extensively documented these technologies, resulting in a vast and authoritative collection of technical standards and clinical guidelines. However, medical physicists often face the challenge of acquiring and applying this extensive and evolving body of knowledge for clinical decision‐making, research, teaching, and patient communication.

To meet the growing demand for efficient knowledge access, there is a need for intelligent learning tools that can deliver concise, accurate responses based on authoritative sources like AAPM reports. Such tools can reduce reliance on time‐consuming manual searches and improve efficiency across clinical, research, and training workflows.

Recent advances in artificial intelligence and natural language processing have made large language models (LLMs), such as OpenAI's Chat‐Generative Pre‐Trained Transformer (ChatGPT),^5^, ^6^, ^7^, ^8^, ^9^ increasingly popular across many domains due to their ability to generate coherent, context‐aware, and conversational responses.^10^, ^11^ While LLMs have been applied to clinical decision support and patient communication in radiotherapy,^12^, ^13^, ^14^, ^15^, ^16^, ^17^, ^18^, ^19^, ^20^ their use in the more specialized domain of radiotherapy physics remains limited.^21^

While general‐purpose LLMs have shown strong performance in many areas,^12^, ^17^, ^20^, ^21^ they often lack access to up‐to‐date or domain‐specific knowledge unless fine‐tuned^22^, ^23^, ^24^ or supported by external information sources using techniques like Retrieval Augmented Generation (RAG).^24^ Additionally, commercial LLMs rely on online knowledge retrieval or proprietary datasets, which raises concerns about data privacy, reference traceability, and response verifiability in clinical settings. Introduced by Patrick et al. in 2020, RAG enables models to retrieve relevant information from a specialized knowledge base during inference. This allows LLMs to supplement their pre‐trained knowledge with up‐to‐date, domain‐specific information without requiring extensive retraining.

In this study, we present RTPhy‐ChatBot, a domain‐specific chatbot for radiotherapy physics. It leverages a knowledge base composed of AAPM reports and applies a RAG framework using the Meta‐LLaMA3‐8B‐Instruct model. This design allows the chatbot to provide fact‐based answers grounded in authoritative publications while preserving privacy and ensuring traceability. We evaluated the chatbot's performance against several commercial LLMs using 20 template questions. Responses were assessed both qualitatively by a panel of senior medical physicists and quantitatively using Rouge metrics.

## 2 MATERIAL AND METHODS

### 2.1 System design: RAG‐based ChatBot architecture

**FIGURE 1:**
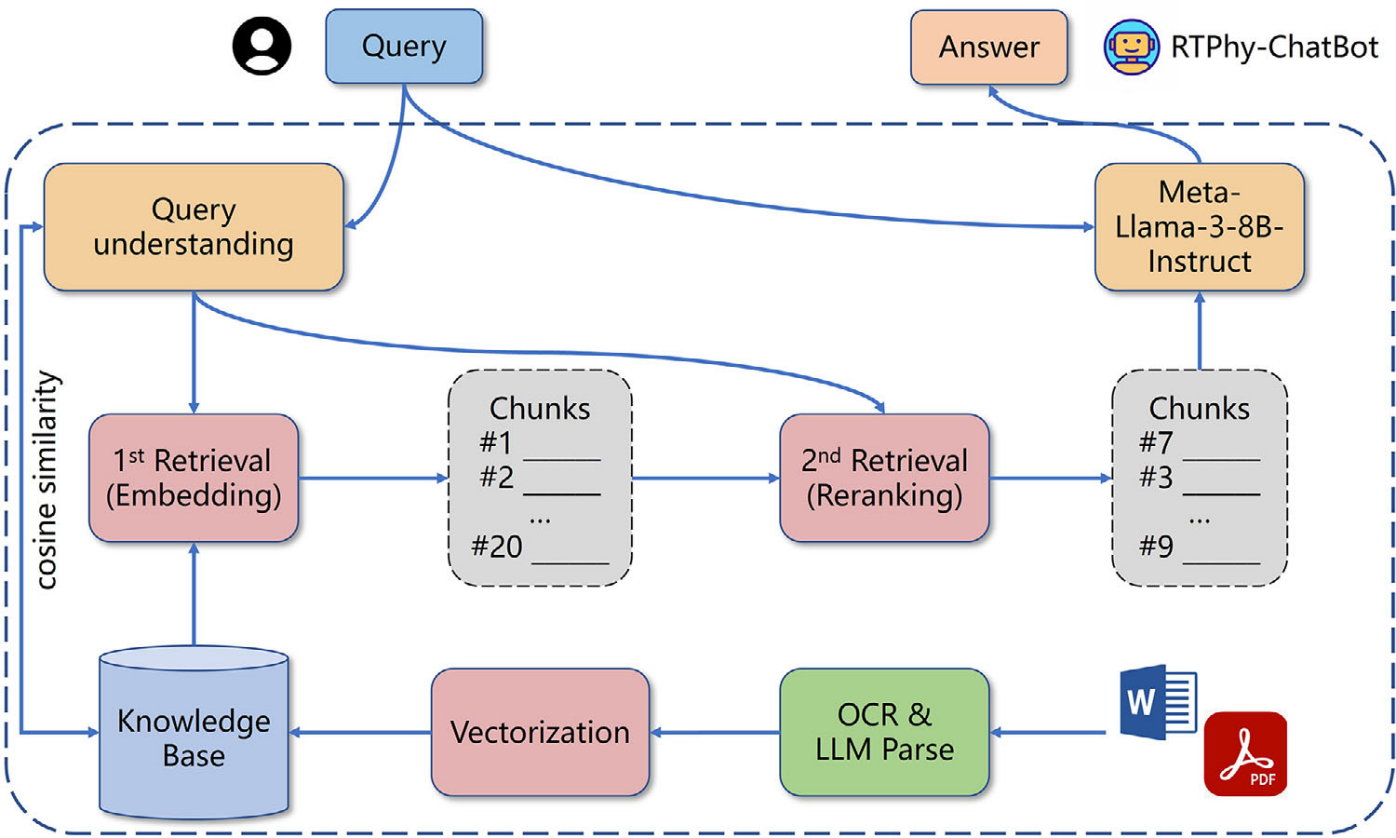
The schematic diagram of the RTPhy‐ChatBot.

The schematic diagram of the RTPhy‐ChatBot is shown in Figure 1. The system comprises two main components: (1) a structured knowledge base built from AAPM publications, and (2) a chatbot interface powered by a large language model integrated with RAG. We developed the RTPhy‐ChatBot using open‐source frameworks, with LlamaIndex serving as the backbone for indexing and querying.^25^ The workflow follows a three‐step process:

1. The user's query is embedded using the same model used for document embeddings.
2. The system retrieves the top‐k relevant chunks from the knowledge base using cosine similarity and reranks them based on relevance.
3. The reranked chunks, along with the original query, are passed to the LLM to generate a grounded and contextually relevant response.

To facilitate real‐time interaction, we also built a user‐friendly web interface, enabling users to query RTPhy‐ChatBot directly.

### 2.2 Knowledge base construction

The knowledge base for RTPhy‐ChatBot was developed using AAPM Reports TG001 to TG377, which were downloaded from the AAPM website in PDF format. These reports contain a mixture of text, images, and tables. To extract the textual content, we applied a combination of Optical Character Recognition (OCR) and LlamaParse, converting the reports into a structured markdown format suitable for downstream processing.

To facilitate semantic retrieval, the markdown content was segmented into small chunks with a length of 256 tokens using LlmaIndex's sentence‐aware splitter.^25^ This approach ensures that sentence boundaries are preserved as much as possible, enhancing retrieval relevance and reducing context fragmentation.

Each chunk was then embedded using the bge‐base‐en‐v1.5 model developed by the Beijing Academy of Artificial Intelligence (BAAI), which is optimized for passage‐level relevance retrieval.^26^ The model produces 768‐dimensional embeddings and is distributed under the MIT license. For each AAPM report, the associated plain text, segmented chunks, embeddings, and metadata (e.g., report ID, title) were grouped into a structured unit of knowledge. During query time, the system retrieves relevant chunks along with their metadata, allowing RTPhy‐ChatBot not only to generate informed responses but also to reference the source publications. This structure ensures transparency, facilitates citation, and helps users validate the provided information.

### 2.3 LLM integration and RAG workflow

In this study, we employed Meta‐LLaMA3‐8B‐Instruct as the core language model for generating responses. While LLaMA3‐8B is considered a relatively small model compared to state‐of‐the‐art commercial LLMs, it offers a favorable balance between performance and computational efficiency. Trained on 15T tokens and equipped with a 128K‐token vocabulary, LLaMA3 supports context windows up to 8000 tokens, making it suitable for complex domain‐specific queries.

To enhance factual accuracy of its responses and ensure domain relevance, we combined the LLM with an RAG framework. This approach allows the model to dynamically access a curated knowledge base of AAPM reports at inference time rather than relying solely on pre‐trained parameters. Given the large size of the knowledge base, a single query retrieval may return some potentially low‐relevance chunks, which could affect the performance of RTPhy‐ChatBot. To address this, we employed the reranking technique.^27^, ^28^, ^29^ By evaluating the query embedding and retrieved chunks, a reranker could re‐filter chunks to filter out irrelevant chunks and prioritize the most relevant chunks. In our study, the reranking model bge‐reranker‐v2‐gemma provided by BAAI was utilized.^30^

The RAG‐based workflow proceeds as follows:

1. Query Embedding: The user's query is embedded using the same embedding model (bge‐base‐en‐v1.5) used for document indexing.
2. Initial Retrieval: The system retrieves the top‐k candidate chunks from the knowledge base based on cosine similarity between query and document embeddings.
3. Reranking: A reranker (bge‐reranker‐v2‐gemma) is applied to evaluate and prioritize the candidate chunks based on contextual relevance.
4. Prompt Construction and Response Generation: The top‐ranked chunks, along with the original query and optional conversational history, are formatted into a prompt and sent to the LLM for final response generation.
5. Metadata Traceability: The generated response includes citation metadata (e.g., AAPM report ID) to improve transparency and traceability.

This architecture allows RTPhy‐ChatBot to produce grounded, explainable answers while maintaining flexibility. It also enables rapid updates to the knowledge base – new guidelines or reports can be added without retraining the model. The modularity and lightweight nature of the LLaMA3‐8B model make the system particularly well‐suited for local deployment in clinical environments, where privacy, efficiency, and resource constraints are critical.

### 2.4 Evaluation design

To assess the performance of RTPhy‐ChatBot, we designed a set of 20 template questions covering various domains in radiotherapy physics, including quality assurance (QA), image‐guided radiotherapy (IGRT), respiratory motion management, particle radiotherapy, radiation dosimetry, and equipment‐specific considerations. Table 1 lists all template questions. Each question was submitted to RTPhy‐ChatBot and three commercial LLMs: Gemini‐2.0‐Flash, GPT‐4o, and Moonshot‐v1 (See Table 2). The LLMs were prompted using the instruction: “According to AAPM TG reports, [question]”, to ensure responses were grounded in authoritative guidelines. Each question was asked once. All conversations were conducted in English.

**TABLE 1:** Template questions used for assessment.

Index	Question	Field in radiotherapy physics	Corresponding AAPM Report
**Q1**	In a monthly QA procedure, what is the tolerance of photon beam profile constancy of an IMRT machine?	QA	TG‐142
**Q2**	What are the advantages of kVCT compared to MVCT?	Imaging	TG‐179
**Q3**	What are the low contrast resolution standards for kV‐CBCT, fan beam MVCT, and MV‐CBCT, respectively?	QA	TG‐179
**Q4**	What is the difference between PS, PBS, and US in particle radiotherapy?	Proton/Heavy ion radiotherapy	TG‐290
**Q5**	What are the respiratory movement management methods in photon radiotherapy?	Dosimetry	TG‐76
**Q6**	In radiotherapy, how much dose should the image‐guided process produce? How to reduce the dose of the image guidance process?	Imaging	TG‐75 & TG‐180
**Q7**	In stereotactic radiotherapy, what specific quality assurance programs are available for small‐field dosimetry?	QA	TG‐135 & TG‐155
**Q8**	What are the sources of error in image registration? How to mitigate error?	Imaging	TG‐132
**Q9**	How to measure the dosimetric indices in small fields?	QA	TG‐155
**Q10**	What are the main sources of out‐of‐field doses in radiation therapy? What factors affect the out‐of‐field dose?	Dosimetry	TG‐158
**Q11**	In radiotherapy, how much impact do couches and different kinds of patient immobilization devices have on patient dose in radiation therapy?	Dosimetry	TG‐176
**Q12**	In proton therapy, how to reduce the impact of high‐atomic number materials?	Proton/Heavy ion radiotherapy	TG‐185
**Q13**	During radiotherapy, what is the maximum dose allowed for a pacemaker? During the treatment process, what should be paid attention to?	Dosimetry	TG‐203
**Q14**	What are the limitations of MLC tracking?	Dosimetry	TG‐264
**Q15**	For deep‐inspiration breath hold (DIBH) for breast radiotherapy treatment with bolus, what should we pay attention to when performing surface‐guided radiotherapy?	Dosimetry	TG‐302
**Q16**	In a monthly quality assurance test, what is the tolerance of laser marking accuracy of an MRI simulator?	QA	TG‐284
**Q17**	What are disadvantages of using a constant RBE in proton therapy?	Proton/Heavy ion radiotherapy	TG‐256
**Q18**	What is the difference between analytic calibration model and pose determination model for a C‐arm CBCT system?	QA	TG‐238
**Q19**	What should be paid attention to when using TLDs or OSLs in the measurement of skin dosimetry?	Dosimetry	TG‐191
**Q20**	What is the tolerance of output factors for a monte‐carlo based treatment planning system?	QA	TG‐157

**TABLE 2:** Commercial LLM used for comparison in this study.

LLM	Provider	Online retrieval capability
Gemini‐2.0‐Flash	Google	Yes
GPT‐4o	OpenAI	Yes
Moonshot‐v1	Moonshot	Yes

A panel of three senior medical physicists independently conducted a blinded assessment of the LLM responses. Each response was scored on a 5‐point scale based on correctness, completeness, and relevance. For each response, the final score was calculated as the mean of the three reviewers’ scores. To represent uncertainty, standard deviations across the three ratings were calculated.

To evaluate statistical significance, we used the Mann–Whitney U test to compare scores between RTPhy‐ChatBot and each commercial LLM. We performed comparisons between the three reviewer scores assigned to RTPhy‐ChatBot and those assigned to each of the other LLMs. All statistical analyses were performed using SPSS.^31^

To supplement assessments with quantitative metrics, we employed the Rouge (Recall‐Oriented Understudy for Gisting Evaluation) scores^32^ to evaluate the textual similarity between LLM‐generated responses and a reference standard. For each of the 20 questions, we created a reference standard answer by aggregating the responses from all LLMs and submitting them to Gemini‐2.0‐Flash, which generated an integrated draft. This draft was then manually reviewed and lightly edited by a panel of senior medical physicists to correct factual errors and ensure completeness, while preserving the core structure and phrasing produced by the model. These final answers were designated as the ground truth. All 20 reference answers are included in Supplementary Material B. We then computed Rouge‐1, Rouge‐2, and Rouge‐L scores for each LLM by comparing its response to the ground truth answer for each question:

Rouge‐1 measures the overlap of unigrams (single words),

Rouge‐2 measures the overlap of bigrams (two‐word sequences),

Rouge‐L measures the longest common subsequence between the two texts.

These metrics provide an objective measure of similarity in content and linguistic structure between model outputs and the reference answer. In addition, we evaluated the effect of introducing zero‐shot Chain‐of‐Thought (CoT) reasoning.^33^ For each question, we rephrased the prompt to encourage step‐by‐step reasoning and regenerated answers using each LLM. The LLM's performance before and after the introduction of the zero‐shot CoT was evaluated.

## 3 RESULTS

### 3.1 System overview and user interaction

**FIGURE 2:**
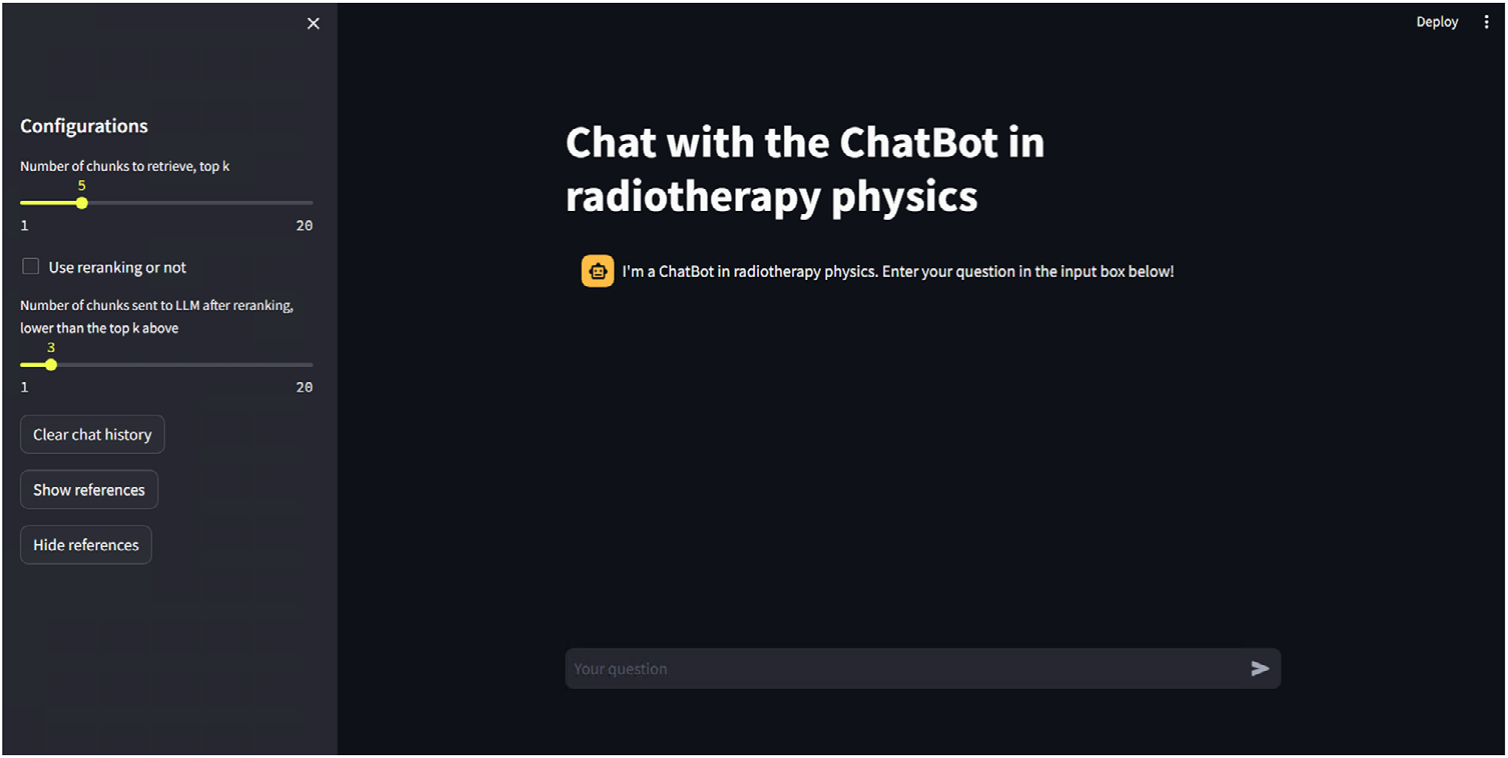
The user interface of the RTPhy‐ChatBot.

Figure 2 showcases the user interface of RTPhy‐ChatBot, a web‐based platform designed for interaction. At the center of the interface is an input box positioned beneath the main display area, where users can enter questions to initiate a conversation with the chatbot. Once a query is submitted, RTPhy‐ChatBot processes it and generates a relevant response within seconds.

The chatbot's reply appears above the input box, clearly formatted for easy reading and comprehension. Each response is tailored to the user's query, offering informative and context‐aware insights drawn from radiotherapy physics.

To further enhance user control and customization, several adjustable configurations are available on the left side of the interface. Uses can modify parameters such as: the number of document chunks retrieved by the retriever, the number of top‐ranked chunks passed to the LLM after reranking, whether to enable or disable the reranking module, and whether to display source references used to generate the response. These configurable settings allow users to tailor the chatbot's behavior to suit different tasks or preferences, improving the transparency, responsiveness, and usability of the system.

### 3.2 Performance comparison with commercial LLMs

We compared the performance of RTPhy‐ChatBot with several commercial LLMs (Gemini‐2.0‐Flash, GPT‐4o, and Moonshot‐v1) across 20 template questions covering four major domains:

QA (Q1, Q3, Q7, Q9, Q16, Q18, Q20): addressed QA standards for devices such as IMRT machines, CBCT systems, MRI simulators, and TPS.

Dosimetry (Q5, Q10, Q11, Q13, Q14, Q15, Q19): focused on small field dosimetry, out‐of‐field dose, motion management, pacemaker dose constraints, MLC tracking, and dosimeter use.

Imaging and Image Guidance (Q2, Q6, Q8): covered imaging modality advantages, image registration error mitigation, and IGRT dose reduction.

Advanced Radiation Therapy Techniques (Q4, Q12, Q17): involved beam delivery types, relative biological effectiveness (RBE), and the dose calculation challenges with high‐Z materials.

Overall, RTPhy‐ChatBot, Gemini‐2.0‐Flash, and GPT‐4o delivered more accurate, guideline‐compliant responses in most categories, while Moonshot‐v1 showed reasonable performance but occasional misunderstandings in complex queries. Notably, RTPhy‐ChatBot exhibited particularly strong performance in questions requiring knowledge of QA standards, with responses closely aligning with AAPM TG recommendations. Supplementary Material A includes the full response set from each model for further review and comparison.

**FIGURE 3:**
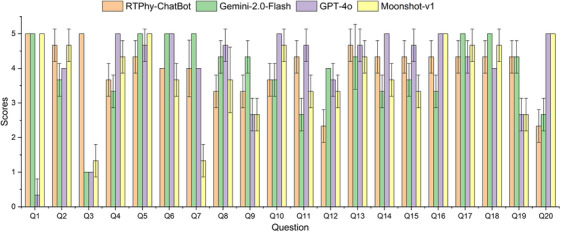
The scores of different LLMs for all template questions.

Table 3 and Figure 3 display the scores assigned by the panel of senior medical physicists, along with the results of the Mann‐Whitney U test for significance. In Figure 3, the height of each bar represents the mean score across the three expert reviewers for each LLM per question, while the error bars depict the standard deviation across the three reviewers. Statistical analysis showed that RTPhy‐ChatBot significantly outperformed commercial LLMs in questions requiring standard answers, such as those involving numerical QA tolerances (e.g., Q3). For open‐ended questions (e.g., Q2 and Q14), RTPhy‐ChatBot performed comparably to the other LLMs, with no significant differences. In some questions – such as Q4, Q6, Q10, and Q12 – GPT‐4o and Gemini‐2.0‐Flash provided more comprehensive answers from multiple perspectives.

**TABLE 3:** The scores and the statistical test results of responses generated by LLMs.

Question ID	RTPhy‐ChatBot	Gemini‐2.0‐Flash		GPT‐4o		Moonshot‐v1	
		Score	*p*‐value	Score	*p*‐value	Score	*p*‐value
Q1	5.0 ± 0.0	5.0 ± 0.0	1.00	0.3 ± 0.5	**0.03**	5.0 ± 0.0	1.00
Q2	4.7 ± 0.5	3.7 ± 0.5	0.10	4.0 ± 0.0	0.11	4.7 ± 0.5	1.00
Q3	5.0 ± 0.0	1.0 ± 0.0	**0.03**	1.0 ± 0.0	**0.03**	1.3 ± 0.5	**0.03**
Q4	3.7 ± 0.5	3.3 ± 0.5	0.46	5.0 ± 0.0	**0.03**	4.3 ± 0.5	0.20
Q5	4.3 ± 0.5	5.0 ± 0.0	0.11	4.7 ± 0.5	0.46	5.0 ± 0.0	0.11
Q6	4.0 ± 0.0	5.0 ± 0.0	**0.03**	5.0 ± 0.0	**0.03**	3.7 ± 0.5	0.32
Q7	4.0 ± 0.8	5.0 ± 0.0	0.12	4.0 ± 0.0	1.00	1.3 ± 0.5	**0.05**
Q8	3.3 ± 0.5	4.3 ± 0.5	0.10	4.7 ± 0.5	0.07	3.7 ± 0.9	**0.80**
Q9	3.3 ± 0.5	4.3 ± 0.5	0.10	2.7 ± 0.5	0.20	2.7 ± 0.5	0.20
Q10	3.7 ± 0.5	3.7 ± 0.5	1.00	5.0 ± 0.0	**0.03**	4.7 ± 0.5	0.10
Q11	4.3 ± 0.5	2.7 ± 0.5	**0.04**	4.7 ± 0.5	0.46	3.3 ± 0.5	0.10
Q12	2.3 ± 0.5	4.0 ± 0.0	**0.03**	3.7 ± 0.5	0.07	3.3 ± 0.5	0.10
Q13	4.7 ± 0.5	4.3 ± 0.9	0.80	4.7 ± 0.5	1.00	4.3 ± 0.5	0.46
Q14	4.3 ± 0.5	3.3 ± 0.5	0.10	5.0 ± 0.0	0.11	3.7 ± 0.5	0.20
Q15	4.3 ± 0.5	3.7 ± 0.5	0.20	4.7 ± 0.5	0.46	3.3 ± 0.5	0.10
Q16	4.3 ± 0.5	3.3 ± 0.5	0.10	5.0 ± 0.0	0.11	5.0 ± 0.0	0.11
Q17	4.3 ± 0.5	5.0 ± 0.0	0.11	4.3 ± 0.5	1.00	4.7 ± 0.5	0.46
Q18	4.3 ± 0.5	5.0 ± 0.0	0.11	4.0 ± 0.0	0.32	4.7 ± 0.5	0.46
Q19	4.3 ± 0.0	4.3 ± 0.5	1.00	2.7 ± 0.5	**0.04**	2.7 ± 0.5	**0.04**
Q20	2.3 ± 0.5	2.7 ± 0.5	0.46	5.0 ± 0.0	**0.03**	5.0 ± 0.0	**0.03**
Total	4.0 ± 0.9	3.9 ± 1.1	0.87	4.0 ± 1.4	0.27	3.8 ± 1.2	0.56

Across all 20 template questions, the average scores were 4.0, 4.0, 3.9, and 3.8 for RTPhy‐ChatBot, GPT‐4o, Gemini‐2.0‐Flash, and Moonshot‐v1, respectively. These findings demonstrate that RTPhy‐ChaBot achieves performance comparable to, and in some domains exceeding, that of leading commercial LLMs.

### 3.3 Rouge‐based quantitative evaluation

Table 4 summarizes the Rouge scores for all models compared to the reference standard answer. RTPhy‐ChatBot achieved Rouge‐1: 0.5127, Rouge‐2: 0.2119, and Rouge‐L: 0.2748. These scores are similar to those of GPT‐4o and Moonshort‐v1. Gemini‐2.0‐Flash achieved the highest Rouge scores across all metrics. This may be partially attributed to the fact that Gemini was used to synthesize the reference answers, resulting in closer alignment in both lexical choice and structure.

**TABLE 4:** Rouge scores for RTPhy‐ChatBot, Gemini‐2.0‐Flash, GPT‐4o, and Moonshot‐v1.

LLM	Rouge‐1	Rouge‐2	Rouge‐L
RTPhy‐ChatBot	0.5127	0.2119	0.2748
Gemini‐2.0‐Flash^a^	0.6095	0.3929	0.4397
GPT‐4o	0.4910	0.1696	0.2398
Moonshot‐v1	0.5390	0.2272	0.2835

^a^ The reference standard answers were synthesized by Gemini‐2.0‐Flash from the responses of all four LLMs and obtained after manual review.

We also compared these results to those reported by Liu et al., who developed RadOnc‐GPT, a fine‐tuned model for radiotherapy plan generation.^15^ Their model achieved Rouge‐1: 0.4341, Rouge‐2: 0.2250, and Rouge‐L: 0.4271. RTPhy‐ChatBot achieved comparable Rouge‐1 and Rouge‐2 scores but slightly lower Rouge‐L, likely due to its use of RAG rather than domain‐specific fine‐tuning. This aligns with Gemini's higher Rouge‐L score and reinforces that fine‐tuning often results in stylistic consistency with the reference output. Overall, the Rouge results support the effectiveness of our RAG‐based approach in generating high‐quality, relevant answers grounded in radiotherapy physics. The reference answers used in this evaluation are provided in Supplementary Material B.

### 3.4 Effect of zero‐shot CoT prompting

Figure 4 presents a comparison of model performance before and after the introduction of zero‐shot CoT prompting. The average scores post‐CoT were: 3.8 ± 1.2, 4.0 ± 1.1, 4.0 ± 1.5, and 3.7 ± 1.5 for RTPhy‐ChatBot, Gemini‐2.0‐Flash, GPT‐4o, and Moonshot‐v1, respectively. These scores were comparable to the pre‐CoT scores, suggesting that CoT prompting did not substantially improve model performance. Some models experienced a slight decline in specific questions:

1. Q3: Gemini‐2.0‐Flash, GPT‐4o, and Moonshot‐v1 failed to provide accurate answers aligned with AAPM TG reports.
2. Q7: Moonshot‐v1 showed marked improvement in both accuracy and detail after CoT.
3. Q11: GPT‐4o omitted discussion of the effects of treatment couches on skin dose and dose distribution.
4. Q15: GPT‐4o overlooked the influence of bolus‐induced light reflection in SGRT. Moonshot‐v1 provided a general, less informative response.

FIGURE 4Comparison of scores of different LLMs before and after the introduction of CoT, (a) for questions Q1‐Q5, (b) for questions Q6‐Q10, (c) for questions Q11‐Q15, (d) for questions Q16‐Q20.

**Figure:**
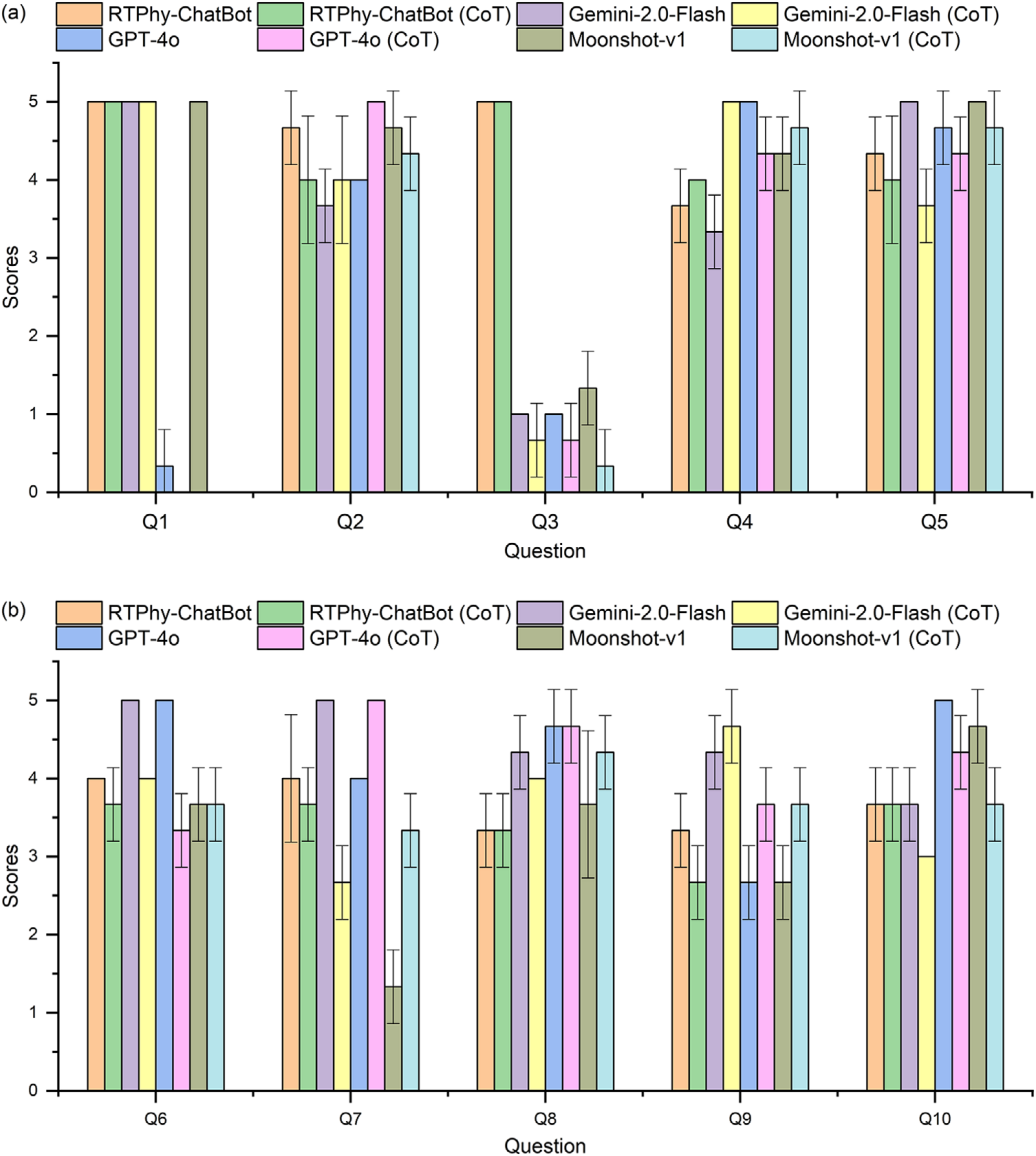

**Figure:**
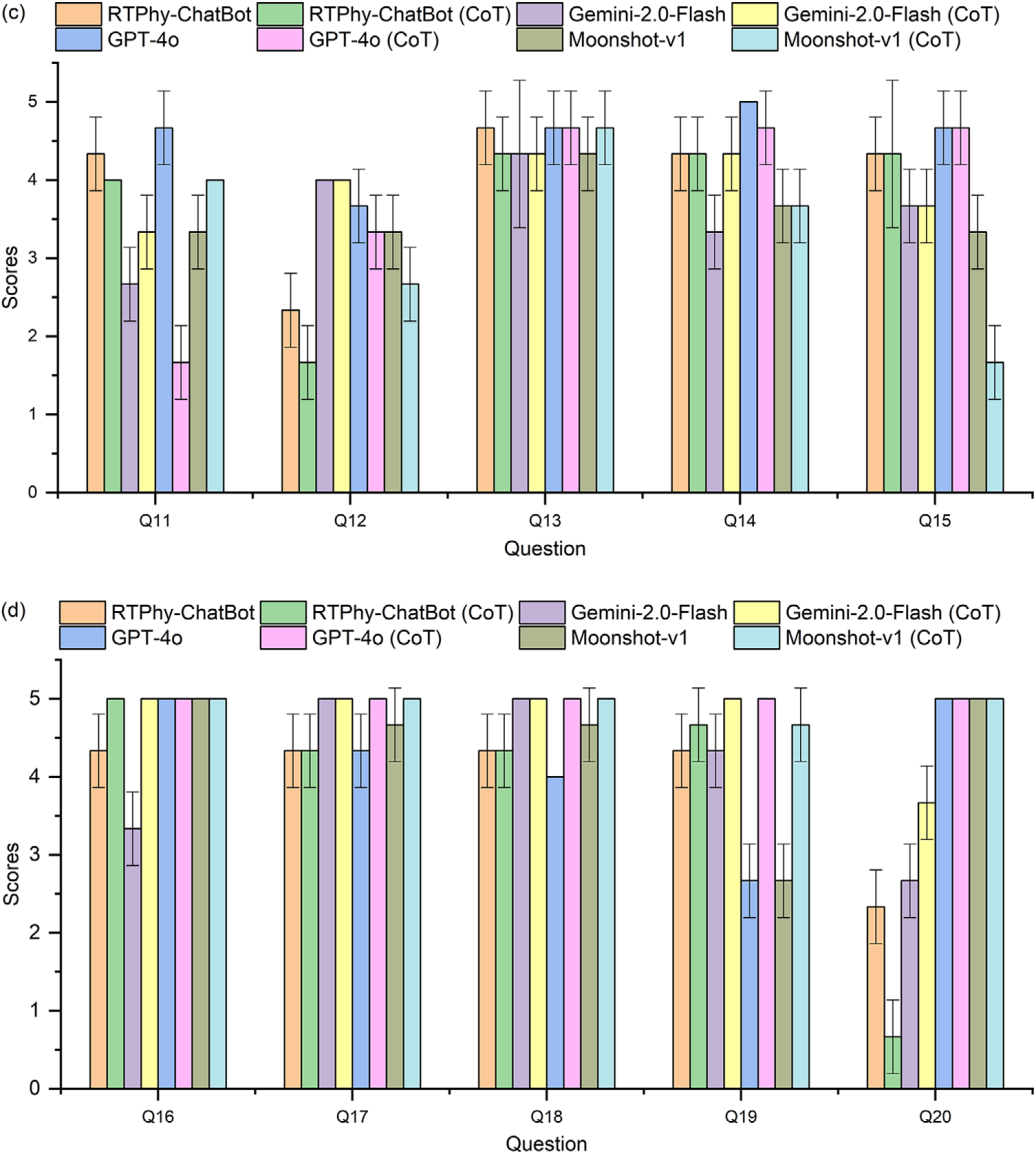

Overall, zero‐shot CoT had a limited benefit for this evaluation. This may be because the questions were primarily factual and relied on information retrieval rather than multi‐step reasoning. As shown in previous research by Sprague et al., CoT is more effective in tasks involving logic or mathematical reasoning than in retrieval‐based question answering.^33^ All CoT‐generated responses are available in Supplementary Material C for detailed examination.

## 4 DISCUSSION

In this study, we developed RTPhy‐ChatBot, an intelligent assistant designed to support medical physicists by leveraging a knowledge base constructed from AAPM publications. Our results demonstrate that RTPhy‐ChatBot effectively utilizes this curated knowledge base to provide accurate, relevant, and reliable responses to domain‐specific queries. Its performance was found to be comparable to, and in some cases better than, that of commercial LLMs such as GPT‐4o.

Unlike commercial LLMs, which often rely on internet search capabilities and publicly available content, RTPhy‐ChatBot draws exclusively from AAPM reports. This approach enhances the relevance and reliability of its response to technical questions in radiotherapy physics. For instance, in questions like Q3, which require specific references to QA standards, RTPhy‐ChatBot produced more accurate and guideline‐consistent answers. However, for questions with broader online coverage (e.g., Q5 and Q13), its performance aligned more closely with that of general‐purpose LLMs.

One limitation of our approach is that RTPhy‐ChatBot currently relies on a static knowledge base, which may exclude the most recent publications or updates. Nevertheless, because the RAG framework allows modular updates, newer documents can be seamlessly added to the knowledge base without retraining the model. Additionally, although RTPhy‐ChatBot achieved strong performance across the 20 template questions tested, radiotherapy physics encompasses a much broader range of clinical and technical issues. Therefore, broader validation is required to confirm the chatbot's effectiveness across the full spectrum of radiotherapy physics.

We selected the Meta‐LLaMA3‐8B‐Instruct model, a relatively small model, to serve as the language model for RTPhy‐ChatBot. While larger LLMs generally outperform smaller models on general‐purpose benchmarks due to their extensive parameters, training scale, and broader pretraining corpora, they also require significantly more computational resources.^34^ By contrast, LLaMA3‐8B offers faster inference, lower hardware demands, and is better suited for clinical environments – especially where data privacy, low latency, and offline operation are essential. When paired with a RAG framework, even a relatively small model like LLaMA3‐8B can achieve domain‐specific performance comparable to that of much larger models. Our findings are consistent with those of Wang et al., who demonstrated that smaller models, when fine‐tuned with radiation oncology data, can match or even exceed the performance of larger LLMs on specialized tasks.^35^

We opted for a RAG‐based architecture rather than fine‐tuning a large model. Fine‐tuning requires extensive annotated datasets and high computational costs, while RAG enables efficient, on‐the‐fly access to a structured knowledge base. RAG is also easier to update—an essential feature in a fast‐evolving domain like radiotherapy physics. While fine‐tuning and RAG serve different purposes—embedding versus retrieving domain knowledge—they can be complementary. In future work, we envision combining RAG with fine‐tuning to improve both the retrieval precision and generative fluency of RTPhy‐ChatBot as the domain‐specific knowledge corpus grows.^24^

In this study, we used direct, concise prompts consisting solely of the problem statement. Although this design allowed for straightforward comparisons, it may have limited the models’ full expressive capabilities. Future research could explore the effects of prompt engineering on model output quality. Regarding CoT reasoning, we employed zero‐shot CoT to test whether step‐by‐step prompting could enhance factual accuracy. However, since most questions in this study required direct information retrieval rather than reasoning, CoT provided limited benefit. Future work could investigate other forms of CoT to assess whether they could yield meaningful improvements.

While AAPM reports are a rich and authoritative source, they may not address every practical question encountered by medical physicists. In daily workflows, physicists often refer to equipment manuals, manufacturer specifications, and internal protocols. Integrating these resources into the knowledge base would significantly improve RTPhy‐ChatBot's ability to solve clinical problems.

Beyond knowledge retrieval, LLMs hold promise for broader clinical applications. Radiotherapy centers generate large volumes of treatment data, which could be used to fine‐tune LLMs for domain‐specific tasks. For instance, LLMs could assist in analyzing target volumes, optimizing dose prescriptions, suggesting beam arrangements, and reviewing treatment plan quality. These capabilities would help automate repetitive tasks, reduce planning time, and support evidence‐based decisions.^36^ As the field evolves, domain‐specialized LLMs will play an increasingly central role in enhancing radiotherapy precision and efficiency.

## 5 CONCLUSION

In this study, we developed and evaluated RTPhy‐ChatBot, a domain‐specific intelligent assistant designed to support clinical and research activities in radiotherapy physics. By leveraging a RAG framework and a knowledge base derived from AAPM reports, RTPhy‐ChatBot provides accurate, referenced responses to a wide range of technical queries. Our comparative evaluation across 20 template questions demonstrated that RTPhy‐ChatBot delivers performance comparable to leading LLMs, particularly excelling in questions requiring precise references to AAPM reports.

This study represents a pilot effort to establish an intelligent assistant tailored to radiotherapy physics. The findings highlight the feasibility and promise of such tools for clinical integration. As future developments incorporate additional publications and explore domain‐specific fine‐tuning, the capabilities and utility of RTPhy‐ChatBot are expected to improve further. Ultimately, the integration of intelligent assistants like RTPhy‐ChatBot has the potential to transform radiotherapy physics practice by providing accurate, efficient, and evidence‐based support to physicists in their daily work, enhancing decision‐making and streamlining clinical workflows.

## AUTHOR CONTRIBUTIONS

**Shuoyang Wei** and **Bo Yang**: paper idea. **Shuoyang Wei** and **Bo Yang**: the source of datasets. **Shuoyang Wei** and **Ankang Hu**: model. **Shuoyang Wei**, **Zhiqun Wang,** and **Xiangying Meng**: assessment of LLMs. **Shuoyang Wei**, **Ankang Hu**, **Zhiqun Wang**, **Xiangying Meng**, **Lang Yu**, **Bo Yang,** and **JieQiu**: writing of the paper.

## CONFLICT OF INTEREST STATEMENT

The authors declare no conflicts of interest.

## Supporting information



Supporting information

Supporting information

Supporting information
