# Supplementary material for: RTPhy‐ChatBot: A RAG‐Based intelligent assistant for radiotherapy physics using LLaMA3 and AAPM reports
Source: J Appl Clin Med Phys. 2025 Oct 9;26(10):e70263. doi: 10.1002/acm2.70263 (PMC12509246; doi:10.1002/acm2.70263)
Supplement: Supplementary file 1 — Supporting information [file ACM2-26-e70263-s001.docx]

Appendix A

Chat history with all LLMs

Questions1 (TG142)

According to AAPM TG reports, in a monthly QA procedure, what is the tolerance of photon beam proﬁle constancy of an IMRT machine?

RTPhy-ChatBot:

According to the context provided, the tolerance for photon beam profile constancy in a monthly QA procedure for an IMRT machine is ±1%. This value represents a deviation from the baseline and not an absolute value. It's important to note that this tolerance may vary depending on the specific requirements of the institution or treatment center.

Additionally, the context mentions that for centers with IMRT QA results unable to meet the recommended tolerance and action limit values, a comprehensive analysis should be performed to determine the sources of differences and remedy them. This can include the use of statistical process control methods, performing TG-119 recommended tests, or conducting independent tests using IROC Houston IMRT phantoms.

It's also worth noting that the tolerance for electron beam profile constancy is ±3%, as per Miften et al.'s report on tolerances and methodologies for IMRT QA. However, this information was not explicitly mentioned in relation to monthly QA procedures.

Therefore, the tolerance for photon beam profile constancy in a monthly QA procedure for an IMRT machine is ±1%, but it's essential to follow the specific requirements of the institution or treatment center and perform comprehensive analysis if necessary to ensure accurate and consistent IMRT delivery.

Gemini-2.0-flash:

According to AAPM Task Group (TG) reports, specifically TG-142: Quality Assurance of Medical Accelerators, the monthly tolerance for photon beam profile constancy of an IMRT machine is 1%.

This means that the measured beam profile should not deviate by more than 1% from the baseline profile established during commissioning. This constancy check is crucial for ensuring accurate and reproducible dose delivery in IMRT treatments.

GPT-4o:

According to the **AAPM Task Group 142 (TG-142)** report, which provides guidelines for quality assurance (QA) of medical accelerators:

**Photon Beam Profile Constancy — Monthly QA Tolerance (IMRT-capable machine)**

- **Test**: **Profile Constancy**
- **Frequency**: **Monthly**
- **Tolerance**: **2%**

**Notes:**

- Profile constancy refers to **the consistency of the lateral beam shape** (i.e., the beam intensity profile across the field) over time.
- It is measured using devices like **ion chamber arrays**, **film**, or **2D detector systems**, and compared to baseline profiles acquired during commissioning or prior QA sessions.
- The goal is to ensure **uniformity of dose delivery**, which is especially critical in **IMRT** where small deviations can impact the cumulative dose distribution.

Moonshot-v1:

According to AAPM TG-142 reports, in a monthly QA procedure, the tolerance of photon beam profile constancy for an IMRT machine is **1%.**

Question2 (TG179)

According to AAPM TG reports, what are the advantages of kVCT compared to MVCT?

RTPhy-ChatBot:

KVCT and MVCT are two different modalities used for image guidance in radiation therapy. Each modality has its unique advantages and disadvantages.

Advantages of kVCT include:

High-resolution images with good contrast, making it ideal for detecting small lesions or subtle changes.

Widely available and well-established technology, with a large user base and extensive clinical experience.

Can be used for both simulation and treatment verification purposes.

Lower dose compared to MVCT, making it a safer option for patients.

Can be integrated into the treatment planning system, allowing for dose calculation based on kVCT images.

Provides anatomical information that can be used to plan treatments more accurately.

Advantages of MVCT include:

Fewer image artifacts due to the absence of bony structures in MVCT images, which can be particularly useful for patients with high-density implants or other metallic devices that cause significant artifacts in kVCT images.

Real-time imaging capability, allowing for online adjustments to treatment parameters or patient position during the treatment session.

It is important to note that the choice between kVCT and MVCT depends on the specific clinical situation and patient needs. Both modalities have their unique advantages and limitations, and a thorough understanding of these factors is essential for making informed decisions about which modality to use in a given case.

Gemini-2.0-flash:

Based on AAPM Task Group (TG) reports, particularly **TG-179: Quality assurance for image-guided radiation therapy utilizing CT-based technologies**, kVCT (Kilovoltage Computed Tomography) offers several advantages compared to MVCT (Megavoltage Computed Tomography) for image-guided radiation therapy (IGRT):

**Advantages of kVCT over MVCT:**

- **Superior Soft Tissue Contrast:** kV photons interact with tissue primarily through photoelectric effect and Compton scattering, resulting in significantly higher contrast between different soft tissues compared to MV photons. MV photons interact predominantly through Compton scattering, which is less dependent on atomic number, leading to poor soft tissue differentiation. This improved contrast in kVCT allows for better visualization of the target volume and surrounding critical structures, especially when bony anatomy is not a reliable surrogate for soft tissue position.
- **Reduced Metal Artifacts in Some Cases:** While both kVCT and MVCT are susceptible to metal artifacts, MVCT can sometimes exhibit more severe artifacts due to the higher energy photons causing more complex scatter patterns. However, this is highly dependent on the specific metal object and imaging parameters. In some scenarios, kVCT might offer slightly reduced artifacts.
- **Lower Imaging Dose:** Generally, kVCT imaging for IGRT can be performed with lower doses compared to MVCT while still providing adequate image quality for patient setup verification. This is a significant advantage for minimizing the overall radiation exposure to the patient.
- **Faster Acquisition Times:** kVCT systems often have faster acquisition times compared to MVCT, which can improve patient comfort and reduce the likelihood of motion artifacts during the scan. This is particularly important for patients who may have difficulty remaining still for extended periods.
- **Integration with Advanced Deformable Image Registration:** The superior soft tissue contrast and potentially fewer artifacts in kVCT make it more suitable for deformable image registration with the planning CT scan (which is also typically a kVCT). Accurate deformable registration is crucial for adaptive radiation therapy workflows involving contour propagation and dose accumulation.
- **Higher Spatial Resolution:** kVCT systems generally offer higher spatial resolution compared to MVCT, allowing for the visualization of finer anatomical details. This can be beneficial for precise patient positioning and target localization.

**In summary, kVCT is generally preferred over MVCT for IGRT due to its superior soft tissue contrast, potentially lower imaging dose, faster acquisition times, and better suitability for deformable image registration, ultimately contributing to more accurate and precise radiation therapy delivery.**

It's important to note that while MVCT has limitations in soft tissue contrast, it can be advantageous in certain situations, such as imaging through dense materials or when metal artifacts are particularly severe in kVCT. The choice between kVCT and MVCT depends on the specific clinical application and the available imaging systems.

GPT-4o:

According to AAPM Task Group reports, including **TG-104**, **TG-179**, and **TG-180**, **kilovoltage CT (kVCT)** and **megavoltage CT (MVCT)** are both used in image-guided radiotherapy (IGRT), but **kVCT has several distinct advantages over MVCT**:

1. Superior soft tissue contrast

kVCT uses lower energy X-rays (~80–140 kVp), which enhances photoelectric interactions, improving contrast between tissues (e.g., soft tissue vs fat). This makes it easier to visualize and register anatomical structures like tumors or organs.

MVCT, operating at megavoltage energies, has lower image contrast because Compton scattering dominates at those energy levels.

2. Lower imaging dose

kVCT typically delivers a **lower radiation dose** to the patient than MVCT, particularly when protocols are optimized.

3. Higher Spatial Resolution

Due to smaller detector elements and finer beam geometry, kVCT provides higher resolution images, allowing for more precise localization of targets and critical structures.

4. Faster imaging time

Modern kVCT systems (especially diagnostic-quality ones) can acquire images more rapidly than MVCT systems, improving workflow efficiency.

5. Better image quality for registration

kVCT images are more compatible with planning CT (also acquired at kV), which improves the accuracy of image registration and fusion for IGRT.

6. Reduced beam hardening artifacts

Compared to MVCT, kVCT shows fewer artifacts from denser materials like dental fillings or prosthetics, improving visualization.

Moonshot-v1:

According to AAPM TG reports and related studies, kVCT (kilovoltage computed tomography) has several advantages over MVCT (megavoltage computed tomography):

1. **Image Quality**
   - **Contrast and Noise**: kVCT generally provides better image contrast and lower noise levels compared to MVCT. This is because kVCT operates at lower energies (typically around 100 kV), resulting in higher contrast and less scatter. For example, in a study comparing ClearRT kVCT and MVCT systems, ClearRT showed significantly lower noise and improved contrast metrics.
   - **Spatial Resolution**: kVCT systems often have better spatial resolution, which helps in more accurate delineation of anatomical structures.
2. **Patient Setup and Target Localization**
   - **Improved Target Visualization**: kVCT’s superior image quality allows for more precise localization of the target volume and critical organs at risk (OARs), which is crucial for accurate patient setup and treatment planning.
   - **Reduced Motion Artifacts**: kVCT can better capture the motion of targets, especially in areas with respiratory motion, compared to MVCT.
3. **Dose**
   - **Lower Dose**: kVCT typically delivers a lower dose to the patient compared to MVCT. This is important in reducing the risk of secondary malignancies, especially in long-term follow-up treatments.
4. **Scan Time**
   - **Faster Scan Times**: kVCT systems generally have faster scan times compared to MVCT systems. For example, ClearRT kVCT can complete scans in a fraction of the time taken by MVCT. This reduces the time patients spend in the treatment room and improves overall workflow efficiency.
5. **Artifacts**
   - **Reduced Artifacts**: kVCT is less prone to artifacts such as “cupping” or “capping” compared to MVCT, which can improve the overall image quality and reliability.

In summary, kVCT offers superior image quality, lower dose, faster scan times, and reduced artifacts compared to MVCT, making it a preferred choice for image-guided radiation therapy (IGRT) and adaptive radiotherapy (ART) applications.

Question3 (TG179)

According to AAPM TG reports, what are the low contrast resolution standards for kV-CBCT, fan beam MVCT, and MV-CBCT, respectively?

RTPhy-ChatBot:

The low contrast resolution requirements for different types of CT imaging systems used in IGRT (Image Guided Radiation Therapy) vary depending on the imaging technique and energy level.

For kV-CBCT (kilovoltage cone-beam computed tomography), the visibility of 1% contrast objects with a diameter of 7 mm has been reported. This means that structures with a difference in linear attenuation coefficient of only 1% compared to their surroundings can still be detected with this imaging modality.

Fan beam MVCT (megavoltage fan-beam computed tomography), on the other hand, can resolve objects with a diameter of 13 mm and density differences of 2% from the background. This is due to the fact that megavoltage beams for imaging suffer from fewer scatter and beam hardening artifacts compared to kV-CBCT, but they inherently cause poorer subject contrast.

MV-CBCT (megavoltage cone-beam computed tomography) can resolve objects with a size of 2 cm and a contrast of 1%. This imaging modality uses a megavoltage beam that is immune to typical high-Z artifacts, making it an attractive option for IGRT applications. The MV-CBCT system consists of an amorphous silicon flat panel adapted for MV imaging attached to a linear accelerator and an integrated workflow application to generate a three-dimensional representation of the patient in the treatment position. The field width is set by the manufacturer at 27.4 cm, and the field length is adjustable to a maximum of 27.4 cm.

It's important to note that these standards may vary depending on the specific implementation and clinical requirements, and regular quality assurance checks should be performed to ensure consistent image quality. The recommended frequency for testing the geometric accuracy and low contrast resolution of these imaging systems is initially monthly, with a 6-month schedule once stability has been demonstrated.

Gemin-2.0-flash:

While AAPM Task Group (TG) reports, particularly **TG-179: Quality assurance for image-guided radiation therapy utilizing CT-based technologies**, discuss image quality parameters for various CT-based IGRT systems, they **do not explicitly define quantitative low-contrast resolution standards or tolerances** in the same way they do for geometric or dosimetric parameters in routine QA.

Instead, TG-179 emphasizes the importance of **establishing baseline image quality at commissioning** and then performing regular checks to ensure that the image quality remains consistent over time. These checks should involve visual inspection of low-contrast phantoms and qualitative assessments.

**In summary, AAPM TG reports, particularly TG-179, do not provide specific quantitative low-contrast resolution standards for kV-CBCT, fan beam MVCT, and MV-CBCT for routine QA.** The emphasis is on establishing a baseline during commissioning using appropriate phantoms and then performing regular qualitative checks to ensure the low-contrast visibility remains consistent over time. Any significant degradation should trigger further investigation.

It's crucial to consult the specific recommendations in AAPM TG-179 and other relevant TG reports (like TG-142 which addresses overall accelerator QA) for a comprehensive understanding of QA procedures for IGRT systems. Vendor-specific guidelines and institutional protocols should also be considered.

GPT-4o:

According to **AAPM TG-179**, **TG-104**, and related task group reports on image guidance and image quality, the **low contrast resolution standards** (or **guidelines**) for different IGRT imaging modalities are as follows:

1. kV-CBCT (Kilovoltage Cone-Beam CT):

**≤5 mm** low-contrast object at ~1% contrast

2. Fan Beam MVCT (Megavoltage Fan-Beam CT):

**~7–8 mm** low-contrast object at ~3% contrast

3. MV-CBCT (Megavoltage Cone-Beam CT):

**>10 mm** low-contrast object at ~5% contrast or worse

Moonshot-v1:

According to AAPM TG reports, the low contrast resolution standards for different imaging modalities are as follows:

**kV-CBCT (Cone Beam Computed Tomography)**

- **Standard**: The low contrast resolution should be able to detect objects with a contrast level of **0.5%** or higher. For example, using the Catphan phantom, the smallest detectable disc diameter at this contrast level should be around **7 mm**.

**Fan Beam MVCT (Megavoltage Computed Tomography)**

- **Standard**: Fan beam MVCT systems typically have better low contrast resolution compared to CBCT. They can detect objects with contrast levels as low as **0.3%**. This means that smaller and lower contrast objects can be more easily visualized in fan beam MVCT images.

**MV-CBCT (Megavoltage Cone Beam Computed Tomography)**

- **Standard**: Similar to kV-CBCT, the low contrast resolution for MV-CBCT should also be able to detect objects with a contrast level of **0.5%**. However, the exact detectability can vary depending on the specific system and imaging conditions.

In summary, fan beam MVCT generally has superior low contrast resolution compared to kV-CBCT and MV-CBCT, allowing for the detection of lower contrast objects.

Question4 (TG290):

According to AAPM TG reports, what is the difference between PS, PBS, and US in particle radiotherapy?

RTPhy-ChatBot:

Particle radiotherapy uses three primary delivery techniques to spread the energetic particle beam for therapeutic applications: passive scattering (PS), pencil beam scattering (PBS), and uniform scattering (US).

Passive Scattering (PS): In PS, one or two high-atomic-number (Z) material scatterers are used to physically spread the beam transversely. The beam enters the scatterer, interacts with its atoms, and exits as a wider beam. This technique is commonly used for ocular or "eye" lines due to its simplicity and ability to provide a uniform dose distribution over a larger area. However, it comes with some disadvantages such as increased beam size, longer treatment times, and potential for increased dose to healthy tissues outside the target volume.

Pencil Beam Scanning (PBS): In PBS, electromagnets are used to sweep the particle beam across the treatment field. The beam remains on during scanning, and the dose at any given location is determined by the beam current and/or the speed of the motion of the beam. This technique offers several advantages over passive scattering, including better control over beam size, improved accuracy, and the ability to deliver modulated doses across the field. However, it also comes with some challenges such as increased complexity, longer treatment times, and potential for increased dose to healthy tissues due to the presence of the magnetic fields.

Uniform Scanning (US): US systems use scanning magnets to distribute a relatively large spot (1r = 6-20 mm at the isocenter in air) across the treatment field at high speed (3-50 Hz). Some US systems use the first scatterer to control the beam spot size, whereas others achieve the desired condition from magnetic optics alone. Uniform scanning patterns include Lissajous, circular, raster, triangle, and spiral configurations. Spot size, scanning frequency, and pattern spacing can vary with beam energy and need to be optimized to meet lateral flatness specifications during the commissioning phase and in turn should be verified for consistency as part of the ongoing QA program. US systems use the method of energy stacking, in which protons of fixed energies deposit dose to individual layers at specific depths, one at a time, in a predefined intensity ratio. To achieve a reasonable overall treatment time, switching from layer to layer quickly is necessary (e.g., less than 1 s).

In summary, passive scattering and pencil beam scattering are methods for physically or electromagnetically spreading the proton beam transversely, while uniform scanning is a method for distributing the beam across the treatment field using magnetic forces. Each technique has its advantages and disadvantages, and the choice between them depends on various factors such as clinical requirements, available technology, and patient-specific considerations.

Gemini-2.0-flash:

According to AAPM Task Group (TG) reports, particularly those focusing on particle therapy like **TG-224: Comprehensive Proton Therapy Machine Quality Assurance** and **TG-290: Respiratory Motion Management for Particle Therapy**, the key differences between Passive Scattering (PS), Pencil Beam Scanning (PBS), and Uniform Scanning (US) in particle radiotherapy (primarily proton therapy) lie in their beam delivery methods and resulting dose distributions:

Here's a breakdown of each technique:

**1. Passive Scattering (PS):**

- **Method:** A broad beam of protons is created by passing a narrow beam through scattering foils and other beam-shaping devices (like apertures and compensators) placed in the beam path. These devices spread out the beam laterally and modulate its depth to conform to the target volume.
- **Dose Distribution:** Results in a relatively uniform dose distribution across the target volume, with a sharp distal fall-off due to the Bragg peak. However, it often leads to a less conformal dose distribution compared to scanning techniques, especially in the lateral direction, as the beam is inherently broad.
- **Advantages:**
  - Historically the first and most established technique.
  - Can be more robust to small setup errors and motion in some situations due to the broader beam.
  - Potentially faster delivery for certain target volumes.
- **Disadvantages:**
  - Less conformal dose distribution, leading to higher integral dose to normal tissues.
  - Limited ability to create complex dose shapes or modulate dose within the target volume.
  - Can generate more secondary neutrons due to the scattering process.

**2. Uniform Scanning (US):**

- **Method:** A narrow proton beam is magnetically scanned across the target volume layer by layer at different depths (energies). The beam intensity is typically uniform within each scanned layer. Range modulation is achieved by varying the energy of the proton beam.
- **Dose Distribution:** Offers improved conformity compared to passive scattering by shaping the dose to the target volume in three dimensions. However, the intensity within each layer is generally uniform.
- **Advantages:**
  - Better conformality than passive scattering, reducing dose to surrounding normal tissues.
  - Greater flexibility in shaping the dose compared to passive scattering.
- **Disadvantages:**
  - Can be more sensitive to motion and setup errors compared to passive scattering.
  - Limited ability for complex dose modulation within the target volume (intensity modulation).

**3. Pencil Beam Scanning (PBS):**

- **Method:** A very narrow ("pencil") beam of protons is precisely scanned across the target volume in three dimensions by rapidly changing the magnetic fields that steer the beam. The intensity (number of protons) and energy of the pencil beam can be varied for each spot, allowing for highly conformal and complex dose distributions, including intensity-modulated proton therapy (IMPT).
- **Dose Distribution:** Provides the most conformal dose distribution, with the ability to sculpt the dose to match the target volume closely and spare adjacent critical structures. It also allows for dose painting within the tumor.
- **Advantages:**
  - Superior dose conformality, minimizing dose to normal tissues and potentially reducing side effects.
  - Ability to create complex dose distributions and perform intensity modulation (IMPT).
  - Potentially lower integral dose compared to scattering techniques.
- **Disadvantages:**
  - Most sensitive to motion and setup errors due to the small beam size. Motion management strategies are crucial.
  - Longer treatment times for large or complex target volumes.
  - Requires sophisticated treatment planning and delivery systems.
  - Can be more susceptible to interplay effects with moving targets if not properly managed.

**In summary, the evolution of particle therapy delivery has moved from the broader, more uniform dose of Passive Scattering to the increasingly precise and conformal Pencil Beam Scanning techniques.** Uniform Scanning represents an intermediate step with improved conformality over PS but without the full intensity modulation capabilities of PBS. The choice of technique depends on the clinical situation, the location and size of the tumor, the proximity of critical structures, and the available technology at the treatment facility. Modern particle therapy centers are increasingly adopting PBS due to its dosimetric advantages.

GPT-4o:

In particle radiotherapy, PS (Passive Scattering), PBS (Pencil Beam Scanning), and US (Uniform Scanning) represent different methods of delivering particle beams (such as proton or heavy ion beams). According to the American Association of Physicists in Medicine (AAPM), these techniques vary in how the particle beam is shaped, modulated, and delivered to the patient. Below is a detailed explanation of each method and their differences:

1. PS (Passive Scattering)

**Description:**

- Uses **scatterers**, **modulators**, and **apertures** to spread and shape the proton or ion beam.
- A **range compensator** (or bolus) is used to match the distal shape of the target.

**⚙️ Key Features:**

- Delivers a **broad, uniform beam** across the target.
- Dose modulation is **mechanical**, not dynamic.
- Beam shaping relies on **patient-specific hardware** (apertures, compensators).

**➕ Advantages:**

- Simpler QA and delivery.
- Historically well-established.

**➖ Disadvantages:**

- **Increased neutron production** due to scattering hardware.
- Less conformal dose compared to scanning.
- Not easily adaptable for intensity modulation (IMPT).

2. PBS (Pencil Beam Scanning)

**✅ Description:**

- A **narrow "pencil-like" beam** is magnetically scanned across the target in a spot-by-spot and layer-by-layer manner.
- The energy of the beam is varied to control depth.

**⚙️ Key Features:**

- Highly **conformal 3D dose distribution**.
- No patient-specific hardware needed.
- Supports **Intensity Modulated Proton Therapy (IMPT)**.

**➕ Advantages:**

- Better sparing of normal tissue.
- Adaptable to complex geometries and moving targets.
- Lower neutron contamination compared to PS.

**➖ Disadvantages:**

- More complex QA and delivery system.
- Sensitive to motion (e.g., respiratory motion).
- Requires **robust optimization** for uncertainties.

3. US (Uniform Scanning)

**✅ Description:**

- A hybrid between passive scattering and pencil beam scanning.
- Uses a **scanned beam**, but instead of spot-by-spot painting like PBS, it delivers a **uniform field layer-by-layer**.

**⚙️ Key Features:**

- Scans laterally with a magnet.
- Uses a **range modulator** to create the spread-out Bragg peak (SOBP).

**➕ Advantages:**

- Better dose control than passive scattering.
- Less hardware-intensive than PS.

**➖ Disadvantages:**

- Still limited compared to PBS in terms of conformality and flexibility.
- Cannot deliver intensity-modulated plans as PBS can.

Moonshot-v1:

According to AAPM TG reports, the differences between PS (Passive Scattering), PBS (Pencil Beam Scanning), and US (Uniform Scanning) in particle radiotherapy are as follows:

**1. Beam Delivery and Dose Distribution**

- **PS (Passive Scattering)**
  - **Beam Delivery**: Uses scatterers and range compensators to shape the beam. The beam is broadened laterally and modulated in depth using physical apertures and range compensators.
  - **Dose Distribution**: Produces a relatively homogeneous dose distribution in the target volume. It is less sensitive to motion due to its continuous delivery method.
- **PBS (Pencil Beam Scanning)**
  - **Beam Delivery**: Uses scanning magnets to direct narrow pencil beams of particles to specific locations within the target volume. The beam is turned off between spots.
  - **Dose Distribution**: Can deliver highly conformal dose distributions, allowing for intensity-modulated proton therapy (IMPT). This method can reduce dose to organs at risk (OARs) but is more sensitive to motion due to its discrete spot delivery.
- **US (Uniform Scanning)**
  - **Beam Delivery**: Similar to PBS but uses continuous scanning of the beam at each energy level. The beam remains on during scanning, and energy switching occurs between different layers.
  - **Dose Distribution**: Provides a lateral dose profile similar to PS but can have different delivery time structures depending on the implementation.

**2. Sensitivity to Motion**

- **PS**: Generally considered the least sensitive to motion due to its continuous delivery method.
- **PBS**: Most sensitive to motion because of its discrete spot delivery and the potential for interplay effects between beam delivery and patient motion.
- **US**: Sensitivity to motion is intermediate between PS and PBS, depending on the specific implementation and energy-switching times.

**3. Clinical Applications and Considerations**

- **PS**: Suitable for treatments where motion is less of a concern. It is often used in fixed-beam or gantry-based systems.
- **PBS**: Ideal for complex target geometries and cases where sparing of OARs is critical. It is more commonly used in gantry-based systems with advanced motion management techniques.
- **US**: Offers a balance between dose conformity and motion sensitivity. It can be used in both fixed-beam and gantry-based systems.

**4. Optimization and Motion Management**

- **PS**: Motion management focuses on reducing the magnitude of motion and ensuring accurate range modulation.
- **PBS**: Advanced optimization techniques, such as robust optimization and 4D optimization, are often used to account for motion uncertainties and improve dose conformity.
- **US**: Motion management strategies are similar to PS, but additional considerations may be needed for energy-switching techniques.

In summary, PS, PBS, and US each have distinct characteristics in terms of beam delivery, dose distribution, and sensitivity to motion. The choice of delivery technique depends on the specific clinical requirements, target geometry, and motion management needs.

Question5 (TG76):

According to AAPM TG reports, introduce the respiratory movement management methods in photon radiotherapy.

RTPhy-ChatBot:

In photon radiotherapy, respiratory motion management is implemented to reduce image artifacts and provide images at specific motion states congruent with those used for treatment delivery. Several techniques are used for managing respiratory motion in photon radiotherapy, including:

1. Motion-encompassing methods: These methods aim to account for the respiratory motion by increasing the planning target volume (PTV) or margin around the tumor to include the expected motion. This approach is commonly used when advanced respiratory management techniques are not available.
2. Breath-hold (BH) techniques: Patients are instructed to hold their breath during treatment delivery, typically for 15-30 seconds. BH techniques can be further categorized into voluntary and involuntary methods. Voluntary methods rely on the patient's ability to cooperate and hold their breath, while involuntary methods use abdominal compression or other devices to help patients hold their breath.
3. Respiratory-gated techniques: These methods synchronize the radiation delivery with the respiratory cycle using various gating techniques such as active breathing control (ABC), 4D CT, or real-time imaging. This approach ensures that the radiation is delivered only during specific phases of the respiratory cycle when the tumor is in a stable position.
4. Forced shallow-breathing methods: Patients are instructed to take shallow breaths during treatment delivery, which can help reduce the magnitude of respiratory motion. This approach may be used as an alternative to BH techniques for patients who cannot hold their breath for extended periods.
5. Respiration-synchronized techniques: These methods adjust the radiation beam based on the patient's respiratory cycle, allowing for continuous delivery during the entire respiratory cycle while minimizing the impact of motion on the target.

The choice of a specific respiratory management technique depends on various factors such as tumor location, size, and motion, as well as the patient's ability to comply with the procedure. The Task Group recommends considering respiratory management techniques if either a greater than 5 mm range of motion is observed in any direction or significant normal tissue sparing can be gained through their use. However, it is important to weigh the benefits of additional imaging information against the potential risks associated with extra patient dose from the imaging methods involved.

Gemini-2.0-flash:

According to AAPM Task Group (TG) reports, particularly **TG-76: The Management of Respiratory Motion in Radiation Oncology** and subsequent reports like **TG-142: Quality Assurance of Medical Accelerators** and **TG-188: Report of AAPM Task Group 188 on Respiratory Motion Management**, several methods are employed to manage respiratory motion in photon radiotherapy. The choice of method depends on factors such as the magnitude and regularity of the motion, the tumor location, the treatment technique, and the patient's ability to cooperate.

Here's an introduction to the common respiratory movement management methods in photon radiotherapy as described in AAPM TG reports:

**1. Motion Encompassing Techniques (Large Margins):**

- **Description:** This is the simplest approach, where a sufficiently large Planning Target Volume (PTV) margin is added around the Clinical Target Volume (CTV) to account for the entire range of respiratory motion.
- **Advantages:** Easy to implement, does not require specialized equipment or patient training.
- **Disadvantages:** Leads to irradiation of a larger volume of healthy tissue, potentially increasing the risk of side effects and limiting dose escalation. Generally less preferred for modern conformal techniques.

**2. Respiratory Gating:**

- **Description:** Radiation beam delivery is synchronized with the patient's breathing cycle. The beam is turned "on" only during a specific, reproducible phase of respiration (e.g., end-expiration or end-inspiration) when the target motion is minimal and consistent.
- **Advantages:** Reduces the volume of irradiated healthy tissue compared to motion encompassing. Can improve dose conformity.
- **Disadvantages:** Increases treatment time as the beam is "off" for a significant portion of the breathing cycle. Requires patient cooperation and a system to monitor the respiratory cycle (e.g., external markers, spirometry). Can be less efficient for irregular breathing patterns.

**3. Breath-Hold Techniques:**

- **Description:** The patient is instructed to hold their breath at a specific phase of respiration (e.g., deep inspiration breath-hold - DIBH) for a short period during beam delivery.
- **Advantages:** Effectively eliminates respiratory motion during treatment. Can significantly reduce the dose to critical organs, especially in thoracic and upper abdominal treatments (e.g., cardiac sparing in left breast cancer).
- **Disadvantages:** Requires significant patient cooperation and the ability to hold their breath consistently. Treatment delivery is often interrupted, increasing overall treatment time. Careful monitoring and coaching are necessary.

**4. Forced Shallow Breathing / Abdominal Compression:**

- **Description:** Techniques aimed at reducing the amplitude of respiratory motion. Forced shallow breathing involves coaching the patient to take smaller, more frequent breaths. Abdominal compression uses a device to restrict the movement of the diaphragm, thereby reducing motion in the upper abdomen and thorax.
- **Advantages:** Can reduce the required PTV margins. May be suitable for patients who cannot tolerate breath-hold.
- **Disadvantages:** May not completely eliminate motion. Abdominal compression can be uncomfortable and may alter the breathing pattern.

**5. Real-Time Tumor Tracking:**

- **Description:** Advanced systems that continuously monitor the position of the tumor (or surrogate markers) in real-time during treatment delivery. The radiation beam is actively steered or gated to follow the moving target.
- **Advantages:** Can potentially deliver highly conformal doses to moving targets while minimizing the irradiation of surrounding healthy tissues.
- **Disadvantages:** Requires sophisticated and expensive technology. Can be complex to implement and QA. Sensitive to the accuracy and latency of the tracking system.

**6. 4D Computed Tomography (4D-CT) for Planning:**

- **Description:** While not a treatment delivery technique, 4D-CT is a crucial imaging modality for characterizing respiratory motion during the treatment planning process. It acquires a series of CT scans correlated with the patient's breathing cycle, allowing visualization and quantification of the target and organ motion.
- **Advantages:** Provides essential information for selecting the appropriate motion management strategy and for accurate treatment planning, including Internal Target Volume (ITV) definition or motion-compensated dose calculation.
- **Disadvantages:** Requires specialized CT scanners and software. Can increase the imaging dose to the patient.

AAPM TG reports emphasize the importance of a comprehensive approach to respiratory motion management, including:

- **Motion Assessment:** Quantifying the magnitude and pattern of respiratory motion using techniques like 4D-CT or fluoroscopy.
- **Strategy Selection:** Choosing the most appropriate motion management technique based on the individual patient and tumor characteristics.
- **Implementation and Monitoring:** Ensuring the chosen technique is implemented correctly and monitoring the patient's breathing during treatment delivery.
- **Quality Assurance (QA):** Establishing and following rigorous QA procedures for all respiratory motion management systems.

In conclusion, AAPM TG reports highlight a range of techniques for managing respiratory motion in photon radiotherapy, each with its own advantages and disadvantages. The goal is to minimize the impact of motion on treatment accuracy and to optimize the therapeutic ratio by improving target coverage and reducing dose to healthy tissues. The trend in modern radiotherapy is towards more sophisticated techniques like gating, breath-hold, and real-time tracking when appropriate, guided by thorough motion assessment using 4D-CT.

GPT-4o:

According to **AAPM Task Group reports** such as **TG-76**, **TG-142**, and **TG-101**, managing **respiratory motion** is essential in **photon radiotherapy**, especially for thoracic and abdominal tumors. Unmanaged motion can cause **target miss** and **increased dose to healthy tissues**.

Below is an overview of the key respiratory motion management strategies in photon radiotherapy:

1. Motion Encompassing Methods

 **What it is**: Captures the full extent of tumor motion during treatment planning and delivery.

 **How**:

- **4DCT** to assess motion and create an **Internal Target Volume (ITV)**.
- Plans are designed to treat the entire volume where the tumor may move.

 **Pros**: Simple and robust.

 **Cons**: Leads to **larger margins** and more dose to healthy tissue.

2. Respiratory Gating

 **What it is**: Radiation is turned on only during a specific phase of the breathing cycle (typically end-exhalation).

 **How**:

- Requires respiratory monitoring (e.g., infrared markers, spirometry).
- Can use external surrogate or internal fiducial.

 **Pros**: Smaller margins, reduces normal tissue dose.

 **Cons**: Longer treatment times, requires **stable and reproducible breathing**.

3. Breath-Hold Techniques

 **What it is**: Patient voluntarily or passively holds their breath during treatment.

 **Types**:

- **DIBH (Deep Inspiration Breath Hold)**: Common in left-sided breast cancer to move the heart away from the chest wall.
- **Active Breath Control (ABC)**: Uses a spirometry-based system to actively control breath-hold.

 **Pros**: Excellent reproducibility, minimal motion.

 **Cons**: Patient cooperation is crucial.

4. Forced Shallow Breathing with Abdominal Compression

 **What it is**: Uses a compression plate to reduce diaphragmatic movement.

 **How**:

- A plate is pressed against the abdomen to limit motion.

 **Pros**: Simple and passive.

 **Cons**: May cause discomfort; not suitable for all patients.

5. Real-Time Tumor Tracking

 **What it is**: Radiation beam tracks the tumor’s motion during breathing.

 **How**:

- Requires **internal fiducials** and real-time imaging (e.g., CyberKnife, or with MLC tracking systems).

 **Pros**: Highest precision.

 **Cons**: Complex and expensive, needs advanced systems and QA.

Moonshot-v1:

According to AAPM TG reports, several respiratory movement management methods are used in photon radiotherapy to address the challenges posed by respiratory motion in patients with thoracic, abdominal, and pelvic tumors. These methods include:

**1. Motion-Encompassing Methods**

- **Description**: These methods account for the entire range of respiratory motion during treatment planning and delivery. They do not actively manage motion but rather design the treatment to encompass the motion.
- **Techniques**:
  - **Slow CT**: Acquiring CT images over several respiratory cycles to account for motion.
  - **Inhalation and Exhalation Breath-Hold CT**: Capturing images at specific respiratory phases to understand motion.
  - **4D CT**: Using time-resolved imaging to capture motion over the respiratory cycle.

**2. Respiratory Gated Techniques**

- **Description**: These methods deliver radiation only during specific phases of the respiratory cycle, typically when the tumor is in a stable position.
- **Techniques**:
  - **External Respiratory Gating**: Using external devices to monitor breathing and trigger radiation delivery.
  - **Internal Respiratory Gating**: Using internal markers or imaging to monitor tumor position and control radiation delivery.

**3. Breath-Hold Techniques**

- **Description**: Patients hold their breath during radiation delivery to minimize motion.
- **Techniques**:
  - **Active Breathing Control (ABC)**: Devices that help patients hold their breath for short periods.
  - **Deep Inspiration Breath Hold (DIBH)**: Patients hold their breath at deep inspiration, reducing motion.

**4. Forced Shallow-Breathing Methods**

- **Description**: Techniques that reduce the amplitude of respiratory motion by encouraging shallow breathing.
- **Techniques**:
  - **Abdominal Compression**: Using belts or boards to restrict abdominal movement.
  - **Metronome Guidance**: Helping patients maintain a regular, shallow breathing pattern.

**5. Respiration-Synchronized Techniques**

- **Description**: These methods synchronize radiation delivery with the respiratory cycle, ensuring that the beam is on only when the tumor is in a specific position.
- **Techniques**:
  - **Real-Time Tumor Tracking**: Using imaging or external markers to track tumor position and adjust the beam in real-time.

**Recommendations**

- **Measurement of Tumor Motion**: AAPM TG-76 recommends measuring tumor motion for each patient when respiratory motion is a concern.
- **Threshold for Motion Management**: If target motion exceeds 5 mm, respiratory motion management is recommended.
- **Quality Assurance**: Respiratory motion management requires additional resources, education, and adherence to QA procedures.

These methods help improve the accuracy of radiation delivery, reduce margins, and spare normal tissues, leading to better treatment outcomes.

.

Question6 (TG75 & TG180)

According to AAPM TG reports, in radiotherapy, how much dose should the image-guided process produce? How to reduce the dose of the image guidance process?

RTPhy-ChatBot:

In radiotherapy, the goal is to ensure effective target localization while minimizing the imaging dose to patients. The imaging dose produced during image-guided radiotherapy (IGRT) can vary depending on the specific procedures used. According to reports by the American Association of Physicists in Medicine (AAPM) Task Group 75 and Task Group 180, recommendations for managing the imaging dose include different methods for its calculation and techniques for reducing it.

Typical organ doses resulting from megavoltage (MV) and kilovoltage (kV) image acquisition procedures have been documented in various studies. According to reports by the American Association of Physicists in Medicine (AAPM) Task Group 75 and Task Group 180, the imaging dose should not exceed 5% of the therapeutic target dose. However, it's important to note that the ALARA (As Low As Reasonably Achievable) principle should always be applied, even if the imaging dose is below this threshold.

To reduce the imaging dose during IGRT, several techniques can be employed:

1. Use of low-dose imaging protocols: Implementing low-dose imaging protocols, such as reducing image acquisition time or using lower kVp settings, can help decrease the amount of radiation exposure to patients without compromising image quality.
2. Adaptive imaging techniques: Utilizing adaptive imaging techniques, like deformable image registration and online adaptive planning, can minimize the need for repeated imaging and reduce overall imaging dose.
3. Dose tracking systems: Implementing dose tracking systems to monitor and record the imaging doses delivered to patients during IGRT sessions can help ensure that the imaging dose remains below acceptable levels.
4. Use of 2D or 3D cone-beam CT (CBCT): CBCT provides high-resolution images, but it also delivers a higher dose compared to other imaging modalities. Using 2D kV imaging instead of CBCT whenever possible can help reduce the imaging dose while maintaining adequate image quality for target localization.
5. Implementing automated image registration: Automated image registration techniques can help improve the accuracy and efficiency of IGRT, reducing the need for repeated imaging sessions and thus decreasing overall imaging doses.
6. Education and training: Ensuring that radiation oncologists, medical physicists, and other healthcare professionals involved in IGRT are well-educated about the importance of managing imaging dose and the available techniques to do so can help promote a culture of ALARA within the radiotherapy community.
7. Continuous improvement: Regularly reviewing and updating IGRT protocols and techniques is essential to minimize imaging doses while maintaining image quality and therapeutic effectiveness. Collaboration between medical physicists, radiation oncologists, and other healthcare professionals can help drive continuous improvement in IGRT practices.

By employing these techniques and following best practices for IGRT, it's possible to minimize the imaging dose while maintaining effective target localization and ensuring high-quality patient care.

Gemini-2.0-flash:

According to AAPM Task Group (TG) reports, particularly **TG-180: Image Guidance Doses Delivered During Radiotherapy: Quantification, Management, and Reduction**, the dose from the image-guided process in radiotherapy should be carefully managed and considered, but there isn't a single "how much dose should it produce" standard. Instead, the focus is on understanding, quantifying, and minimizing this dose according to the **ALARA (As Low As Reasonably Achievable)** principle.

Here's a breakdown of the key points from AAPM TG reports regarding image guidance dose:

**How much dose does the image-guided process produce?**

- **Varies significantly:** The dose delivered during image guidance varies greatly depending on the imaging modality (kV Cone Beam CT (CBCT), MV CBCT, kV planar imaging, MV planar imaging, etc.), the imaging protocol (number of projections, scan length, mAs, kVp, etc.), and the frequency of imaging.
- **Can be a non-negligible fraction of the therapeutic dose:** While historically considered small, with the increasing use of frequent and volumetric image guidance, the cumulative imaging dose over a course of radiotherapy can become a significant fraction of the total therapeutic dose, potentially leading to increased risk of secondary cancers, especially in long-term survivors.
- **TG-180 Recommendation:** AAPM TG-180 recommends a **threshold of 5% of the therapeutic target dose** beyond which the imaging dose should be considered in the treatment planning process and carefully evaluated.
- **Typical Organ Doses:** TG-180 provides typical organ doses resulting from various MV and kV image acquisition procedures to help medical physicists quantify these doses.

**How to reduce the dose of the image guidance process?**

AAPM TG reports, including **TG-180** and **TG-179: Quality assurance for image-guided radiation therapy utilizing CT-based technologies**, outline several strategies to reduce the dose from the image guidance process:

- **Optimize Imaging Protocols:**
  - **Use the lowest clinically acceptable mAs and kVp:** Reducing these parameters directly lowers the radiation output. Image quality should be sufficient for the intended purpose (e.g., bony anatomy matching may require lower image quality than soft tissue visualization).
  - **Minimize the field of view (FOV) and scan length:** Restrict the imaging volume to the region of interest.
  - **Reduce the number of projections/views:** Use the minimum number of images required for accurate patient positioning.
  - **Increase the pitch in helical CT scans:** This acquires data over a longer volume per rotation, potentially reducing dose.
- **Utilize Low-Dose Imaging Modes:** Many modern IGRT systems offer specific low-dose protocols designed for patient setup verification.
- **Limit Imaging Frequency:** Implement imaging schedules based on risk assessment and clinical necessity rather than routine daily imaging for all patients. Consider imaging less frequently as treatment progresses if setup reproducibility is well-established.
- **Cone Beam CT (CBCT) Optimization:**
  - **Use shorter scan times and fewer projections.**
  - **Employ iterative reconstruction algorithms:** These advanced algorithms can produce diagnostic image quality with lower radiation dose compared to traditional filtered back-projection.
  - **Consider half-fan or other dose-reduction techniques if available.**
- **Planar Imaging Optimization:**
  - **Use larger pixel sizes (binning).**
  - **Reduce the number of frames in cine loops.**
  - **Use appropriate filters.**
  - **Minimize magnification.**
- **Consider Surrogate Markers:** When appropriate, use external markers to infer internal target position, potentially reducing the need for frequent volumetric imaging.
- **Image Fusion Strategies:** Utilize deformable image registration to fuse the planning CT with less frequent IGRT images, potentially reducing the need for daily high-dose imaging.
- **Education and Awareness:** Ensure that all clinical staff involved in the IGRT process are aware of the potential imaging doses and the importance of dose reduction strategies.
- **Regular Review of Imaging Protocols:** Periodically review and optimize imaging protocols based on clinical needs and technological advancements to ensure doses are ALARA.
- **Document and Track Imaging Dose:** Implement methods to estimate and record the cumulative imaging dose delivered to each patient.

By implementing these strategies, radiotherapy departments can ensure that the benefits of precise image guidance are achieved while minimizing the associated radiation dose to patients, adhering to the ALARA principle as recommended by AAPM TG reports.

GPT-4o:

According to **AAPM Task Group reports**, especially **TG-75**, **TG-180**, and **TG-179**, the **dose from image-guided radiotherapy (IGRT)** is a critical consideration to **balance localization accuracy and patient safety**.

**According to AAPM TG-75 (2007) and TG-180 (2012):**

- **There is no strict upper limit**, but the **goal is ALARA**: **As Low As Reasonably Achievable**, without compromising imaging quality and localization accuracy.
- **Dose ranges vary by modality**:

kV Imaging (2D Radiographs):

Dose per image: ~0.1 – 0.3 cGy.

kV Cone-Beam CT (kV-CBCT):

Dose per scan: ~1 - 6 mGy, depending on scan parameters (field size, resolution, and scan length).

MV Imaging (2D Radiographs):

Dose per image: ~1 - 5 cGy.

MV Cone-Beam CT (MV-CBCT):

Dose per scan: ~5 - 20 cGy.

To minimize the imaging dose while maintaining image quality and treatment accuracy, the following strategies can be employed:

1. Optimize Imaging Protocols

Use low-dose presets when high image quality isn’t required (e.g., bone alignment).

Limit scan length and reduce mAs or frame numbers when possible.

2. Use Imaging Only When Necessary

Do not overuse daily CBCT unless needed. Instead:

Use less frequent imaging with offline review protocols for certain patients.

Consider plan-of-the-day or adaptive protocols to reduce repeated scans..

3. Choose Lower-Dose Imaging Modalities

kV imaging delivers significantly lower dose and better contrast (especially for soft tissue).

4. Field Size and Region of Interest Limitation

Limit the imaging field size to the target region only, not the whole anatomy.

5. Shielding and Positioning

Use collimators or shielding (if available) to block OARs not needed in the image.

Optimize patient setup to minimize repeated imaging.

6. Technology Upgrades

Newer systems have dose-saving modes, automatic exposure control, and adaptive imaging algorithms.

Moonshot-v1:

According to AAPM TG-180, the imaging dose delivered during image-guided radiotherapy (IGRT) should be carefully managed due to its potential impact on patient safety. The recommended threshold for considering imaging dose in the treatment planning process is **5% of the therapeutic target dose**. This threshold ensures that the additional dose from imaging procedures does not significantly increase the risk of secondary cancers or other radiation-related complications.

**Typical Dose Levels from Different Imaging Modalities**

- **MV Portal Imaging**: Results in the highest dose to anatomical structures, with doses ranging from 3.3 to 4.8 cGy for certain organs.
- **kV-CBCT (kV Cone-Beam CT)**: Delivers lower doses compared to MV portal imaging, with organ doses ranging from 0.04 to 0.42 cGy.
- **kV Radiographs**: Generally result in the lowest imaging doses, with organ doses around 0.07 cGy or less.

**Methods to Reduce Imaging Dose**

1. **Optimize Imaging Techniques**:
   - **Lower Tube Potential (kV)**: Using lower tube potentials can reduce dose while maintaining image quality.
   - **Reduce Tube Current**: Lowering the tube current during imaging can significantly reduce dose, especially in kV imaging.
   - **Intermittent Imaging Modes**: For procedures like CT fluoroscopy, using intermittent modes (e.g., quick check mode) instead of continuous mode can reduce dose.
2. **Adjust Imaging Protocols**:
   - **Limit Scan Range**: Restricting the scan range to only necessary anatomy can reduce unnecessary dose.
   - **Use of Bow-Tie Filters**: Applying bow-tie filters during kV imaging can reduce organ doses by 15-70%.
3. **Patient-Specific Adjustments**:
   - **Patient Weight Considerations**: For patients below 80 kg, lower kV settings (e.g., 90 kV instead of 120 kV) can be used without significantly compromising image quality.
   - **Breath-Hold Techniques**: Using breath-hold techniques during imaging can reduce motion artifacts and allow for lower dose settings.
4. **Advanced Imaging Systems**:
   - **Modern kV Imaging Systems**: These systems generally deliver much lower doses compared to MV imaging, making them preferable for IGRT.
   - **4D CT and Real-Time Imaging**: Using advanced imaging techniques like 4D CT can help in better motion management and dose reduction.

**Conclusion**

Managing imaging dose during IGRT is crucial to balance the benefits of accurate target localization with the risks of additional radiation exposure. By optimizing imaging techniques, adjusting protocols, and using advanced imaging systems, the dose from image-guided procedures can be significantly reduced while maintaining clinical effectiveness.

Question7 (TG135 & TG155)

According to AAPM TG reports, in stereotactic radiotherapy, what specific quality assurance programs are available for small field dosimetry?

RTPhy-ChatBot:

In stereotactic radiotherapy (SRT), the use of small fields and beamlets of less than 10 mm in diameter is common to achieve highly focused and precisely modulated dose distributions. Measuring small photon beams for SRT and intensity-modulated radiation therapy (IMRT) poses challenges due to the loss of lateral electronic equilibrium, volume averaging, detector-interface artifacts, collimator effects, and detector position-orientation effects. To ensure accurate dosimetry in such small fields, an appropriate dosimeter with a spatial resolution of approximately 1 mm or better (stereotactic detectors) is required to measure the basic dosimetry data, such as total scatter factor or relative output factor, tissue-maximum ratio, and off-axis ratios.

Even with stereotactic detectors, careful detector-phantom setup, and detailed dose corrections, one might still find more than 10% discrepancies among the measurements of very small fields (10 mm in diameter). MLC-shaped fields have more geometry and dosimetry uncertainties than those of circular cones.

A comprehensive quality assurance program for small field dosimetry in stereotactic radiotherapy should include:

1. Verification of the mechanical tolerances of the linac, collimator, and stereotactic frame system to ensure accurate positioning and alignment of the treatment fields.
2. X-ray/light field/laser alignment with isocenter using diode detectors or ionization chambers for small fields (≤3 cm × 3 cm) and larger ionization chambers for larger fields (from 3 cm × 3 cm to 10 cm × 10 cm reference field).
3. Verification of the target/tumor with the isocenter prior to treatment using stereotactic imaging techniques such as CT, MRI, or cone-beam CT.
4. Regular calibration and QA of the dosimetry system, including detectors, phantoms, and measurement software.
5. Use of appropriate dosimetric formalisms for small fields, such as the recently proposed dosimetric formalism to a robotic radiosurgery system.
6. Implementation of advanced beam modeling and verification techniques to account for the unique characteristics of small photon beams in SRT.
7. Regular review and analysis of the data from small field dosimetry measurements to identify any trends or discrepancies, and implementation of corrective actions as needed.
8. Continuous monitoring and improvement of the quality assurance program through regular audits, inspections, and training programs for staff involved in small field dosimetry measurements and treatment delivery.

Gemini-2.0-flash:

While AAPM Task Group (TG) reports emphasize the critical importance of accurate small field dosimetry in stereotactic radiotherapy (SRT) and stereotactic body radiotherapy (SBRT) due to the high doses per fraction and small target volumes, they don't outline specific, distinct "programs" solely dedicated to small field dosimetry QA. Instead, they integrate the necessary QA procedures for small fields within broader recommendations for linac QA and specific guidance for SRT/SBRT.

Here's a breakdown of the relevant aspects and how AAPM TG reports address small field dosimetry QA:

**Key AAPM TG Reports Relevant to Small Field Dosimetry QA in SRT/SBRT:**

- **TG-142: Quality Assurance of Medical Accelerators:** This report provides comprehensive QA recommendations for linear accelerators, including specific considerations and tighter tolerances for machines used for SRS/SBRT compared to conventional and IMRT treatments. It emphasizes the need for accurate output calibration, beam profile measurements, and field size verification, all crucial for small fields.
- **TG-106: Accelerator Beam Data Commissioning Equipment and Procedures:** This report focuses on the accurate commissioning of linac beam data, which is foundational for accurate dose calculations in small fields. It provides guidance on selecting appropriate detectors and phantoms for small field measurements.
- **TG-178: Recommendations on the Practice of Calibration, Dosimetry, and Quality Assurance for Gamma Stereotactic Radiosurgery:** ^1^ While focused on Gamma Knife, this report highlights the general principles of QA for intracranial SRT, including the need for precise source calibration and spatial accuracy.
- **TG-155: Megavoltage Photon Beam Dosimetry in Small Fields and Non-Equilibrium Conditions (Report under review by Medical Physics journal as of late 2024):** This highly anticipated report is specifically dedicated to the challenges and best practices in small field dosimetry. It is expected to provide detailed recommendations on appropriate detectors, measurement techniques, and correction factors needed for accurate dosimetry in non-standard conditions encountered in small fields.
- **ACR-AAPM Technical Standard for Medical Physics Performance Monitoring of Stereotactic Body Radiation Therapy (SBRT):** This document, jointly published, provides guidance on QA for SBRT, emphasizing geometric and dosimetric precision and accuracy, which inherently includes small field dosimetry.

**Integrated QA Procedures Relevant to Small Field Dosimetry:**

Within these broader frameworks, the following specific QA procedures are essential for small field dosimetry in SRT/SBRT:

- **Accurate Output Calibration:** TG-142 recommends stringent monthly output constancy checks (typically within 3% for SRS/SBRT). Accurate calibration is paramount as small field doses are highly sensitive to output variations. Reference dosimetry protocols like AAPM TG-51 and its addenda are crucial.
- **Small Field Output Factors (SFOFs) Measurement and Verification:** Unlike large fields, SFOFs in small fields are significantly affected by factors like source occlusion and detector size. TG reports emphasize the need for careful measurement of SFOFs using appropriate small detectors (e.g., small ion chambers, diodes, microdiamond detectors, film) and phantoms. Consistency checks of SFOFs should be performed regularly.
- **Beam Profile Measurements:** Accurate measurement of beam profiles (lateral and depth) is critical for ensuring correct target coverage and dose gradients in SRT/SBRT. TG-142 recommends tight tolerances for beam flatness and symmetry. Small field profiles should be measured with high spatial resolution detectors.
- **Field Size Verification:** Precise verification of small field sizes defined by collimators (cones or MLCs) is essential. This includes mechanical checks and radiation field size measurements using film or other suitable detectors. TG-142 provides tolerances for light/radiation field coincidence, which becomes more critical for small fields.
- **Treatment Planning System (TPS) Validation for Small Fields:** The accuracy of the TPS in calculating doses for small, non-standard fields must be rigorously validated during commissioning and periodically. This involves comparing TPS calculations with independent measurements in phantoms using SRT/SBRT treatment plans. TG-106 and guidelines on TPS commissioning are relevant here.
- **End-to-End Testing:** Comprehensive end-to-end tests that simulate the entire clinical SRT/SBRT process, from imaging to treatment delivery, are crucial. These tests should include dosimetric verification of the planned dose distribution in phantoms using small fields.
- **Detector Calibration and Correction Factors:** Accurate dosimetry in small fields requires careful calibration of detectors and the application of appropriate correction factors to account for factors like volume averaging, perturbation effects, and energy dependence. TG-155 is expected to provide detailed guidance on this.
- **Regular QA of Imaging Modalities:** Given the image-guided nature of SRT/SBRT, rigorous QA of the imaging systems (CT, CBCT, etc.) used for target localization and verification is essential for overall treatment accuracy. This indirectly supports accurate small field dosimetry by ensuring correct target definition and beam alignment.

**In conclusion, while there aren't standalone "programs" specifically labeled for small field dosimetry QA in AAPM TG reports for SRT, the critical aspects of small field dosimetry are thoroughly integrated into the recommendations for linac QA (TG-142), commissioning (TG-106), and specific guidance for stereotactic treatments (e.g., TG-178 and the upcoming TG-155). The emphasis is on using appropriate detectors and measurement techniques, adhering to tighter tolerances, and performing comprehensive end-to-end testing to ensure the accuracy and safety of these high-precision treatments.** The forthcoming TG-155 is expected to consolidate and expand on these recommendations, providing a more focused guide for small field dosimetry QA.

GPT-4o:

**✅ AAPM TG-155: *"Small Field Dosimetry"***

- The **most comprehensive guide** on small field dosimetry QA.
- Provides recommendations for:
  - Detector selection,
  - Beam data commissioning,
  - Output factor measurements,
  - Measurement corrections.

**✅ AAPM TG-101: *"Stereotactic Body Radiation Therapy (SBRT)"***

- Provides clinical QA guidelines specific to SBRT, including **dosimetric precision** for small fields.
- Emphasizes **end-to-end testing**, **high-resolution dosimetry**, and **strict tolerances**.

**✅ AAPM TG-142: *"QA of Medical Accelerators"***

- Covers **routine QA** (daily, monthly, annual) and recommends tighter tolerances for SRS/SBRT systems.

**🎯 Specific QA Programs and Recommendations**

**1. Commissioning and Output Factor Measurement**

- Use **small field-appropriate detectors**:
  - **Diodes (shielded/unshielded)**, **plastic scintillators**, or **micro-ion chambers** (with corrections).
- Account for **volume averaging**, **detector size**, and **stem effects**.
- Use **correction factors** from **IAEA TRS-483**, which complements TG-155.

**2. End-to-End Testing**

- Perform with **anthropomorphic phantoms** and **film/3D dosimeters** (e.g., radiochromic film, gel, or polymer dosimeters).
- Simulates entire treatment chain from **simulation to delivery**.
- Measures targeting accuracy and absolute dose.

**3. MLC and Collimator QA**

- **Leaf positioning accuracy** is critical.
- Use high-resolution methods (e.g., film, log file analysis).
- TG-101 suggests **<1 mm** positioning accuracy for small fields.

**4. Daily/Monthly QA (per TG-142 with SRS addendum)**

- **Daily**: Winston-Lutz test or similar for isocenter verification.
- **Monthly**: Output constancy checks with a **small field phantom**, using a high spatial resolution detector.

**5. Dose Calculation Algorithm Validation**

- Validate TPS algorithms (e.g., AAA, Acuros, Monte Carlo) for **small field accuracy**.
- Cross-check with measured data.

Moonshot-v1:

**Specific QA Programs for Small Field Dosimetry in Stereotactic Radiotherapy (SRT) and Stereotactic Body Radiotherapy (SBRT)**

**QA Programs and Guidelines**

- **TG-101 and TG-142 Reports**: These AAPM task group reports provide comprehensive guidance on quality assurance (QA) for SBRT, including small field dosimetry. TG-101 emphasizes the need to validate dose calculation algorithms for accuracy, especially in heterogeneous media.
- **TG-178 Report**: Specifically for Gamma Knife Stereotactic Radiosurgery (GSR), this report provides detailed protocols for dose rate calibration and implementation data for ionization chambers commonly used in GSR dosimetry.

**Specific QA Measures for Small Field Dosimetry**

- **Dose Rate Calibration**: TG-178 recommends specific protocols for dose rate calibration to ensure accurate dose output from Gamma Knife devices.
- **Mechanical and Dosimetric Checks**: The report also describes routine mechanical, dosimetric, and safety checks for GSR equipment and provides QA recommendations for the treatment process.
- **Special Considerations for Small Fields**: In SBRT, special attention is needed for dose calculation algorithms in heterogeneous media and validation of small field dosimetry.

**Implementation Recommendations**

- **Standard Phantoms and Measurement Devices**: QA programs should include the use of standard dosimetry phantoms and measurement devices, such as ionization chambers and semiconductor detectors, to verify the accuracy of small field dosimetry.
- **Regular Calibration and Validation**: Regular calibration and validation of equipment are essential to ensure accurate and consistent dose output.
- **Patient-Specific QA**: For each patient, specific QA checks should be performed, including validation of dose calculations and review of treatment plans.

**Documentation and Reporting**

- **Recording and Reporting QA Results**: All QA results should be meticulously documented and reported as needed. This helps in tracking equipment performance and taking timely action if issues arise.

By implementing these QA programs, the accuracy and safety of small field dosimetry in stereotactic radiotherapy can be ensured, thereby improving treatment outcomes and patient safety.

Question8 (TG132)

According to AAPM TG reports, what are the sources of error in image registration? How to mitigate error?

RTPhy-ChatBot:

Image registration is a crucial step in various medical imaging applications such as radiation therapy, image-guided surgery, and diagnostic imaging. However, it is susceptible to errors that can have significant consequences for patient treatment or evaluation. In this response, we will discuss the sources of error in image registration and potential mitigation strategies.

Sources of Error:

1. Input: The quality and extent of individual imaging studies can affect the accuracy of their registration with other studies. Extent differences, scan parameters, and image quality are some factors that can introduce errors. For instance, significant differences in physical extent between two studies can limit the volumes available for registration and evaluation of accuracy. Scan parameters such as voxel size and resolution can also impact the integrity of images and require resampling or manipulation to match other studies.
2. Algorithm: The choice of registration algorithms and their limitations can introduce errors. For example, models with limited capabilities may struggle to register complex changes accurately. Incorrect selection of registration algorithm parameters can also lead to errors.
3. Output: The use and interpretation of the registration results can be a source of error. Improper interpolation or extrapolation of the registration field of view can cause errors. Invalid use of the registration results for downstream processes such as contour propagation, dose accumulation, or image guidance can also introduce errors.

Mitigation Strategies:

1. Input: To mitigate errors due to input, it is essential to ensure that imaging studies are of high quality and have consistent scan parameters. Preprocessing steps such as normalization, resampling, and registration can help reduce differences in extent and resolution between studies. Additionally, using automated methods for identifying and selecting fiducial points can improve the accuracy and reproducibility of registration.
2. Algorithm: To mitigate errors due to algorithms, it is crucial to choose appropriate registration algorithms based on the imaging modalities and expected changes. Using multiple registration algorithms in a hierarchical or consensus manner can help reduce errors by combining the strengths of different methods. Additionally, using deformable registration techniques that model complex anatomical changes can improve accuracy.
3. Output: To mitigate errors due to output, it is essential to validate the registration results using appropriate evaluation metrics and tolerances. For example, point-based accuracy metrics such as target registration error (TRE), mean distance to agreement (MDA), dice similarity coefficient (DSC), and consistency can be used to assess registration accuracy. Additionally, using quality assurance procedures and processes can help ensure that the registration results are free of spatial distortions and preserve overall image quality.

Commissioning: As outlined in the context provided, commissioning and routine procedural quality assurance checks are essential for validating post-transfer image data quality and spatial integrity, image orientation, chirality, registration accuracy, and other system functionality. The exact design of the commissioning process and routine QA tests will depend on individual system features and institutional goals. However, the overall goal should be to ensure that images used for treatment planning, delivery, and evaluation are accurately registered with remaining uncertainties accounted for, free of spatial distortions, and that the overall image quality is preserved through various image manipulations.

Gemini-2.0-flash:

According to AAPM Task Group (TG) reports, particularly **TG-132: Use of Image Registration and Fusion Algorithms and Techniques in Radiotherapy**, several sources of error can affect the accuracy of image registration in radiotherapy. Understanding these sources is crucial for implementing effective mitigation strategies.

**Sources of Error in Image Registration:**

- **Image Artifacts:**
  - **Metal artifacts:** Caused by high-density objects like implants, dental fillings, or surgical clips, these can distort the image and hinder accurate registration, especially in CT and CBCT.
  - **Motion artifacts:** Patient movement during image acquisition can lead to blurring or ghosting, making it difficult to align images accurately. This is particularly relevant for thoracic and abdominal imaging.
  - **Noise:** High levels of noise in the images can obscure anatomical details and reduce the accuracy of registration algorithms.
  - **Incomplete or inconsistent field of view (FOV):** If the images being registered do not cover the same anatomical extent, accurate registration in the overlapping regions might be compromised, and extrapolation outside the FOV can introduce significant errors.
  - **Variations in image quality:** Differences in contrast, resolution, and slice thickness between the images being registered can negatively impact the performance of registration algorithms.
- **Anatomical Changes:**
  - **Inter-fractional changes:** Changes in patient anatomy between imaging sessions (e.g., weight loss, tumor shrinkage/growth, organ filling) can make rigid registration inaccurate. Deformable registration attempts to account for these changes but can still introduce errors if the deformation is complex or rapid.
  - **Intra-fractional motion:** Residual motion not completely accounted for by immobilization or motion management techniques can lead to registration errors.
  - **Surgical changes:** Post-operative anatomy can be significantly different from pre-operative scans, posing challenges for registration.
- **Algorithm Limitations and Parameter Selection:**
  - **Choice of registration algorithm:** Different algorithms (rigid, affine, deformable) have varying capabilities and assumptions about the nature of the anatomical differences. An inappropriate choice can lead to inaccurate results.
  - **Suboptimal parameter selection:** Registration algorithms often have numerous parameters that need to be tuned correctly for the specific clinical application and image characteristics. Incorrect parameter settings can lead to suboptimal registration.
  - **Limitations of deformable registration:** While powerful, deformable registration algorithms can be sensitive to noise, artifacts, and large deformations, potentially leading to physically implausible or inaccurate deformation fields.
- **Contouring Variability:** If registration relies on aligning pre-defined contours, inconsistencies in contouring between different image sets or observers will directly translate to registration errors.
- **Feature Selection:** Algorithms that rely on specific image features for registration can be affected by the choice and robustness of these features.
- **Interpolation and Extrapolation:** Image registration often involves interpolation to resample images onto a common grid. Errors can be introduced during this process, especially with large deformations or significant differences in image resolution. Extrapolation of deformation fields outside the imaged volume is unreliable.

**How to Mitigate Error:**

AAPM TG reports recommend several strategies to mitigate errors in image registration:

- **Optimize Image Acquisition:**
  - Employ techniques to minimize motion artifacts (e.g., respiratory gating, breath-hold, patient coaching, shorter scan times).
  - Reduce metal artifacts using appropriate CT acquisition parameters (e.g., metal artifact reduction algorithms) or by carefully positioning the patient.
  - Acquire images with sufficient spatial resolution, contrast, and FOV relevant to the clinical task.
  - Ensure consistent image quality across different time points.
- **Careful Algorithm Selection and Parameter Tuning:**
  - Understand the principles and limitations of different registration algorithms.
  - Select the most appropriate algorithm (rigid, affine, or deformable) based on the expected anatomical changes.
  - Optimize algorithm parameters through validation studies and clinical experience.
- **Rigorous Validation and Quality Assurance (QA):**
  - Establish comprehensive QA procedures for image registration software, including commissioning and regular performance monitoring.
  - Use phantoms with known transformations to quantitatively assess registration accuracy.
  - Visually inspect registration results for plausibility and accuracy, especially for deformable registration.
  - Utilize independent methods (e.g., manual landmark matching) to verify registration accuracy in clinical cases.
  - Employ quantitative metrics (e.g., target registration error, Dice similarity coefficient for overlapping structures) to evaluate registration quality.
  - Follow the recommendations outlined in AAPM TG-132 for validation and QA of image registration.
- **Clinical Workflow Considerations:**
  - Clearly define the clinical goals of image registration and the required accuracy.
  - Provide clear instructions and training to all users on the proper use of registration tools.
  - Establish protocols for reviewing and accepting registration results.
  - Account for residual registration uncertainties in treatment planning margins (e.g., PTV).
- **Motion Management Strategies:** Implement effective motion management techniques to minimize intra-fractional motion, thereby improving the accuracy of registration between planning and treatment delivery images.
- **Contour Propagation and Verification:** When using deformable registration to propagate contours, carefully review and edit the propagated contours to account for anatomical changes and registration inaccuracies.

By understanding the sources of error and implementing these mitigation strategies, radiotherapy departments can improve the accuracy and reliability of image registration, ultimately leading to more precise and effective cancer treatments. The recommendations in AAPM TG reports, particularly TG-132, provide a valuable framework for establishing and maintaining a robust image registration process.

GPT-4o:

According to **AAPM Task Group reports**, especially **TG-132** (focused on image registration and fusion), **image registration** is vital in radiotherapy for **aligning planning and treatment images**, and errors here can directly impact dose delivery.

Sources of Error in Image Registration (TG-132)

Image registration can be rigid (translation/rotation) or deformable (non-rigid, anatomical warping). Errors can arise from several sources:

1. Image Quality Issues

Low resolution

Noise or artifacts (e.g., metal implants)

Motion blurring (especially in 4DCT, CBCT)

2. Inconsistent Patient Positioning

Differences in immobilization or breath-hold state

Anatomical changes (e.g., bladder filling, tumor shrinkage)

3. Modality Differences

CT vs MRI vs PET all have different contrast mechanisms.

Misalignment due to different imaging geometries or distortions (especially in MRI).

4. Algorithm Limitations

Poor performance in low-contrast or complex anatomy areas.

Deformable registration may introduce unrealistic deformations if not properly constrained.

5. User-Dependent Factors

Manual landmark selection errors

Inconsistent contouring during validation

6. Interpolation & Resampling Errors

Data interpolation may distort voxel values.

Resampling can lead to loss of resolution or introduction of grid artifacts.

________________________________________

How to Mitigate Image Registration Errors (TG-132 Recommendations)

1. Use High-Quality Imaging

Ensure good image resolution and signal-to-noise ratio.

Minimize artifacts (e.g., metal artifact reduction techniques).

2. Standardize Patient Setup

Use consistent immobilization devices across imaging sessions.

Apply breath-hold or gating when necessary (e.g., for lung or liver).

3. Perform Modality-Specific Corrections

For MRI: correct for geometric distortions using calibration phantoms.

For PET/CT: ensure attenuation correction is properly aligned.

4. Choose the Right Algorithm

Use rigid registration when only translation/rotation is expected.

Use deformable registration for anatomical changes, but always validate output.

Prefer validated, commercial or peer-reviewed algorithms.

5. Perform Visual and Quantitative QA

Always review registration overlays.

Use fiducial markers, landmarks, or contours for validation.

Quantitative metrics: Dice similarity coefficient, Hausdorff distance, etc.

6. Use QA Tools and Phantom Testing

Perform phantom-based registration QA periodically.

Test deformable algorithms on known deformation phantoms.

7. Document and Audit Registration

Follow institutional policies for review and approval of registrations.

Maintain a log of manual overrides or adjustments.

Moonshot-v1:

According to AAPM TG reports and related studies, the sources of error in image registration and methods to mitigate these errors are as follows:

**Sources of Error in Image Registration**

1. **Geometrical Distortion in Preoperative Images**:
   - **Magnetic Field Inhomogeneity**: In MRI, static magnetic field inhomogeneities and gradient nonlinearities can cause geometrical distortions. These can be corrected using shims and electronic compensation circuits.
   - **Metal Artifacts**: Metals, especially ferromagnetic materials, can cause severe local warping of the static field. Common sources include dental fillings, implants, and shrapnel.
   - **Susceptibility Differences**: Objects with different magnetic susceptibilities (e.g., air-tissue interfaces) can induce perturbations in the static magnetic field, leading to distortions.
2. **Error Inherent in the Registration Process**:
   - **Image Quality**: Noisy images, movement artifacts, and distortions can affect registration accuracy.
   - **Missing Correspondences**: Pathological differences between images can lead to missing correspondences, complicating accurate alignment.
   - **Contrast Differences**: Variations in image contrast can mislead registration algorithms.
   - **Modeling Assumptions**: Incorrect assumptions in the registration model or optimization parameters can result in errors.
   - **Homogeneous Regions**: Lack of distinct features in homogeneous regions can hinder accurate local deformations.
3. **Intraoperative Brain Deformation**:
   - **Physiological Changes**: Variations in water content or anatomical changes due to treatment (e.g., lung tissue inflammation, tumor shedding) can affect registration accuracy.

**Methods to Mitigate Error**

1. **Improve Image Quality**:
   - **Use of Higher Gradient Strengths**: Increasing the frequency-encoding gradient strength in MRI can reduce spatial errors caused by susceptibility differences.
   - **Artifact Reduction Techniques**: Employing techniques to minimize metal artifacts and susceptibility-induced distortions can improve image quality.
2. **Enhance Registration Algorithms**:
   - **Hybrid Approaches**: Combining image-based registration with mechanical modeling techniques can improve accuracy, especially in regions with significant deformations.
   - **Multi-Metric Registration**: Using multiple metrics (e.g., mean distance to agreement, DICE similarity coefficient, Jacobian determinant) to evaluate and refine registration performance.
3. **Regular Quality Assurance**:
   - **Routine Calibration and Validation**: Regularly calibrate and validate imaging and registration systems to ensure consistent performance.
   - **Patient-Specific Adjustments**: Tailor registration protocols to individual patient needs, considering anatomical variations and potential sources of error.
4. **Advanced Imaging Protocols**:
   - **Real-Time Imaging**: Utilizing real-time imaging techniques to account for intraoperative changes and improve registration accuracy.

By addressing these sources of error and implementing the suggested mitigation strategies, the accuracy and reliability of image registration in clinical applications can be significantly enhanced.

Question9 (TG155)

According to AAPM TG reports, how to measure the dosimetric indices in small fields?

RTPhy-ChatBot:

Measuring dosimetric indices in small fields presents several challenges due to the lack of lateral electronic equilibrium, overlap of penumbrae, and change in energy spectrum. To address these issues, various recommendations have been made in the literature.

Firstly, it is important to note that detectors used for dosimetric measurements should be appropriate for small fields. Many conventional ionization chambers are too large and cannot resolve penumbra widths of the beam profiles. Instead, microion chambers or small solid-state detectors with minimum energy, dose, and dose rate dependency are recommended. However, it is essential to evaluate the signal-to-noise issue and perturbation factor of these detectors.

Secondly, for the determination of absorbed dose in small fields, a fine calculation grid size (≤1 mm) should be used. Independent checks, such as measurements or external audits, are also recommended to ensure accuracy. An end-to-end test from CT simulation through treatment delivery can also be performed for verification.

Thirdly, for the measurement of field output factors in small fields, the intermediate field method is proposed in IAEA TRS-483. This method involves using an ionization chamber down to a field size where the detector boundaries are still at least a distance r LCPE away from the field edge and then using a suitable small solid-state detector for smaller fields.

Lastly, it is important to consider the use of appropriate detectors and measurement setups, as well as TPS calculations for small fields. The choice of detectors should include more than one appropriate option, and the setup should be designed to minimize perturbations and ensure accurate centering of the detector. Additionally, a fine calculation grid size should be used for small-field applications.

Overall, measuring dosimetric indices in small fields requires careful consideration of detector choice, measurement setup, and verification methods to ensure accuracy and account for the unique challenges presented by these fields.

Gemini-2.0-flash:

According to AAPM Task Group (TG) reports, particularly the highly relevant **TG-155: Megavoltage Photon Beam Dosimetry in Small Fields and Non-Equilibrium Conditions**, measuring dosimetric indices in small fields requires careful consideration due to several challenges:

- **Loss of Lateral Charged Particle Equilibrium (LCPE):** In small fields, the lateral range of secondary electrons may be larger than the field dimensions, leading to a loss of CPE at the central axis and within the field. This affects the dose distribution and the response of detectors.
- **Source Occlusion:** The finite size of the radiation source and the collimating devices can lead to variations in the effective source position and penumbra characteristics as field size changes.
- **Detector Volume Averaging:** The physical size of the detector can average out the steep dose gradients present in small fields, leading to inaccurate readings, especially at the edges.
- **Perturbation Effects:** Detectors themselves can perturb the radiation field, and these effects can be more pronounced in small, non-equilibrium conditions.

Given these challenges, AAPM TG reports recommend the following approaches for measuring dosimetric indices in small fields:

**1. Detector Selection:**

- **Small Volume Detectors:** Using detectors with small sensitive volumes is crucial to minimize volume averaging. Suitable detectors include:
  - **Small Ionization Chambers:** Pinpoint chambers with volumes around 0.01-0.03 cm³ are often used. However, their perturbation factors can be significant and energy-dependent in small fields.
  - **Semiconductor Diodes:** These have very small active volumes and high sensitivity, making them suitable for profile measurements. However, they can exhibit energy dependence and dose-rate dependence.
  - **Diamond Detectors:** Synthetic single-crystal diamond detectors offer near tissue equivalence, small size, and high radiation hardness, making them attractive for small field dosimetry.
  - **Plastic Scintillators:** These are water-equivalent and have small sensitive volumes, offering potential advantages in small field measurements.
  - **Radiochromic Film:** Film offers high spatial resolution and can be useful for 2D dose distribution measurements and profile verification, but requires careful calibration and handling.

**2. Measurement Techniques:**

- **Output Factors (OFs):**
  - Measure OFs relative to a reference field (typically 10x10 cm²).
  - Apply appropriate correction factors to account for detector perturbation and volume averaging. TG-155 and IAEA TRS-483 provide guidance on these corrections.
  - Consider using multiple detectors and comparing results.
- **Percentage Depth Dose (PDD) and Tissue Maximum Ratio (TMR):**
  - Use small volume detectors and ensure accurate positioning.
  - Account for the effective point of measurement (EPoM) of the detector, which may shift with field size.
  - Compare measurements with Monte Carlo simulations or published data when available.
- **Beam Profiles:**
  - Use detectors with high spatial resolution (e.g., diodes, film, small ion chambers).
  - Ensure sufficient step size during scanning to capture the steep dose gradients in the penumbra region accurately.
  - Correct for detector volume averaging effects, especially for very small fields.
- **Dosimetric Field Size:**
  - Determine the field size based on a specific isodose level (e.g., 50% of the central axis dose at a reference depth).
  - Use high-resolution profiles to accurately determine the field edges.

**3. Correction Factors:**

- AAPM TG-155 emphasizes the importance of applying correction factors to detector readings in small fields to obtain accurate dose measurements. These factors account for:
  - **Volume Averaging Correction (kv):** Corrects for the finite size of the detector averaging the non-uniform dose distribution.
  - **Fluence Perturbation Correction (kp):** Accounts for the detector perturbing the photon and electron fluence in the measurement field compared to the reference field.
  - **Detector-Specific Correction Factors:** Some detectors may require additional corrections based on their material and design.

**4. Monte Carlo Simulations:**

- Monte Carlo (MC) simulations are increasingly used as a benchmark for validating small field dosimetry measurements and for determining correction factors. MC can accurately model the complex radiation transport in small fields and the interaction with detectors.

**5. Phantoms:**

- Use appropriate phantoms (e.g., water phantoms) with sufficient size to minimize scatter artifacts from the phantom edges.

**Key Recommendations from AAPM TG Reports:**

- **Commissioning:** Thorough commissioning of small field dosimetry equipment and techniques is essential, including careful measurements and validation against independent methods (e.g., MC).
- **Regular QA:** Implement regular quality assurance procedures to ensure the stability and accuracy of small field dosimetry measurements. This includes checks of detector calibration, output constancy, and beam profiles.
- **Documentation:** Maintain detailed records of all small field dosimetry measurements, correction factors applied, and QA procedures.

In summary, measuring dosimetric indices in small fields according to AAPM TG reports requires the use of appropriate small volume detectors, careful measurement techniques, and the application of correction factors to account for the unique challenges associated with non-equilibrium conditions and steep dose gradients. Monte Carlo simulations play an increasingly important role in validating measurements and determining these correction factors. The upcoming TG-155 report is expected to provide even more detailed and consolidated guidance on this critical aspect of stereotactic radiotherapy.

GPT-4o:

Measuring **dosimetric indices in small fields** is one of the most challenging aspects of modern radiotherapy, especially for **SRS/SBRT** and **IMRT**. According to **AAPM TG-155** (Small Field Dosimetry) and supported by **IAEA TRS-483**, there are specific protocols and tools for accurate measurement.

**1. Choose the Right Detector:**

Due to the loss of lateral charged particle equilibrium (LCPE) and steep dose gradients in small fields, choosing suitable detectors is crucial. The AAPM recommends using detectors with high spatial resolution and minimal volume averaging effects. Detectors such as silicon diodes, plastic scintillator, diamond detectors, radiochromic film, and certain types of ionization chambers are preferred for their precision in small field measurements.

**2. Output Factor Measurement:**

Measure output for each small field size.Normalize to a reference field size. Apply output correction factors to account for detector response.

**3. PDD and TMR Measurement:**

Use a scanning system in a water phantom.

Use high-resolution detectors (e.g., scintillator or film).

Scan at small increments (e.g., 1 mm) due to steep dose gradients.

**4. Beam Modeling and Commissioning:**

Use film or diode arrays for lateral beam profiles.

Determine Full Width at Half Maximum (FWHM) and penumbra width.

Important for MLC modeling and treatment planning validation.

**5. Absolute Dose (Point Dose)**

Place detector at **isocenter** in water phantom or solid water.

Use **correction factors** and cross-calibrated electrometers.

Validate with film in high-gradient regions (if needed).

Moonshot-v1:

According to AAPM TG reports and related literature, measuring dosimetric indices in small fields involves several key considerations and recommendations:

**Measurement Techniques and Recommendations**

1. **Detector Selection**:
   - **Small Volume Detectors**: Use detectors with minimal energy, dose, and dose rate dependence. Micro-ionization chambers are recommended for small field dosimetry, though signal-to-noise ratio (SNR) should be carefully evaluated.
   - **Stereotactic Diodes**: For field sizes less than 1×1 cm², stereotactic diodes or electron diodes are recommended. These diodes are also ideal for radiosurgery beams.
   - **Liquid-Filled Ion Chambers, Diamonds, and Diodes**: These detectors are still being characterized and commissioned for reference dosimetry.
2. **Measurement Conditions**:
   - **Source-to-Surface Distance (SSD)**: If the field size is small compared to the detector and electronic equilibrium could be compromised, measurements should be performed at a greater SSD with proper correction.
   - **Multiple Detectors**: Dosimetric measurements should be carried out using more than one detector system to ensure accuracy.
3. **Reference Dosimetry**:
   - **IAEA/AAPM Formalism**: The IAEA/AAPM formalism provides a structured approach for reference dosimetry in small static MV photon fields. This includes defining machine-specific reference fields and clinical fields for accurate dosimetry.
   - **Perturbation Factors**: The lack of perturbation factors for small field ionization chambers increases measurement uncertainty. Efforts are ongoing to derive these factors.
4. **Relative Dosimetry**:
   - **Depth Dose and Output Factors**: Relative dose measurements, including depth dose functions and output factors, should be carefully evaluated. Specialized systems and alternative procedures may be necessary for accurate measurements.
5. **Treatment Planning System Validation**:
   - **Algorithm Suitability**: Treatment planning systems should be validated for small field dosimetry, especially in heterogeneous media. Monte Carlo methods are recommended for modeling dose from small fields in such media.
6. **Error Analysis and Uncertainty**:
   - **Uncertainty Limits**: Provide error analysis and limits of uncertainty in measurements. Spot checks and verifications of smaller fields should be carried out using at least one independent method (e.g., TLD, film, Monte Carlo).

**Practical Considerations**

- **Energy Spectrum Variations**: While the energy spectrum does vary in small fields (e.g., SRS, IMRT), these changes result in insignificant variations in stopping power ratios.
- **Stopping Power Ratios**: For most ion chambers, stopping power ratios in small fields are relatively the same as those in the reference field used in dosimetry codes of practice.
- **Independent Audits**: Small field dosimetry should have an independent audit by a different physicist, either internal or external (e.g., Radiological Physics Center verification).

By following these guidelines and recommendations, medical physicists can improve the accuracy and reliability of dosimetric measurements in small fields, enhancing the precision of treatments like stereotactic radiosurgery and stereotactic body radiotherapy.

Question10 (TG158)

According to AAPM TG reports, what are the main sources of out-of-field dose in radiation therapy? What factors affect the out-of-field dose?

RTPhy-ChatBot:

The main sources of out-of-field dose in radiation therapy include photon leakage through the treatment head of the machine, radiation scattered from collimators and beam modifiers, and radiation scattered within the patient from the treatment beams. For higher-energy (>10 MV) photon beams, there is an additional contribution from neutrons emanating from the treatment head, neutrons produced from photoneutron interactions in the patient, and radioactive isotopes produced in photoneutron interactions.

The relative contributions of collimator scatter, head leakage, and patient scatter to peripheral dose have been investigated. Fraass and van de Geijn found that collimator scatter plus leakage is of the same order of magnitude as patient scatter, and for some machines, the leakage component can vary by a factor of 2 depending on the collimator angle. Kase et al. also indicate that near the beam, collimator scatter contributes 20% to 40% of the total peripheral dose, and leakage becomes the main contributor at greater distances from the field edge.

The out-of-field dose depends on several factors, including distance from the field edge, beam energy, depth in the patient, field size, and accelerator model. The distance from the field edge is typically considered as simply the orthogonal distance from the plane of the field edge, and the dose varies minimally with depth. However, near the surface of the patient, the dose is highly elevated.

The out-of-field dose increases with increasing field size because larger irradiated volumes produce more patient scatter. This field-size dependence is most pronounced close to the field edge, where patient scattered radiation is the most important component of the out-of-field dose. The dose varies little with beam energy and depth in the patient, except near the surface.

The dose estimates outside the treated volume also differ slightly among accelerator models due to variations in head shielding designs. Larger target volumes (such as head and neck) will cause more patient scatter and therefore higher out-of-field doses, particularly near the treatment field. More modulation (i.e., more MU/dose) will cause more head leakage and therefore higher out-of-field doses far from the treatment field. Among other influences, modality affects the degree of modulation. For example, sliding window delivery typically requires 20–30% more MU than step-and-shoot IMRT.

Tomotherapy: The out-of-field doses associated with various forms of tomotherapy, including serial tomotherapy and more modern helical systems, have been evaluated. While the dose varies with the complexity of the target volume and the total number of MUs delivered, in general, the magnitude and distribution of the out-of-field doses from tomotherapy are similar to those of IMRT.

Gemini-2.0-flash:

According to AAPM Task Group (TG) reports, particularly **TG-158: Measurement and Calculation of Doses Outside the Treated Volume from External-Beam Radiation Therapy**, the main sources of out-of-field dose in photon radiation therapy are:

- **Head Leakage:** Photons that penetrate the shielding of the linear accelerator (linac) treatment head. This leakage is present even when the jaws or multileaf collimators (MLCs) are fully closed.
- **Scattered Radiation from Collimation and Beam Modifiers:** Photons that scatter off the primary and secondary collimators (jaws, MLCs), wedges, blocks, and other beam shaping devices in the treatment head. The amount of scatter depends on the field size, the complexity of the collimation, and the presence of beam modifiers.
- **Patient Scatter:** Photons that interact with the patient's body and scatter outside the primary treatment field. The amount and distribution of patient scatter depend on the beam energy, field size, and the patient's anatomy.

**Factors that affect the out-of-field dose:**

Several factors can influence the magnitude and distribution of the out-of-field dose:

- **Treatment Technique:**
  - **IMRT (Intensity-Modulated Radiation Therapy) and VMAT (Volumetric Modulated Arc Therapy):** These techniques often involve more complex beam arrangements, smaller field segments, and longer beam-on times, which can lead to increased head leakage and scatter compared to conventional 3D conformal radiotherapy. However, they typically reduce the in-field dose to surrounding normal tissues.
  - **Conventional 3D Conformal Radiotherapy:** While generally having lower head leakage and scatter compared to IMRT/VMAT for the same target volume, larger margins used in these techniques can result in a larger volume of normal tissue receiving a significant dose.
- **Beam Energy:** Higher energy photon beams generally produce more penetrating leakage radiation and can also influence the amount and direction of patient scatter. They are also associated with the production of photoneutrons, which contribute to the out-of-field dose, especially at energies above 10 MV.
- **Field Size:** Larger treatment fields generally result in increased scatter from the collimators and within the patient, leading to a higher out-of-field dose.
- **Distance from the Field Edge:** The out-of-field dose typically decreases with increasing distance from the edge of the treatment field.
- **Beam Modifiers:** The use of wedges, blocks, and compensators can alter the amount and distribution of scattered radiation.
- **Accelerator Design:** The shielding characteristics and design of the linac treatment head significantly impact the amount of head leakage. Different linac models from various manufacturers can have varying levels of leakage.
- **Monitor Units (MU):** A higher number of MUs delivered during treatment will generally result in a higher out-of-field dose due to increased leakage and scatter.
- **Patient Size and Composition:** The amount of patient scatter depends on the patient's overall size and the density of the tissues being irradiated.
- **Treatment Room Characteristics:** Scatter from the treatment room walls, ceiling, and floor can also contribute to the very low dose levels far from the treatment field.

Understanding these sources and factors is crucial for accurately estimating and minimizing the out-of-field dose to reduce the risk of late effects, such as secondary cancers, in radiotherapy patients. AAPM TG-158 provides detailed guidance on measuring, calculating, and managing these non-target doses.

GPT-4o:

According to **AAPM TG reports**, especially **TG-158** (*Measurement and calculation of doses outside the treated volume*), understanding and managing **out-of-field dose** is crucial for **patient safety**, particularly in pediatric patients and secondary cancer risk modeling.

Out-of-field dose refers to radiation dose received by tissues and organs that are not in or near the target treatment volume — often more than 5 cm outside the field edge.

**Main Sources of Out-of-Field Dose**

1. **Patient scatter:**
   - Scattering of primary beam within the patient's body. Usually the **largest contributor close to the field edge**.
2. **Head leakage radiation:**
   - Radiation that escapes through the linac head shielding (e.g., through MLC, jaws, or gantry head).
3. **Collimator scatter:**
   - Scattering from beam-shaping devices (MLCs, jaws, compensators).
4. **Room scatter:**
   - Radiation scattering off walls, floor, ceiling. Becomes relevant farther away from the field.
5. **Imaging dose (indirect):**
   - Not part of the treatment beam but contributes to peripheral dose (kV/MV imaging).

**Factors Affecting Out-of-Field Dose**

**1. Field Size**

- Larger field → more patient scatter and leakage.

**2. Beam Energy**

- Higher photon energies → increased neutron production (>10 MV) and deeper penetration.
- Neutron dose becomes a concern especially with energies above 15 MV.

**3. Treatment Technique**

- IMRT (Intensity-Modulated Radiation Therapy) or VMAT (Volumetric Modulated Arc Therapy) involve multiple beam angles and longer beam-on times, increasing leakage and scattered radiation.

**4. Distance from Field Edge**

- Dose drops off rapidly with distance.
- Near the field edge: **patient scatter dominates**.
- Far from the field edge: **head leakage becomes dominant**.

**5. Linac Head Design**

- Shielding efficiency, MLC design, and leakage protection vary by vendor/model.

**6. Use of Accessories**

- Bolus, compensators, and shielding can scatter dose in unintended directions.

**7. Patient Anatomy**

- Body size, density, and anatomy can alter scatter patterns.

**Mitigation Strategies**

 **Use lower photon energies** (<10 MV) to avoid neutron dose.

 **Limit field size** to the minimum necessary.

 **Use tighter MLC/jaw collimation**.

 **Avoid unnecessary beam angles** that expose OARs.

 **Limit MU** by optimizing treatment plans.

 **Use proper shielding** in room design and patient-specific setups.

 Consider **out-of-field organs in dose calculation** and risk assessment (especially pediatrics).

Understanding these sources and factors is essential for minimizing unintended exposure and improving patient safety.

Moonshot-v1:

According to AAPM TG reports and related studies, the main sources of out-of-field dose in radiation therapy include:

1. **Leakage from the Linear Accelerator (Linac) Treatment Head**:
   - Leakage radiation from the linac head contributes significantly to out-of-field dose. This leakage can vary depending on the design of the linac, with some models having higher leakage than others.
2. **Scatter from Collimation Devices**:
   - Scatter from collimators, including multileaf collimators (MLCs) and jaws, is another major source of out-of-field dose. The design of the collimation system can influence the amount of scatter radiation.
3. **Scatter from Within the Patient’s Body**:
   - Patient scatter occurs when the primary beam interacts with the patient’s tissues, resulting in secondary radiation that contributes to out-of-field dose. The extent of patient scatter can depend on the size and shape of the treatment field.

**Factors Affecting Out-of-Field Dose**

1. **Linac Design and Type**:
   - Different linac models can produce varying levels of out-of-field dose due to differences in shielding and collimation designs. For example, linacs with larger horizontal waveguides or bending magnets may produce higher out-of-field doses.
2. **Treatment Technique**:
   - Intensity-modulated radiotherapy (IMRT) and stereotactic radiotherapy (SRT) often involve higher out-of-field doses compared to conventional techniques. This is partly due to the increased number of monitor units required for these advanced techniques.
3. **Field Size**:
   - Larger field sizes generally result in higher out-of-field doses due to increased patient scatter. However, the difference in out-of-field dose between different field sizes decreases with increasing distance from the field edge.
4. **Beam Energy**:
   - Lower-energy beams tend to produce higher out-of-field photon doses compared to higher-energy beams. However, high-energy beams may generate neutrons through photonuclear interactions, which can also contribute to out-of-field dose.
5. **Collimator Orientation**:
   - The orientation of the collimator can significantly affect out-of-field dose. Aligning the patient’s craniocaudal axis with the x-plane of the collimator can reduce out-of-field dose by up to an order of magnitude.
6. **Treatment Planning and Delivery**:
   - The specific treatment plan and delivery method can influence out-of-field dose. For example, using conformal beams and avoiding beams directed toward the trunk can reduce dose to organs at risk.

Understanding these sources and factors can help in optimizing treatment planning and delivery to minimize out-of-field dose and associated risks.

Question11 (TG176)

According to AAPM TG reports, in radiotherapy, how much impact does couch and different kinds of patient immobilization devices have on patient dose in radiation therapy?

RTPhy-ChatBot:

Couch tops and various types of patient immobilization devices can significantly affect the delivered dose to patients in radiation therapy. The primary concerns are related to increased skin dose due to the buildup effect of the couch top, as well as attenuation caused by these devices, which can impact the target dose.

The dosimetric effects of couch tops and immobilization devices depend on their composition and thickness. For instance, carbon fiber couch tops and immobilization devices have been shown to increase attenuation and skin dose with magnitudes varying from about 2% for normally incident beams to as much as 17% for denser sections. The combination of the couch top and immobilization device results in even greater attenuation effects.

Skin sparing is diminished when beams transit through the couch, and the additional material of an immobilization device all but eliminates it. Attenuation effects from the combination of couch and various immobilization devices, including body and head frames used for stereotactic radiosurgery, have been reported to reach up to 11%.

For proton radiotherapy, the couch top and immobilization devices act as range shifters. Carbon fiber couches with sandwich designs are commonly used in most proton centers in the U.S., and their wet thickness is taken into account in dose calculations. The change of proton distal range due to the couch top must be considered during the treatment planning process to accurately predict dose in the patient.

It's important for protocol designers to consider how best to collect dose data in regions affected by these devices while taking into account the limitations in dose calculation accuracy. Indexed patient immobilization systems are now commonly used to establish reproducible patient position relative to the couch, providing the best opportunity to accurately account for the couch top and rails during the planning process.

The literature on the dosimetric effect of treatment couches or immobilization devices for heavy charged particles like protons is sparse. However, it's crucial to take these effects into account in the treatment planning process to ensure accurate dose predictions for patients.

To mitigate the impact of couch and immobilization devices on patient dose in radiation therapy, several strategies can be employed:

1. Accurate dose calculation: The protocol designers should consider how best to collect the dose data in regions affected by these devices while taking into account the limitations in the dose calculation accuracy.
2. Use of indexed patient immobilization systems: These systems establish reproducible patient position relative to the couch, providing the best opportunity to accurately account for the couch top and rails during the planning process.
3. Use of compensating filters or bolus material: Compensating filters or bolus material can be used to mitigate the impact of attenuation on target dose. However, this approach requires careful consideration as it may introduce additional uncertainties in the treatment plan.
4. Regular quality assurance and verification: Regularly checking the performance of the couch top and immobilization devices through quality assurance and verification procedures is essential to ensure accurate dose delivery to patients.
5. Continuous monitoring and improvement: Continuously monitoring and improving the design, materials, and manufacturing processes for couch tops and immobilization devices can help minimize their impact on patient dose while maintaining the necessary rigidity, lightweight, and artifact-free imaging capabilities.

Gemini-2.0-flash:

According to AAPM Task Group (TG) reports, particularly **TG-176: Dosimetric effects caused by couch tops and immobilization devices**, the impact of the couch and different kinds of patient immobilization devices on patient dose in radiation therapy is a complex combination of:

- **Increased Skin Dose:** Devices in the beam path, especially those close to the patient's surface, can reduce the skin-sparing effect of megavoltage photon beams. This occurs because electrons generated in the device material can scatter into the skin, increasing the surface dose.
- **Reduced Tumor Dose (Attenuation):** The materials of the couch and immobilization devices attenuate the radiation beam to some extent, meaning they absorb some of the photons before they reach the patient. This can result in a lower dose delivered to the target volume, especially for beams passing through thicker or denser parts of these devices.
- **Altered Dose Distribution:** The presence of these external devices can scatter the radiation beam, which can alter the dose distribution within the patient. This effect depends on the material, thickness, and geometry of the device relative to the beam.

Here's a more detailed look at the impact of each:

**Treatment Couch:**

- Modern couches, especially those made of monolithic carbon fiber, which are optimal for IGRT due to minimal sag and good imaging properties, can cause a more significant increase in skin dose and attenuation compared to older "tennis racket" style couches.
- The magnitude of the effect depends on the beam energy, gantry angle, field size, and the specific couch material and thickness.
- For posterior beams (gantry angle 180°), the skin dose increase is most pronounced as the beam enters the patient through the couch top.
- Attenuation by the couch can lead to a localized dose reduction of several percentage points (4% to 16% reported in some studies, depending on the factors mentioned above) if not accounted for in treatment planning.

**Immobilization Devices:**

A wide variety of immobilization devices are used to improve patient positioning reproducibility, including:

- **Thermoplastic Masks:** Used primarily for head and neck treatments, these masks can cause a measurable increase in skin dose in the areas they cover. The attenuation is generally less significant due to their relatively low density and thickness.
- **Vacuum Cushions (Vac-Lok):** These can conform to the patient's body and provide stable immobilization. The dosimetric impact depends on the thickness and material of the cushion in the beam path.
- **Body Fixation Systems (e.g., All-in-One positioning system - AIO-PS):** These more rigid systems can have denser components that cause both increased skin dose and attenuation.
- **Belly Boards and Breast Boards:** These devices position the patient in a specific way to spare organs at risk. Their impact on dose depends on whether any part of the board is directly in the beam path to the target volume. They can increase skin dose in contact areas and attenuate the beam if dense materials are used.

**AAPM Recommendations and Mitigation Strategies:**

AAPM TG-176 provides several recommendations to address the dosimetric impact of couches and immobilization devices:

- **Include External Devices in Treatment Planning:** Ideally, the couch top and immobilization devices should be included in the patient's CT simulation scan so that their attenuation and scatter effects can be modeled by the treatment planning system (TPS).
- **Accurate Modeling in TPS:** TPS vendors should provide accurate models of commonly used couches and immobilization devices, including their material properties and geometry, to allow for correct dose calculations.
- **Measurements for Validation:** When accurate TPS models are unavailable or when avoidance of high-density structures is not possible, physicists should perform measurements to quantify the attenuation and skin dose effects of these devices for the energies and beam geometries used clinically.
- **Consider Skin Dose:** The increase in skin dose, especially for beams entering through these devices, should be considered, particularly for treatments with high surface doses or large single fractions.
- **Avoid High-Density Structures:** During treatment planning, efforts should be made to avoid having dense parts of immobilization devices directly in the beam path to the target volume whenever clinically feasible.
- **Report Device Properties:** Device vendors should provide information on the attenuation and skin dose characteristics of their products.

In conclusion, AAPM TG reports emphasize that the couch and patient immobilization devices can have a non-negligible impact on the patient dose in radiation therapy, affecting both the skin dose and the dose delivered to the target volume. Accurate modeling in the TPS and/or direct measurements are recommended to account for these effects and ensure accurate dose delivery.

GPT-4o:

According to the **AAPM Task Group reports**, particularly **TG-148** (*Patient Immobilization in Radiation Therapy*), the impact of **couch** and **immobilization devices** on patient dose in radiation therapy depends on various factors such as the materials used, the geometry of the device, and the treatment technique. Let's go through it step by step.

**📌 How Do Couch and Immobilization Devices Impact Patient Dose?**

**1. Couch Impact on Dose**

- The **treatment couch** (often called the "patient support surface") can contribute to **dose attenuation**, particularly with **high-energy beams** (e.g., 15 MV and above).

**Key Factors:**

- **Material of the Couch**
  - **Carbon fiber** couches are commonly used for their low attenuation properties.
  - **Plastic-based or composite materials** may cause more **beam attenuation** and **scatter**.
  - **Metal components** (e.g., locking mechanisms, couch arms) can cause **scattering** and **local dose increases** or **hot spots**.

**Impact:**

- **Up to 5% dose reduction** at the **isocenter** depending on the material and energy.
- The **impact increases at higher energies**, particularly when the beam passes through a thick section of the couch.
- **Higher attenuation in the legs and arms** due to the couch material, especially if the treatment involves a large angle or a wide-field technique.

**2. Immobilization Devices Impact on Dose**

Immobilization devices are crucial for patient positioning and minimizing movement during treatment. However, these devices can also introduce **dose perturbations**. The impact depends on the material, thickness, and how the device is placed relative to the treatment beam.

**Types of Immobilization Devices:**

- **Thermoplastic Masks (for head and neck treatments)**
  - **Minimal impact on dose** if they are thin, but **can cause hot spots** or **dose heterogeneity** due to the material properties.
  - **Thickness** and **positioning** affect dose distribution.
- **Vacuum Cushions and Foam Pads**
  - Generally **low impact** due to the **low-density** nature of the material.
  - Can affect **beam attenuation** if placed close to the treatment area.
- **Belly Boards or Thoracic Cushions (for breast treatments)**
  - Can **reduce dose to the heart** by positioning the patient and **affect dose to surrounding tissue**.
  - **Material and thickness** can slightly alter the **distribution of radiation**.

**Key Impact Factors:**

- **Material Composition**:
  - Materials like **thermoplastics**, **vacuum bags**, and **foam** typically cause **minimal dose perturbation**.
  - Dense materials (e.g., **metal supports**, **contrast agents**) increase **scatter** and **attenuate** the dose more significantly.
- **Thickness of the Device**:
  - Thicker devices attenuate more dose.
  - **Close proximity to the treatment site** (such as immobilization close to the treatment area) can lead to **localized dose increases** or **hot spots**.
- **Positioning of the Device**:
  - Incorrect alignment or incorrect placement of immobilization devices can lead to **misalignment** of the patient, which may result in **target miss** or increased dose to surrounding organs.

**✅ How to Mitigate the Impact of Couch and Immobilization Devices on Dose**

**1. Use Low-Atomic Number Materials**

- **Carbon fiber** for the couch is preferred because it **minimizes attenuation** and **scattering** compared to conventional materials.
- **Vacuum cushions and thermoplastic masks** are designed to be **thin** and **low-density**, minimizing their effect on dose.

**2. Proper Positioning and Alignment**

- **QA checks** of patient setup and alignment should be done regularly to ensure the patient is correctly positioned and immobilized.
- Devices like **vacuum cushions** or **foam pads** should be placed in a way that **does not interfere with critical areas** being treated.

**3. Phantom Studies**

- **Dosimetric measurements** should be made with patient-specific phantoms or on a **regular QA basis** to assess the dose impact of specific couch and immobilization devices.
- These measurements can help **quantify the dose perturbation** and adjust treatment planning or technique accordingly.

**4. Customized Patient-Specific Planning**

- For treatments involving **high-energy beams** (e.g., >10 MV), it's recommended to **consider the dose contribution** from the immobilization device and **couch** and **adjust the treatment planning** to minimize unwanted dose.
- **Advanced planning systems** like **Monte Carlo simulations** or **second-dose calculation algorithms** can model the effects of couch and immobilization devices more accurately.

Moonshot-v1:

According to AAPM TG reports and related studies, the impact of couches and patient immobilization devices on patient dose in radiation therapy can be significant and needs to be carefully considered. Here are the main findings:

**Impact of Couches and Immobilization Devices on Patient Dose**

1. **Couch Tops**:
   - **Skin Dose Increase**: Couch tops, especially those made of carbon fiber, can significantly increase skin dose. For example, studies have shown that surface doses for a 10×10 cm² field can increase by 26%–37.4% for 6 MV and 20%–43.5% for 15 MV photons.
   - **Attenuation of Beam**: Couch tops can also attenuate the photon beam, reducing the dose delivered to the target volume. For example, attenuation through carbon fiber couch tops can range from about 2% for normally incident beams to about 6% for highly oblique beams.
   - **Impact on Dose Distribution**: The presence of couch tops can alter the dose distribution, potentially reducing target coverage. For example, Pulliam et al. showed that treatment couches and rails could reduce target dose coverage by up to 35% in IMRT plans and 18% in RapidArc plans if not accounted for in dose calculations.
2. **Immobilization Devices**:
   - **Skin Dose Increase**: Immobilization devices, such as vacuum bags and head-and-neck immobilization devices, can significantly increase skin dose. For example, vacuum immobilization bags can increase surface dose from 16% to about 68% for an open 6 MV beam.
   - **Attenuation of Beam**: These devices can also attenuate the beam, reducing the dose at depth. For example, Alpha-Cradles and vacuum immobilization bags can cause attenuation of about 1%.
   - **Impact on Dose Distribution**: The presence of immobilization devices can affect dose distribution, potentially reducing target coverage and increasing dose to organs at risk (OARs). For example, neglecting the attenuation effects of immobilization devices in head and neck VMAT plans can cause PTV V95 discrepancies of 0.4–2.3% and D100 deviations of up to 71.4%.

**Factors Affecting the Impact**

- **Beam Energy**: Lower-energy beams tend to be more affected by attenuation and scatter from couch tops and immobilization devices.
- **Device Composition**: The material and thickness of the couch top or immobilization device can significantly affect the magnitude of dose perturbation.
- **Beam Angle**: The angle at which the beam interacts with the device can influence the degree of attenuation and scatter.
- **Patient Positioning**: The presence of air gaps between the device and the patient’s skin can reduce skin dose and affect dose distribution.

**Recommendations**

- **Incorporate Devices in Treatment Planning**: AAPM TG-176 recommends contouring and including immobilization devices and couch tops in treatment planning to account for their dosimetric effects.
- **Regular QA and Calibration**: Regular quality assurance and calibration of treatment planning systems are essential to ensure accurate dose calculations.
- **Use of Advanced Segmentation Techniques**: For 3D-printed immobilization devices, advanced segmentation techniques can help accurately include these devices in dose calculations.

In summary, couch tops and immobilization devices can significantly impact patient dose in radiation therapy, increasing skin dose and reducing dose to the target volume. These effects need to be carefully considered and accounted for in treatment planning to ensure accurate dose delivery and optimal treatment outcomes.

Question12 (TG185)

According to AAPM TG reports, in proton therapy, how to reduce the impact of high-atomic number materials?

RTPhy-ChatBot:

The presence of high-atomic number materials, such as hip prostheses, can significantly affect the dose delivered to the prescription point and to tissue shielded by the prosthesis in radiotherapy. This can result in dramatic differences in treatment outcomes and potential complications. To reduce the impact of high-atomic number materials on radiotherapy, several strategies can be employed:

1. Use lower-energy beams where possible: Lower-energy beams have less penetration power and are therefore less affected by high-atomic number materials. This can help to minimize the dose perturbation caused by these materials.
2. Avoid or minimize the use of materials with high photon and neutron activation cross sections in accelerator components, treatment couches, and accessories: High-atomic number materials used in the construction of these components can lead to increased radiation exposure for both patients and staff due to their radioactive decay products. Minimizing the use of such materials can help to reduce this risk.
3. Restrict the use of high-energy treatment to the latter part of the day: High-energy treatments can result in significant buildup of radioactive isotopes over time. By restricting the use of high-energy treatments to the latter part of the day, the buildup of activity throughout the day can be minimized, reducing the risk of exposure for both patients and staff.
4. Consider delayed entry of staff to the treatment room: Delaying entry of staff into the treatment room for a period after irradiation can help to reduce their exposure to radioactive isotopes. This time interval is most effective for isotopes with half-lives up to several minutes, but longer delays may be required for isotopes with longer half-lives.
5. Use advanced dosimetry techniques: Monte Carlo methods are well suited to accurately predicting the dose near high-atomic number implants and can help to minimize the impact of these materials on treatment outcomes. While correction or model-based TPS will still be used in most clinics, the use of advanced dosimetry techniques such as Monte Carlo simulations can help to improve the accuracy of dose calculations and reduce the impact of high-atomic number materials on radiotherapy. It is important to note that while these strategies can help to minimize the impact of high-atomic number materials on radiotherapy, they cannot completely eliminate the risk. Continued research and development in this area are necessary to fully understand the dosimetric effects of high-atomic number implants and to develop more effective strategies for minimizing their impact on treatment outcomes.

Gemini-2.0-flash:

While AAPM Task Group (TG) reports don't have specific sections solely dedicated to reducing the impact of high-atomic number (high-Z) materials in proton therapy like they do for photon therapy (e.g., TG-63), the general principles and strategies discussed in various reports and the inherent properties of proton beams offer insights into minimizing their impact.

Here's a breakdown of how to reduce the impact of high-Z materials in proton therapy, drawing from general AAPM recommendations and the nature of proton interactions:

**Understanding the Challenges:**

High-Z materials (like metal implants) in the proton beam path can cause several issues:

- **Range Uncertainty:** Protons experience increased and less predictable scattering in high-Z materials compared to tissue. This can lead to uncertainties in the final range of the proton beam, potentially affecting target coverage and increasing dose to distal normal tissues.
- **Dose Perturbations:** High-Z materials can cause local dose enhancements at the entrance surface due to increased energy deposition from secondary electrons generated within the high-Z material. They can also cause dose reductions (shadowing) distally due to attenuation and scattering.
- **Artifacts in Imaging:** Metal artifacts in CT scans used for treatment planning can obscure the anatomy around the implant, leading to inaccuracies in target delineation and dose calculation.

**Strategies to Reduce the Impact:**

1. **Treatment Planning Optimization and Avoidance:**
   - **Beam Angle Selection:** Carefully choose beam angles to minimize the path length of the proton beam through high-Z materials. If possible, select beam angles that avoid direct irradiation of the implant or critical structures distal to it.
   - **Range Robustness:** Utilize robust treatment planning techniques that account for potential range uncertainties caused by the presence of high-Z materials. This might involve creating multiple scenarios with varying densities of the high-Z material or using optimization algorithms that are less sensitive to these uncertainties.
   - **Distal Margin Expansion:** Increase distal margins beyond the implant to account for potential range overshoots due to scattering in the high-Z material. However, this needs to be balanced with sparing distal critical structures.
   - **Dose Painting:** In advanced proton therapy techniques like intensity-modulated proton therapy (IMPT), consider dose painting strategies to potentially compensate for dose perturbations caused by the implant, although this is complex and requires accurate modeling.
2. **Imaging Artifact Reduction:**
   - **Metal Artifact Reduction (MAR) Algorithms:** Utilize MAR algorithms during CT image reconstruction to reduce the artifacts caused by high-Z materials. This can improve the accuracy of target delineation and density information used for treatment planning.
   - **Dual-Energy CT:** In some cases, dual-energy CT might provide better material differentiation and help reduce metal artifacts.
   - **Alternative Imaging Modalities:** Consider using alternative imaging modalities like MRI or PET/CT, which are less susceptible to metal artifacts, for target delineation and registration when appropriate.
3. **Accurate Material Characterization and Treatment Planning System Modeling:**
   - **Density Override:** If the density of the high-Z material is known, override the CT-based density in the treatment planning system to a more accurate value. This can improve range and dose calculation accuracy.
   - **Advanced TPS Modeling:** Advocate for and utilize treatment planning systems that have advanced algorithms capable of more accurately modeling proton interactions in heterogeneous media, including high-Z materials. This is an ongoing area of development.
4. **Experimental Verification:**
   - **Phantom Studies:** For complex cases with large high-Z implants, consider performing patient-specific phantom studies to experimentally verify the dose distribution in the presence of the implant. This can help validate the treatment plan and identify potential issues.
5. **Clinical Experience and Guidelines:**
   - Follow any emerging clinical guidelines and best practices shared within the proton therapy community regarding the treatment of patients with high-Z materials.

**Important Considerations:**

- **Material Composition:** The specific type of high-Z material (e.g., titanium vs. stainless steel) will affect the amount of scattering and attenuation.
- **Size and Location:** The size and location of the high-Z implant relative to the target volume and beam path are critical factors in determining the magnitude of the impact.

While there isn't a single AAPM TG report dedicated solely to this issue in proton therapy, the principles of accurate treatment planning, imaging artifact reduction, and understanding particle interactions, as discussed across various TG reports related to proton therapy (e.g., TG-224 on QA, TG-185 on commissioning of IMPT), are crucial in mitigating the impact of high-Z materials. The field is continuously evolving, and future TG reports may provide more specific guidance as clinical experience and research in this area grow.

GPT-4o:

In proton therapy, the presence of high-atomic-number (high-Z) materials, such as metallic implants, can significantly impact the proton beam due to increased scattering, attenuation, and range uncertainty. The American Association of Physicists in Medicine (AAPM) provides guidelines and recommendations to mitigate these effects. Strategies include:

**1. Avoid Beam Entrance Through High-Z Regions**

- **First line of defense**: Design beam angles that **bypass high-Z implants**.
- Use **beam eye view (BEV)** and digitally reconstructed radiographs (DRRs) to assess.
- If beams must pass near metals, **minimize traversal length** through the material.

**2. Use Robust Treatment Planning**

- Incorporate **robust optimization** to account for uncertainties caused by high-Z regions.
- Account for range and setup uncertainties in optimization (±3.5% range shift, ±3–5 mm setup shifts).
- TG-185 strongly recommends **robust planning techniques** when metallic implants are near or within the beam path.

**3. Override CT Numbers with Known Material Properties**

- **CT artifacts can lead to inaccurate HU → SPR conversion**.
- Solution: **Manually override** the CT numbers or SPR values for the high-Z object based on known composition.
- Use **treatment planning system (TPS) overrides**:
  - For example, titanium implant → assign correct **SPR ~3.2–3.4**
- Refer to **ICRU-49 or NIST tables** for accurate stopping powers.

**4. Use Dual-Energy CT (DECT) or Monte Carlo-Based Methods**

- DECT can improve **SPR estimation** over single-energy CT, especially for high-Z materials.
- Use Monte Carlo dose calculation engines for **better modeling of scattering and energy loss**.
- TG-185 and TG-256 recommend **Monte Carlo** when high precision is needed near high-Z materials.

**5. Add Margins or Use Range Compensators (If Available)**

- In older passive scattering systems, a **range compensator** can be customized to account for inhomogeneities.
- In pencil beam scanning (PBS), add **range margins** during planning to account for high-Z uncertainty.

**6. Consider Secondary Imaging (MRI, Orthogonal X-rays)**

- To reduce **artifact impact**, use other imaging modalities to visualize tumor location and implant placement.
- MRI (if safe) can help delineate soft tissue structures that are obscured on CT.

**7. Patient-Specific QA and Phantom Verification**

- If a beam path involves or is close to a high-Z material, do **end-to-end QA testing** using anthropomorphic phantoms with embedded metal.
- Validate with **film dosimetry**, **ion chambers**, or **3D dosimeters**.

Moonshot-v1:

According to AAPM TG reports and related studies, reducing the impact of high-atomic number materials in proton therapy involves several strategies:

**Sources of Impact**

1. **Range Shift and Dose Perturbation**:
   - High-atomic number materials (e.g., lead, beryllium) can significantly alter the range and dose distribution of proton beams. This is due to the increased stopping power and nuclear interactions of these materials.
   - For example, the presence of high-Z materials can cause range shifts and increased dose perturbations, especially at higher proton energies.

**Strategies to Mitigate the Impact**

1. **Accurate Range Estimation**:
   - Use water-equivalent thickness (WET) measurements to account for the range shift caused by high-Z materials.
   - Employ dual-energy CT (DECT) to better estimate tissue properties and reduce proton range uncertainty.
2. **Optimized Treatment Planning**:
   - Incorporate high-Z materials into treatment planning models to account for their dosimetric effects.
   - Use Monte Carlo simulations to accurately model the interaction of protons with high-Z materials and optimize dose delivery.
3. **Material Selection and Design**:
   - Choose materials with lower atomic numbers for immobilization devices and other components to minimize dose perturbations.
   - Design treatment setups to minimize the interaction of proton beams with high-Z materials.
4. **Regular Quality Assurance (QA)**:
   - Perform regular QA checks to ensure the accuracy of proton beam delivery in the presence of high-Z materials.
   - Validate treatment plans and delivery systems using phantoms and advanced imaging techniques.

By implementing these strategies, the impact of high-atomic number materials on proton therapy can be significantly reduced, ensuring more accurate dose delivery and improved treatment outcomes.

Question13 (TG203)

According to AAPM TG reports, during radiotherapy, what is the maximum dose allowed for a pacemaker? During the treatment process, what should be paid attention to?

RTPhy-ChatBot:

According to the report of AAPM Task Group No. 34, contemporary cardiac pacemakers can fail from radiation damage at doses as low as 10 gray and exhibit functional changes at doses as low as 2 gray. Therefore, it is recommended that radiation therapy patients with implanted pacemakers be planned so as to limit accumulated dose to the pacemaker to 2 gray.

During radiotherapy, the following points should be paid attention to:

1. The radiation oncologist should consult with and dose to the part of the CIED (Cardiac Implantable Electronic Device) body closest to the radiation and inform the treating cardiologist about the indications for field. Absorbed dose to the pacemaker should be evaluated by an MP (Medical Physicist) and documented.
2. The patient should be informed about the small risks involved and requested to move the CIED out of the direct radiation field if possible. If placement within the field is unavoidable, the physicist, radiation oncologist, and cardiology department should engage in a consultation. The patient should be associated with the High-Risk category.
3. The quality of the treatment plan should not generally be compromised for the sake of sparing the device because controlling the disease will be more important than reducing the comparatively low risk of device failure.
4. If double-exposure portal imaging in radiation therapy exposes the pacemaker to the direct beam, which could possibly cause momentary transient interference, further study is needed to investigate this effect, particularly since it may be dose rate and/or pulse frequency dependent.
5. The use of other radiation therapy machines should be evaluated on an individual basis and approached with caution.
6. It is unreasonable to propose that all pacemaker manufacturers radiation harden their devices due to the significant expense that would eventually have to be passed on to all cardiac pacemaker patients.

Gemini-2.0-flash:

According to AAPM Task Group (TG) reports, particularly **TG-34 (published in 1994) and the more recent TG-203 (published in 2019)**, there isn't a single absolute "maximum dose allowed" for a pacemaker during radiotherapy due to variations in device sensitivity and the complexity of radiation interactions with electronic components. However, these reports provide guidance and recommendations based on available data:

**Maximum Dose Considerations:**

- **TG-34 (1994):** This report suggested limiting the accumulated dose to the pacemaker to **2 Gray (Gy)** if possible, based on in vitro experiments and limited clinical data at the time. Functional changes in pacemakers were observed at doses as low as 2 Gy, and failures at around 10 Gy.
- **TG-203 (2019):** This more recent report provides a risk-based approach. It categorizes patients based on:
  - **Pacing dependency:** Whether the patient's heart function relies on the pacemaker.
  - **Cumulative dose to the device.**
  - **Presence of neutrons:** Higher energy photon beams (>10 MV) and proton therapy can produce neutrons, which have a higher risk of causing device malfunction.

TG-203 suggests the following risk categories based on cumulative dose with photon energies ≤ 10 MV (non-neutron producing):

- - **Low Risk:** Pacing-independent and dose to CIED < 2 Gy.
  - **Medium Risk:** Pacing-independent and dose to CIED 2-5 Gy OR Pacing-dependent and dose to CIED < 5 Gy.
  - **High Risk:** Dose to CIED ≥ 5 Gy for both pacing-independent and pacing-dependent patients.

For neutron-producing beams (photons > 10 MV or protons), all cases are considered high risk.

**Key Takeaway on Maximum Dose:** While earlier recommendations suggested a 2 Gy limit, TG-203 acknowledges that higher doses might be acceptable in certain low-risk scenarios (pacing-independent, <10 MV photons). However, the overarching principle is to **minimize the dose to the pacemaker as much as reasonably achievable (ALARA)**.

**During the Treatment Process, What Should Be Paid Attention To?**

According to AAPM TG reports, several factors should be carefully considered and managed during the radiotherapy process for patients with pacemakers:

1. **Pre-Treatment Assessment and Planning:**
   - **Identify the presence and type of pacemaker:** Obtain detailed information about the device (manufacturer, model, date of implant, pacing dependency) from the patient and their cardiologist.
   - **Evaluate pacemaker function:** A baseline device check by a cardiologist or electrophysiologist is recommended before starting radiotherapy, especially for high-risk patients or if the device hasn't been checked recently.
   - **Estimate the dose to the pacemaker:** The treatment planning system (TPS) should be used to calculate the expected dose to the device based on its location relative to the treatment fields. If the device is near or within the treatment field, accurate dose calculation is crucial. For devices outside the primary field by more than a few centimeters, the TPS might underestimate the dose from scatter and leakage, and in-vivo dosimetry might be considered.
   - **Minimize dose to the pacemaker:** Treatment planning should aim to keep the pacemaker out of the direct beam whenever possible. Techniques like beam shaping, blocking, and careful field arrangement should be employed to reduce the dose to the device. Using photon energies ≤ 10 MV is recommended to avoid neutron production.
   - **Consider device location:** If the pacemaker is located in a region that is unavoidable in the treatment field, discuss the risks and benefits with the cardiologist.
2. **During Treatment Delivery:**
   - **Avoid direct irradiation:** Ensure that the pacemaker is not directly in the path of the primary radiation beam.
   - **Monitor the patient:** Observe the patient during each treatment for any signs or symptoms of pacemaker malfunction (e.g., dizziness, palpitations, syncope). The need for continuous ECG monitoring should be determined based on the patient's risk category and clinical status.
   - **Minimize imaging dose:** When using image guidance (IGRT), such as CBCT, ensure the pacemaker is outside the imaging field of view as much as possible to reduce the cumulative dose to the device from imaging.
   - **Be aware of potential electromagnetic interference (EMI):** While modern radiotherapy equipment is generally designed to minimize EMI, be aware of the potential for interactions, especially with older devices or specific equipment malfunctions.
3. **Post-Treatment Follow-up:**
   - **Follow-up device check:** A post-treatment device check by a cardiologist or electrophysiologist is recommended, especially if the estimated or measured dose to the pacemaker exceeded the low-risk threshold or if the patient experienced any symptoms during treatment.
   - **Long-term monitoring:** Patients should be educated about the potential for delayed device malfunction and advised to report any unusual symptoms to their cardiologist.

In summary, the management of radiotherapy patients with pacemakers requires a collaborative approach involving the radiation oncologist, medical physicist, and cardiologist. The goal is to deliver the necessary cancer treatment while minimizing the risk of pacemaker malfunction by carefully planning the treatment to avoid direct irradiation and limit the dose to the device, monitoring the patient during treatment, and ensuring appropriate follow-up. The recommendations in AAPM TG-203 provide a comprehensive framework for this process.

GPT-4o:

Based on guidance from the AAPM Task Group 203 (TG-203): “Management of Radiotherapy Patients with Implanted Cardiac Pacemakers and Defibrillators.”. AAPM **does not set an absolute “safe” dose limit**, but provides **risk-based stratification** to guide clinical decision-making. Here’s how it works:

Low Risk: <2Gy

Moderate Risk: 2-10Gy

High Risk: >10Gy

**Considerations During the Treatment Process**

**1. Determine Device Type**

- **Pacemaker** vs. **ICD**
- ICDs are **more sensitive** to radiation.
- Know the **manufacturer and model** — they have different tolerances and behaviors.

**2. Estimate and Measure Dose to the Device**

- During treatment planning:
  - **Contouring the device** is essential.
  - Use **dose calculation tools** or perform **in vivo dosimetry** with TLDs or OSLDs.
- Include **scattered dose** in estimations — the TPS may **underestimate dose** to out-of-field regions.

**3. Avoid Direct Beam Path**

- Place beams to **avoid irradiating the device directly**.
- Use **beam angles**, **blocking**, or **replanning** to minimize exposure.

**4. Daily Monitoring (for moderate or high-risk cases)**

- Interrogate the device **before and after treatment** sessions.
- **Continuous ECG monitoring** may be needed during treatment, especially for dependent patients.
- Emergency equipment and personnel should be available.

**5. Minimize Use of Neutron-Producing Beams**

- Avoid energies **>10 MV photons or >20 MeV electrons**, which produce **neutrons** that can disrupt electronics.
- If high-energy beams are required, **plan accordingly and increase monitoring**.

**6. Communicate with Cardiology**

- Collaborate with the patient's **cardiologist or electrophysiologist** to assess dependence on the device and plan for contingencies.

Moonshot-v1:

According to AAPM TG-34 and other relevant guidelines, the maximum dose allowed for a cardiac pacemaker during radiotherapy should be limited to **2 Gy**. This recommendation is based on studies showing that pacemakers can exhibit functional changes at doses as low as 2 Gy and can fail at doses as low as 10 Gy.

**Precautions During Treatment**

1. **Avoid Direct Beam Exposure**:
   - Pacemakers should not be placed in the direct (unshielded) therapy beam, as some accelerator beams can cause transient malfunction.
2. **Estimate and Monitor Dose**:
   - The absorbed dose to the pacemaker should be estimated before treatment. If the total estimated dose to the pacemaker might exceed 2 Gy, the pacemaker function should be checked prior to therapy and possibly at the start of each following week of therapy.
3. **Programming Considerations**:
   - If the average dose rate at the device is >1 cGy/min, the pacemaker should be programmed into asynchronous pacing mode.
4. **Beam Energy**:
   - The beam energy should be <10 MV to minimize the risk of neutron contamination, which can damage the pacemaker.
5. **Regular Monitoring**:
   - Close monitoring of the pacemaker function during the first treatment and subsequent treatments is recommended. Continuous electrocardiogram (ECG) monitoring during treatment may be considered in patients with implantable cardioverter-defibrillators (ICDs) who have had prior treatments for ventricular arrhythmias.
6. **Device Relocation**:
   - In some cases, it may be necessary to relocate the pacemaker further away from the radiation field to reduce dose exposure.
7. **Multidisciplinary Collaboration**:
   - Collaboration among cardiologists, radiation oncologists, and medical physicists is essential to minimize the risk to patients.

By following these guidelines and precautions, the risk of pacemaker malfunction during radiotherapy can be significantly reduced, ensuring patient safety and effective treatment outcomes.

Question14 (TG264)

According to AAPM TG reports, what are the limitations of MLC tracking?

RTPhy-ChatBot:

MLC tracking is a technique used to improve the agreement between the planned and delivered dose distributions to the treatment target(s) and OARs by continuously adjusting the multileaf collimator (MLC) positions based on real-time target position information. However, there are several limitations to MLC tracking that need to be considered:

1. Compatibility with other motion management strategies: While MLC tracking can be used as the sole motion management strategy, it is often used in conjunction with other techniques such as gating, compression or breath hold devices, and couch tracking. The goal of MLC tracking is to improve the agreement between the planned and delivered dose distributions, but it cannot control all aspects of motion management.
2. Target position monitoring system requirements: MLC tracking relies on a real-time target position monitoring system to provide accurate information about the target position. The uncertainty in the surrogate target relationship must be known and typically should not exceed 2 mm. Additionally, the position monitoring signal must be an established surrogate for the target motion.
3. Leaf width limitation: MLC tracking can only adjust the positions of the leaves within the available leaf widths. This limitation may impact the ability to deliver accurate doses to small targets or complex treatment plans.
4. Fallback strategy in case of MLC tracking failure: In the event of MLC tracking failure, there must be a fallback strategy in place to ensure safe and effective delivery of radiation therapy.
5. Trade-offs between target and critical structures: When treating targets that are close to or overlap critical structures, there may be dosimetric consequences. Mitigation strategies such as PRV concept, gating thresholds, and trade-offs between target and critical structure may need to be considered.
6. Complexity of treatment plans: MLC tracking requires a significant amount of computational resources to continuously adjust the leaf positions based on real-time target position information. Constraints on plan complexity may need to be considered to ensure safe and effective implementation of MLC tracking.
7. Clinical decisions and considerations: Safe implementation of MLC tracking requires careful consideration of clinical decisions such as pretreatment imaging, unanticipated events, and patient-specific factors. Compatibility with other motion management strategies, QA programs, and regulatory agencies also need to be considered.
8. Uncertainties and limitations of prediction: Prediction is often used for respiratory motion targets to account for system latencies. However, uncertainties and limitations of prediction may impact the accuracy of MLC tracking.
9. Safe implementation considerations: The requirements and decisions for the clinical implementation of MLC tracking include compatibility with other motion management strategies, minimum requirements for a real-time target position monitoring system, and safe implementation considerations such as QA programs and regulatory agencies. Overall, while MLC tracking offers significant benefits in improving the agreement between the planned and delivered dose distributions, it is important to carefully consider the limitations and challenges associated with this technique to ensure safe and effective implementation in radiotherapy.

Gemini-2.0-flash:

According to AAPM Task Group (TG) reports, particularly **TG-264: The Safe Clinical Implementation of MLC Tracking in Radiotherapy**, MLC tracking, while offering significant potential for improving radiotherapy by adapting the treatment beam to target motion in real-time, has several limitations:

**1. Technical Limitations:**

- **Latency:** There is an inherent delay between the detection of target motion and the corresponding movement of the MLC leaves. This latency can lead to a geometric mismatch between the beam aperture and the moving target, especially for fast or irregular motion.
- **Accuracy of Tracking System:** The overall accuracy of MLC tracking is dependent on the accuracy of the motion detection system (e.g., optical tracking, electromagnetic tracking, image-based tracking). Errors in target localization directly translate to errors in MLC positioning.
- **MLC Mechanical Limitations:** MLC leaves have a finite speed and acceleration. For rapid target motion, the MLCs may not be able to keep up with the target's velocity and trajectory, leading to underdosing or overdosing.
- **Geometric Uncertainty:** Even with accurate tracking, there's a residual geometric uncertainty due to the interplay between the moving target and the dynamic MLC aperture. This uncertainty needs to be carefully evaluated and potentially accounted for in treatment margins.
- **System Integration Complexity:** Implementing MLC tracking requires tight integration between the motion tracking system and the linac control system. This complexity can introduce potential points of failure and requires rigorous commissioning and quality assurance.

**2. Clinical Implementation Challenges:**

- **Target Visibility:** Image-based tracking methods rely on sufficient contrast between the tumor and surrounding tissues. Reduced tumor visibility due to overlaying structures or poor contrast can lead to target localization failure and inaccurate MLC tracking.
- **Multi-Target Tracking:** Tracking multiple targets with independent motion simultaneously adds significant complexity to the system and can introduce further limitations in accuracy and deliverability.
- **Deformable Motion:** Most current MLC tracking systems primarily account for rigid body motion (translation and rotation). Managing complex deformable motion of the target remains a significant challenge.
- **Interplay Effects:** For scanned beam modalities like VMAT, the interplay between the moving MLCs and the moving target can create complex dose distributions that may deviate from the planned dose.
- **No Independent Verification System:** As highlighted in TG-264, there is often a lack of independent verification systems for MLC tracking that are separate from the accelerator hardware and tracking software. This makes it challenging to detect latent errors in the feedback loops.
- **Increased Failure Modes:** MLC tracking introduces additional potential failure modes into the treatment delivery workflow, requiring thorough risk assessment and mitigation strategies.

**3. Dosimetric Considerations:**

- **Dose Blurring:** Even with perfect tracking, the continuous motion of the target and the adapting MLCs can lead to some degree of dose blurring compared to a static delivery.
- **Impact on OAR Sparing:** While MLC tracking aims to improve target coverage and spare organs at risk (OARs), inaccuracies in tracking can potentially lead to unintended dose to nearby critical structures. The benefits of tracking need to be carefully weighed against these potential risks.

**In summary, while MLC tracking holds great promise for improving the precision of radiotherapy for moving targets, its limitations in terms of latency, accuracy of the tracking system, mechanical constraints of the MLCs, target visibility, deformable motion, and the complexity of system integration must be carefully considered during clinical implementation and quality assurance.** AAPM TG-264 provides comprehensive guidance on the safe and effective clinical use of this technology, emphasizing the need for thorough commissioning, rigorous QA, and a clear understanding of its limitations.

GPT-4o:

**MLC tracking** is a dynamic technique where the MLC leaves move in real time to compensate for **intrafraction motion**, such as **tumor motion due to respiration**. The idea is to keep the radiation beam aligned with the **moving target**.

**⚠️ AAPM-Reported Limitations of MLC Tracking**

**1. Latency (System Delay)**

- **Problem**: There's a **time delay** between detecting target motion and executing leaf movement.
- Even small delays (e.g., 100–300 ms) can cause **geometric errors**.
- **AAPM TG-142** emphasizes that latency must be measured and minimized.
- **Impact**: Reduced tracking accuracy, potential target miss.

**2. Accuracy Limitations**

- MLCs can only follow motion in the **MLC plane direction (usually X or Y)** — not in all directions (like Z or diagonal vectors).
- **Complex 3D motion** (e.g., tumors moving diagonally or with rotation) cannot be fully compensated.

**3. Mechanical Constraints**

- **MLC leaves have physical limits**:
  - Maximum speed
  - Maximum acceleration
  - Minimum leaf gap
- Rapid or irregular tumor motion can exceed the **mechanical capabilities** of the MLC system.

**4. Dosimetric Uncertainty**

- Rapid, real-time motion compensation can cause:
  - **Underdosing** if leaves lag behind
  - **Overdosing** if motion prediction is incorrect
- **Dosimetric modeling** in the TPS often does **not fully account for dynamic tracking motion**, leading to inaccuracies in **dose calculation**.

**5. Limited Integration with Imaging and Motion Detection**

- MLC tracking requires:
  - **Real-time motion monitoring** (via surface guidance, fiducial tracking, or internal imaging)
  - **Fast feedback loops**
- Integration between motion detection systems and treatment machines can be **limited** or **vendor-dependent**.
- **AAPM TG-76** stresses the importance of reliable motion tracking systems — errors here propagate to MLC tracking.

**6. Increased QA Complexity**

- Daily QA must verify that:
  - MLCs respond correctly to motion input
  - Latency and accuracy are within limits
- QA for dynamic systems is **more complex and time-consuming** than for static or gated treatments.
- **AAPM TG-142** and **TG-147** provide guidance on dynamic MLC QA.

**7. Limited Clinical Availability**

- Only certain **linear accelerators** and software platforms currently support **real-time MLC tracking**.
- Integration is still **under development** in many commercial systems.

**8. Motion Prediction Inaccuracy**

- Some systems use **motion prediction algorithms** to compensate for latency.
- These can fail when **motion becomes irregular**, e.g., due to coughing, shallow breathing, or patient movement.

Moonshot-v1:

According to AAPM TG reports and related studies, the limitations of multi-leaf collimator (MLC) tracking in radiotherapy include the following:

**1. Dosimetric Errors for Organs-at-Risk (OARs)**

- **Single-Point Tracking Limitation**: MLC aperture responds in real-time to the positional change of a single point (typically, an external/internal surrogate for the tumor centroid) and does not account for OARs that may come in the beamline during respiration. This can lead to significant dosimetric errors, especially for centrally-located tumors.

**2. Plan Complexity and Target Motion**

- **Plan Complexity**: The complexity of the treatment plan, including leaf thickness, target shape, and aligning the collimator angle with the major axis of motion, can affect the accuracy of MLC tracking.
- **Target Motion Complexity**: The complexity of the target motion can also impact the effectiveness of MLC tracking. For example, large target motion perpendicular to the MLC leaves can create dose artifacts with regions of overdose in the clinical target volume (CTV).

**3. Latency and Real-Time Imaging Requirements**

- **Latency**: Real-time tracking should have a total latency of less than 500 ms to ensure effective motion management.
- **Real-Time Imaging**: The surrogate for the target must be established with known uncertainty (typically ≤ 2 mm), and the position monitoring signal must be accurate and precise (typically ≤ 1 mm). The frequency of the position monitoring signal measurement should meet the definition of real-time (typically ≥ 3 Hz for targets affected by respiratory motion).

**4. Margin Considerations**

- **Margin Uncertainties**: MLC tracking does not necessarily mean smaller margins. Some patients may not benefit from tracking due to uncertainties in motion surrogate accuracy, deformation (and rotation) of targets with respiration, MLC tracking errors, and target delineation error.

**5. Quality Assurance (QA)**

- **Pre-Treatment QA**: Pre-treatment QA should include patient-specific, system QA, and post-treatment dose reconstruction to ensure spatial and dosimetric accuracy, efficient dose delivery, and detection of and response to anomalous conditions.

**6. Multi-Target Tracking**

- **Sub-Optimal Delivered Dose**: Current MLC tracking approaches use 2D geometric optimization where the MLC aperture shape is simply translated to correspond to the target's motion, resulting in sub-optimal delivered dose. A dose-optimized multi-target MLC tracking method is needed to minimize 3D dosimetric error.

**7. Systematic and Random Errors**

- **Systematic Errors**: Systematic errors in MLC tracking can lead to dose delivery inaccuracies. For example, the latencies and geometric errors produced by the system can impact the effectiveness of MLC tracking.
- **Random Errors**: Random errors in the position monitoring signal and MLC leaf movement can also affect the accuracy of dose delivery.

These limitations highlight the need for continued research and development to improve the accuracy and effectiveness of MLC tracking in radiotherapy.

Question15 (TG302)

According to AAPM TG reports, for deep-inspiration breath hold (DIBH) for breast radiotherapy treatment with bolus, what should we pay attention to when performing surface guided radiotherapy?

RTPhy-ChatBot:

When performing surface guided radiotherapy for DIBH for breast radiotherapy treatment with bolus, there are several important considerations.

Firstly, it is essential to acquire a new reference surface at each treatment session with the bolus in place and confirm its accuracy. Conventional bolus with a reflective surface may not be accurate for DIBH guided by SGRT due to minimal movement occurring at the apex of the lung during deep inspiration.

Secondly, it is crucial to verify heart position for left-sided cancers using another IGRT modality (MV, fluoroscopy, CBCT, MRI, etc.) at first treatment and thereafter at least once per week. This step ensures accurate target localization and minimizes the risk of inaccurate dose delivery to critical structures such as the heart.

Thirdly, fine-tune positioning using SGRT at DIBH is necessary after initial positioning at FB. X-ray based target localization (e.g., kV/MV ports) can be used to confirm DIBH position and for troubleshooting as needed.

Fourthly, automatic beam hold should be enabled when possible during bolus fields to ensure accurate delivery of radiation doses.

Lastly, selected tolerances should depend on surface type, immobilization, and setup workflow but should be reviewed and tightened as the institution gains experience. For a DICOM reference surface, most clinics should be able to satisfy 2-3° rotational and 3-5 mm translational thresholds in each dimension.

Overall, careful attention to these factors is necessary when performing surface guided radiotherapy for DIBH for breast radiotherapy treatment with bolus to ensure accurate target localization, minimize the risk of inaccurate dose delivery, and optimize patient outcomes.

Gemini-2.0-flash:

According to AAPM Task Group (TG) reports, particularly those concerning surface-guided radiotherapy (SGRT) like **TG-302: Surface Guidance for Radiation Therapy** and those discussing breast radiotherapy and respiratory motion management (like **TG-188** and potentially updates to **TG-76**), when performing SGRT for deep-inspiration breath-hold (DIBH) breast radiotherapy treatment *with bolus*, several key aspects require careful attention:

**1. Baseline Surface Setup and Reproducibility:**

- **Accurate Baseline Acquisition:** A stable and reproducible baseline surface image of the patient in the DIBH position *with the bolus in place* is crucial. The bolus can significantly alter the surface topography, and the baseline must reflect the treatment setup.
- **Bolus Placement Consistency:** Ensure the bolus is positioned consistently for every fraction, as variations in its placement will affect the surface geometry and potentially the SGRT tracking accuracy. Document the bolus placement meticulously.
- **Patient Positioning with Bolus:** The patient's overall position on the treatment couch must be highly reproducible with the bolus in place. Any shifts or rotations relative to the baseline can lead to inaccurate beam delivery.

**2. Surface Tracking Accuracy with Bolus:**

- **Bolus Stability:** The bolus itself must remain stable relative to the patient's skin surface during the breath-hold. Movement or shifting of the bolus can introduce errors in surface tracking. Consider using securement methods for the bolus if necessary.
- **Surface Changes Due to Bolus:** The bolus can compress or deform the underlying breast tissue, leading to surface changes compared to a no-bolus DIBH. The SGRT system must accurately track the relevant surface features *with the bolus present*.
- **Potential for Occlusion:** The bolus might obscure some of the tracked surface points, potentially reducing the accuracy or robustness of the SGRT system. Ensure sufficient trackable surface area remains visible.
- **Impact on Tracking Algorithms:** The presence of the bolus might affect the performance of the SGRT tracking algorithms. Validate the system's accuracy with the bolus in place during commissioning and QA.

**3. Breath-Hold Monitoring and Gating:**

- **Correlation with Internal Anatomy:** While SGRT tracks the external surface, it's crucial to ensure a strong and consistent correlation between the external surface changes and the internal target (breast tissue and lymph nodes) displacement during DIBH *with the bolus*. The bolus might influence this correlation.
- **Breath-Hold Stability:** Monitor the duration and stability of the patient's breath-holds *with the added weight and potential discomfort of the bolus*. Inconsistent breath-holds can lead to treatment interruptions and reduced accuracy.
- **Gating Thresholds:** Set appropriate gating thresholds based on the surface motion observed during DIBH *with the bolus*. These thresholds might differ from those used without a bolus.

**4. Dosimetric Considerations Related to SGRT and Bolus:**

- **Bolus Material and Thickness:** The material and thickness of the bolus will affect the surface dose and the dose distribution within the breast tissue. This is a primary reason for using a bolus and must be accounted for in the treatment plan. SGRT ensures the beam is delivered to the correct skin surface where the bolus is placed.
- **Air Gaps:** Ensure minimal air gaps between the bolus and the skin surface, as air gaps can alter the intended skin dose enhancement. SGRT helps maintain the planned patient setup where the bolus conforms to the surface.
- **Beam Alignment Relative to Bolus:** SGRT ensures the radiation beam is accurately aligned with the patient's surface where the bolus is positioned, maximizing the intended dosimetric effect of the bolus on the superficial tissues.

**5. Quality Assurance (QA):**

- **End-to-End Testing with Bolus:** Perform end-to-end tests that include the bolus to verify the accuracy and reproducibility of the entire treatment process, from simulation to delivery, under SGRT guidance.
- **Regular QA Checks:** Include checks with the bolus in place during routine SGRT QA procedures to ensure the system's continued accuracy under these specific conditions.

**In summary, when using SGRT for DIBH breast radiotherapy with a bolus, the key attention points revolve around ensuring the SGRT system accurately captures and tracks the patient's surface *with the bolus in place*, maintaining a consistent correlation between external surface motion and internal target position during DIBH with the bolus, and verifying the overall accuracy and reproducibility of the treatment delivery with the bolus integrated into the setup.** The presence of the bolus introduces additional complexities that require careful consideration throughout the entire treatment workflow. Consulting relevant AAPM TG reports on SGRT and breast radiotherapy will provide further detailed guidance.

GPT-4o:

**Key Considerations for SGRT in DIBH with Bolus**

**1. ✅ Bolus Placement Must Be Reproducible**

- **Challenge**: Bolus adds to surface irregularity. Poor placement can:
  - Alter beam attenuation
  - Cause misregistration in SGRT systems
  - Create dosimetric uncertainty
- **Recommendation**:
  - Use **consistent placement markers** (tattoos, skin marks, or immobilization molds)
  - Align bolus **before SGRT activation**
  - Consider **custom-molded or 3D-printed bolus** for reproducibility

**2. ✅ Register to a Consistent Surface ROI**

- **AAPM TG-302** recommends defining a **region of interest (ROI)** for SGRT that:
  - Excludes **high-motion regions** (e.g., inferior breast tissue or soft bolus)
  - Includes **rigid anatomy** (e.g., sternum, upper chest wall)
- If bolus covers part of the ROI, **adjust the ROI to avoid mismatches** in surface alignment.

**3. ✅ Account for Bolus in Planning CT and SGRT Reference**

- The **SGRT reference surface** must match the **planning CT** surface.
- If bolus is used during treatment:
  - It **must also be present** in the CT used to generate the SGRT reference surface
- Any inconsistency can lead to **false positives or misalignment in surface monitoring**

**4. ⚠️ Breath-Hold Stability and Monitoring**

- DIBH requires **precise and stable breath holds**.
- With bolus in place, patients might experience:
  - **Discomfort**
  - Difficulty **reproducing DIBH depth**
- **Use SGRT gating features** to:
  - Only deliver beam during predefined breath-hold windows
  - Ensure consistent **chest wall expansion** across fractions

**5. ⚠️ Skin Dose and Imaging Artifacts**

- Bolus can **affect SGRT optical tracking**:
  - Especially if it is **shiny**, **irregular**, or **moves slightly**
- Mitigation:
  - Use **matte-finish bolus**
  - Avoid air gaps
  - Secure bolus firmly
  - Perform **surface imaging QA** with bolus present before the first treatment

**6. ✅ Bolus Material Matters**

- Use bolus with **consistent optical reflectance** properties for SGRT visibility
- AAPM recommends choosing materials that are **SGRT-compatible** (e.g., tissue-equivalent, optically matte, non-slipping)

Moonshot-v1:

When performing surface-guided radiotherapy (SGRT) for deep-inspiration breath hold (DIBH) in breast radiotherapy treatment with bolus, several important considerations and precautions should be taken to ensure accurate treatment delivery and patient safety, as outlined in AAPM TG reports and related studies:

**Key Considerations and Precautions**

1. **Patient Setup and Positioning**:
   - **Two-Step Setup Procedure**: Use a two-step setup procedure to align the patient first in free-breathing (FB) and then transition to DIBH. This ensures minimal deformation and accurate positioning of the breast and local lymph nodes.
   - **Arm and Chin Alignment**: Ensure proper alignment of the arm and chin during the FB setup to minimize breast deformation and accurately reproduce the positions of local lymph nodes.
2. **Region of Interest (ROI) Definition**:
   - **Uniform ROI Definition**: Define a large ROI that covers the ipsilateral breast and part of the contralateral breast to ensure robust surface matching and minimize ambiguity. This ROI should include stable areas like the sternum and provide sufficient visible surface area for real-time monitoring.
3. **Monitoring and Adjustments**:
   - **Vertical Shift Monitoring**: Closely monitor the vertical shift of the surface from FB to DIBH, as this directly correlates with chest wall separation from the heart. The goal is to achieve minimal residual shift in the vertical direction.
   - **Real-Time Motion Monitoring**: Use real-time surface imaging to monitor patient motion during treatment. If motion exceeds a set tolerance (typically ±3 mm), the beam should be held until the patient returns to the correct position.
4. **Bolus Application**:
   - **Bolus Placement**: If a bolus is used, it should be applied quickly within a DIBH and a new reference surface captured immediately. This ensures accurate setup and motion monitoring regardless of bolus application.
   - **Bolus Material and Imaging**: Use bolus materials that can be effectively imaged by the optical surface imaging system. For example, sticky bolus with an outer white-cloth surface can be used for better imaging.
5. **Safety and Patient Comfort**:
   - **Breath-Hold Duration**: Ensure that patients can comfortably hold their breath for the required duration (typically around 20 seconds) without significant discomfort.
   - **Safety Mechanisms**: Equip the patient with a safety button to terminate the treatment cycle if they experience breathing difficulties. The treating physician should also monitor the patient’s breathing from the console.
6. **Technical Considerations**:
   - **Surface Imaging System**: Use advanced surface imaging systems like AlignRT, which provide 3D/4D surface guidance for both patient setup and DIBH gating. These systems can communicate with the linear accelerator to apply SGRT shifts and gate the radiation beam.
   - **Daily Reference Capture**: Always capture a new on-site reference surface after patient setup, regardless of whether bolus is used. This resets any residual setup uncertainties and simplifies clinical operations.

**Summary**

By following these guidelines, the accuracy and reproducibility of DIBH treatment can be significantly improved, ensuring optimal sparing of critical organs like the heart while delivering the prescribed dose to the target volume. Proper patient setup, ROI definition, real-time monitoring, and careful bolus application are crucial for successful SGRT in breast radiotherapy.

Questions16 (TG284)

According to AAPM TG reports, in a monthly quality assurance test, what is the tolerance of laser marking accuracy of an MRI simulator?

RTPhy-ChatBot:

The AAPM TG-284 report recommends a tolerance of ±2 mm for laser marking accuracy on an MRI simulator. However, with high precision laser localizer systems commercially available, a tolerance of ±1 mm should be achievable for both laser alignment and orthogonality tests. It is important to note that specific needs for SRS should be obtained by consulting the ongoing AAPM TG-117 report focused on this special use case. Additionally, during initial MR simulator equipment evaluation, it is crucial for a QMP to ensure that vendor-provided specifications of magnetic field homogeneity and gradient nonlinearity are verified. The quality assurance guidelines and tolerances recommended by the upcoming AAPM TG-284 report should be followed for producing images with the highest spatial fidelity possible in radiotherapy applications. Furthermore, image registration quality assurance and long-term accuracy are essential areas that require ongoing attention but have not yet reported specific test frequencies or achievable tolerances. The AAPM TG-179 recommends that tolerances for couch motions match those specified in TG-142, but difficulties in differentiating soft tissues can arise with CT-based image-guidance systems, leading to the adoption of a two-staged image guidance approach. The end-to-end QC test described in Sec. IV G is recommended and tested on a daily basis for accurate couch motions.

Gemini-2.0-flash:

Based on my search, I could not find a specific AAPM Task Group (TG) report dedicated solely to MRI simulators in the context of radiotherapy. However, AAPM TG reports address quality assurance in radiation therapy, which could include aspects relevant to MRI simulators used for treatment planning.

For instance, AAPM TG-66 report, titled "Quality assurance for computed-tomography simulators and the computed-tomography-simulation process," mentions patient marking/positioning lasers as part of the QA procedures for CT simulators. While this report focuses on CT simulators, the concept of laser marking accuracy for patient positioning is also crucial for MRI simulators used in radiation therapy.

AAPM TG-142, "Quality assurance of medical accelerators," provides tolerances for various mechanical and dosimetric parameters of linear accelerators. Although this report doesn't directly address MRI simulators, it establishes a framework for QA tolerances in radiation therapy.

Without a specific TG report for MRI simulators, it's challenging to provide a definitive tolerance for laser marking accuracy in monthly QA. However, based on the general principles of accurate patient positioning in radiation therapy and the tolerances mentioned for CT simulators and linacs in other AAPM TG reports, a reasonable expectation for laser marking accuracy in an MRI simulator would likely be within **1-2 mm**.

It is recommended to consult the specific quality assurance protocols established by your institution and any guidelines provided by the MRI simulator vendor. These resources would offer the most accurate and relevant tolerances for laser marking accuracy in your specific clinical context.

GPT-4o:

According to the **American Association of Physicists in Medicine (AAPM) Task Group 284 (TG-284)** report, which provides comprehensive guidance for quality assurance (QA) of **MRI simulators**, the **monthly QA tolerance for laser marking accuracy** is:

**±2 mm**

This means that during monthly QA, the alignment between the MRI laser system (used for patient positioning) and the scanner’s imaging center should be within **2 millimeters**.

Moonshot-v1:

According to AAPM TG-284, the tolerance for laser marking accuracy in an MRI simulator during a monthly quality assurance test is ±2.0 mm. This tolerance applies to the accuracy of laser markings in the axial, sagittal, and coronal planes.

Key Points:

Laser Marking Accuracy:

The laser marking accuracy should be within ±2.0 mm for both axial and sagittal planes. This ensures that the laser markings are precise and reliable for patient setup and treatment planning.

Testing Method:

The accuracy can be tested using a phantom with internal landmarks. The phantom is aligned with the external lasers, and the MRI is acquired to determine the offset. The process is iterated until the tolerance is met.

Additional Considerations:

For high-precision laser localizer systems, a tighter tolerance of ±1.0 mm can be achievable. This higher precision may be necessary for specific use cases like stereotactic radiosurgery (SRS).

Questions17 (TG256)

According to AAPM TG reports, what are disadvantages of using a constant RBE in proton therapy?

RTPhy-ChatBot:

The use of a constant RBE in proton therapy has some disadvantages. One major disadvantage is that it may not accurately reflect the biological effects of proton beams, as RBE can vary depending on factors such as fraction size, total treatment dose, dose rate, and beam properties. This means that using a constant RBE could lead to underestimation or overestimation of the biological effect of the proton therapy in certain situations. Another disadvantage is that it may not account for the heterogeneity of LETd distributions within tumors and critical structures, which can result in significant variations in RBE values. For example, in intensity-modulated proton therapy (IMPT), the spot intensities and LETd distributions in the target are highly heterogeneous, which can lead to significant deviations from a constant RBE of 1.1. Additionally, clinical dosimetry is based on absorbed dose distributions in the target, and commercial treatment planning systems do not currently provide an option to compute dose distributions weighted by variable proton RBE. This means that using a constant RBE simplifies the process of converting photon doses into biologically equivalent proton doses for clinical trials and makes clinical dosimetry straightforward. However, it is important to note that while there are disadvantages to using a constant RBE in proton therapy, it has also been shown to be a reasonable approximation for many clinical situations. The continued use of a constant RBE of 1.1 has been endorsed by organizations such as the International Commission on Radiation Units and Measurements (ICRU) and the International Atomic Energy Agency (IAEA). Therefore, while using a constant RBE may not be the most accurate approach, it is currently the most practical one for many clinical situations. However, there are ongoing efforts to develop more sophisticated models that can account for variable RBE values in proton therapy, and these models may eventually lead to improved clinical outcomes by allowing for more precise dosing based on the biological effects of the proton beams.

Gemini-2.0-flash:

While there isn't a single AAPM TG report that solely focuses on the disadvantages of using a constant Relative Biological Effectiveness (RBE) of 1.1 in proton therapy, the limitations are discussed across several reports, particularly **AAPM TG-256** ("Report of the AAPM TG-256 on the relative biological effectiveness of proton beams in radiation therapy"). Based on the information in these reports and general knowledge in the field, here are the disadvantages:

- **Oversimplification of a complex biological effect:** The RBE of protons is not a fixed value but varies depending on several factors, including:
  - **Linear Energy Transfer (LET):** RBE generally increases with increasing LET, which changes along the proton beam path, especially at the distal edge (Bragg peak). A constant RBE ignores this significant variation.
  - **Tissue Type:** Different tissues exhibit varying radiosensitivities and can have different RBE responses to protons.
  - **Dose per Fraction:** RBE can be dose-dependent, particularly for late-responding tissues.
  - **Biological Endpoint:** The RBE can differ for various biological effects, such as cell killing, DNA damage, and mutation.
- **Potential for under- or overdosing the tumor:** If the actual RBE within the tumor is higher than 1.1, using a constant value could lead to delivering a lower biologically effective dose, potentially compromising tumor control. Conversely, if the RBE is lower in some parts of the tumor, the prescribed dose might be unnecessarily high.
- **Increased risk of normal tissue complications:** The RBE is known to increase at the distal edge of the proton beam where LET is highest. Assuming a constant RBE of 1.1 might underestimate the biological dose delivered to normal tissues beyond the tumor, potentially increasing the risk of late toxicities in these critical structures.
- **Limited ability to optimize treatment plans biologically:** A constant RBE prevents the integration of LET distribution into treatment planning optimization. This means regions of higher LET (and potentially higher biological effect) are not specifically targeted for dose escalation or preferentially spared in critical normal tissues.
- **Uncertainties in comparing proton therapy with photon therapy:** Clinical trials often compare proton and photon therapy outcomes using a constant RBE of 1.1 for protons. If the actual RBE varies, these comparisons may be inaccurate, making it difficult to draw definitive conclusions about the relative benefits of each modality.
- **May not reflect clinical reality:** Emerging clinical data and radiobiological studies suggest that the in vivo RBE can deviate significantly from 1.1 in certain situations, potentially impacting clinical outcomes that are not accounted for by a constant value.

In summary, while a constant RBE of 1.1 has been a practical standard in proton therapy, it is a simplification that introduces uncertainties and may lead to suboptimal treatment planning and outcome prediction. AAPM TG-256 acknowledges these limitations and emphasizes the need for a more nuanced approach to RBE in proton therapy, even while not recommending a specific variable RBE model for immediate clinical implementation due to current uncertainties. The field is actively researching to better understand and model RBE variations for potential clinical benefits in the future.

GPT-4o:

According to AAPM Task Group reports, particularly TG-256, using a constant RBE of 1.1 in proton therapy has several disadvantages.

First, it oversimplifies the biological effect of protons. RBE actually varies depending on factors like linear energy transfer (LET), tissue type, dose per fraction, and the biological endpoint being considered. Assuming it is constant ignores these important variations.

Second, it can lead to an underestimation of the biological dose to normal tissues, especially near the end of the proton range (the Bragg peak), where LET – and thus RBE – tends to be higher. This can increase the risk of damage to nearby healthy tissues and organs at risk.

Third, it may overestimate the effectiveness of the dose delivered to the tumor in lower LET regions, potentially reducing tumor control.

Fourth, it limits the accuracy of advanced treatment planning. As proton therapy becomes more precise and biologically informed, the use of a constant RBE becomes a limiting factor and does not support biologically optimized planning approaches.

Lastly, clinical outcomes such as unexpected toxicities have shown that the constant RBE model may not adequately reflect the real biological effects, especially in sensitive structures near high-LET regions.

In summary, while using a constant RBE simplifies clinical workflows, it introduces uncertainties that can affect both treatment effectiveness and safety.

Moonshot-v1:

According to AAPM TG-256 and related studies, using a constant relative biological effectiveness (RBE) in proton therapy has several disadvantages:

**Disadvantages of Using a Constant RBE**

1. **Suboptimal Treatment Plans**:
   - Using a constant RBE may lead to suboptimal treatment plans that do not fully exploit the potential of proton therapy. This can result in less effective dose delivery to the target volume and potentially higher doses to normal tissues.
2. **Inaccurate Dose Distribution**:
   - The assumption of a constant RBE does not account for the spatial variations in RBE within the target volume and surrounding tissues. This can lead to inaccuracies in the dose distribution, potentially resulting in under- or overdosing of critical structures.
3. **Increased Toxicity and Reduced Efficacy**:
   - Ignoring the spatial and dosimetric variations in RBE can lead to increased toxicity in normal tissues and reduced efficacy in tumor control. This is particularly concerning for tissues with known low α/β ratios.
4. **Complexity in Treatment Planning**:
   - Modern proton therapy techniques, such as pencil beam scanning (PBS), produce more complex dose distributions compared to passive scattering. The constant RBE assumption may not accurately reflect the biological effect of these complex dose distributions.
5. **Misleading Interpretation of Outcome Data**:
   - When tissue- and spatially variant RBE variations are neglected, the interpretation of treatment outcome data can be misleading. This can affect the assessment of treatment efficacy and the incidence of side effects.

**Recommendations**

1. **Variable RBE Models**:
   - There is a need to develop and implement variable RBE models that account for dose, linear energy transfer (LET), and tissue-specific biological effects. This can improve the accuracy of dose planning and delivery.
2. **Clinical Data Collection**:
   - It is important to collect clinical data to reconstruct RBE doses and correlate them with clinical outcomes. This will help in understanding the true impact of RBE variations on treatment efficacy and toxicity.
3. **Advanced Treatment Planning Systems**:
   - Treatment planning systems should be updated to incorporate variable RBE models and LET distributions. This will allow for more accurate biological dose optimization.
4. **Robust Planning Techniques**:
   - Robust planning techniques should be used to account for uncertainties in RBE and LET. This can help in creating treatment plans that are less sensitive to these variations.

In summary, while the use of a constant RBE simplifies treatment planning, it may not fully reflect the biological effects of proton therapy. Implementing variable RBE models and collecting more clinical data are essential steps to improve the accuracy and efficacy of proton therapy.

Questions18 (TG238)

According to AAPM TG reports, what is the difference between analytic calibration model and pose determination model for a C-arm CBCT system?

RTPhy-ChatBot:

According to the provided context from AAPM TG Report 238, both analytic calibration model and pose determination model are used for geometric calibration in a C-arm CBCT system. However, they approach the calculation of system parameters differently.

The analytic calibration model is an exact description of all required system parameters for image reconstruction and pose determination. It mathematically describes the relationship between the 3D position of the physical phantom and the measured image of the phantom at the detector. This approach can be broadly classified into two methods: those based on non-linear optimization and those based on a direct analytical solution. The calibration phantoms used for this method are specific to the derived method and vary in shape, marker size, and number of markers.

On the other hand, the pose determination model describes all system parameters in a calibration matrix containing extrinsic and intrinsic parameters. Although these parameters can be estimated from projection matrices, the results can be unstable due to challenges in estimating the source to detector distance, which can vary due to minor instabilities. The methods suggested for this approach also range in their mathematical formulation but are founded on geometrical descriptions relating the 3D position of the physical phantom to the measured image at the detector.

In summary, both analytic calibration model and pose determination model serve the same purpose of geometric calibration in a C-arm CBCT system, but they differ in their mathematical approaches and methods for calculating system parameters. The choice between these two models depends on the specific requirements and constraints of the C-arm CBCT system being used.

Gemini-2.0-flash:

While AAPM Task Group (TG) reports, particularly **TG-238** on "3D C-arms with volumetric imaging capability," discuss geometric calibration for C-arm CBCT systems, they don't explicitly use the distinct terms "analytic calibration model" and "pose determination model" as separate, named categories. However, we can understand these concepts based on the descriptions of geometric calibration processes and the broader literature in the field of C-arm CBCT calibration.

Here's a breakdown of the potential differences between what might be referred to as an "analytic calibration model" and a "pose determination model" in the context of C-arm CBCT geometric calibration:

**1. Analytic Calibration Model:**

- **Focus:** This type of model aims to determine the **intrinsic and extrinsic geometric parameters** of the C-arm CBCT system through a well-defined mathematical framework.
  - **Intrinsic parameters** describe the internal characteristics of the detector, such as pixel size, principal point, and potential detector distortions.
  - **Extrinsic parameters** describe the spatial relationship between the X-ray source, the detector, and the system's mechanical components (like the rotation axis and isocenter). These include source-to-detector distance (SDD), source-to-isocenter distance (SID), detector tilt and offset, and the projection geometry for each view angle.
- **Methodology:** Analytic calibration typically involves imaging a **calibration phantom** with known geometric properties (e.g., a grid of markers, spheres in a specific arrangement). The 2D projections of these markers are then used in a mathematical optimization process to solve for the geometric parameters. This often involves minimizing the reprojection error – the difference between the projected locations of the 3D markers based on the model and their actual locations in the 2D images.
- **Outcome:** The result is a set of fixed parameters that describe the system's geometry. These parameters are then used in the reconstruction algorithm to produce geometrically accurate 3D images.
- **AAPM TG Relevance:** TG-238 discusses the need for accurate geometric calibration and mentions the use of calibration phantoms and vendor-specific procedures to determine these fundamental geometric parameters. The mathematical models used by vendors to perform this calibration would fall under the umbrella of an "analytic calibration model."

**2. Pose Determination Model:**

- **Focus:** This type of model is more concerned with **determining the precise spatial pose (position and orientation)** of the C-arm (specifically the X-ray source and detector) *during* the acquisition of a CBCT scan. This is particularly important for systems where the mechanical motion of the C-arm might not be perfectly reproducible or where external factors could influence its position.
- **Methodology:** Pose determination can involve various techniques:
  - **Tracking external markers:** Optical or electromagnetic tracking systems can monitor the position and orientation of markers attached to the C-arm components during the scan.
  - **Using onboard sensors:** Some advanced systems might incorporate sensors (e.g., encoders, inclinometers) to provide real-time feedback on the C-arm's position.
  - **Self-calibration techniques:** These methods might use the projection data itself to simultaneously estimate the object being imaged and the system's pose without relying solely on a pre-calibrated geometric model.
- **Outcome:** Instead of a fixed set of geometric parameters, a pose determination model provides a dynamic record of the C-arm's position and orientation for each projection angle acquired. This information can then be used in more sophisticated reconstruction algorithms that account for the actual, potentially non-ideal, acquisition trajectory.
- **AAPM TG Relevance:** While TG-238 emphasizes the importance of geometric calibration, it also acknowledges the complexities of C-arm motion and potential deviations from ideal trajectories. Advanced techniques for pose determination could be seen as a way to enhance the accuracy of the geometric information used in reconstruction, especially in scenarios where mechanical inaccuracies are a concern. Some research in the field, though not explicitly detailed as a separate "pose determination model" in the context of standard calibration in TG reports, aligns with this concept.

In conclusion, while AAPM TG reports like TG-238 focus on the essential geometric calibration of C-arm CBCT systems using phantoms to establish fundamental geometric parameters (akin to an "analytic calibration model"), the concept of a "pose determination model" addresses the dynamic tracking of the C-arm's actual position during a scan. This distinction is important for achieving high geometric accuracy, especially in systems with complex or potentially variable mechanical motion. Advanced C-arm CBCT systems might incorporate elements of both approaches to ensure optimal image quality and geometric fidelity.

GPT-4o:

According to AAPM Task Group reports such as **TG-179**, there are two main approaches for **geometric calibration** of a **C-arm CBCT system**: the **analytic calibration model** and the **pose determination model**. These models are used to determine the spatial relationship between the X-ray source, the detector, and the imaging isocenter during CBCT image acquisition.

Here is the difference between the two:

**1. Analytic Calibration Model:**

- This method assumes that the system geometry (i.e., source and detector trajectories) follows an **ideal or known analytic path**, such as a perfect circular or helical arc.
- Calibration involves measuring a small number of parameters, such as the source-to-axis distance (SAD), source-to-detector distance (SDD), and detector offsets.
- It is **faster and simpler**, but it assumes that the mechanical system is well-behaved and that any deviations from the ideal path are minimal.
- This model may not fully account for mechanical flex, sagging, or other real-world imperfections in the C-arm motion.

**2. Pose Determination Model:**

- This method does **not assume an ideal trajectory**. Instead, it uses a **calibration phantom with known marker positions** to determine the actual position (pose) of the X-ray source and detector for each projection image.
- It reconstructs the source-detector geometry from the **measured positions of markers** seen in the acquired projection images.
- It is **more flexible and accurate**, especially for systems where mechanical deviations (e.g., gantry flex or non-circular paths) are significant.
- It is commonly used in practice for more precise CBCT systems, especially when accurate 3D imaging and registration are critical.

In summary, the analytic model is based on an assumed ideal geometry and is simpler, while the pose determination model directly calculates actual geometry from image data and is more accurate for systems with non-ideal mechanics.

Moonshot-v1:

According to AAPM TG-238 and related studies, the differences between the analytic calibration model and the pose determination model for a C-arm CBCT system are as follows:

**Analytic Calibration Model:**

- **Description**: This model aims to describe the geometry of the imaging system exactly by providing a numerical value for all defined system parameters. It involves complex geometric descriptions relating the 3D position of a physical phantom to the measured image of the phantom at the detector.
- **Advantages**:
  - Provides an exact description of the system geometry.
  - Can be used for precise calibration when all parameters are accurately estimated.
- **Challenges**:
  - The results can be unstable due to challenges in estimating certain parameters, such as the source-to-detector distance, from projection matrices.
  - Requires a detailed understanding of the system's geometric relationships and often involves complex mathematical formulations.
  - The calibration process can be time-consuming and may require specific calibration phantoms with precise marker configurations.

**Pose Determination Model:**

- **Description**: This model is based on the pinhole camera model and describes the system parameters in a calibration matrix containing both extrinsic and intrinsic parameters. It focuses on determining the pose (position and orientation) of the C-arm system relative to a reference coordinate frame.
- **Advantages**:
  - Simplifies the calibration process by using a linear transformation of homogeneous coordinates to model the relationship between 3D and 2D coordinates.
  - Allows for efficient optimization of the projection matrix, which minimizes the error between known 3D calibration objects and acquired projection images.
  - Can be more robust in handling variations in the system's pose and is widely used in practice.
- **Challenges**:
  - May not provide as detailed a description of the system geometry as the analytic model.
  - The accuracy of the pose determination depends on the quality of the calibration data and the optimization process.
  - Requires a good initial estimate of the system's pose to achieve accurate results.

**Summary:**

- The **analytic calibration model** provides a detailed and exact description of the system geometry but can be complex and challenging to implement accurately.
- The **pose determination model** offers a more simplified and robust approach to calibration, focusing on optimizing the system's pose relative to a reference frame.

Both models have their own strengths and are used depending on the specific requirements and constraints of the calibration process for C-arm CBCT systems.

Questions19 (TG191)

According to AAPM TG reports, what should be paid attention to when using TLDs or OSLs in the measurement of skin dosimetry?

RTPhy-ChatBot:

According to AAPM TG reports, skin dosimetry is a serious challenge due to the very superficial depth of only 0.07 mm recommended for measuring the most superficial skin layers. Thermoluminescent dosimeters (TLDs) and optically stimulated luminescent dosimeters (OSLDs) have been used for skin dose or surface dose measurements, but they typically require extrapolation due to their relatively thick size compared to the dose gradient. The high density of TLDs means that the effective point of measurement is downstream from the middle of the detector, leading to an overestimation of the surface dose by 10-40%. One technique for overcoming this is to make measurements with detectors of different thicknesses and extrapolate to the desired depth. However, even thin TLDs can still overestimate the surface dose significantly. Dot OSLDs have an effective point of measurement that is 0.8 mm deep, which also substantially overestimates the surface/skin dose. Therefore, it is important to consider these limitations when using TLDs or OSLDs for skin dosimetry measurements.

Gemini-2.0-flash:

While I don't have specific details from all AAPM TG reports regarding skin dosimetry measurements with TLDs and OSLs, **AAPM TG-191** provides comprehensive guidance on the use of these dosimeters in a clinical setting. Based on this report and general best practices in skin dosimetry, here are key considerations:

**1. Detector Characteristics and Energy Dependence:**

- **Thin Detectors:** For accurate skin dose measurements, it is crucial to use thin TLDs or OSLs to minimize perturbation of the radiation field and to ensure that the measurement reflects the dose at the skin surface or within the superficial layers of interest.
- **Energy Response:** Be aware of the energy dependence of the TLD or OSL material. The response of these dosimeters can vary with the energy of the incident radiation. Appropriate energy correction factors, determined through calibration with radiation beams of relevant energies, must be applied.
- **Calibration:** Each batch of TLDs or OSLs should be carefully calibrated against a traceable standard in a geometry that closely matches the measurement conditions. Calibration should be performed for the relevant energy range and dose levels expected in skin dosimetry.

**2. Placement and Contact with the Skin:**

- **Intimate Contact:** Ensure that the TLD or OSL dosimeter is in direct and firm contact with the skin surface. Any air gaps or separation can significantly affect the measured dose due to changes in backscatter and attenuation.
- **Securement:** The dosimeter must be securely fixed to the skin to prevent movement during irradiation, which could lead to inaccurate readings or loss of the detector. Consider using hypoallergenic tape or dressings that do not interfere with the radiation beam.
- **Contour Following:** On curved surfaces, use flexible dosimeters or multiple small dosimeters to accurately map the skin dose distribution. Rigid dosimeters on curved surfaces can lead to air gaps and inaccurate measurements.

**3. Environmental Factors and Handling:**

- **Temperature and Humidity:** TLD and OSL materials can be sensitive to temperature and humidity. Follow the manufacturer's recommendations for storage, handling, and exposure conditions to minimize fading or spurious signals.
- **Light Sensitivity (OSLs):** OSL dosimeters are sensitive to light. Handle and store them in dark conditions before and after irradiation to prevent unwanted stimulation and loss of signal.
- **Multiple Readings (TLDs):** Be aware that TLDs are typically read once, and the information is erased. If multiple readings are needed, multiple dosimeters must be used. OSLs, in some cases, can be partially read and re-analyzed, but this should be done with caution and according to the manufacturer's guidelines.

**4. Measurement Considerations:**

- **Number and Location of Detectors:** The number and placement of dosimeters should be sufficient to adequately characterize the skin dose distribution, especially in regions where dose gradients are expected.
- **Reference Points:** Clearly document the location of each dosimeter on the patient's skin with respect to anatomical landmarks or treatment field edges. This is crucial for correlating the measured dose with the intended treatment area.
- **Time of Measurement:** Record the start and end times of irradiation for each dosimeter to account for any potential variations in dose rate or treatment duration.

**5. Data Analysis and Uncertainty:**

- **Reader Calibration and Quality Control:** Ensure that the TLD or OSL reader is properly calibrated and maintained. Regular quality control checks should be performed to ensure the reliability of the readout process.
- **Background Correction:** Account for any background signal or zero-dose reading of the dosimeters.
- **Uncertainty Analysis:** A thorough uncertainty analysis should be performed, considering factors such as calibration uncertainty, energy dependence correction, reader precision, and variations in detector sensitivity.

**In summary, according to AAPM TG reports and general best practices, careful attention to detector selection, calibration, proper placement ensuring good skin contact, control of environmental factors, meticulous measurement techniques, and thorough data analysis with appropriate uncertainty assessment are paramount for accurate skin dosimetry using TLDs or OSLs.**

It's recommended to consult the specific AAPM TG reports, particularly TG-191, and the literature provided by TLD/OSL manufacturers for detailed protocols and recommendations relevant to your specific clinical application.

GPT-4o:

According to AAPM Task Group reports such as **TG-190** (on surface and buildup dose measurements) and **TG-158** (on dosimetry for patient-specific QA), when using **TLDs (thermoluminescent dosimeters)** or **OSLs (optically stimulated luminescent dosimeters)** for **skin dosimetry**, several important considerations should be kept in mind to ensure accurate and meaningful measurements:

1. **Energy Dependence**

TLDs and OSLs can have a response that varies with photon energy. Since surface or skin doses often involve lower-energy scatter and contaminant electrons, calibration should match the energy spectrum as closely as possible.

1. **Angular Dependence**

These detectors may show variation in response based on the angle of incident radiation. This is especially relevant for skin dosimetry since beams often strike the skin at oblique angles.

1. **Positioning and Buildup**

TLDs and OSLs must be placed very close to or directly on the skin surface. However, some types have a small amount of material (like encapsulation or filters) that adds **buildup**, which may **shift the effective measurement point deeper than the actual skin**. This must be accounted for when interpreting results.

1. **Detector Size and Spatial Resolution**

The physical size of TLDs or OSLs can average dose over several millimeters, which may smooth out steep gradients near the skin. For accurate skin dose, a smaller detector is preferred.

1. **Calibration and Readout**

Calibration must be done under similar conditions to those used clinically, including energy, field size, and geometry. TLDs and OSLs also require careful readout procedures to avoid introducing uncertainty through reader variability or fading effects (especially for OSLs).

1. **Environmental Sensitivity**

Both types of detectors are sensitive to environmental factors like light (OSLs) or heat (TLDs), and must be handled properly before and after irradiation to avoid data loss or degradation.

1. **Reusability and Fading**

OSLs and TLDs are reusable but can suffer from signal fading over time. Corrections may be needed depending on the delay between exposure and readout.

In summary, when using TLDs or OSLs for skin dosimetry, attention must be paid to energy and angular dependence, detector placement and buildup, proper calibration, and environmental handling to ensure accurate and reliable measurements.

Moonshot-v1:

According to AAPM TG reports, skin dosimetry is a serious challenge due to the very

When using TLDs (Thermoluminescent Dosimeters) or OSLDs (Optically Stimulated Luminescent Dosimeters) for skin dosimetry, several key considerations should be taken into account, as outlined in AAPM TG-191 and related studies:

**1. Material Selection and Calibration**

- **Material Properties**: Both TLDs and OSLDs are effective for point dosimetry, but their performance can vary. TLDs have been widely used for decades, while OSLDs offer advantages such as faster readout and reusability.
- **Calibration**: Both types of dosimeters should be carefully calibrated to ensure accurate dose measurements. Calibration should account for factors such as energy dependence, dose rate dependence, and angular dependence.

**2. Handling and Storage**

- **Light Sensitivity**: OSLDs must be kept in light-tight conditions prior to reading to prevent signal depletion. TLDs, on the other hand, need to be handled carefully to avoid surface contamination.
- **Environmental Conditions**: The dose sensitivity of OSLDs is not dependent on temperature in the range of 10-40 °C, making them suitable for a wide range of clinical environments.

**3. Measurement Accuracy and Precision**

- **Precision**: OSLDs can achieve better than 1% reproducibility in dose measurements, while TLDs typically achieve around 1.5%.
- **Energy Dependence**: Both TLDs and OSLDs should be tested for energy dependence to ensure accurate dose measurements across different photon energies.

**4. Special Considerations for Skin Dosimetry**

- **Surface Dose Measurement**: OSLDs are particularly well-suited for measuring surface dose due to their low intrinsic buildup.
- **In Vivo Dosimetry**: Both TLDs and OSLDs can be used for in vivo dosimetry, but OSLDs offer the advantage of being read long after irradiation, which can be beneficial for clinical workflows.

**5. Quality Assurance and Documentation**

- **Quality Assurance**: Regular quality assurance checks should be performed to ensure the dosimeters are functioning correctly. This includes checking for any signs of damage or contamination.
- **Documentation**: All calibration and QA procedures should be carefully documented for future reference and regulatory compliance.

**Summary**

When using TLDs or OSLDs for skin dosimetry, it is crucial to select the appropriate material based on the specific clinical needs, ensure proper calibration and handling, and maintain rigorous quality assurance procedures. OSLDs offer several advantages, including faster readout and better precision, making them a valuable tool for skin dosimetry applications.

Questions20 (TG157)

According to AAPM TG reports, what is the tolerance of output factors for a monte-carlo based treatment planning system?

RTPhy-ChatBot:

The tolerance of output factors for a Monte Carlo (MC) based treatment planning system should be checked against the commissioning data or treatment planning system baseline established during commissioning to ensure that the calculated dose is consistent with the intended dose. While it's not essential to duplicate all efforts made during commissioning, a smaller subset of output factors will suffice for accurate dose calculations. It's important to recognize that tolerances on individual machine parameters may vary somewhat between different departments depending on use and treatment objectives, but given uncertainties should be kept as low as reasonably achievable.

Output ratios, which are MC calculations of output performed in a measurement-like geometry, can be used to verify the accuracy of output factors. Output ratios should agree with measurements within 1% - 2% for square field sizes ranging from 3 to 40 cm, and comprehensive verification should include testing for small segmental fields located off-axis, which are commonly used in Intensity Modulated Radiation Therapy (IMRT) planning.

MC calculations of output should be performed in a measurement-like geometry, usually consisting of a central axis point dose estimate at a fixed depth in a water phantom for square field sizes defined by the collimating jaws. If the backscattered radiation into the monitor chamber is correctly modeled, it's possible to calculate output ratios to within 1% - 2% agreement with measurements over a range of square field sizes. Comprehensive verification of output ratios should include testing for small segmental fields located off-axis and testing against measurements of specially designed IMRT fields, such as junction narrow slit fields, in which the effects of small field dosimetry are magnified.

Gemini-2.0-flash:

While I cannot provide a definitive tolerance for output factors specifically for Monte Carlo-based treatment planning systems according to all AAPM TG reports, here's a summary of relevant information and considerations based on available reports:

AAPM TG reports generally recommend a tolerance of ±2% to ±3% for the constancy of the monitor output for photon and electron beams under reference conditions (e.g., TG-40, TG-142).

Direct measurements remain crucial for verifying the accuracy of the MC-calculated output factors during commissioning and routine quality assurance. The tolerance for the agreement between calculated and measured output factors should align with the general recommendations (±2% to ±3%).

In conclusion, while a specific tolerance solely for MC-calculated output factors might not be explicitly stated in all AAPM TG reports, the general tolerance for output constancy applies, and thorough validation against measurements is paramount to ensure the accuracy of Monte Carlo-based treatment planning systems. You should consult specific TG reports like TG-105 and TG-157 for detailed guidance on commissioning and QA of MC-based TPS.

GPT-4o:

According to **AAPM TG-114** and **TG-105**, which provide guidance on validation and commissioning of **Monte Carlo-based treatment planning systems (TPS)**, the **tolerance for output factors** is generally **±2%.**

**Details:**

- **Output factors** represent the relative dose output for various field sizes compared to a reference field (usually 10×10 cm²).
- In **Monte Carlo TPS**, output factors must be carefully validated against **measured data** during commissioning.
- A **±2% agreement** between calculated and measured output factors is considered acceptable.
- If discrepancies exceed this tolerance, further investigation and potential adjustments to modeling parameters or measurements are recommended.

**Additional Considerations:**

- For very small field sizes (e.g., in SRS or SBRT), tighter tolerances may not be achievable due to higher uncertainties in measurement and modeling. In such cases, **±3%** might be acceptable, but must be justified.
- Ensure that both **electron transport settings** and **phantom geometry** in the Monte Carlo simulation accurately reflect the measurement setup.

In summary, the standard tolerance for output factors in a Monte Carlo-based TPS is **±2%**, in line with general radiotherapy commissioning guidelines.

Moonshot-v1:

According to AAPM TG reports, the tolerance for output factors in a Monte Carlo-based treatment planning system is typically within **2%** of measured values. This tolerance level ensures that the calculated dose distributions are sufficiently accurate for clinical use.

**Key Considerations for Output Factors in Monte Carlo-Based Treatment Planning Systems**

1. **Accurate Modeling**:
   - The Monte Carlo simulation should accurately model the linear accelerator treatment head, including the primary photon source, flattening filter, and collimators. Accurate source modeling is especially important for small fields, which are commonly used in IMRT planning.
2. **Statistical Uncertainties**:
   - Statistical uncertainties can affect dose distributions, including output factors. These uncertainties should be minimized to ensure that the calculated dose is within the acceptable tolerance range.
3. **Experimental Verification**:
   - Experimental verification is crucial to validate the Monte Carlo simulations. This includes comparing calculated output factors with measured values over a range of field sizes and SSDs (source-to-surface distances) to ensure accuracy.
4. **Complex Dose Delivery Scenarios**:
   - Monte Carlo methods are particularly useful for complex dose delivery scenarios, such as IMRT, where conventional algorithms may not accurately account for scatter equilibrium and output ratio variations.

By adhering to these considerations, the Monte Carlo-based treatment planning system can provide accurate dose calculations, ensuring effective and safe radiation therapy treatments.
