# Supplementary material for: RTPhy‐ChatBot: A RAG‐Based intelligent assistant for radiotherapy physics using LLaMA3 and AAPM reports
Source: J Appl Clin Med Phys. 2025 Oct 9;26(10):e70263. doi: 10.1002/acm2.70263 (PMC12509246; doi:10.1002/acm2.70263)
Supplement: Supplementary file 2 — Supporting information [file ACM2-26-e70263-s002.docx]

Appendix B

Comprehensive response

Questions1:

According to AAPM TG reports, in a monthly QA procedure, what is the tolerance of photon beam proﬁle constancy of an IMRT machine?

Answer:

According to AAPM Task Group 142 (TG-142), the tolerance for photon beam profile constancy in a monthly QA procedure for an IMRT machine is: ±1%.

Question2:

According to AAPM TG reports, what are the advantages of kVCT compared to MVCT?

Answer:

According to AAPM TG-179 and supporting reports (such as TG-104 and TG-180), kilovoltage CT (kVCT) offers several advantages over megavoltage CT (MVCT) in the context of image-guided radiation therapy (IGRT) and adaptive radiation therapy (ART):

Key Advantages of kVCT over MVCT

1. Superior Soft Tissue Contrast

kVCT uses lower-energy photons, which interact primarily via the photoelectric effect, enhancing soft tissue contrast. MVCT operates in the megavoltage range, where Compton scattering dominates, leading to lower contrast between soft tissues. This improved contrast is critical for target visualization and accurate contouring, especially in soft-tissue-dominated anatomical regions (e.g., prostate, head and neck).

2. Lower Imaging Dose

kVCT typically delivers a lower radiation dose than MVCT, particularly when low-dose protocols are used. Lower dose imaging helps reduce cumulative exposure across multiple treatment sessions, which is especially beneficial for pediatric patients or those undergoing long treatment courses.

3. Higher Spatial Resolution

kVCT systems generally have finer detector elements and narrower slice thickness, enabling sharper images and better delineation of anatomical structures. This is crucial for precise patient positioning and contour accuracy.

4. Faster Acquisition Times

Many kVCT systems, particularly those derived from diagnostic CT technology, offer faster scan speeds than MVCT systems. Shorter scan durations reduce the chance of patient motion and improve clinical workflow efficiency.

5. Improved Image Registration and Integration

kVCT images are acquired with similar energy and contrast characteristics as the planning CT, which is typically also a kVCT. This makes image registration more accurate, especially for deformable image registration, facilitating adaptive radiotherapy workflows like dose accumulation and contour propagation.

Question3:

According to AAPM TG reports, what are the low contrast resolution standards for kV-CBCT, fan beam MVCT, and MV-CBCT, respectively?

Answer:

According to AAPM TG-179, which provides guidance on quality assurance for CT-based image-guided radiation therapy (IGRT), the low contrast resolution capability varies significantly between imaging modalities due to differences in x-ray energy, detector design, and system geometry. Here's a modality-wise breakdown based on TG-179 and corroborated data:

1. kV-CBCT (Kilovoltage Cone-Beam CT)

Standard: Should be able to detect 7 mm diameter objects at approximately 1% contrast difference.

2. Fan Beam MVCT (Megavoltage Fan-Beam CT)

Standard: Detectability of objects with 13 mm diameter at 2% contrast difference.

3. MV-CBCT (Megavoltage Cone-Beam CT)

Standard: Capable of resolving 20 mm (2 cm) diameter objects with 1% contrast difference.

Question4:

According to AAPM TG reports, what is the difference between PS, PBS, and US in particle radiotherapy?

Answer:

1. Passive Scattering (PS)

Beam Delivery: Uses physical devices like scattering foils, range modulators, apertures, and compensators to spread and shape the proton beam.

Dose Distribution: Creates a uniform dose across the treatment field using a Spread-Out Bragg Peak (SOBP).

Advantage: Simple, robust, less sensitive to motion

Disadvantages: Requires patient-specific hardware, limited conformality, and higher neutron contamination

2. Uniform Scanning (US)

Beam Delivery: Uses scanning magnets to deliver a larger beam spot in a pattern (e.g., raster, spiral) to uniformly scan the treatment field at each energy layer.

Dose Distribution: Similar to PS but delivered via scanned beams instead of scatterers; energy layers are switched to stack dose depth-wise.

Advantages: More conformal than PS, No patient-specific apertures needed

Disadvantages: Not capable of intensity modulation (IMPT), Intermediate sensitivity to motion

3. Pencil Beam Scanning (PBS)

Beam Delivery: Uses narrow pencil beams scanned spot-by-spot and layer-by-layer by steering magnets; each spot can be individually controlled for position, dose, and energy.

Dose Distribution: Highly conformal with full 3D control, enabling IMPT (Intensity Modulated Proton Therapy).

Advantages: Best conformality and OAR sparing, No hardware customization per patient

Disadvantages: Most sensitive to motion, Requires robust planning and motion management, Potential interplay effects with respiration

Question5:

Answer:

Respiratory motion can significantly affect the accuracy of photon radiotherapy, especially for tumors in the thoracic and abdominal regions. AAPM Task Group 76 (TG-76), along with other related reports, describes several techniques to manage this motion to improve target coverage and reduce unnecessary dose to normal tissues.

1. Motion-Encompassing Methods: This method involves accounting for the full extent of tumor motion by expanding the Planning Target Volume (PTV) to include the entire range of respiratory motion. The motion is typically evaluated using 4D-CT during treatment planning, which allows the creation of an Internal Target Volume (ITV) that encompasses the motion envelope. This is the simplest method and does not require any active intervention during treatment. However, it increases the volume of irradiated healthy tissue and may not be ideal for high-precision treatments.

2. Breath-Hold Techniques: In this method, patients hold their breath during treatment delivery to immobilize internal anatomy and reduce or eliminate tumor motion. Techniques include voluntary breath-hold and assisted methods like Deep Inspiration Breath-Hold (DIBH) or Active Breathing Control (ABC). These techniques are particularly useful for sparing nearby organs at risk, such as the heart in left-sided breast cancer. The effectiveness of this method depends on the patient's ability to hold their breath consistently and for sufficient duration.

3. Respiratory-Gated Techniques: These methods synchronize radiation delivery with a specific phase of the patient’s respiratory cycle, such as end-inhalation or end-exhalation, when the tumor position is most stable. External monitoring systems (like infrared markers or spirometry) are used to track the respiratory cycle. 4D-CT is used during planning to determine the appropriate gating window. This approach reduces PTV margins and exposure to normal tissues but typically increases treatment time and requires stable, reproducible breathing.

4. Forced Shallow Breathing and Abdominal Compression: These techniques aim to limit the amplitude of respiratory motion. In shallow breathing, the patient is coached to breathe gently and regularly. Abdominal compression uses a mechanical device to restrict diaphragmatic movement. These methods reduce tumor motion without requiring breath-holds but may not eliminate motion completely. Compression can be uncomfortable for some patients.

5. Respiration-Synchronized Techniques (Real-Time Tumor Tracking): This advanced method involves tracking the tumor or a surrogate in real-time and adjusting the radiation beam dynamically to follow the tumor as it moves. It may use implanted fiducial markers, real-time imaging, or external surrogates. This technique offers highly precise dose delivery with minimal margins but requires sophisticated equipment and rigorous quality assurance.

According to AAPM TG-76, respiratory motion management should be considered if the tumor motion exceeds 5 mm in any direction or if there is a potential for significant reduction in dose to surrounding normal tissues. Accurate motion assessment, often using 4D-CT, is essential to guide the selection of the appropriate motion management strategy. All techniques require proper implementation and quality assurance to ensure safe and effective treatment.

Question6

According to AAPM TG reports, in radiotherapy, how much dose should the image-guided process produce? How to reduce the dose of the image guidance process?

Answer:

In image-guided radiotherapy (IGRT), the imaging dose should be carefully managed to ensure precise patient setup while minimizing unnecessary radiation exposure. According to AAPM Task Group reports, especially TG-75, TG-180, and TG-179, the imaging dose should ideally be kept below 5% of the therapeutic target dose. Even if the imaging dose is within this limit, the ALARA (As Low As Reasonably Achievable) principle should always be followed.

The actual dose from image guidance can vary significantly depending on several factors, including the imaging modality (e.g., kV or MV imaging, CBCT), imaging protocol (e.g., scan length, number of projections, mAs, kVp), and frequency of imaging. While kV imaging generally results in lower doses, MV imaging and frequent CBCT scans can contribute a considerable cumulative dose during the course of treatment.

To reduce imaging dose, AAPM reports recommend several strategies:

1. Optimize imaging protocols by using the lowest acceptable mAs and kVp, minimizing the scan length, reducing the number of projections or images, and increasing pitch for helical scans.

2. Use low-dose imaging modes available on modern IGRT systems.

3. Limit the frequency of imaging based on clinical necessity and individual patient risk assessment, rather than applying uniform daily imaging protocols.

4. Use 2D kV radiographs instead of CBCT when high-resolution volumetric images are not necessary.

5. Employ iterative reconstruction algorithms, which allow for lower dose while maintaining image quality.

6. Limit the imaging field of view and only scan the necessary anatomical region.

7. Use surrogate markers and deformable image registration techniques when appropriate to reduce the need for repeated volumetric imaging.

8. Ensure all staff involved in IGRT are trained to understand dose management strategies and the importance of dose tracking.

9. Continuously review and improve IGRT protocols based on clinical outcomes and new technology to ensure doses remain ALARA.

10. Document and monitor the cumulative imaging dose for each patient throughout the treatment course.

By applying these strategies, imaging doses in IGRT can be effectively managed to maintain high treatment accuracy while minimizing radiation exposure to patients.

Question7

According to AAPM TG reports, in stereotactic radiotherapy, what specific quality assurance programs are available for small field dosimetry?

Answer:

In stereotactic radiotherapy (SRT), accurate dosimetry of small fields is crucial due to the high dose per fraction and the small size of treatment fields, often less than 10 mm in diameter. These small fields pose significant challenges such as loss of lateral electronic equilibrium, detector volume averaging, detector positioning uncertainties, and source occlusion. To address these challenges, the AAPM Task Group reports provide integrated QA recommendations rather than a standalone QA program specifically for small field dosimetry. The most relevant reports include TG-101, TG-106, TG-142, TG-178, and especially TG-155, which provides detailed recommendations on how to handle fields smaller than 3cm×3cm.

TG-155 provides detailed recommendations for small field output factor measurements, detector selection, and the application of correction factors. Output factor measurement is a critical part of small field dosimetry QA. The output factor represents the dose output of a given field size relative to a standard 10cm×10cm field. For small fields, output factors are influenced by partial occlusion of the source and detector response issues. Therefore, specialized detectors such as unshielded diodes, plastic scintillators, or microDiamond detectors should be used. Due to the perturbations caused by detector characteristics in small fields, appropriate correction factors—such as those provided by TG-155 and IAEA TRS-483—must be applied to achieve accurate output factor measurements. These measurements are essential for treatment planning system commissioning, periodic QA, and patient-specific QA.

Other critical QA components recommended across the TG reports include beam data commissioning with high-resolution detectors (TG-106), end-to-end testing using anthropomorphic phantoms (TG-101), regular calibration and validation of the dosimetry system (TG-142), and mechanical accuracy checks such as isocenter verification and MLC positioning accuracy. Daily QA includes basic mechanical and imaging checks; monthly QA includes output constancy checks using small field detectors. Treatment planning algorithms must also be validated specifically for small fields to ensure accurate dose calculation.

In conclusion, the AAPM TG reports integrate all necessary procedures into existing QA frameworks. These steps ensure the accuracy and safety of small field dose delivery in stereotactic radiotherapy.

Question8

According to AAPM TG reports, what are the sources of error in image registration? How to mitigate error?

Answer:

Sources of Error in Image Registration:

1. Input-related errors: Poor image quality such as noise, low contrast, motion artifacts, differences in scan parameters like voxel size, slice thickness, or field of view, metal artifacts, and anatomical changes between sessions (like bladder filling or tumor changes) can all introduce registration errors.

2. Algorithm-related errors: Using the wrong type of registration (e.g., rigid instead of deformable), poor tuning of algorithm parameters, or limitations in the algorithm’s ability to handle complex deformations can cause inaccuracies. Errors may also arise when extrapolating deformation fields outside the imaged volume.

3. Output-related errors: Misinterpretation or misapplication of the deformation field, contour propagation inaccuracies, or using registration results for dose warping or guidance without validation can introduce further errors.

4. User or process-related errors: Errors from manual selection of landmarks, inconsistencies in contouring between image sets or observers, and a lack of validation or QA of registration results are also common.

How to Mitigate Errors:

1. Improve image quality and consistency: Use standard imaging protocols, reduce motion artifacts using techniques like breath-hold or respiratory gating, reduce metal artifacts, and ensure consistent anatomical coverage.

2. Use of immobilization devices: Employ proper and reproducible immobilization systems such as thermoplastic masks, vacuum cushions, or body molds during both planning and treatment imaging to reduce patient motion and ensure consistent positioning between sessions. This helps reduce intra-fraction and inter-fraction variability, which is critical for accurate registration.

3. Choose the correct algorithm and tune parameters: Select appropriate registration algorithms (rigid, affine, deformable) depending on the clinical situation, anatomy, and expected changes. Validate and tune parameters for specific use cases.

4. Validate and verify the output: Always inspect the registration results visually and use quantitative validation tools such as Dice similarity coefficient, target registration error, or landmark matching. Avoid relying solely on software output.

5. Perform quality assurance and commissioning: Commission registration tools using phantoms with known geometries, perform regular QA checks, and document any manual interventions or overrides to ensure consistency and traceability.

6. Standardize workflow and train staff: Ensure staff are trained in using registration software, interpreting results, and reviewing output. Define clinical criteria for acceptable registration and include residual registration uncertainty in planning margins such as PTV expansion.

Question9

According to AAPM TG reports, how to measure the dosimetric indices in small fields?

Answer:

According to AAPM TG reports such as TG-155, the following steps are recommended for measuring dosimetric indices in small fields:

1. Use appropriate detectors. Small volume detectors such as micro-ionization chambers, silicon diodes, plastic scintillators, diamond detectors, and radiochromic films are recommended. These detectors help reduce volume averaging and improve spatial resolution. Large conventional ion chambers should be avoided in very small fields due to their inability to accurately measure steep dose gradients.

2. Apply correction factors. Output measurements in small fields require correction factors due to changes in detector response, lack of lateral charged particle equilibrium, and source occlusion effects. Use field output correction factors as specified in IAEA TRS-483 or TG-155 to adjust the readings.

3. Use the intermediate field method. For output factor measurements, start with a relatively larger field where your detector works accurately, then use intermediate fields to scale down to the smaller field using appropriate detectors and correction factors.

4. Use a high-resolution scanning setup. Depth dose (PDD), profiles, and beam data should be acquired using step sizes of 1 mm or less. This is necessary to capture steep dose gradients, especially near field edges and in the penumbra region.

5. Perform measurements in a proper phantom. Water phantoms are preferred for dosimetric accuracy. Ensure full buildup and lateral scatter conditions. Solid water phantoms can be used if carefully calibrated and corrected.

6. Ensure accurate detector positioning. Small fields have narrow and steep dose distributions, so the detector must be carefully aligned at the correct depth and position to avoid significant errors.

7. Validate with Monte Carlo simulation. Monte Carlo methods provide high-accuracy modeling and are useful for comparison or generation of correction factors when measuring in very small fields.

8. Perform end-to-end testing. Run the full clinical workflow from CT simulation to treatment delivery using a phantom and suitable detector (such as film or scintillator) to verify dosimetric accuracy in small fields.

9. Use more than one detector. When possible, use at least two different types of detectors to cross-check results, as each may have its own strengths and weaknesses in small field measurements.

Question10

According to AAPM TG reports, what are the main sources of out-of-field dose in radiation therapy? What factors affect the out-of-field dose?

Answer:

According to AAPM Task Group reports, especially TG-158, the main sources of out-of-field dose in radiation therapy are:

1. Patient Scatter: Scattered radiation from interactions of the primary beam within the patient's body. This is usually the dominant source near the field edge.

2. Head Leakage: Radiation that leaks through the shielding of the linac treatment head. This becomes the main contributor at larger distances from the treatment field.

3. Collimator Scatter: Radiation scattered off the jaws, MLCs, wedges, and other components in the beam path.

4. Room Scatter: Radiation that reflects off the walls, ceiling, and floor of the treatment room, contributing to dose farther away from the field.

5. Imaging Dose: Additional peripheral dose from kV or MV imaging used during image-guided radiation therapy.

Factors that affect the out-of-field dose include:

1. Field Size: Larger fields increase both patient and collimator scatter, raising out-of-field dose.

2. Beam Energy: Higher photon energies (>10 MV) can produce photoneutrons, adding to the dose. Neutron dose becomes a concern especially above 15 MV.

3. Treatment Technique: IMRT and VMAT require more monitor units, which increases head leakage and collimator scatter. More complex beam arrangements also spread scatter more broadly.

4. Distance from Field Edge: The dose drops rapidly with distance from the field. Near the edge, patient scatter dominates; farther out, head leakage becomes more important.

5. Linac Design: Variations in shielding, MLC design, and head construction between linac models affect leakage radiation.

6. Monitor Units (MU): More MUs mean longer beam-on times, leading to more leakage and scatter.

7. Patient Size and Anatomy: Larger or denser patients produce more scatter.

8. Use of Beam Modifiers: Wedges, compensators, and bolus can affect the pattern and amount of scattered radiation.

9. Collimator Orientation: Changing the collimator angle can change leakage and scatter patterns, affecting dose to organs at risk.

Understanding and minimizing out-of-field dose is essential for reducing the risk of secondary cancers and protecting healthy tissues, especially in pediatric and long-survival patients.

Question11

According to AAPM TG reports, in radiotherapy, how much impact does couch and different kinds of patient immobilization devices have on patient dose in radiation therapy?

Answer:

According to AAPM Task Group reports, particularly TG-176 and TG-148, both the treatment couch and various patient immobilization devices can significantly impact the dose delivered to patients in radiation therapy. The impact can be broken down into several key points:

1. The treatment couch, especially modern carbon fiber couches, can increase the skin dose and attenuate the therapeutic beam. For beams passing through the couch, the skin dose can increase by 20-40%, and attenuation of the beam can range from about 2% for perpendicular beams to as much as 16% for highly oblique angles. If the couch and its components like rails are not accounted for in the treatment planning system (TPS), this can lead to underdosage of the target volume by up to 35% in some IMRT plans.

2. Immobilization devices such as thermoplastic masks, vacuum bags, body cradles, and breast or belly boards can also increase skin dose and attenuate the beam. For example, vacuum bags can raise the surface dose from about 16% up to 68% depending on the material and beam energy. Attenuation from these devices is generally lower than from the couch, but can still reach up to 5%, and in some head and neck VMAT plans, ignoring their impact has led to dose deviations of up to 71% for certain dose metrics.

3. The dosimetric impact of these devices depends on several factors including the material (e.g., carbon fiber vs. denser plastics or metal), the thickness of the device, its position relative to the beam path, and the beam energy. Lower energy beams and oblique angles tend to produce more significant dose perturbations.

4. To minimize these effects, AAPM recommends including the couch and immobilization devices in the CT simulation and modeling them in the TPS. This ensures their impact on dose distribution is properly calculated. Using low-density materials, avoiding beam paths through dense components, and performing QA measurements when needed are also recommended strategies.

5. In proton therapy, the couch and immobilization devices can act as range shifters, altering the range of the beam. Their water-equivalent thickness must be accounted for during planning to avoid incorrect dose delivery.

In summary, if the treatment couch and immobilization devices are not properly considered in the treatment planning process, they can cause clinically significant dose errors, including underdosage to the tumor and increased dose to the skin and normal tissues.

Question12

According to AAPM TG reports, in proton therapy, how to reduce the impact of high-atomic number materials?

Answer:

To reduce the impact of high-atomic number (high-Z) materials in proton therapy, AAPM Task Group reports and related literature suggest the following strategies:

1. Avoid beam paths through high-Z materials: Select beam angles that steer clear of metal implants to reduce scattering and range uncertainty.

2. Use robust treatment planning: Apply robust optimization techniques that account for range and setup uncertainties, as recommended in AAPM TG-185. This helps maintain target coverage even in the presence of metal.

3. Override CT numbers with known material values: High-Z materials can create artifacts on CT images, leading to errors in dose calculation. Override the CT values in the treatment planning system using known stopping power ratios (e.g., SPR of titanium ≈ 3.2–3.4).

4. Use advanced imaging techniques: Dual-energy CT (DECT) or metal artifact reduction (MAR) algorithms help reduce artifacts and improve accuracy of tissue density estimation. MRI can be used for better soft tissue visualization if safe.

5. Employ Monte Carlo dose calculation: Monte Carlo methods provide highly accurate modeling of proton interactions in heterogeneous media, especially around high-Z materials. Recommended in TG-256 and TG-185.

6. Add distal margins or range compensators: In passive scattering systems, range compensators can help. In pencil beam scanning (PBS), include extra distal margins to account for range uncertainties.

7. Perform patient-specific QA: Use phantoms with embedded metals to verify dose distribution through end-to-end tests. Tools include ion chambers, film, and 3D dosimeters.

These methods aim to minimize range uncertainty and dose errors caused by high-Z materials and improve safety and accuracy in proton therapy.

Question13

According to AAPM TG reports, during radiotherapy, what is the maximum dose allowed for a pacemaker? During the treatment process, what should be paid attention to?

Answer:

According to AAPM Task Group reports, the maximum dose allowed for a pacemaker during radiotherapy should be limited to 2 Gray (Gy). Pacemakers may experience functional changes at doses as low as 2 Gy and failure at doses around 10 Gy. During the treatment process, the following should be considered:

1. Identify the presence and type of pacemaker and consult with the cardiologist.

2. Estimate the dose to the pacemaker using the treatment planning system (TPS).

3. Minimize the dose to the pacemaker by avoiding direct irradiation when possible.

4. Ensure that the pacemaker is not in the direct beam path.

5. Monitor the patient for any symptoms related to pacemaker malfunction.

6. Limit the use of high-energy beams (e.g., photons >10 MV) to reduce the risk of neutron production.

7. Perform a follow-up check on the pacemaker, especially if the dose exceeds 2 Gy.

8. Consider continuous ECG monitoring during treatment, particularly for patients who are pacing-dependent.

The goal is to minimize the risk to the pacemaker while delivering the necessary cancer treatment. Regular monitoring and collaboration between the radiation oncologist, medical physicist, and cardiologist are essential.

Question14

According to AAPM TG reports, what are the limitations of MLC tracking?

Answer:

According to AAPM TG reports, particularly TG-264, the limitations of MLC tracking include:

1. Latency (System Delay):

There is a delay between detecting the target motion and moving the MLC leaves. This can cause geometric errors, especially with fast or irregular motion.

2. Accuracy of Tracking System:

The effectiveness of MLC tracking depends on the accuracy of the motion detection system. Errors in target localization directly affect MLC positioning.

3. Mechanical Limitations:

MLC leaves have speed, acceleration, and gap limitations. Rapid target motion may exceed the mechanical capabilities of the MLC system, leading to issues like underdosing or overdosing.

4. Geometric Uncertainty:

Even with accurate tracking, there is residual uncertainty in the alignment between the moving target and MLC aperture, which may impact dose delivery.

5. Motion Prediction Inaccuracy:

Some systems rely on motion prediction algorithms, which can fail if the target motion becomes irregular, for example, due to coughing or shallow breathing.

6. System Integration Challenges:

MLC tracking requires seamless integration between motion detection systems and the treatment machine, which can be complex and prone to failure.

7. Increased QA Complexity:

Quality assurance for MLC tracking systems is more complex and time-consuming compared to static treatments, requiring verification of latency, accuracy, and motion response.

8. Clinical Availability:

Not all linear accelerators and software platforms support real-time MLC tracking, and its integration into commercial systems is still under development.

9. Dosimetric Errors for Organs-at-Risk (OARs):

MLC tracking typically focuses on a single point (the tumor centroid) and may not account for motion of surrounding organs at risk, leading to dosimetric errors.

10. Multi-Target Tracking:

Tracking multiple targets simultaneously can complicate the system, reducing its accuracy and affecting the dose delivered to the targets.

11. Plan Complexity:

Complex treatment plans or intricate target motion patterns may reduce the effectiveness of MLC tracking, leading to dose artifacts or inaccuracies.

These limitations underscore the need for careful system integration, accurate motion detection, and thorough quality assurance to ensure safe and effective use of MLC tracking in radiotherapy.

Question15

According to AAPM TG reports, for deep-inspiration breath hold (DIBH) for breast radiotherapy treatment with bolus, what should we pay attention to when performing surface guided radiotherapy?

Answer:

When performing surface-guided radiotherapy (SGRT) for deep-inspiration breath hold (DIBH) in breast radiotherapy treatment with bolus, it is important to consider several factors to ensure accurate and safe treatment delivery, as outlined in AAPM Task Group reports:

1. Bolus placement must be consistent across treatments, as any variations in its position can affect the surface geometry and tracking accuracy.

2. Use materials that are compatible with SGRT systems (e.g., optically matte and non-slipping boluses). The bolus should fit snugly against the skin to minimize air gaps that could alter the dose delivery.

3. Bolus can deform the breast tissue and alter the external surface, so it’s important to monitor how well the SGRT system tracks with the bolus in place. Ensure that the bolus doesn’t obstruct the surface points needed for accurate tracking.

4. Adjust the region of interest (ROI) in SGRT systems to ensure alignment despite the surface changes caused by the bolus.

5. Patients may experience discomfort with bolus in place, affecting their ability to hold their breath consistently. Monitor breath-hold duration and stability to ensure treatment accuracy.

6. Establish appropriate gating thresholds based on observed surface motion during DIBH. This ensures that radiation is only delivered during optimal breath-hold periods.

7. Ensure that the bolus is tissue-equivalent and doesn’t create significant artifacts that could affect the dose distribution.

8. Minimize air gaps between the bolus and the skin to prevent dosimetric errors, as any gaps could reduce the skin dose enhancement intended by the bolus.

9. Perform QA with the bolus in place to verify that the SGRT system tracks correctly and the treatment is reproducible.

10. Daily reference surface captures should be performed to ensure accuracy, especially when bolus is used.

11. Ensure that the patient can comfortably hold their breath during treatment. Provide a safety mechanism, like a button to stop treatment if the patient experiences difficulty.

By addressing these factors, SGRT with DIBH and bolus can be safely and effectively implemented, providing optimal dose delivery to the target while minimizing dose to critical structures such as the heart.

Question16

According to AAPM TG reports, in a monthly quality assurance test, what is the tolerance of laser marking accuracy of an MRI simulator?

Answer:

According to AAPM Task Group 284, the monthly quality assurance tolerance for laser marking accuracy on an MRI simulator is ±2 mm. This tolerance applies in the axial, sagittal, and coronal directions to ensure that the lasers used for patient positioning are aligned accurately with the MRI scanner's imaging center. The accuracy is typically tested using a phantom with internal markers. The phantom is aligned using the lasers, an MRI is taken, and the difference between the laser-defined position and the image center is measured. Although ±2 mm is the standard tolerance, systems with high-precision laser localizers may achieve tighter tolerances, such as ±1 mm, which may be necessary for applications like stereotactic radiosurgery.

Question17

According to AAPM TG reports, what are disadvantages of using a constant RBE in proton therapy?

Answer:

According to AAPM Task Group 256, there are several disadvantages to using a constant relative biological effectiveness (RBE), typically 1.1, in proton therapy:

1. Oversimplification of biological effects: RBE is not actually constant. It varies based on factors like linear energy transfer (LET), tissue type, dose per fraction, and the specific biological endpoint (e.g., cell death, mutation). Using a fixed value ignores these variations.

2. Risk of underdosing the tumor: If the actual RBE in the tumor region is higher than 1.1, assuming a constant RBE could lead to delivering less biological dose than intended, which might reduce tumor control.

3. Risk of overdosing normal tissues: LET increases at the end of the proton range (the Bragg peak), which can result in a higher actual RBE. Using a constant RBE can underestimate the biological effect in surrounding healthy tissues, increasing the risk of toxicity.

4. Suboptimal treatment plans: Constant RBE doesn’t allow treatment planning systems to optimize based on LET or variable biological effectiveness. This limits the ability to spare healthy tissues and target tumors more effectively.

5. Misleading clinical comparisons: Clinical trials that compare photon and proton therapy based on a fixed RBE might draw inaccurate conclusions if the actual RBE varies significantly.

6. Limited biological modeling: Current treatment planning systems using a constant RBE cannot incorporate more advanced biological optimization or take advantage of emerging knowledge about spatial and tissue-dependent variations in RBE.

In summary, while a constant RBE is convenient and practical, it introduces uncertainties in dose delivery and treatment planning. AAPM TG-256 acknowledges this and highlights the need for more advanced models that account for variable RBE in clinical practice.

Question18

According to AAPM TG reports, what is the difference between analytic calibration model and pose determination model for a C-arm CBCT system?

Answer:

According to AAPM TG-238, the analytic calibration model and the pose determination model are both used for geometric calibration of C-arm CBCT systems, but they differ in their assumptions and methods.

The analytic calibration model assumes that the system follows an idealized geometry, such as a circular or helical arc. It relies on a fixed set of geometric parameters, including source-to-detector distance, source-to-isocenter distance, and detector offsets. These parameters are often derived using a calibration phantom and mathematical optimization. This model works well when the mechanical motion of the system is stable and predictable. However, it may not capture real-world imperfections such as mechanical flex or sag.

The pose determination model, on the other hand, does not assume an ideal path. Instead, it calculates the actual pose—meaning the position and orientation—of the X-ray source and detector for each projection image. It uses a calibration phantom with known marker positions and derives the system's geometry from how these markers appear in the projection images. This model is more flexible and accurate, especially when the system’s motion is inconsistent or prone to mechanical variations. It can adapt to real-time changes and is useful for high-precision applications, though it is more computationally intensive.

Question19

According to AAPM TG reports, what should be paid attention to when using TLDs or OSLs in the measurement of skin dosimetry?

Answer:

According to AAPM TG-191, when using TLDs or OSLDs for skin dosimetry, several important considerations must be kept in mind due to the shallow depth (0.07 mm) of interest for skin dose:

1. Effective measurement depth: TLDs and OSLDs have physical thickness, so their effective point of measurement lies deeper than the actual skin surface. For example, dot OSLDs have a depth of around 0.8 mm, which can significantly overestimate the true surface dose.

2. Overestimation of skin dose: Because of the above point, both TLDs and OSLDs tend to overestimate surface dose by approximately 10-40%. This is due to the sharp dose gradient near the surface and the detector's depth-averaging effect.

3. Extrapolation technique: One method to address overestimation is to use detectors of varying thickness and extrapolate the dose to the true surface depth (0.07 mm), though even this method has limitations.

4. Contact with skin: Dosimeters must be placed in direct and firm contact with the skin. Any gap or misplacement can lead to inaccurate measurements due to changes in backscatter or attenuation.

5. Size and shape of dosimeters: Larger or rigid detectors may not conform well to curved surfaces, which can create air gaps and affect accuracy. Using smaller or flexible detectors is recommended for curved body parts.

6. Light sensitivity: OSLDs are sensitive to light. They must be protected from exposure before and after irradiation to prevent signal degradation.

7. Calibration: TLDs and OSLDs must be calibrated for the energy range used in the measurements. Calibration should also reflect the clinical setup as closely as possible.

8. Environmental factors: Both detector types are sensitive to storage and handling conditions like temperature and humidity, which can influence results.

In summary, when using TLDs or OSLDs for skin dosimetry, it is critical to account for the detector’s measurement depth, ensure proper placement, and consider the need for calibration and corrections due to overestimation at shallow depths.

Question20

According to AAPM TG reports, what is the tolerance of output factors for a monte-carlo based treatment planning system?

Answer:

According to AAPM TG-157, the tolerance for output factors in a Monte Carlo-based treatment planning system is typically within ±2% when comparing calculated values to measured data during commissioning or validation.

Monte Carlo (MC) methods simulate dose with high accuracy, but careful modeling and validation are essential. The following points are important:

1. Validation against measurements: Output factors for square fields (especially from 3 cm × 3 cm to 40 cm × 40 cm) should match measured values within ±2%. This helps ensure that the Monte Carlo model of the linear accelerator is accurate.

2. Small field dosimetry: For very small fields (e.g., in SRS/SBRT), higher uncertainties may exist, but the goal is still to stay within ±2% if possible. In difficult cases, up to ±3% may be acceptable, but this must be justified.

3. Output ratios: These are Monte Carlo-calculated values that mimic clinical measurements and can be compared directly with water phantom data. Agreement within ±2% across field sizes is a key acceptance criterion.

4. Off-axis fields and IMRT scenarios: Validation should also include small, off-axis fields and test cases designed to stress the algorithm, such as narrow junction fields, to ensure reliability in clinical treatment plans.

In short, for Monte Carlo-based TPSs, a ±2% tolerance is the benchmark for output factor agreement, according to TG-157.
