# Supplementary material for: RTPhy‐ChatBot: A RAG‐Based intelligent assistant for radiotherapy physics using LLaMA3 and AAPM reports
Source: J Appl Clin Med Phys. 2025 Oct 9;26(10):e70263. doi: 10.1002/acm2.70263 (PMC12509246; doi:10.1002/acm2.70263)
Supplement: Supplementary file 3 — Supporting information [file ACM2-26-e70263-s003.docx]

Appendix C

Chat history with all LLMs using zero-shot CoT

Questions1 (TG142)

Let’s think step by step. According to AAPM TG reports, in a monthly QA procedure, what is the tolerance of photon beam proﬁle constancy of an IMRT machine?

RTPhy-ChatBot:

According to the context provided, there are different tolerances for photon beam profile constancy depending on the specific application and machine type. However, in the context of a comprehensive QA program for IMRT, the tolerance for photon beam profile constancy is ±1%. This value is a deviation from baseline, not an absolute value. It's important to note that this tolerance may vary between institutions or even within different departments of the same institution, depending on their specific requirements and capabilities.

In addition, it's worth mentioning that there are other aspects of dosimetric and mechanical machine QA, as well as procedural QA, that should be considered in a monthly QA procedure for an IMRT machine. These include periodic QA baselines, site-specific IMRT plans and QA measurements on phantoms, and in-depth analysis of any discrepancies or failures to meet tolerance limits.

Furthermore, it's important to recognize that the tolerance limits for photon beam profile constancy are just one aspect of IMRT QA. Other metrics such as dose difference (DTA) and gamma passing rate should also be considered when evaluating the quality of IMRT delivery. The recommendations in the context provided aim to improve the IMRT QA process and establish consistent, comparable IMRT QA criteria among institutions.

Gemini-2.0-flash:

According to AAPM TG-142 report, the monthly tolerance for photon beam profile constancy of an IMRT machine is **1%**.

GPT-4o:

let’s walk through this **step by step** based on the AAPM **Task Group 142 (TG-142)** report, which provides the standard quality assurance (QA) guidelines for medical linear accelerators, including **IMRT** systems.

**🎯 What is Photon Beam Profile Constancy?**

- **Beam profile constancy** checks the **shape and symmetry** of the radiation field across the treatment area.
- It ensures that the **flatness and symmetry** of the beam remain consistent over time, especially important in **IMRT**, where dose modulation is sensitive to beam quality.

**📅 Monthly QA — TG-142 Requirements**

According to **AAPM TG-142**, the **tolerance for photon beam profile constancy** during **monthly QA** is:

**✅ 2% for both:**

- **Flatness**
- **Symmetry**

This applies to **IMRT-capable machines**, and is typically verified with:

- 1D or 2D ion chamber arrays
- Diode arrays
- Film or EPID-based profiles
- Measurements compared to baseline/reference profiles

Moonshot-v1:

According to AAPM TG reports, in a monthly QA procedure for an IMRT machine, the tolerance for photon beam profile constancy is 2%.

Question2 (TG179)

Let’s think step by step. According to AAPM TG reports, what are the advantages of kVCT compared to MVCT?

RTPhy-ChatBot:

The advantages of kVCT (kilovoltage computed tomography) compared to MVCT (megavoltage computed tomography) include:

1. Higher contrast resolution: kVCT produces images with higher contrast resolution, making it easier to distinguish between different tissues and structures in the body. This is particularly important for cancer treatment planning and imaging of small lesions or complex anatomies.
2. Lower patient dose: kVCT uses lower energy x-rays than MVCT, resulting in a lower patient dose. The dose from a single kVCT image session ranges from 0.01 to 0.05 Gy, while typical exposures during a single MVCT image acquisition range from 0.02 to 0.15 Gy (although lower doses are becoming more achievable).
3. Modeling in treatment planning systems: kVCT images can be modeled in treatment planning systems and the dose can therefore be accounted for in the treatment plan, while MVCT images cannot be directly modeled in treatment planning systems due to their different physical interaction probabilities with tissues.
4. Wider availability: kVCT is more widely available than MVCT, as it is used primarily for diagnostic purposes and is already installed in many hospitals and clinics. In contrast, MVCT is mainly used for image-guided radiation therapy (IGRT) purposes and requires specialized equipment and expertise to operate.

Ultimately, the choice between kVCT and MVCT depends on the specific clinical application and the trade-offs between image quality, patient dose, and real-time imaging capabilities.

Gemini-2.0-flash:

According to AAPM TG reports and current literature, kVCT (kilovoltage Computed Tomography) offers several advantages compared to MVCT (megavoltage Computed Tomography) for image-guided radiation therapy (IGRT):

- **Superior Image Quality and Contrast:** kVCT generally provides higher image contrast, especially for soft tissues. This is because kV photons interact with tissue primarily through photoelectric effect and Compton scattering, both of which are energy-dependent and result in better differentiation between tissues with slightly different densities. MV photons, on the other hand, interact predominantly via Compton scattering, which is less dependent on atomic number, leading to lower soft tissue contrast.
- **Enhanced Visualization of Anatomical Structures:** The improved image quality of kVCT allows for clearer visualization and delineation of both bony anatomy and soft tissues, including organs at risk (OARs) and the target volume. This is crucial for accurate patient setup and target localization before each treatment fraction.
- **Lower Dose to the Patient:** Typically, kVCT imaging delivers a lower radiation dose to the patient compared to MVCT scans that provide comparable image quality. This is an important consideration for minimizing the overall radiation exposure during the course of radiotherapy.
- **Faster Acquisition Times:** kVCT scans are generally faster to acquire than MVCT scans. This reduces the time the patient needs to be on the treatment couch, improving patient comfort and potentially increasing clinic efficiency.
- **Improved CT Number Accuracy and Stability:** kVCT systems tend to have more stable and accurate CT numbers, which can be beneficial if the images are used for dose recalculation in adaptive radiation therapy (ART). While MVCT CT numbers are related to electron density, kVCT provides a more direct relationship to physical density, which can be advantageous for certain dose calculation algorithms.
- **Reduced Metal Artifacts in Some Cases:** While metal artifacts can be an issue in both kVCT and MVCT, in some scenarios, MVCT might be preferred for reducing artifacts from high-density materials like metallic implants. However, advancements in kVCT reconstruction algorithms are continuously improving metal artifact reduction.

**General Information:**

- **kVCT Systems:** These systems typically use an X-ray tube operating in the kilovoltage range (e.g., 80-140 kVp) and a flat-panel detector. They are often integrated into modern linear accelerators to provide onboard imaging capabilities. Examples include cone-beam kVCT (CBCT) systems.
- **MVCT Systems:** These systems utilize the megavoltage treatment beam of the linear accelerator and the electronic portal imaging device (EPID) to acquire CT images. While they can provide volumetric imaging for IGRT, their soft tissue contrast is generally lower than kVCT.

The choice between kVCT and MVCT often depends on the specific clinical application, the anatomical site being imaged, and the available technology. However, the superior soft tissue contrast and lower dose of kVCT have made it the preferred modality for many IGRT applications where accurate visualization of both bony and soft tissue structures is critical.

GPT-4o:

Let's break this down **step by step** based on insights from **AAPM Task Group reports** like **TG-104**, **TG-180**, **TG-148**, and others that address **imaging in radiotherapy**.

**🧩 kVCT vs. MVCT in Radiotherapy Imaging**

**📘 Definitions:**

- **kVCT (kilovoltage CT)**: Acquired with kilovoltage X-rays (typically 80–140 kVp), used in planning and IGRT.
- **MVCT (megavoltage CT)**: Acquired with megavoltage X-rays (∼3–6 MV), often used in systems like TomoTherapy for daily IGRT.

**✅ Advantages of kVCT Over MVCT (According to AAPM TG Reports)**

**1. Superior Image Quality (Contrast Resolution)**

- **AAPM TG-104** and **TG-148** state:
  - **kVCT** offers **better soft tissue contrast** than MVCT.
  - Especially important for prostate, head & neck, or abdominal tumors.

**Why?**

- kV photons interact more via the **photoelectric effect**, which is more sensitive to atomic number differences.
- MV photons rely on **Compton scattering**, which doesn’t differentiate tissue types as well.

**2. Lower Imaging Dose**

- **TG-180** emphasizes that:
  - **kVCT typically delivers lower dose** than MVCT for equivalent image quality.
  - Important for daily imaging and pediatric patients.

**3. Higher Spatial Resolution**

- kV systems have **finer detector elements**, resulting in better **spatial resolution** (important for bony anatomy, fiducials).
- **TG-180** supports the clinical use of kVCT for high-precision alignment due to its clarity.

**4. Better HU (Hounsfield Unit) Accuracy and Consistency**

- HU values from kVCT scans are more consistent with **treatment planning systems**, leading to:
  - More accurate **electron density mapping**
  - Better dose calculation
- MVCT tends to have **compressed HU ranges**, making tissue differentiation and density conversion harder.

**5. Faster Scanning**

- Many modern kVCT systems can scan **more quickly** than MVCT (especially with multi-slice or helical systems).
- This reduces motion artifacts.

Moonshot-v1:

According to AAPM TG reports and related studies, the advantages of kVCT (kilovoltage computed tomography) compared to MVCT (megavoltage computed tomography) in radiotherapy include:

**1. Improved Image Quality**

- **Higher Contrast Resolution**: kVCT generally provides better contrast resolution compared to MVCT. This is due to the lower energy of the kV beam, which results in higher contrast and better visualization of soft tissues.
- **Reduced Noise Levels**: kVCT systems typically produce images with lower noise levels compared to MVCT. For example, the ClearRT helical kVCT system demonstrated noise levels similar to a Siemens Edge kVCT, while MVCT systems had noise levels 1.5–5 times higher.

**2. Better Motion Capture**

- **Full Excursion Capture**: kVCT can more accurately capture the full excursion of a target during respiratory motion due to its conical acquisition of projections over multiple respiratory cycles. This is particularly useful in treatments like lung stereotactic body radiation therapy (SBRT) where motion management is critical.
- **Reduced Motion Underestimation**: While both kVCT and MVCT can underestimate target motion, kVCT has the potential to provide more accurate soft-tissue registration when window and level settings are optimized.

**3. Reduced Artifacts**

- **Lower Artifact Levels**: kVCT systems generally produce fewer artifacts compared to MVCT. For example, MVCT often exhibits significant “capping” artifacts, whereas kVCT systems like ClearRT show reduced artifacts and better overall image uniformity.

**4. Faster Scan Times**

- **Reduced Scan Duration**: kVCT systems can achieve faster scan times compared to MVCT. For example, ClearRT kVCT scans can be up to 4–8 times faster than MVCT scans, depending on the acquisition parameters. This can improve workflow efficiency and patient comfort.

**5. Enhanced Clinical Utility**

- **Superior Image Guidance**: The improved image quality and reduced noise of kVCT make it more suitable for daily image guidance and adaptive radiotherapy (ART) compared to MVCT. This can enhance the accuracy of patient setup and treatment delivery.
- **Better Soft-Tissue Contrast**: kVCT provides better soft-tissue contrast, which is crucial for delineating anatomical structures and improving treatment planning.

**Conclusion**

kVCT offers significant advantages over MVCT in terms of image quality, motion capture, reduced artifacts, faster scan times, and enhanced clinical utility. These benefits make kVCT a preferred choice for image-guided radiotherapy (IGRT) and adaptive radiotherapy (ART) applications.

Question3 (TG179)

Let’s think step by step. According to AAPM TG reports, what are the low contrast resolution standards for kV-CBCT, fan beam MVCT, and MV-CBCT, respectively?

RTPhy-ChatBot:

he low contrast resolution requirements for CT-based IGRT systems are generally looser than those for diagnostic CT scanners due to the primary use of IGRT being for localization of preidentified structures rather than diagnosing diseases.

For kV-CBCT systems, visibility of 1% contrast objects that are 7 mm in diameter has been reported. Fan-beam MVCT can resolve 13 mm diameter objects with 2% density differences from the background, and MV-CBCT can resolve 2 cm objects with 1% contrast.

It is important to note that these values depend on various factors such as phantom size, object size, reconstructed voxel size, and imaging technique. Therefore, it is crucial to obtain test images using clinically relevant parameters and keep them constant for quality control checks.

Regarding the specific standards for each technology:

- For kV-CBCT, there isn't a widely accepted standard for low contrast resolution. However, the visibility of 1% contrast objects with a diameter of 7 mm is often used as a benchmark.
- For fan-beam MVCT, the ability to detect 13 mm diameter objects with 2% density differences from the background is considered acceptable.
- For MV-CBCT, there isn't a widely accepted standard for low contrast resolution either. However, some studies have reported that it can resolve 2 cm objects with 1% contrast.

It is essential to remember that these values are approximate and may vary depending on the specific system and imaging conditions. Regular testing and calibration procedures should be performed to ensure accurate and reliable low contrast resolution for each CT-based IGRT system.

Gemin-2.0-flash:

It is important to note that specific quantitative low contrast resolution standards for kV-CBCT, fan beam MVCT, and MV-CBCT are **not explicitly defined with numerical tolerances in the main AAPM TG reports like TG-142 or TG-179 in the same way as some other QA parameters.**

Instead, the AAPM TG reports emphasize the establishment of **baseline image quality** during commissioning and recommend that routine QA should ensure that the image quality remains consistent with this baseline. Any significant deviation from the baseline should trigger investigation and corrective action.

Here's a breakdown of how low contrast resolution is typically addressed in the context of these imaging modalities according to AAPM TG reports:

**1. kV-CBCT (Kilovoltage Cone-Beam CT):**

- AAPM TG-179, which specifically addresses QA for CT-based IGRT, recommends **qualitative assessment** of low contrast detectability as part of routine QA.
- It suggests using phantoms with low contrast inserts (e.g., CatPhan phantom with its low contrast section) and visually inspecting the images to ensure that the low contrast objects are still discernible.
- The report emphasizes establishing a baseline level of low contrast resolution during commissioning and maintaining that level over time. There isn't a specific numerical tolerance provided for the number of discernible low contrast objects.

**2. Fan Beam MVCT (Megavoltage Computed Tomography with Fan Beam):**

- AAPM TG-179 also addresses fan beam MVCT. Due to the inherent lower soft tissue contrast of MVCT compared to kVCT, the focus on low contrast resolution is often less emphasized.
- The report still recommends **qualitative assessment** using appropriate phantoms to ensure that the low contrast capabilities of the system are not significantly degraded over time.
- Again, the emphasis is on maintaining the baseline performance established during commissioning. Specific numerical tolerances for low contrast resolution are not typically provided.

**3. MV-CBCT (Megavoltage Cone-Beam CT):**

- Similar to fan beam MVCT, MV-CBCT generally has lower soft tissue contrast compared to kV-CBCT.
- AAPM TG-179 recommends **qualitative evaluation** of image quality, including low contrast aspects, using appropriate phantoms.
- The goal is to ensure the consistency of image quality relative to the baseline established during commissioning. No specific numerical tolerances for low contrast resolution are usually defined.

**In summary:**

AAPM TG reports for IGRT utilizing CT-based technologies (like TG-142 and TG-179) primarily recommend a **baseline and consistency approach** for low contrast resolution in kV-CBCT, fan beam MVCT, and MV-CBCT. This involves:

- **Establishing a baseline:** Assessing and documenting the low contrast resolution capabilities of the system during commissioning using appropriate phantoms.
- **Routine qualitative assessment:** Regularly evaluating low contrast detectability using the same phantoms and ensuring that the image quality remains consistent with the established baseline through visual inspection.

**There are no universally defined numerical tolerances (e.g., being able to visualize a certain percentage contrast difference for a specific object size) for low contrast resolution in these modalities specified in the main AAPM TG reports.** The acceptable level of low contrast resolution is often clinically determined based on the intended use of the images for patient setup and target localization.

GPT-4o:

let’s go step by step based on **AAPM TG reports** such as **TG-142**, **TG-179**, **TG-180**, and **TG-148**, which address **image quality metrics**, including **low contrast resolution (LCR)** for IGRT systems.

**🧩 What is Low Contrast Resolution (LCR)?**

**Low contrast resolution** is the ability of an imaging system to detect small differences in density between objects and their background. It's critical in identifying soft tissues or subtle anatomical changes — especially important for **daily image guidance** in radiotherapy.

**📘 According to AAPM TG Reports — Standards for LCR:**

**1. ✅ kV-CBCT (kilovoltage cone-beam CT)**

**From AAPM TG-142** and **TG-179**:

- **Standard**:
  Ability to distinguish **≤4 mm objects** with **low contrast difference** (typically ~1% or less).
- **Expected performance**:
  - Best among the three modalities
  - High soft tissue visibility
  - Typical use cases: **prostate**, **H&N**, **lung**, **liver**, **pelvis**

**2. ⚠️ Fan Beam MVCT (Tomotherapy)**

**From AAPM TG-148**:

- **Standard**:
  Ability to distinguish **≥6–8 mm** low contrast objects
  - LCR is limited compared to kV-CBCT
  - Contrast difference required is usually **≥2–3%**
- **Use case**: Primarily used for **bony alignment**, and **less for soft tissue** unless fiducials are present

**3. ❗ MV-CBCT (megavoltage cone-beam CT)**

**From AAPM TG-180**:

- **Standard**:
  Very limited LCR; typically can only distinguish **objects ≥8–10 mm** with contrast differences ≥3–5%
- **Reason**:
  - Lower contrast-to-noise ratio due to Compton scatter dominance at MV energies
  - Often used when **metal artifacts** are present or in systems without kV imaging

Moonshot-v1:

According to AAPM TG reports and related studies, the low contrast resolution standards for different imaging modalities are as follows:

**1. kV-CBCT (kV Cone-Beam CT)**

- **Standard**: The low contrast resolution for kV-CBCT should be able to detect objects with a contrast level of **0.5%**. For example, using the Catphan phantom, the smallest detectable disc diameter at this contrast level should be around **7 mm**.

**2. Fan Beam MVCT (Megavoltage Computed Tomography)**

- **Standard**: Fan beam MVCT systems typically have better low contrast resolution compared to CBCT. They can detect objects with contrast levels as low as **0.3%**. This means that smaller and lower contrast objects can be more easily visualized in fan beam MVCT images.

**3. MV-CBCT (Megavoltage Cone-Beam CT)**

- **Standard**: Similar to kV-CBCT, the low contrast resolution for MV-CBCT should also be able to detect objects with a contrast level of **0.5%**. However, the exact detectability can vary depending on the specific system and imaging conditions.

**Summary**

- **kV-CBCT**: Detect objects with a contrast level of **0.5%**.
- **Fan Beam MVCT**: Detect objects with a contrast level of **0.3%**.
- **MV-CBCT**: Detect objects with a contrast level of **0.5%**.

These standards help ensure that the imaging systems are capable of detecting clinically relevant low contrast objects, which is crucial for accurate treatment planning and delivery in radiotherapy.

Question4 (TG290):

Let’s think step by step. According to AAPM TG reports, what is the difference between PS, PBS, and US in particle radiotherapy?

RTPhy-ChatBot:

In particle radiotherapy, there are three primary delivery techniques used: Passive Scattering (PS), Uniform Scanning (US), and Pencil Beam Scanning (PBS). Let's discuss each technique step by step:

1. Passive Scattering (PS): PS is a method where the proton beam is scattered using a range shifter or degrader, which increases the angular dispersion and emittance of the beam. The beam is then collimated to create a uniform dose distribution across the target volume. This technique uses a large amount of scattering material, leading to increased neutron production and reduced particle efficiency. However, it offers some advantages such as better range accuracy and improved conformity for larger targets.
2. Uniform Scanning (US): US is another delivery method where the proton beam is scanned uniformly across the target volume using compensators and apertures. This technique uses a smaller amount of scattering material compared to PS, reducing neutron production and improving particle efficiency. However, it requires more time for delivery due to the layer-by-layer approach. US is commonly used with static range modulators or in conjunction with energy stacking techniques.
3. Pencil Beam Scanning (PBS): PBS is a modern delivery technique where the proton beam is scanned pencil by pencil across the target volume using magnetic deflection. This technique offers superior range accuracy and conformity, making it ideal for treating small targets with high precision. However, it requires more sophisticated equipment and longer delivery times compared to PS and US. PBS also introduces the capability to vary the proton dose across any given field, making the concept of a consistent SOBP irrelevant for PBS delivery QA. Passive motion-management techniques can be applied regardless of the delivery mode (DS, US, PBS) and include adequate beam angle selection, motion encompassing techniques using 4DCT, and target CT number overrides for range compensator design and smearing. Active motion-management techniques are used to reduce the effective range of motion and can be used with passive motion-management techniques. The choice of a delivery technique depends on various factors such as tumor size, shape, location, and patient motion. For instance, PS is commonly used for ocular or "eye" lines due to its better range accuracy and improved conformity for larger targets. US is often used when target volumes are large and complex, while PBS is preferred for small, well-defined targets with high precision requirements. In summary, the main differences between PS, PBS, and US in particle radiotherapy lie in their delivery techniques, advantages, disadvantages, and applications. Understanding these differences can help clinicians choose the most appropriate technique for a given patient's treatment needs.

Gemini-2.0-flash:

While AAPM Task Group (TG) reports may not dedicate a specific report solely to defining the differences between PS, PBS, and US in particle radiotherapy, these terms are discussed within reports concerning the clinical implementation and quality assurance of particle therapy. Based on the general understanding within the field and as described in relevant literature and potentially mentioned within sections of TG reports (like those focusing on delivery techniques in proton therapy), here's a breakdown of the differences:

**1. PS - Passive Scattering:**

- **Technique:** This is an older technique for beam delivery in particle therapy. It uses physical beam-shaping devices placed in the beam path to create a broad, uniform radiation field that conforms laterally to the target volume.
- **Components:** These devices typically include:
  - **Scatters:** Thin foils (usually made of materials like lead or tantalum) that broaden the initially narrow particle beam. Often, dual scattering systems are used to achieve a more uniform field.
  - **Range Modulators (Spread-Out Bragg Peak - SOBP):** Rotating or stepped devices with varying thicknesses of material that broaden the sharp Bragg peak in depth to cover the target volume longitudinally.
  - **Apertures (Collimators):** Custom-made or multi-leaf collimators (MLCs) made of dense materials that shape the lateral extent of the beam to match the projected shape of the tumor at a specific depth.
  - **Compensators:** Patient-specific devices made of varying thicknesses of a tissue-equivalent material placed downstream of the aperture to account for patient surface irregularities and ensure the beam stops at the distal edge of the tumor.
- **Characteristics:**
  - Creates a relatively uniform dose distribution across the target volume.
  - Simpler in terms of delivery control compared to scanning techniques.
  - Can result in higher integral dose to healthy tissues due to the scatter and the need for a broader initial beam.
  - Less conformal to complex target shapes and concave volumes.
  - Limited ability for intensity modulation.

**2. PBS - Pencil Beam Scanning (also known as Spot Scanning):**

- **Technique:** This is an active beam delivery technique where a narrow pencil beam of particles (typically a few millimeters in diameter) is magnetically steered to sequentially "paint" the target volume in three dimensions.
- **Components:**
  - **Magnetic Scanning System:** Rapidly deflects the pencil beam in the lateral (X and Y) directions.
  - **Energy Modulation System:** Varies the energy of the beam pulse-by-pulse to control the penetration depth (Z direction), effectively creating the SOBP electronically.
  - **Nozzle:** Contains beam monitoring devices but lacks the bulky scattering foils, range modulators, and fixed apertures of passive scattering. Some nozzles may include dynamic multi-leaf collimators (DMLCs) for further beam shaping or motion management.
- **Characteristics:**
  - Highly conformal dose distributions that can precisely match complex tumor shapes.
  - Lower integral dose to healthy tissues compared to passive scattering due to reduced scatter and the ability to shape the beam more tightly around the target.
  - Enables intensity-modulated particle therapy (IMPT) by varying the number of particles delivered to each spot, allowing for optimization of the dose distribution to spare critical structures.
  - More susceptible to motion artifacts due to the sequential delivery of spots (interplay effect). Motion management strategies are crucial.
  - Requires sophisticated treatment planning and delivery systems.

**3. US - Uniform Scanning (a less commonly used term, often considered a subset or early form of PBS):**

- **Technique:** This term is less consistently defined and used in the literature compared to PS and PBS. It generally refers to a scanning technique where the pencil beam is scanned across the target volume in a raster pattern with the goal of creating a relatively uniform dose distribution within each slice of the target.
- **Relationship to PBS:** Uniform scanning can be seen as an earlier or simpler form of pencil beam scanning. While PBS often implies the ability to modulate the intensity of each spot for IMPT, uniform scanning might focus more on achieving a homogeneous dose within the target volume through the overlapping of scanned spots without explicit intensity optimization.
- **Characteristics:**
  - Offers some level of conformality compared to passive scattering.
  - May have a lower integral dose than passive scattering.
  - Might have limited or no intensity modulation capabilities compared to advanced PBS techniques.
  - Still susceptible to motion artifacts.

**In summary, according to the general understanding in particle therapy (which would be reflected in the context of relevant AAPM TG reports):**

- **Passive Scattering (PS)** uses physical devices to create a broad, uniform beam. It's simpler but less conformal and delivers a higher integral dose.
- **Pencil Beam Scanning (PBS)** uses magnetic steering and energy modulation to precisely paint the target volume with a narrow beam, allowing for highly conformal and intensity-modulated treatments with lower integral dose.
- **Uniform Scanning (US)** is a less common term that generally describes a scanning technique aimed at creating a uniform dose within the target, potentially representing an earlier or simpler form of PBS with limited or no intensity modulation.

For specific details and recommendations from AAPM TG reports, it would be necessary to consult reports that discuss particle therapy delivery techniques, such as those focusing on clinical implementation, commissioning, and quality assurance of proton or carbon ion therapy. These reports would provide the AAPM's perspective on the characteristics and clinical considerations for each of these delivery methods.

GPT-4o:

Let's think this through **step by step** using terminology and standards from **AAPM Task Group reports**, especially those related to **proton and heavy ion therapy**, like **AAPM TG-185**, **TG-202**, and **TG-290**.

**✅ 1. PS – Passive Scattering**

**🌟 How It Works:**

- **The proton beam is broadened laterally using scattering foils, and range modulators (like rotating wheels) spread the dose in depth to cover the entire target.**
- **Apertures and compensators are used to shape the beam.**

**🔍 Characteristics:**

- **Simple and robust**
- **Fixed field shapes**
- **Uniform dose in target, but less conformal**
- **More neutron production due to beamline components**

**🧪 QA Notes (TG-185):**

- **QA of field-shaping hardware (apertures, compensators) is critical**
- **Less sensitive to motion than PBS**

**✅ 2. PBS – Pencil Beam Scanning**

**🌟 How It Works:**

- **A narrow “pencil” beam is magnetically scanned across the tumor, spot by spot and layer by layer.**
- **No need for physical apertures or compensators.**

**🔍 Characteristics:**

- **Highly conformal and intensity-modulated (IMPT)**
- **Allows dose painting**
- **Less material in beam path → lower neutron dose**
- **Very sensitive to motion (especially breathing)**

**🧪 QA Notes (TG-185, TG-290):**

- **Requires complex dose and position verification**
- **Motion mitigation (e.g., gating, repainting) is critical**

**✅ 3. US – Uniform Scanning**

**🌟 How It Works:**

- **Combines a scanning magnet (for lateral spreading) with a range modulator (for depth).**
- **Delivers a broad beam like PS, but using scanning rather than passive scattering.**

**🔍 Characteristics:**

- **Hybrid between PS and PBS**
- **Better dose conformity than PS**
- **Still uses apertures/compensators in some systems**
- **Less flexible than PBS**

**🧪 QA Notes (TG-202):**

- **QA includes both scanning system checks and beam shaping**

Moonshot-v1:

According to AAPM TG reports and related studies, the differences between Passive Scattering (PS), Pencil Beam Scanning (PBS), and Uniform Scanning (US) in particle radiotherapy are as follows:

**1. Beam Delivery and Dose Distribution**

- **PS (Passive Scattering)**:
  - **Beam Delivery**: Uses scatterers and range compensators to shape the beam. The beam is broadened laterally and modulated in depth using physical apertures and range compensators.
  - **Dose Distribution**: Produces a relatively homogeneous dose distribution in the target volume. It is less sensitive to motion due to its continuous delivery method.
- **PBS (Pencil Beam Scanning)**:
  - **Beam Delivery**: Uses scanning magnets to direct narrow pencil beams of particles to specific locations within the target volume. The beam is turned off between spots.
  - **Dose Distribution**: Can deliver highly conformal dose distributions, allowing for intensity-modulated proton therapy (IMPT). This method can reduce dose to organs at risk (OARs) but is more sensitive to motion due to its discrete spot delivery.
- **US (Uniform Scanning)**:
  - **Beam Delivery**: Similar to PBS but uses continuous scanning of the beam at each energy level. The beam remains on during scanning, and energy switching occurs between different layers.
  - **Dose Distribution**: Provides a lateral dose profile similar to PS but can have different delivery time structures depending on the implementation.

**2. Sensitivity to Motion**

- **PS**: Generally considered the least sensitive to motion due to its continuous delivery method.
- **PBS**: Most sensitive to motion because of its discrete spot delivery and the potential for interplay effects between beam delivery and patient motion.
- **US**: Sensitivity to motion is intermediate between PS and PBS, depending on the specific implementation and energy-switching times.

**3. Clinical Applications and Considerations**

- **PS**: Suitable for treatments where motion is less of a concern. It is often used in fixed-beam or gantry-based systems.
- **PBS**: Ideal for complex target geometries and cases where sparing of OARs is critical. It is more commonly used in gantry-based systems with advanced motion management techniques.
- **US**: Offers a balance between dose conformity and motion sensitivity. It can be used in both fixed-beam and gantry-based systems.

**4. Optimization and Motion Management**

- **PS**: Motion management focuses on reducing the magnitude of motion and ensuring accurate range modulation.
- **PBS**: Advanced optimization techniques, such as robust optimization and 4D optimization, are often used to account for motion uncertainties and improve dose conformity.
- **US**: Motion management strategies are similar to PS, but additional considerations may be needed for energy-switching techniques.

In summary, PS, PBS, and US each have distinct characteristics in terms of beam delivery, dose distribution, and sensitivity to motion. The choice of delivery technique depends on the specific clinical requirements, target geometry, and motion management needs.

Question5 (TG76):

Let’s think step by step. According to AAPM TG reports, introduce the respiratory movement management methods in photon radiotherapy.

RTPhy-ChatBot:

In photon radiotherapy, respiratory motion management methods are used to minimize the impact of respiratory motion on treatment accuracy and ensure that the radiation is delivered to the target while minimizing exposure to healthy tissues. The following are the common respiratory motion management methods in photon radiotherapy:

1. Motion-encompassing methods: These methods aim to account for respiratory motion by designing the treatment plan to include the motion range. This approach is often used when real-time motion tracking or gating is not available. The planning target volume (PTV) is expanded to account for the expected respiratory motion, typically in the order of 1-2 cm depending on the tumor site and patient's respiratory pattern.
2. Respiratory gated techniques: In these methods, the radiation delivery is synchronized with the patient's breathing cycle using various techniques such as active or passive gating. Active gating uses the patient's breath hold or abdominal compression to synchronize the beam delivery with a specific phase of the respiratory cycle. Passive gating uses external sensors to detect the patient's respiratory motion and adjust the beam delivery accordingly.
3. Breath-hold techniques: In these methods, the patient is instructed to hold their breath during treatment delivery. The duration of the breath hold depends on the specific technique used and the tumor site. For example, in shallow breathing techniques, the patient takes a deep breath and holds it for a few seconds before exhaling slowly. In deep inspiration breath-hold techniques, the patient takes a deep breath and holds it for several minutes.
4. Forced shallow-breathing methods: These methods involve instructing the patient to take shallow breaths during treatment delivery. This approach can be useful when the tumor is located near the diaphragm or when the patient has limited lung capacity. The radiation delivery is synchronized with the patient's breathing cycle using various techniques such as active or passive gating.
5. Respiration-synchronized techniques: In these methods, the radiation delivery is adjusted in real-time based on the patient's respiratory motion. This approach can be useful when the tumor motion is significant and cannot be accounted for by other methods. For example, in some cases, the beam delivery may be paused during periods of large respiratory motion and resumed during quieter phases.

It is important to note that not all patients require respiratory motion management, and the choice of method depends on various factors such as tumor site, patient's respiratory pattern, available technology, and clinical goals. The benefits and risks of each method should be carefully considered before implementation. Additionally, imaging methods used for respiratory motion management may involve additional ionizing radiation, which should be weighed against the potential benefits.

Gemini-2.0-flash:

According to AAPM Task Group (TG) reports, particularly TG-76 ("Management of Respiratory Motion in Radiation Oncology") and discussions in other relevant reports (like those on stereotactic body radiation therapy - SBRT), several methods are employed to manage respiratory motion in photon radiotherapy. The goal of these methods is to minimize the impact of breathing on the accuracy of radiation delivery to moving tumors in the thorax and upper abdomen. These methods can be broadly categorized as:

**1. Reduction of Motion Amplitude:**

- **Abdominal Compression:** This technique involves applying pressure to the patient's abdomen using a device (e.g., a rigid plate or inflatable bladder). This restricts the diaphragm's movement, thereby reducing the amplitude of respiratory motion of the tumor.
  - **Advantages:** Relatively simple to implement.
  - **Disadvantages:** Can be uncomfortable for the patient, may alter tumor position or shape, and may not be suitable for all patients or tumor locations. Requires careful monitoring of patient tolerance and reproducibility.

**2. Gating Techniques:**

- **Respiratory Gating (Breath-Hold Techniques):** Radiation delivery is synchronized with the patient's breathing cycle. The beam is only turned "on" during a specific, stable phase of respiration (e.g., end-inspiration or end-expiration) and turned "off" during other phases.
  - **Prospective Gating:** The system predicts the desired respiratory phase and starts irradiation when it is reached.
  - **Retrospective Gating:** The patient's breathing is tracked throughout the treatment, and the delivered dose is correlated with the respiratory phase. Only the dose delivered during the target phase is considered effective.
  - **Active Breathing Control (ABC):** A specific type of breath-hold technique where the patient is coached to hold their breath at a consistent lung volume using a mouthpiece connected to a spirometer. The beam is only activated when the breath-hold is within a predefined volume range.
  - **Advantages:** Can significantly reduce motion blurring and improve targeting accuracy.
  - **Disadvantages:** Increases treatment time due to beam-on duty cycle limitations. Requires patient cooperation and training. May not be suitable for patients who cannot reliably hold their breath or maintain a consistent breathing pattern.

**3. Tracking Techniques:**

- **Real-time Tumor Tracking:** The position of the tumor (or surrogate markers correlated with tumor motion) is continuously monitored in real-time during treatment delivery. The radiation beam is dynamically adjusted (e.g., by moving the treatment couch or using a robotic arm) to follow the tumor's movement.
  - **Marker-based Tracking:** Implanted fiducial markers near or within the tumor are tracked using real-time imaging (e.g., fluoroscopy, optical tracking of external markers correlated to internal motion).
  - **Markerless Tracking:** Direct tracking of the tumor using real-time imaging (e.g., cone-beam CT, kV imaging) without the need for implanted markers. This is more challenging due to image quality and processing speed.
  - **Advantages:** Can potentially deliver highly conformal doses to moving targets without significantly increasing treatment time.
  - **Disadvantages:** Requires sophisticated and integrated imaging and delivery systems. Marker-based tracking involves an invasive procedure for marker implantation. Markerless tracking is technically demanding. Correlation between external surrogates and internal tumor motion may vary.

**4. Motion Encompassing Techniques:**

- **Internal Target Volume (ITV) Planning:** Multiple CT scans are acquired at different phases of the respiratory cycle (e.g., using 4D-CT). The ITV is then defined as the union of the gross tumor volume (GTV) across all respiratory phases. Treatment plans are designed to cover the entire ITV.
  - **Advantages:** Relatively straightforward to implement as it relies on pre-treatment imaging. Does not require real-time monitoring or beam gating/tracking during delivery.
  - **Disadvantages:** Can lead to irradiation of a larger volume of healthy tissue compared to gating or tracking, as the ITV encompasses the entire range of motion. May not be optimal for highly mobile tumors or tumors near critical structures.
- **Enlarged Planning Target Volume (PTV) Margins:** A larger margin is added to the clinical target volume (CTV) to account for respiratory motion. This margin is determined based on the observed range of motion from imaging (e.g., 4D-CT).
  - **Advantages:** Simplest method to implement as it only requires adjustments in treatment planning.
  - **Disadvantages:** Similar to ITV planning, it can result in a larger volume of healthy tissue being irradiated, potentially increasing the risk of side effects. Does not account for intra-fractional variations in motion.

**Key Considerations Highlighted in AAPM TG Reports:**

- **Characterization of Motion:** Accurate assessment of the magnitude and pattern of respiratory motion using 4D-CT or other imaging modalities is crucial for selecting the appropriate management strategy and determining necessary margins.
- **Reproducibility:** The chosen motion management technique should be reproducible throughout the course of treatment. Regular monitoring and verification are essential.
- **Patient Compliance and Comfort:** The chosen method should be well-tolerated by the patient to ensure consistent and accurate treatment delivery.
- **Technology and Resources:** The availability of specific technologies (e.g., gating systems, real-time tracking) will influence the choice of motion management strategy.
- **Clinical Workflow:** The chosen technique should be integrated efficiently into the clinical workflow without causing excessive delays.
- **Quality Assurance:** Rigorous QA procedures are necessary to ensure the accurate and safe implementation of respiratory motion management techniques.

AAPM TG reports emphasize a tailored approach to respiratory motion management, considering the specific characteristics of the tumor, the patient's breathing pattern, available resources, and the desired level of precision for treatment delivery. The reports provide guidance on the implementation, quality assurance, and clinical considerations for each of these techniques.

GPT-4o:

Let’s walk through this **step by step** using guidance from **AAPM Task Group reports**, especially **TG-76**, **TG-142**, **TG-147**, and **TG-101**, which cover **respiratory motion management (RMM)** in **photon radiotherapy**.

**🎯 Why Manage Respiratory Motion?**

- During photon radiotherapy, **tumors in the thorax and abdomen** (e.g., lung, liver, pancreas) **move with breathing**.
- If unmanaged, this can lead to:
  - **Underdosing** the tumor
  - **Overdosing** healthy tissue
  - **Image artifacts** during IGRT

**🧩 AAPM-Recommended Respiratory Management Methods**

Based on **AAPM TG-76**, the **6 primary methods** to manage respiratory motion are:

**✅ 1. Motion Encompassing (Internal Target Volume - ITV)**

- Use **4D-CT** to capture tumor motion across the respiratory cycle.
- Contour an **ITV** that includes the full motion range.
- **Simple, but leads to larger treatment volumes**.

✅ *Best for:* Tumors with **predictable motion**
❌ *Limitations:* Larger margins, higher normal tissue dose

**✅ 2. Respiratory Gating**

- Radiation is delivered **only during a selected phase** of the breathing cycle (e.g., exhale).
- Requires external breathing signal or internal surrogate (e.g., fiducials, diaphragm).

✅ *Best for:* Patients with **consistent breathing**
❌ *Limitations:* Longer treatment time, complex QA

**✅ 3. Breath-Hold Techniques (DIBH / DEBH)**

- Patient holds breath in a specific phase:
  - **DIBH (Deep-Inspiration Breath Hold)**: Common in **left breast** and **lung** cases to spare heart/lung.
  - **Active Breath Control (ABC)** may be used to help.

✅ *Best for:* Breast, lung, and upper abdomen
❌ *Limitations:* Patient compliance, training required

**✅ 4. Real-Time Tumor Tracking**

- Imaging (e.g., fluoroscopy or implanted fiducials) tracks the tumor during treatment.
- Beam follows the tumor in real time (e.g., CyberKnife, MLC tracking).

✅ *Best for:* Tumors with **large, irregular motion**
❌ *Limitations:* Expensive, system-dependent, QA intensive

**✅ 5. Forced Shallow Breathing with Abdominal Compression**

- A **compression device** limits diaphragmatic movement, reducing tumor motion.

✅ *Best for:* Liver, pancreas
❌ *Limitations:* Discomfort, may not fully eliminate motion

**✅ 6. Gated Imaging or 4D Imaging**

- Used for **planning and verification**, not treatment itself.
- **4D-CT** or **4D-CBCT** helps assess motion during setup.

✅ *Best for:* All moving targets, for motion assessment
❌ *Limitations:* Imaging dose, time-consuming

Moonshot-v1:

According to AAPM TG reports and related studies, the respiratory movement management methods in photon radiotherapy can be categorized into several types, each with its own advantages and applications:

**1. Motion-Encompassing Methods**

- **Description**: These methods account for the entire range of respiratory motion during treatment planning and delivery. They do not actively manage motion but rather design the treatment to encompass the motion.
- **Techniques**:
  - **Slow CT**: Acquiring CT images over several respiratory cycles to account for motion.
  - **Inhalation and Exhalation Breath-Hold CT**: Capturing images at specific respiratory phases to understand motion.
  - **4D CT**: Using time-resolved imaging to capture motion over the respiratory cycle.

**2. Respiratory Gated Techniques**

- **Description**: These methods deliver radiation only during specific phases of the respiratory cycle, typically when the tumor is in a stable position.
- **Techniques**:
  - **External Respiratory Gating**: Using external devices to monitor breathing and trigger radiation delivery.
  - **Internal Respiratory Gating**: Using internal markers or imaging to monitor tumor position and control radiation delivery.

**3. Breath-Hold Techniques**

- **Description**: Patients hold their breath during radiation delivery to minimize motion.
- **Techniques**:
  - **Active Breathing Control (ABC)**: Devices that help patients hold their breath for short periods.
  - **Deep Inspiration Breath Hold (DIBH)**: Patients hold their breath at deep inspiration, reducing motion.

**4. Forced Shallow-Breathing Methods**

- **Description**: Techniques that reduce the amplitude of respiratory motion by encouraging shallow breathing.
- **Techniques**:
  - **Abdominal Compression**: Using belts or boards to restrict abdominal movement.
  - **Metronome Guidance**: Helping patients maintain a regular, shallow breathing pattern.

**5. Respiration-Synchronized Techniques**

- **Description**: These methods synchronize radiation delivery with the respiratory cycle, ensuring that the beam is on only when the tumor is in a specific position.
- **Techniques**:
  - **Real-Time Tumor Tracking**: Using imaging or external markers to track tumor position and adjust the beam in real-time.

**Recommendations**

- **Measurement of Tumor Motion**: AAPM TG-76 recommends measuring tumor motion for each patient when respiratory motion is a concern.
- **Threshold for Motion Management**: If target motion exceeds 5 mm, respiratory motion management is recommended.
- **Quality Assurance**: Respiratory motion management requires additional resources, education, and adherence to QA procedures.

These methods help improve the accuracy of radiation delivery, reduce margins, and spare normal tissues, leading to better treatment outcomes.

Question6 (TG75 & TG180)

Let’s think step by step. According to AAPM TG reports, in radiotherapy, how much dose should the image-guided process produce? How to reduce the dose of the image guidance process?

RTPhy-ChatBot:

In radiotherapy, the primary goal is to deliver an accurate and effective therapeutic dose to the target tumor while minimizing exposure to healthy tissues surrounding it. Image-guided radiotherapy (IGRT) plays a crucial role in ensuring precise tumor localization and positioning before each treatment session. However, IGRT also involves additional imaging doses that can contribute significantly to the overall radiation exposure for both patients and staff.

The amount of dose produced by the image-guided process depends on several factors such as the type and frequency of imaging modalities used, the imaging protocols, and the patient's anatomy. Generally, IGRT doses can range from a few cGy to several Gy depending on the specific application. For instance, kV X-rays used for cone-beam computed tomography (CBCT) can deliver doses ranging from 1 to 5 Gy, while kilovoltage (kV) or megavoltage (MV) portal imaging can deliver doses as low as a few cGy. According to reports by the American Association of Physicists in Medicine (AAPM) Task Group 75 and Task Group 180, the imaging dose should not exceed 5% of the therapeutic target dose.

To reduce the dose of the image guidance process, several optimization strategies have been recommended in the literature. Some of these strategies include:

1. Dose reduction techniques: Utilizing dose reduction techniques such as iterative reconstruction algorithms, adaptive shielding, and image noise reduction can help minimize the amount of imaging dose while maintaining diagnostic image quality.
2. Image fusion: Combining multiple imaging modalities (e.g., CT, MRI, CBCT) using image registration and fusion techniques can help reduce the need for repeated imaging sessions, thereby reducing overall imaging doses.
3. Adaptive planning: Implementing adaptive planning strategies that allow for online adjustments to treatment plans based on real-time imaging data can help minimize the number of imaging sessions required, thus reducing cumulative imaging doses.
4. Dose tracking and monitoring: Regularly monitoring and tracking the accumulated imaging doses for both patients and staff is essential to ensure that doses remain within acceptable limits. Implementing dose reduction techniques when possible can help minimize unnecessary exposures.
5. Education and training: Providing education and training to radiotherapy personnel on the importance of minimizing imaging doses, as well as best practices for implementing dose reduction strategies, can help promote a culture of safety and efficiency in radiotherapy departments.
6. Regulatory guidelines: Adhering to regulatory guidelines and standards for imaging dose optimization, such as those set forth by organizations like the American Association of Physicists in Medicine (AAPM), can help ensure that imaging doses are kept as low as reasonably achievable while maintaining diagnostic image quality and therapeutic efficacy.

By implementing these strategies, radiotherapy departments can reduce the amount of imaging dose produced during IGRT without sacrificing essential imaging information, ultimately enabling the design of more effective and efficient image guidance regimens.

Gemini-2.0-flash:

According to AAPM Task Group (TG) reports, particularly **TG-75 ("The management of imaging dose during image-guided radiotherapy")** and **TG-180 ("Image Guidance Doses Delivered During Radiotherapy: Quantification, Management, and Reduction")**, the dose produced by the image-guided process in radiotherapy is **not a fixed value or a strict limit.** Instead, the reports emphasize the following principles:

**How Much Dose Does Image Guidance Produce?**

- **Variability:** The dose from image guidance varies significantly depending on the imaging modality (kV Cone Beam CT - CBCT, MV CBCT, Electronic Portal Imaging - EPI, kV planar imaging, etc.), the frequency of imaging, the imaging protocols used (e.g., number of projections, mAs, kVp), and the size of the imaged volume.
- **Non-Negligible Dose:** AAPM reports highlight that the cumulative dose from repeated image guidance procedures throughout a course of radiotherapy can be **non-negligible** and should not be ignored. In some cases, it can contribute a significant fraction of the total dose received by the patient, especially to superficial tissues and organs outside the treatment target.
- **Quantification is Important:** The reports stress the need for **quantifying and documenting the imaging dose** delivered to patients. This includes understanding the dose from each imaging modality and the total cumulative dose over the treatment course.
- **Threshold for Consideration:** TG-180 suggests a **recommended threshold of 5% of the therapeutic target dose** beyond which the imaging dose should be explicitly considered in the treatment planning process. However, this is a guideline, and the ALARA principle should always be applied.

**How to Reduce the Dose of the Image Guidance Process:**

AAPM TG reports provide several strategies for reducing the dose from image-guided radiotherapy, emphasizing the **ALARA (As Low As Reasonably Achievable)** principle:

- **Optimize Imaging Frequency:**
  - Image only when clinically necessary. Avoid routine daily imaging for all patients if not warranted by the treatment site and potential for setup variations.
  - Consider risk-adapted imaging strategies where imaging frequency is adjusted based on the likelihood of setup errors or anatomical changes.
- **Optimize Imaging Protocols:**
  - **Reduce mAs (tube current-time product):** Lowering mAs directly reduces the number of photons produced, thus decreasing the dose. Image quality should be balanced with dose reduction.
  - **Reduce kVp (tube voltage):** While primarily affecting contrast, lower kVp can sometimes be used judiciously to reduce dose, especially for kV imaging.
  - **Reduce the Field of View (FOV):** Collimating the imaging beam to the region of interest minimizes the volume of tissue irradiated.
  - **Reduce the Number of Projections/Views:** For CBCT and planar imaging, using fewer projections can significantly lower the dose, although this may impact image quality.
  - **Use Low-Dose Protocols:** Many vendors offer specific low-dose imaging protocols that have been optimized for patient setup while minimizing radiation exposure.
  - **Consider Alternative Imaging Modalities:** If clinically appropriate, consider using lower-dose imaging modalities (e.g., kV planar imaging instead of kV-CBCT when only bony anatomy verification is needed).
- **Optimize Image Processing and Analysis:**
  - **Iterative Reconstruction Algorithms:** These advanced reconstruction techniques can improve image quality at lower doses compared to traditional filtered back-projection methods.
  - **Image Registration Techniques:** Accurate and efficient image registration can reduce the need for repeat imaging.
- **Utilize External Markers and Surrogate Tracking:** When appropriate, using external markers correlated with internal anatomy can reduce the frequency of high-dose volumetric imaging.
- **Education and Awareness:** Educating staff about the imaging doses associated with different protocols and the importance of dose reduction strategies is crucial.
- **Regular Review of Imaging Protocols:** Clinics should regularly review their imaging protocols to ensure they are optimized for the clinical need while adhering to the ALARA principle.
- **Consider Automated Workflows:** Implementing efficient workflows can minimize the time needed for imaging and patient setup, potentially reducing the need for repeat scans.

In summary, AAPM TG reports emphasize that the imaging dose in radiotherapy should be carefully managed and kept as low as reasonably achievable. While there isn't a strict dose limit, the cumulative imaging dose can be significant and should be quantified. Implementing various dose reduction strategies and adhering to the ALARA principle are essential to minimize the risk of secondary cancers and other radiation-induced effects.

GPT-4o:

Let’s break this down **step by step** without using tables, focusing on what AAPM Task Group (TG) reports say about **imaging dose in radiotherapy** and how to reduce it.

**Step 1: Understanding Why Imaging Dose Matters**

In IGRT (image-guided radiation therapy), frequent imaging—like kV planar X-rays, CBCT (cone-beam CT), and MV imaging—is used to ensure accurate patient positioning. However, these imaging procedures add a **non-therapeutic dose** to the patient. Although these doses are generally low, they **accumulate over the course of treatment** and must be managed, especially in pediatrics and long-course treatments.

**Step 2: How Much Imaging Dose is Typically Delivered?**

According to AAPM TG-75 and TG-180:

- **kV planar imaging** usually delivers a small dose, around 0.1 to 0.5 cGy per exposure.
- **kV-CBCT** can deliver between 1 and 3 cGy per scan, depending on the body site and scan length.
- **MV imaging** (planar or CBCT) tends to deliver higher doses—often between 2 to 5 cGy or more per scan.
- **4D-CBCT**, used for respiratory motion tracking, can be among the highest, possibly exceeding 5 cGy per scan.

These numbers vary depending on imaging technique, body region, scan parameters (kVp, mAs), and whether any dose-reduction protocols are in place.

**Step 3: How to Reduce Imaging Dose (According to AAPM)**

**First**, optimize imaging protocols. This means choosing the lowest possible kVp and mAs that still give usable image quality. You don’t need high-quality soft-tissue imaging for every case. For instance, bony alignment might be enough for prostate or head-and-neck treatments.

**Second**, reduce imaging frequency. Not every patient or every fraction needs daily CBCT. For stable setups, consider imaging every other day or weekly, depending on the site, patient-specific factors, and physician’s judgment.

**Third**, choose lower-dose imaging modalities when possible. kV planar imaging is much lower in dose than CBCT. When bony anatomy is sufficient for alignment, avoid CBCT. Also, avoid using MV imaging unless there’s a specific reason—MV imaging often has higher dose and poorer image contrast.

**Fourth**, minimize field size and scan length. Collimate your imaging field so it only includes what you need. Avoid unnecessarily long CBCT scans, especially when you're only treating a small region. For example, if you're treating the brain, don’t scan all the way through the thorax.

**Fifth**, use shielding when applicable. For certain areas—like the gonads or the lens of the eye—placing shields (when feasible) can protect sensitive tissues. This is especially important in pediatric or long-course treatments.

**Lastly**, perform regular QA and dose tracking. Follow AAPM TG-75 and TG-142 guidelines for imaging QA. Use phantoms to measure actual imaging dose periodically. Some departments implement systems to track cumulative imaging dose for each patient.

**Summary**

In summary, AAPM recommends keeping imaging dose as low as reasonably achievable (ALARA) while ensuring accurate treatment. Imaging dose per session typically ranges from a fraction of a cGy for planar imaging to a few cGy for CBCT. Strategies like protocol optimization, frequency reduction, modality selection, field limitation, shielding, and consistent QA can significantly reduce cumulative imaging dose over the course of treatment.

Moonshot-v1:

According to AAPM TG reports, the dose from the image-guided process in radiotherapy should be carefully managed to minimize patient exposure while ensuring effective treatment. Here are the key points:

**Dose from Image-Guided Process**

- The recommended threshold for considering imaging dose in the treatment planning process is **5% of the therapeutic target dose**. This means that if the imaging dose exceeds 5% of the dose intended for the target volume, it should be taken into account during planning.

**Methods to Reduce Imaging Dose**

1. **Optimize Imaging Techniques**:
   - Use **lower tube potentials** and **reduce tube current** to decrease dose while maintaining image quality.
   - Employ **bow-tie filters** during kV radiographs to reduce organ doses by 15-70%.
2. **Adjust Imaging Protocols**:
   - Limit the **scan range** to the region of interest to reduce unnecessary dose.
   - Use **intermittent imaging modes** (e.g., quick check mode) instead of continuous mode for procedures like CT fluoroscopy.
3. **Patient-Specific Adjustments**:
   - For patients below 80 kg, use **lower kV settings** (e.g., 90 kV instead of 120 kV) to reduce dose without significantly compromising image quality.
4. **Advanced Imaging Systems**:
   - Modern **kV imaging systems** generally deliver much lower doses compared to MV imaging, making them preferable for IGRT.
   - Use **4D CT and real-time imaging** techniques to better manage motion and reduce dose.

**General Recommendations**

- Always apply the **ALARA principle** (As Low As Reasonably Achievable) in practice to minimize imaging dose.
- Medical physicists should inform radiation oncologists about the imaging doses delivered to patients under their care.

By following these guidelines, the dose from the image-guided process can be effectively managed, ensuring patient safety while maintaining the accuracy and efficacy of radiotherapy treatments.

Question7

Let’s think step by step. According to AAPM TG reports, in stereotactic radiotherapy, what specific quality assurance programs are available for small field dosimetry?

RTPhy-ChatBot:

Stereotactic radiotherapy (SRT) and other advanced radiation therapy techniques such as intensity-modulated radiotherapy (IMRT) and volumetric-modulated arc therapy (VMAT) use small static photon fields to achieve highly focused and precisely modulated dose distributions. Measuring the dosimetry of these small fields is complicated due to various factors like loss of lateral electronic equilibrium, volume averaging, detector-interface artifacts, collimator effects, and detector position-orientation effects.

To ensure accurate and consistent dose delivery in stereotactic radiotherapy, specific quality assurance (QA) programs have been developed for small field dosimetry. These QA programs include:

1. Verification of mechanical tolerances: This involves checking the alignment and accuracy of the linac components, such as the collimator, gantry, and isocenter, to ensure that they meet the required specifications for delivering small fields.
2. X-ray/light field/laser alignment with isocenter: Proper alignment of the x-ray beam, light field, and laser with the isocenter is crucial in stereotactic radiotherapy. Misalignment can lead to significant dosimetric errors. Therefore, regular checks are performed to ensure accurate alignment.
3. Verification of target/tumor with isocenter prior to treatment: Before delivering the treatment, it's essential to confirm that the target or tumor is correctly positioned at the isocenter. This can be done using imaging techniques such as CT, MRI, or cone-beam CT (CBCT).
4. Software QA: Regular testing and calibration of the software used for treatment planning and dose calculation are necessary to ensure accurate results. This includes checking the frame coordinates of a target with a previously known coordinate and archiving test cases for routine software QA.
5. Dose/MU verification: Accurate measurement of the dose delivered to small fields is essential. Unshielded stereotactic diodes can be used for measurements in field sizes less than 3 cm × 3 cm, while small ionization chambers can be used for larger fields. Careful detector-phantom setup and detailed dose corrections are necessary to minimize discrepancies among the measurements.
6. Modeling of small fields: Advanced algorithms with more sophisticated inclusions such as 3D volume measurements (3D volumetric modulated arc therapy [VMAT] and intensity-modulated radiation therapy [IMRT]) have been shown to improve the quality of calculations for small fields. These programs should be tested in inhomogeneous media, including low-density materials, to ensure accurate results.
7. Use of appropriate detectors: The use of stereotactic detectors with a spatial resolution of approximately 1 mm or better is recommended for measuring the basic dosimetry data, such as total scatter factor, relative output factor, tissue-maximum ratio, and off-axis ratios, in small fields.
8. Regular reviews and evaluations: The algorithms for independent/second use with small fields should be regularly reviewed and evaluated to ensure their accuracy and suitability for clinical use. This includes describing commissioning and benchmark QA of secondary MU calculation programs and proposing additional modeling of small fields for homogeneous water medium.
9. Awareness and verification: Evaluation as IMRT/VMAT users should be aware of the complexity of these programs and the criteria for their use. More advanced algorithms with more sophisticated inclusions such as 3D volume measurements (3D VMAT and IMRT) have been shown to improve the quality of their calculations. Recommendations include the need for verification of the dose/MU calculation before treatment delivery or patient-specific quality assurance measurements.
10. Proper setup and careful handling: Careful detector-phantom setup, handling, and detailed dose corrections are necessary to minimize discrepancies among the measurements even with stereotactic detectors.

These QA programs aim to ensure accurate and consistent dose delivery in stereotactic radiotherapy while minimizing dosimetric errors and maintaining a high level of safety for patients and personnel.

Gemini-2.0-flash:

While AAPM TG reports like **TG-142 ("Quality Assurance of Medical Accelerators")** and **TG-218 ("Tolerance Limits and Methodologies for IMRT Measurement-Based Verification QA")** provide frameworks for quality assurance in radiotherapy, they emphasize the need for **tighter tolerances and more specific procedures** when dealing with the small fields used in stereotactic radiotherapy (SRS) and stereotactic body radiotherapy (SBRT).

Here's a breakdown of specific quality assurance programs and considerations for small field dosimetry in stereotactic radiotherapy, as informed by these and other relevant TG reports and the general understanding in the field:

**1. Commissioning of the Treatment Machine and Small Field Delivery System:**

- **Accurate Beam Modeling in the Treatment Planning System (TPS):** TG reports emphasize that the TPS must accurately model the characteristics of small fields, including output factors, beam profiles, and penumbra. This requires comprehensive commissioning measurements using appropriate small field detectors.
- **Small Field Output Factor Measurements:** Accurate determination of output factors for various small field sizes (cones or MLC-defined fields) is crucial. This often requires detectors with small active volumes to minimize volume averaging effects. AAPM TG-155 provides detailed recommendations on small field dosimetry.
- **Beam Profile Measurements:** Characterization of beam profiles (in-plane and cross-plane) at different depths for small fields is essential to ensure accurate dose calculation and delivery. High-resolution detectors are needed to capture the steep dose gradients in small fields.
- **Light-Radiation Field Coincidence:** Precise alignment between the light field and the radiation field is critical for accurate targeting in SRS/SBRT. Tolerances are typically tighter (e.g., ≤ 1 mm) compared to conventional radiotherapy.

**2. Routine Quality Assurance Procedures Specific to Small Fields:**

- **Output Constancy Checks:** Regular checks of the machine output for small field sizes should be performed with tight tolerances (e.g., ≤ 2%).
- **Beam Profile Constancy:** Monthly checks of beam profiles for representative small field sizes should be performed to ensure consistency with baseline data. Tolerances on profile parameters (e.g., full width at half maximum - FWHM, penumbra) are typically tighter (e.g., ≤ 1 mm).
- **Light-Radiation Field Coincidence Checks:** These checks should be performed regularly (e.g., monthly) with strict tolerances (e.g., ≤ 1 mm).
- **Isocenter Verification:** The accuracy and stability of the treatment isocenter are paramount in SRS/SBRT. QA procedures should include checks of the mechanical and radiation isocenter using tools with high precision (e.g., Winston-Lutz test). Tolerances are very tight (e.g., ≤ 1 mm).
- **Stereotactic Localization Accuracy:** End-to-end tests that simulate the entire treatment process, from imaging to delivery, are crucial. These tests often involve irradiating a phantom with known target structures and verifying the accuracy of targeting using imaging and dosimetry. Tolerances for targeting accuracy are typically ≤ 1-2 mm.

**3. Patient-Specific Quality Assurance (PSQA):**

- **Independent Dose Calculation Verification:** For each SRS/SBRT plan, an independent dose calculation should be performed to verify the TPS calculations, especially for small and highly conformal fields.
- **Measurement-Based Verification:** Due to the complexities of small field dosimetry and the high doses per fraction in SRS/SBRT, measurement-based PSQA is highly recommended. This often involves using small detectors or high-resolution dosimetry systems (e.g., film, diode arrays, ionization chamber arrays with fine spatial resolution) to verify the delivered dose distribution.
- **Gamma Analysis with Tighter Criteria:** When using gamma analysis to compare measured and calculated dose distributions, tighter criteria (e.g., ≤ 3% dose difference and ≤ 1-2 mm distance-to-agreement) are typically used compared to conventional IMRT QA. AAPM TG-218 provides a framework for establishing appropriate tolerance limits.
- **Consideration of Detector Size and Perturbation Effects:** When performing PSQA for small fields, it's crucial to use detectors with small active volumes and to apply appropriate correction factors to account for perturbation effects.

**Specific AAPM TG Reports Relevant to Small Field Dosimetry QA:**

- **AAPM TG-142:** While a general linac QA report, it emphasizes the need for tighter tolerances for SRS/SBRT modalities in various tests (e.g., mechanical isocenter, light/radiation field coincidence, output constancy).
- **AAPM TG-155 ("Megavoltage photon beam dosimetry in small fields and non-equilibrium conditions"):** This report specifically addresses the challenges and provides recommendations for accurate dosimetry in small photon fields, which is directly applicable to SRS/SBRT. It covers detector selection, output factor determination, and beam profile measurements.
- **AAPM TG-218:** While focused on IMRT QA, the principles of establishing tolerance limits and the importance of measurement-based verification are applicable to SRS/SBRT, with the caveat that tighter tolerances are generally required.
- **AAPM TG-101 ("Stereotactic Radiosurgery"):** This report provides comprehensive recommendations on the clinical implementation, medical physics support, and quality assurance of stereotactic radiosurgery programs. It discusses various aspects of small field dosimetry QA.
- **AAPM TG-135 ("Quality Assurance for Robotic Radiosurgery"):** This report focuses on the specific QA requirements for robotic radiosurgery systems, which often utilize small fields and require stringent accuracy.

**In summary, quality assurance for small field dosimetry in stereotactic radiotherapy requires a comprehensive program that includes:**

- Thorough commissioning with accurate small field characterization.
- Routine QA procedures with tighter tolerances specific to SRS/SBRT.
- Patient-specific QA with independent dose calculation verification and measurement-based verification using appropriate detectors and analysis criteria.
- Adherence to the recommendations provided in relevant AAPM TG reports, particularly TG-155 and those addressing SRS/SBRT specifically.

The emphasis is on ensuring the highest possible accuracy in dose delivery to the small target volumes while minimizing dose to surrounding critical structures, which necessitates rigorous and specialized QA procedures.

GPT-4o:

let’s walk through this **step by step** based on guidance from **AAPM Task Group reports**, particularly **TG-101**, **TG-155**, **TG-142**, and **TG-135**, which address **small field dosimetry and QA in stereotactic radiotherapy (SRS/SBRT)**.

**Step 1: Why Small Field Dosimetry Is Challenging**

Small fields, often less than 3x3 cm², are frequently used in stereotactic treatments (e.g., SRS, SBRT). These fields introduce significant dosimetric challenges due to:

- **Loss of lateral charged particle equilibrium**
- **Detector size relative to the field**
- **Partial occlusion by MLC leaves**
- **Steep dose gradients**

Therefore, **specialized QA procedures and tools** are essential to ensure **accuracy and patient safety**.

**Step 2: Relevant AAPM TG Reports**

Several AAPM Task Group reports offer guidance on small field dosimetry and QA:

- **TG-101**: SRS/SBRT QA guidance
- **TG-155**: Reference dosimetry for small and nonstandard fields
- **TG-142**: QA of medical linear accelerators (includes small-field tests)
- **TG-135**: CyberKnife-specific QA (a key SRS/SBRT system)

**Step 3: QA Programs for Small Field Dosimetry**

Here are the **specific QA programs and recommendations** from these reports:

**1. Detector Selection and Commissioning (TG-155)**
Use **detectors suitable for small fields**—ideally with:

- Small active volume (e.g., micro-ionization chambers, diodes, diamond detectors)
- High spatial resolution
- Minimal perturbation of the field

**2. Output Factor Measurement (TG-155)**
Measure **output factors** for all clinical small fields. Corrections may be needed due to detector response differences. Cross-comparison with Monte Carlo data or reference data sets is recommended.

**3. Beam Model Validation (TG-101, TG-155)**
Validate the **TPS beam model** for small fields using:

- Point dose measurements
- Profile comparisons (penumbra widths, FWHM)
- Film or high-resolution detectors in phantoms

**4. End-to-End Testing (TG-101, TG-135)**
Perform **end-to-end QA** using anthropomorphic phantoms that simulate:

- Imaging
- Planning
- Setup
- Dose delivery

Film and gel dosimetry can be used to verify spatial accuracy and dosimetric agreement.

**5. Periodic Machine QA (TG-142)**
Perform periodic QA of small field parameters including:

- MLC positioning accuracy
- Beam output constancy for small fields
- Winston-Lutz tests for geometric accuracy
- Beam profile constancy and symmetry

**6. CyberKnife-Specific QA (TG-135)**
If using CyberKnife or other non-isocentric systems, follow TG-135 guidelines:

- Use dedicated QA phantoms and log file analysis
- Test beam path accuracy and robotic arm repeatability

**Step 4: Frequency and Scope of QA**

- **Daily/Monthly QA**: Includes output constancy checks with small fields, MLC positioning, and image guidance system QA
- **Annual QA**: Includes comprehensive small-field dosimetry, output factor revalidation, and end-to-end testing

**Step 5: Summary and Key Takeaways**

To ensure accurate small field dosimetry in stereotactic radiotherapy:

- Use **small-volume, high-resolution detectors**
- Carefully **measure and validate output factors**
- Perform **end-to-end QA** for the full treatment chain
- Follow **TG-101, TG-155, TG-135, and TG-142** guidance
- Schedule QA tests appropriately (daily, monthly, annually)

Moonshot-v1:

According to AAPM TG reports, specific quality assurance (QA) programs for small field dosimetry in stereotactic radiotherapy (SRT) include the following:

**1. AAPM TG-155**

- **Introduction**: TG-155 provides comprehensive guidance on small field dosimetry, which is crucial for modern radiation therapy techniques like SRT, SRS (Stereotactic Radiosurgery), and SBRT (Stereotactic Body Radiation Therapy). These techniques often involve the use of small fields defined by multileaf collimators (MLCs) and cones.
- **Dosimetric Tools and Techniques**: The report discusses the dosimetric tools and techniques necessary for accurate small field dosimetry. This includes the use of microdetectors and correction factors for different types of detectors.
- **Field Size and Output Correction Factors**: TG-155 emphasizes the importance of determining field size experimentally, especially in small fields, and provides guidance on calculating output correction factors based on field size.

**2. AAPM TG-178**

- **Gamma Stereotactic Radiosurgery (GSR)**: TG-178 focuses on the dosimetry and QA for gamma stereotactic radiosurgery, specifically for devices like the Gamma Knife. The report provides detailed calibration protocols and QA recommendations for these specialized systems.
- **Calibration and QA Protocols**: The report recommends a GSR dose rate calibration formalism and provides tabulated data for implementing it with ionization chambers commonly used in GSR dosimetry.

**3. General QA Recommendations**

- **Routine Mechanical, Dosimetric, and Safety Checks**: Both TG-155 and TG-178 emphasize the importance of routine checks for mechanical, dosimetric, and safety aspects of the equipment used in stereotactic treatments.
- **Standardization and Safety**: The overall goal of these reports is to standardize practices and promote the safe implementation of stereotactic technologies.

These QA programs aim to ensure accurate dosimetry and safe delivery of radiation in small field applications, which are critical for effective treatment outcomes in stereotactic radiotherapy.

Question8 (TG132)

Let’s think step by step. According to AAPM TG reports, what are the sources of error in image registration? How to mitigate error?

RTPhy-ChatBot:

Image registration is a crucial process in medical imaging that involves aligning and integrating data from multiple modalities or frames within a single coordinate system for further analysis, treatment planning, or guidance. However, this process can introduce uncertainties due to various sources of errors. The following are the primary sources of error in image registration:

1. Input errors: These errors arise from the quality and accuracy of the input data used for registration. For instance, differences in image resolution, orientation, and distortions between modalities or frames can lead to registration errors. Additionally, misalignment of anatomical structures due to patient motion or organ deformation during scanning can also introduce errors.
2. Algorithmic errors: The choice of registration algorithm and its parameters can significantly impact the accuracy of the registration results. For example, rigid body registration may not be suitable for registering images with complex deformations or large translations. Similarly, the selection of inappropriate registration parameters, such as search range or iteration number, can lead to suboptimal registration results.
3. Output errors: The final output of the registration process can also introduce errors if it is not correctly interpreted or used for downstream applications. For instance, incorrect interpolation or extrapolation of registration results can lead to inaccurate contour propagation, dose accumulation, or image guidance. To mitigate these errors, several strategies can be employed:
4. Quality assurance and quality control techniques: These include verification and validation processes to ensure the accuracy and integrity of input data, as well as the selection of appropriate registration algorithms and parameters. Regular calibration and maintenance of imaging systems can also help reduce errors due to system misalignments or hidden indirect parameters.
5. Error propagation analysis: Fault trees and other error propagation analysis tools can be used to identify potential sources of error and their impact on the overall registration process. This information can then be used to prioritize error mitigation strategies and optimize workflows.
6. Mitigation techniques: Depending on the specific source of error, various mitigation techniques can be employed. For instance, image preprocessing steps such as normalization, denoising, or registration can help reduce errors due to differences in image resolution or distortions. Similarly, advanced registration algorithms, such as deformable registration or non-rigid body registration, can be used to register images with complex deformations or large translations.
7. User education and training: Proper user education and training on the use of imaging systems and registration software can help reduce errors due to incorrect interpretation or usage of registration results. Additionally, regular updates and maintenance of registration software can help ensure that users have access to the latest features and bug fixes.
8. Collaborative efforts: Finally, collaborative efforts between manufacturers, researchers, and clinicians can help identify and address sources of error in image registration. For instance, sharing best practices, developing standardized protocols, and participating in ongoing research initiatives can help improve the overall accuracy and reliability of the registration process.

Gemini-2.0-flash:

According to AAPM Task Group (TG) reports, particularly **TG-132 ("Use of image registration and fusion algorithms and techniques in radiotherapy")**, several sources of error can affect the accuracy of image registration in radiotherapy. Understanding these errors is crucial for ensuring the reliable use of image registration for tasks like target delineation, dose accumulation, and image-guided radiation therapy (IGRT).

Here are the main sources of error identified in AAPM TG reports:

**1. Image Artifacts and Noise:**

- **Metal Artifacts:** High-density materials like implants can cause significant distortions and signal voids in CT and MRI images, hindering accurate registration.
- **Motion Artifacts:** Patient movement during image acquisition (e.g., respiratory or peristaltic motion) can lead to blurring and geometric inaccuracies, making registration challenging.
- **Noise:** Random fluctuations in the image signal can affect the robustness and accuracy of registration algorithms, especially in low-contrast regions.
- **Contrast Differences:** Variations in contrast between different image modalities or time points can make it difficult for algorithms to find consistent features for alignment.

**2. Anatomical Changes and Deformations:**

- **Inter-fractional Changes:** Anatomical variations between different imaging sessions (e.g., tumor shrinkage, weight loss, organ filling) can introduce significant errors, especially for deformable registration.
- **Intra-fractional Motion:** Residual motion during image acquisition or treatment delivery that is not fully accounted for can lead to misregistration.
- **Deformable Anatomy:** Organs and soft tissues can change shape and position non-rigidly, making accurate deformable registration complex.
- **Surgical Changes:** Post-operative anatomical changes can make registration to pre-operative images challenging.

**3. Algorithm Limitations and Parameter Selection:**

- **Algorithm Type:** Different registration algorithms (rigid, affine, deformable) have varying capabilities and limitations in handling different types of transformations and anatomical changes. Choosing an inappropriate algorithm can lead to significant errors.
- **Parameter Settings:** Registration algorithms often have numerous parameters that need to be carefully tuned. Suboptimal parameter selection can result in inaccurate registration.
- **Interpolation Errors:** During image transformation, interpolation is used to estimate voxel values at new locations. The choice of interpolation method can introduce errors, especially with large deformations.
- **Assumptions of the Algorithm:** Algorithms often make assumptions about the nature of the transformation (e.g., small deformations, intensity consistency) that may not always hold true in clinical scenarios.

**4. Image Acquisition and Pre-processing:**

- **Geometric Distortions:** Imaging systems can have inherent geometric distortions that are not fully corrected during reconstruction, leading to misregistration.
- **Voxel Size and Resolution:** Differences in voxel size and image resolution between the reference and moving images can limit the achievable registration accuracy.
- **Image Orientation and Field of View:** Inconsistent image orientation or significant differences in the field of view can complicate the registration process.
- **Pre-processing Steps:** Image pre-processing techniques (e.g., filtering, intensity normalization) can affect the image content and subsequently the registration accuracy.

**5. User Error and Workflow Issues:**

- **Incorrect Landmark Placement:** If landmark-based registration is used, inaccurate manual placement of landmarks can introduce significant errors.
- **Inappropriate Region of Interest (ROI) Selection:** Defining an ROI that does not encompass the relevant anatomical structures can negatively impact registration accuracy.
- **Lack of Validation:** Not performing adequate validation of the registration results can lead to the acceptance of inaccurate registrations.

**How to Mitigate Error:**

AAPM TG reports recommend several strategies to mitigate errors in image registration:

- **Careful Image Acquisition:** Employ imaging protocols that minimize artifacts and distortions. Use immobilization devices to reduce patient motion.
- **Appropriate Algorithm Selection:** Choose a registration algorithm that is suitable for the specific clinical application and the expected types of transformations and anatomical changes.
- **Optimal Parameter Tuning:** Carefully select and optimize the parameters of the registration algorithm, potentially through validation studies.
- **Image Pre-processing:** Apply appropriate pre-processing techniques to improve image quality and reduce intensity differences, but be mindful of potential artifacts.
- **Use of External Markers or Fiducials:** Implanted or external markers can provide reliable corresponding points for registration, especially in regions with significant soft tissue deformation.
- **Region of Interest (ROI) Definition:** Carefully define the ROI to include relevant anatomical structures and exclude areas with significant artifacts or irrelevant motion.
- **Robust Validation Techniques:** Implement comprehensive validation procedures to assess the accuracy of the registration. This can include:
  - **Visual Inspection:** Qualitative assessment of the overlay and blended images.
  - **Landmark-Based Validation:** Comparing the positions of corresponding anatomical landmarks before and after registration.
  - **Contour-Based Validation:** Evaluating the overlap of corresponding contours using metrics like the Dice Similarity Coefficient.
  - **Phantom Studies:** Using phantoms with known transformations to quantify registration accuracy.
- **End-to-End Testing:** Incorporate image registration into end-to-end tests that simulate the entire radiotherapy workflow to identify potential errors.
- **Training and Standardization:** Ensure that all users are properly trained on the image registration software and adhere to standardized protocols.
- **Documentation:** Maintain thorough documentation of the registration parameters, validation results, and any identified issues.
- **Regular Quality Assurance:** Implement a regular QA program to monitor the performance of the image registration software and identify potential sources of error.
- **Consider Uncertainty:** Acknowledge and, where possible, quantify the uncertainty associated with the image registration process and consider its impact on downstream applications.

By understanding the potential sources of error and implementing appropriate mitigation strategies and robust QA procedures, the accuracy and reliability of image registration in radiotherapy can be significantly improved, leading to more precise and effective treatments.

GPT-4o:

let’s go step by step using guidance from **AAPM Task Group reports**, particularly **TG-132**, which is the primary report on **image registration and fusion** in radiotherapy.

**✅ Step 1: Understand What Image Registration Is**

**Image registration** is the process of aligning two or more images (CT, MRI, PET, CBCT, etc.) of the patient, either from different time points or from different modalities. This is essential in:

- Treatment planning (e.g., combining CT and MRI)
- Daily setup (e.g., aligning CBCT to planning CT)
- Adaptive radiotherapy

But registration can introduce errors — which can compromise **target coverage** or **OAR sparing** — so understanding and mitigating those errors is key.

**❌ Step 2: Main Sources of Error in Image Registration (from TG-132)**

**1. Modality Differences**
Different imaging modalities visualize tissues differently. For example, CT shows electron density, while MRI shows soft tissue contrast. This can lead to poor alignment, especially in automatic algorithms.

**2. Patient Anatomy Changes**
Weight loss, tumor shrinkage, or organ filling can lead to significant mismatches between images taken at different times.

**3. Motion Artifacts**
Respiration, cardiac motion, or patient movement during scan acquisition can distort anatomy and introduce misalignment.

**4. Image Quality Issues**
Low signal-to-noise ratio, poor contrast, or artifacts (like metal streaks in CT or distortion in MRI) make registration less accurate.

**5. Registration Method Error**
Errors can occur from the choice of registration algorithm:

- Rigid registration assumes no deformation (not ideal for soft tissue changes).
- Deformable registration can overfit or warp incorrectly if poorly constrained or validated.

**6. Human Error**
Manual or semi-automated registrations are user-dependent. Misinterpretation of anatomy, inconsistent landmark selection, or poor windowing can lead to mistakes.

**7. Inter-observer Variability**
Different users may align images differently, especially in ambiguous regions or soft tissues.

**✔️ Step 3: How to Mitigate Image Registration Errors**

**1. Validate with Anatomy**
Always confirm alignment using **known anatomical landmarks** — bones, airways, or organ boundaries — in multiple planes. Use fused views or checkerboard overlays.

**2. Use High-Quality Images**
Ensure imaging protocols provide **adequate contrast, resolution**, and minimal artifacts. Use immobilization and gating if needed to reduce motion.

**3. Choose the Right Algorithm**

- Use **rigid registration** for bony alignment or when no deformation is expected.
- Use **deformable registration** only when soft tissue shift is significant — and always validate carefully.
- Know the algorithm’s assumptions and limitations.

**4. Apply QA and Commissioning (TG-132 guidance)**
Before clinical use, each registration algorithm or software should be:

- **Commissioned** using digital and physical phantoms
- **Tested** with known deformations
- **Evaluated** for uncertainty ranges

**5. Perform Inter-observer Studies**
Regularly compare results between clinicians or physicists to check for consistency and refine protocols.

**6. Document Everything**
Clearly record:

- What images were registered
- Which algorithm was used
- Who performed the registration
- What quality checks were done

**7. Use Contour Propagation with Caution**
For propagated structures (especially via deformable registration), always **review and correct contours** as needed.

**🧠 Step 4: Key Takeaway**

According to AAPM TG-132, image registration is **not inherently accurate** — it requires **validation, user awareness**, and **quality assurance**. Sources of error come from anatomy, motion, modality differences, algorithm choice, and user input. These can be mitigated with **systematic review**, **good imaging practices**, and **QA protocols**.

Moonshot-v1:

According to AAPM TG reports and related studies, the sources of error in image registration and methods to mitigate these errors are as follows:

**Sources of Error in Image Registration**

1. **Geometrical Distortion in Preoperative Images**:
   - **Magnetic Field Inhomogeneity**: In MRI, static magnetic field inhomogeneities and gradient nonlinearities can cause geometrical distortions. These can be corrected using shims and electronic compensation circuits.
   - **Metal Artifacts**: Metals, especially ferromagnetic materials, can cause severe local warping of the static field. Common sources include dental fillings, implants, and shrapnel.
   - **Susceptibility Differences**: Objects with different magnetic susceptibilities (e.g., air-tissue interfaces) can induce perturbations in the static magnetic field, leading to distortions.
2. **Error Inherent in the Registration Process**:
   - **Image Quality**: Noisy images, movement artifacts, and distortions can affect registration accuracy.
   - **Missing Correspondences**: Pathological differences between images can lead to missing correspondences, complicating accurate alignment.
   - **Contrast Differences**: Variations in image contrast can mislead registration algorithms.
   - **Modeling Assumptions**: Incorrect assumptions in the registration model or optimization parameters can result in errors.
   - **Homogeneous Regions**: Lack of distinct features in homogeneous regions can hinder accurate local deformations.
3. **Intraoperative Brain Deformation**:
   - **Physiological Changes**: Variations in water content or anatomical changes due to treatment (e.g., lung tissue inflammation, tumor shedding) can affect registration accuracy.

**Methods to Mitigate Error**

1. **Improve Image Quality**:
   - Use higher gradient strengths in MRI to reduce spatial errors caused by susceptibility differences.
   - Employ artifact reduction techniques to minimize metal artifacts and susceptibility-induced distortions.
2. **Enhance Registration Algorithms**:
   - Combine image-based registration with mechanical modeling techniques to improve accuracy, especially in regions with significant deformations.
   - Use multi-metric registration, including mean distance to agreement (MDA), DICE similarity coefficient (DSC), Jacobian determinant (JD), and inverse consistency error (ICE) to evaluate and refine registration performance.
3. **Regular Quality Assurance**:
   - Regularly calibrate and validate imaging and registration systems to ensure consistent performance.
   - Perform routine mechanical, dosimetric, and safety checks for frameless image registration systems.
4. **Advanced Imaging Protocols**:
   - Utilize real-time imaging techniques to account for intraoperative changes and improve registration accuracy.

By addressing these sources of error and implementing the suggested mitigation strategies, the accuracy and reliability of image registration in clinical applications can be significantly enhanced.

Question9 (TG155)

Let’s think step by step. According to AAPM TG reports, how to measure the dosimetric indices in small fields?

RTPhy-ChatBot:

Measuring dosimetric indices in small fields presents unique challenges due to the size of the detectors and the lack of lateral electronic equilibrium. Here's a step-by-step approach to measuring dosimetric indices in small fields:

1. Define the Small Field: A small field is typically defined as having lateral dimensions smaller than 1-2 cm, which causes penumbrae at both sides of the field to overlap. The dimensions of most detectors commonly used in conventional fields become large compared to the small-field size, leading to physical repercussions on dosimetry.
2. Use Appropriate Detectors: In large fields, ionization chambers with negligible change in response as a function of field size are recommended for determining field output factors. However, even the smallest commercially available ionization chambers exhibit large fluence perturbations due to volume averaging in small fields. The solution proposed in the literature is to use an ionization chamber down to a field size where the boundaries of the detector are still at least a distance r LCPE away from the field edge and use a suitable small solid-state detector for smaller fields.
3. Consider Beam Quality: Small photon fields differ from conventional reference fields in their lateral dimensions, causing the dimensions of most detectors to become large compared to the small-field size. This has physical repercussions on dosimetry and requires consideration of beam quality.
4. Use Intermediate Field Method: The intermediate field method is a commonly adopted approach for measuring field output factors in small fields. It involves using an ionization chamber down to a field size where the boundaries of the detector are still at least a distance r LCPE away from the field edge and using a suitable small solid-state detector for smaller fields.
5. Evaluate Signal-to-Noise Issue: Microion chambers are best suited for small field dosimetry, but their signal-to-noise issue should be evaluated. Additionally, perturbation factors of these detectors should be taken into account.
6. Verify Centering of the Detector: Output factors are very sensitive to the position of the detector. Thus, verification of centering of the detector is important and could be performed by scanning across the field in both lateral dimensions to check that the maximum along each dimension coincide.
7. Consider Inhomogeneous Media: The implementation of calculations for inhomogeneous media is important. Evaluation as IMRT/VMAT users should be aware of the complexity of these programs and the criteria for their use. More advanced algorithms with more sophisticated inclusions such as 3D volume measurements (3D) volumetric modulated arc therapy (VMAT) and intensity-modulated radiation therapy (IMRT) have been shown to improve the quality of their calculations. Recommendations for the use of these programs include the need for verification of the dose/MU calculation before treatment delivery or patient-specific quality assurance measurements.
8. Independent Checks: Small field dosimetric measurements should be verified independently, either through measurements or through an independent external audit, such as that carried out by IROC-Houston or in European countries with postal dosimetry services. An end-to-end test from CT simulation through treatment delivery should also be performed and may involve an independent assessment of the delivered dose.
9. Guidelines and Recommendations: There are a number of guidelines and recommendations related to small-field dosimetry, including IAEA TRS-483, which focuses on relative dose measurements with respect to detector choice and the importance of more than one appropriate detector for these measurements. Important considerations for the measurement setup were also defined as well as some limited information regarding TPS calculations for small fields.

Gemini-2.0-flash:

According to AAPM Task Group (TG) reports, particularly **TG-155 ("Megavoltage photon beam dosimetry in small fields and non-equilibrium conditions")**, the measurement of dosimetric indices in small fields requires careful consideration due to the challenges arising from:

- **Loss of Lateral Charged Particle Equilibrium (LCPE):** In small fields, secondary electrons generated within the field may scatter out of the field before depositing their energy, leading to a change in the dose distribution compared to larger fields.
- **Source Occlusion:** The finite size of the radiation source and the collimating devices can lead to variations in the effective source position and penumbra characteristics for small fields.
- **Detector Volume Averaging:** The physical size of the detector can be comparable to or larger than the steep dose gradients in small fields, leading to an averaging of the measured dose and inaccuracies in determining peak doses and penumbra widths.
- **Detector Perturbation Effects:** The presence of the detector itself can perturb the radiation field, especially in small fields where the detector size is significant relative to the field dimensions.

**AAPM TG-155 provides comprehensive recommendations on how to measure dosimetric indices accurately in small fields. Key aspects include:**

**1. Detector Selection:**

- **Small Active Volume:** Use detectors with the smallest possible active volume to minimize volume averaging, such as:
  - **Small-diode detectors:** These offer high spatial resolution and are less energy-dependent than other solid-state detectors.
  - **Micro ionization chambers:** These have very small sensitive volumes (e.g., 0.01-0.001 cm³) but may have lower sensitivity.
  - **Radiochromic film:** Offers very high spatial resolution and is suitable for profile measurements but requires careful calibration and processing.
  - **Scintillation detectors:** Can have small effective measurement volumes and are water equivalent.
- **Water Equivalence:** Detectors should ideally be water equivalent to minimize perturbation of the photon and electron fluence.
- **Energy Dependence:** Consider the energy dependence of the detector response, especially when measuring off-axis ratios where the beam spectrum can change.
- **Perturbation Correction Factors:** Recognize that all detectors perturb the field to some extent. Apply appropriate perturbation correction factors (pdet​) when converting detector readings to absorbed dose. These factors depend on the detector type, size, and the field size. TG-155 provides guidance and references for these corrections.

**2. Measurement Techniques for Specific Dosimetric Indices:**

- **Output Factors (OF):**
  - Use small volume detectors and apply appropriate pdet​ factors, which can be significant for very small fields.
  - Extrapolation techniques using a series of detectors with varying sizes can help estimate the true output factor for very small fields.
  - Consider using the ratio of readings at a reference depth for the small field to that of a reference field (e.g., 10x10 cm²) measured with the same detector and applying the ratio of their respective perturbation correction factors.
- **Percentage Depth Dose (PDD) and Tissue Maximum Ratio (TMR/TPR):**
  - Use small volume detectors to minimize volume averaging, especially in the build-up region and regions with steep dose gradients.
  - Account for changes in the beam spectrum with depth, which can affect detector response.
  - For very small fields, the depth of maximum dose (dmax​) may shift superficially.
- **Beam Profiles and Penumbra:**
  - Use detectors with high spatial resolution (e.g., small diodes, film) to accurately measure the steep dose gradients in the penumbra region of small fields.
  - The penumbra width in small fields can be influenced by the source size and collimator design.
  - Correct for volume averaging effects of the detector by deconvolving the measured profile with the detector's spatial response function if necessary.
- **Off-Axis Ratios (OAR):**
  - Measure OARs with small, energy-independent detectors to account for changes in the beam spectrum and the loss of LCPE off-axis.
  - Volume averaging can be more significant off-axis where the field size relative to the detector size might be effectively larger in one dimension.

**3. Considerations for Different Small Field Delivery Systems:**

- **Cones:** For collimator cones used in stereotactic radiosurgery (SRS), the field size is well-defined at a specific distance. Measurements should be performed at the intended treatment distance.
- **Micro-Leaf Collimators (MLCs):** Small fields created with MLCs can have complex shapes and varying degrees of source occlusion depending on the leaf positions. Accurate modeling and measurement require careful attention to the effective field size and potential variations in output.
- **Robotic Radiosurgery Systems:** These systems often use non-coplanar beams and small, non-standard fields. Dosimetry requires specialized phantoms and detectors suitable for these complex geometries.

**In summary, AAPM TG-155 emphasizes the need for:**

- Using **small volume detectors** with well-understood characteristics.
- Applying appropriate **perturbation correction factors**.
- Employing measurement techniques that minimize **volume averaging**.
- Being aware of the unique challenges associated with different **small field delivery systems**.
- Validating measurements with **multiple detector types** and **independent methods** when possible.

By following these recommendations, medical physicists can obtain more accurate dosimetric data for the small fields used in stereotactic radiotherapy, leading to improved treatment planning and delivery.

GPT-4o:

Let’s break this down **step by step** based on guidance from AAPM Task Group reports, especially **TG-155** and **TG-101**, which deal with **small field dosimetry** in radiotherapy — critical for high-precision treatments like **SRS and SBRT**.

**✅ Step 1: Why Small Field Dosimetry Is Special**

Small radiation fields (typically <3×3 cm²) are used in stereotactic and IMRT/VMAT plans. Unlike standard fields, small fields:

- Lack lateral charged particle equilibrium
- Can be smaller than the detector’s active volume
- Are more sensitive to positioning errors and penumbra effects
- Often involve complex beam modulation (e.g., MLC shapes)

So, **conventional detectors and methods don’t work well**, and special care is required when measuring **dosimetric indices** like:

- Output factors
- PDD (percent depth dose)
- TPR (tissue phantom ratio)
- Profiles (FWHM, penumbra width)
- Relative dose distributions

**🧪 Step 2: Choose the Right Detector (TG-155 Guidance)**

You need detectors that are **small enough to avoid volume averaging** and **minimally perturb the field**. Recommended types include:

- **Silicon diodes** (e.g., SRS diodes)
- **Plastic scintillators**
- **Synthetic diamond detectors**
- **Small-volume ion chambers** (e.g., PTW PinPoint), with caution

Important: Each detector has strengths and weaknesses (e.g., diodes can be energy-dependent), so **correction factors may be required** — TG-155 provides guidance on that.

**📏 Step 3: Measurement of Dosimetric Indices**

**1. Output Factors**

- Measure the ratio of dose in small field to that in reference field (e.g., 10×10 cm²).
- Use correction factors (k-field, k-output) as described in TG-155.
- Place the detector precisely at the central axis — even small misalignments affect results.

**2. PDD or TPR**

- Use detectors with high spatial resolution to track steep gradients.
- Water phantom scanning systems are ideal.
- Scans should be centered with fine step sizes (~0.5 mm near dmax).

**3. Beam Profiles**

- Measure lateral dose profiles (in-plane and cross-plane) at relevant depths (e.g., dmax, 10 cm).
- Use high-resolution detectors (or film) to evaluate:
  - **FWHM**
  - **Penumbra width** (80%–20%)
  - **Flatness and symmetry**

**4. Dose Distribution Validation**

- Use radiochromic film or 2D arrays to compare calculated vs. measured dose distributions.
- Perform gamma analysis (e.g., 2%/1 mm or tighter for SRS).

**🔄 Step 4: Apply Correction Factors (TG-155 Emphasis)**

Many detectors **don’t read “true dose”** in small fields. You must apply **output correction factors** (kQclin,Qmsr) that compensate for:

- Detector size
- Energy response
- Perturbation effects

AAPM TG-155 gives detailed guidance and tables of correction factors based on field size, energy, and detector type.

**🧠 Step 5: Key Best Practices**

- Always **cross-check results** using multiple detector types if possible.
- Validate small field data against **published datasets** or Monte Carlo results.
- Perform **end-to-end tests** using phantoms to confirm overall system accuracy.
- Ensure that **beam model in TPS** matches measured small-field behavior (especially output factors and profiles).

**✅ Summary**

According to AAPM TG-155 and TG-101, to measure dosimetric indices in small fields:

1. Use **appropriate detectors** (small, high-res, minimal perturbation).
2. Apply **correction factors** to account for detector-specific responses.
3. Measure carefully: small errors in position or alignment = big errors in dose.
4. Validate measurements through **comparison, modeling, and QA tests**.

Moonshot-v1:

According to AAPM TG reports and related literature, measuring dosimetric indices in small fields involves several key considerations and recommendations:

**Measurement Techniques and Recommendations**

1. **Detector Selection**:
   - **Small Volume Detectors**: Use detectors with minimal energy, dose, and dose rate dependence. Micro-ionization chambers are recommended for small field dosimetry, though signal-to-noise ratio (SNR) should be carefully evaluated.
   - **Stereotactic Diodes**: For field sizes less than 1×1 cm², stereotactic diodes or electron diodes are recommended. These diodes are also ideal for radiosurgery beams.
   - **Liquid-Filled Ion Chambers, Diamonds, and Diodes**: These detectors are still being characterized and commissioned for reference dosimetry.
2. **Measurement Conditions**:
   - **Source-to-Surface Distance (SSD)**: If the field size is small compared to the detector and electronic equilibrium could be compromised, measurements should be performed at a greater SSD with proper correction.
   - **Multiple Detectors**: Dosimetric measurements should be carried out using more than one detector system to ensure accuracy.
3. **Reference Dosimetry**:
   - **IAEA/AAPM Formalism**: The IAEA/AAPM formalism provides a structured approach for reference dosimetry in small static MV photon fields. This includes defining machine-specific reference fields and clinical fields for accurate dosimetry.
   - **Perturbation Factors**: The lack of perturbation factors for small field ionization chambers increases measurement uncertainty. Efforts are ongoing to derive these factors.
4. **Relative Dosimetry**:
   - **Depth Dose and Output Factors**: Relative dose measurements, including depth dose functions and output factors, should be carefully evaluated. Specialized systems and alternative procedures may be necessary for accurate measurements.
5. **Treatment Planning System Validation**:
   - **Algorithm Suitability**: Treatment planning systems should be validated for small field dosimetry, especially in heterogeneous media. Monte Carlo methods are recommended for modeling dose from small fields in such media.
6. **Error Analysis and Uncertainty**:
   - **Uncertainty Limits**: Provide error analysis and limits of uncertainty in measurements. Spot checks and verifications of smaller fields should be carried out using at least one independent method (e.g., TLD, film, Monte Carlo).

**Practical Considerations**

- **Energy Spectrum Variations**: While the energy spectrum does vary in small fields (e.g., SRS, IMRT), these changes result in insignificant variations in stopping power ratios.
- **Stopping Power Ratios**: For most ion chambers, stopping power ratios in small fields are relatively the same as those in the reference field used in dosimetry codes of practice.
- **Independent Audits**: Small field dosimetry should have an independent audit by a different physicist, either internal or external (e.g., Radiological Physics Center verification).

By following these guidelines and recommendations, medical physicists can improve the accuracy and reliability of dosimetric measurements in small fields, enhancing the precision of treatments like stereotactic radiosurgery and stereotactic body radiotherapy.

Question10 (TG158)

Let’s think step by step. According to AAPM TG reports, what are the main sources of out-of-field dose in radiation therapy? What factors affect the out-of-field dose?

RTPhy-ChatBot:

The main sources of out-of-field dose in radiation therapy include photon leakage through the treatment head, radiation scattered from collimators and beam modifiers, patient scatter within the body, and neutrons produced from various interactions. For higher-energy (>10 MV) photon beams, there is an additional contribution from neutrons emanating from the treatment head, neutrons produced from photoneutron interactions in the patient, and radioactive isotopes produced in photoneutron interactions.

The out-of-field dose depends on several factors, including:

1. Distance from the field edge: The out-of-field photon dose decreases in a roughly exponential manner with distance from the field edge. In high gradient regions near the field edge, small differences in plan optimization can substantially impact the absolute dose delivered to a point in the patient.
2. Field size: The out-of-field dose increases with increasing field size because larger irradiated volumes produce more patient scatter. This field-size dependence is most pronounced close to the field edge, where patient scattered radiation is the most important component of the out-of-field dose. Farther from the field edge, there is less field size dependence because this dose is dominated by head leakage.
3. Modulation: For modulated treatments, the dose outside the treatment field also depends on beam modulation. The dose increases with increasing modulation because more head leakage and more collimator scatter is generated as the number of monitor units (MU) increases to deliver the modulated fields. The dependence on modulation is most noticeable farther from the treatment field; near the field edge, the dose is dominated by patient scatter, and patient scatter depends only on the volume of tissue irradiated.
4. Accelerator model: Out-of-field dose varies slightly among accelerator models due to differences in head shielding designs.
5. Neutron production: Neutron production increases with beam energy as well as with field size. In scanning beams, internal neutrons are the dominant source of out-of-field dose equivalent because the external neutron contribution is very low. For both passive scattering and scanning beam proton therapy, the dose due to neutrons decreases as a function of distance from the field edge, largely because the internal neutron production increases with beam energy as well as with field size.
6. Depth in the patient: The dose varies minimally with depth in the patient, except near the surface of the patient, where it is highly elevated (typically by a factor of 2-3).
7. Collimator angle and design: Fraass and van de Geijn found that collimator scatter plus leakage is of the same order of magnitude as patient scatter, and for some machines, the leakage component can vary by a factor of 2 depending on the collimator angle. Kase et al. also indicate that near the beam, collimator scatter contributes 20% to 40% of the total peripheral dose, and leakage becomes the main contributor at greater distances from the field edge. Greene et al.

In summary, the main sources of out-of-field dose in radiation therapy are photon leakage, collimator scatter, patient scatter, and neutrons. The out-of-field dose depends on factors such as distance from the field edge, field size, modulation, accelerator model, depth in the patient, and collimator angle and design.

Gemini-2.0-flash:

According to AAPM Task Group (TG) reports, particularly **TG-158 ("Measurement and calculation of doses outside the treated volume from external-beam radiation therapy")**, the main sources of out-of-field dose in photon radiation therapy are:

**1. Head Leakage:**

- Photons that penetrate the shielding of the linear accelerator (linac) head. This leakage radiation is present even when the beam is nominally "off."

**2. Collimator Scatter:**

- Photons scattered from the primary and secondary collimators (jaws and MLCs) as the beam is shaped. This scatter originates from interactions of the primary beam with the collimating devices.

**3. Patient Scatter:**

- Photons scattered within the patient's body from the primary beam interacting with tissues. This scatter can travel to regions outside the direct beam path.

**Factors that affect the out-of-field dose:**

Several factors influence the magnitude and distribution of the out-of-field dose:

- **Beam Energy:** Higher energy photons generally produce more penetrating scatter and can increase head leakage, potentially leading to higher out-of-field doses at larger distances from the field edge. However, the angular distribution of patient scatter also depends on energy.
- **Field Size:** Larger field sizes generally result in more scatter from the collimators and within the patient, increasing the out-of-field dose, especially closer to the field edge. However, at larger distances, head leakage might become a more dominant component, and its dependence on field size might be less direct.
- **Treatment Technique:**
  - **IMRT (Intensity-Modulated Radiation Therapy) and VMAT (Volumetric Modulated Arc Therapy):** These techniques often involve more complex beam modulation with smaller segments and greater use of the MLCs, which can increase head leakage and scatter compared to conventional techniques.
  - **Stereotactic Radiotherapy (SRS) and SBRT (Stereotactic Body Radiation Therapy):** While using small fields, the high doses per fraction can still lead to non-negligible out-of-field doses.
- **Distance from the Field Edge:** The out-of-field dose generally decreases with increasing distance from the edge of the treatment field. The rate of decrease depends on the source of the radiation (leakage, scatter) and the attenuating properties of the materials involved.
- **Depth in Tissue:** The out-of-field dose distribution within the patient will vary with depth due to attenuation and scatter of the secondary radiation.
- **Beam Modifiers:** The use of wedges, bolus, or other beam shaping devices can affect the amount and distribution of scattered radiation, thus influencing the out-of-field dose.
- **Linac Design:** The shielding effectiveness of the linac head and the design of the collimation system are critical factors in determining the amount of head leakage. Different linac models and manufacturers can have varying levels of leakage.
- **Treatment Room Characteristics:** Scatter from the treatment room walls, ceiling, and floor can contribute to the very low dose levels far from the treatment field, although this is usually a minor component compared to head leakage and patient scatter closer to the field.
- **Patient Size and Composition:** The amount of patient scatter depends on the volume of tissue irradiated and its density. Larger patients will generally produce more scatter.

Understanding these sources and factors is crucial for estimating and potentially reducing the out-of-field dose, which is important for minimizing the risk of secondary cancers and other late effects, especially in long-term survivors. AAPM TG-158 provides detailed guidance on the measurement and calculation of these doses.

GPT-4o:

Let’s break this down **step by step**, using the guidance provided by the AAPM Task Group reports, specifically **TG-101**, **TG-111**, **TG-154**, and **TG-156**, which address the key concepts related to **out-of-field dose** in radiation therapy.

**✅ Step 1: What is Out-of-Field Dose?**

**Out-of-field dose** refers to the dose received by tissues outside the primary radiation treatment field. It is not part of the treatment volume but can still contribute to unintended radiation exposure. This dose arises from scattered radiation, leakage, and secondary radiation production.

In radiation therapy, **out-of-field dose** is a concern because:

- It can contribute to **secondary cancers** or **normal tissue damage**.
- It may affect **organ-at-risk (OAR)** tissues that are close to the treatment field.

**❌ Step 2: Main Sources of Out-of-Field Dose**

According to AAPM TG-101 and TG-111, the primary sources of out-of-field dose include:

**1. Scatter Radiation**

- **Primary beam scatter**: As the radiation interacts with the treatment volume (e.g., tumor or tissue), a portion of the primary beam is scattered and extends outside the field.
- **Electron contamination**: Particularly in electron beam treatments, electrons can scatter and contribute to dose beyond the prescribed field.

**2. Leakage Radiation**

- **Linear accelerator leakage**: Despite shielding, some radiation will leak from the collimator, head, and other parts of the treatment machine.
- **Radiation leakage from the treatment head**: This radiation contributes to out-of-field dose even when the beam is not directed at those regions.

**3. Secondary Radiation Production**

- **Neutron production**: High-energy photon beams (>10 MV) can produce neutrons when interacting with matter. These neutrons can scatter and contribute to out-of-field dose, potentially delivering higher doses further from the treatment field.
- **Prompt gamma rays**: These are secondary photons produced during interactions within the patient. In some cases, these photons can scatter and affect out-of-field tissues.

**⚖️ Step 3: Factors That Affect Out-of-Field Dose**

AAPM TG-111 and TG-156 explain the main factors that influence the magnitude and distribution of out-of-field dose:

**1. Beam Energy**

- Higher energy beams (e.g., >10 MV) produce **more neutron contamination**, leading to higher out-of-field doses. Neutron production increases with energy, and these neutrons can travel farther and deposit dose at greater distances from the treatment site.

**2. Field Size and Shape**

- **Larger treatment fields** tend to produce higher out-of-field doses, as there is more scattered radiation to be generated.
- **Field shape** can also affect the pattern of scatter — more irregular fields (e.g., with MLC modulation) may produce dose distributions that are different from simple rectangular fields.

**3. Treatment Technique**

- Techniques like **IMRT** and **VMAT** can lead to more out-of-field dose due to **multiple segments** and **modulated beams**. As the number of treatment fields increases, the total scatter dose outside the target volume increases.
- **Stereotactic treatments** (SRS, SBRT) are often more concentrated in smaller fields, but the **high dose gradients** near the edge of the field can also cause significant out-of-field doses in tissues adjacent to the treatment site.

**4. Patient Geometry and Anatomy**

- The **patient's size and shape** affect how much scatter occurs. Larger patients may lead to higher scatter doses.
- The presence of **bone or dense tissue** within or near the treatment area can increase scatter radiation since these materials tend to scatter more radiation.

**5. Treatment Delivery System**

- **Multileaf collimators (MLCs)**: While MLCs help shape the beam, they also contribute to scatter outside the field. The **positioning of the MLC leaves** and the **width of the leaves** can impact the amount of out-of-field dose produced.
- **Treatment technique**: As noted, **IMRT, VMAT**, and **stereotactic treatments** may have more out-of-field dose due to the modulated, multiple beams or arcs used.

**6. Collimator and Shielding**

- The use of **collimators** or **beam modifiers** (e.g., bolus, compensators) affects the dose distribution, as they can increase or decrease scatter in the out-of-field regions.
- **Machine shielding**: The amount and quality of shielding in the treatment machine affect leakage radiation levels. Poor shielding leads to more leakage and higher out-of-field doses.

**✔️ Step 4: How to Mitigate Out-of-Field Dose**

Based on the recommendations from TG-101 and TG-111, here are several methods for minimizing out-of-field dose:

**1. Use Lower-Energy Beams (when possible)**

- Using lower-energy beams can reduce neutron production, but this comes with trade-offs in terms of tumor penetration. In many cases, higher-energy beams may be necessary for deeper tumors.

**2. Shielding**

- Ensure adequate **shielding of the treatment head** to minimize leakage radiation. Modern linear accelerators have improved shielding designs that can reduce out-of-field dose.

**3. Minimize Field Size**

- Use **smaller fields** whenever possible to reduce the scatter dose produced. Techniques like **IMRT** should be optimized to minimize unnecessary segments or areas that contribute to out-of-field dose.

**4. Use Techniques That Minimize Scatter**

- **Proton therapy** and other heavy ion therapies can significantly reduce out-of-field doses compared to photon-based therapies, due to their **Bragg peak** characteristics, which deliver most of the dose within a small region.

**5. Regular QA for Machine Performance**

- **Check for leakage radiation** in regular QA procedures, ensuring that any excessive leakage is corrected through shielding or maintenance.
- Test **MLC accuracy** and alignment during QA to minimize scatter from misaligned leaves.

**6. Limit Treatment Volume and Delivery Time**

- Reducing the treatment volume or limiting the number of treatment fields (especially in complex plans like IMRT) can help minimize scatter and leakage.

**🔑 Key Takeaways**

From AAPM TG-101 and TG-111, the main sources of out-of-field dose are **scatter radiation, leakage radiation**, and **secondary radiation (such as neutrons)**. Factors affecting it include **beam energy, field size, patient anatomy**, and the **treatment technique**. Mitigating strategies include using **lower-energy beams, effective shielding**, **smaller fields**, and optimizing treatment techniques.

Moonshot-v1:

According to AAPM TG reports and related studies, the main sources of out-of-field dose in radiation therapy and the factors that affect it are as follows:

**Main Sources of Out-of-Field Dose**

1. **Leakage from the Linear Accelerator (Linac) Treatment Head**:
   - Leakage radiation from the linac head contributes significantly to out-of-field dose. Different linac models with varying shielding designs can generate different levels of out-of-field dose.
2. **Scatter from Collimation Devices**:
   - Scatter from collimators, including multileaf collimators (MLCs) and jaws, is another major source of out-of-field dose. The design of the collimation system can influence the amount of scatter radiation.
3. **Scatter from Within the Patient’s Body**:
   - Patient scatter occurs when the primary beam interacts with the patient’s tissues, resulting in secondary radiation that contributes to out-of-field dose. The extent of patient scatter can depend on the size and shape of the treatment field.

**Factors Affecting Out-of-Field Dose**

1. **Linac Design and Type**:
   - Different linac models can produce varying levels of out-of-field dose due to differences in shielding and collimation designs. For example, linacs with larger horizontal waveguides or bending magnets may produce higher out-of-field doses.
2. **Treatment Technique**:
   - Intensity-modulated radiotherapy (IMRT) and stereotactic radiotherapy (SRT) often involve higher out-of-field doses compared to conventional techniques. This is partly due to the increased number of monitor units required for these advanced techniques.
3. **Field Size**:
   - Larger field sizes generally result in higher out-of-field doses due to increased patient scatter. However, the difference in out-of-field dose between different field sizes decreases with increasing distance from the field edge.
4. **Beam Energy**:
   - Lower-energy beams tend to produce higher out-of-field photon doses compared to higher-energy beams. However, high-energy beams may generate neutrons through photonuclear interactions, which can also contribute to out-of-field dose.
5. **Collimator Orientation**:
   - The orientation of the collimator can significantly affect out-of-field dose. Aligning the patient’s craniocaudal axis with the x-plane of the collimator can reduce out-of-field dose by up to an order of magnitude.
6. **Treatment Planning and Delivery**:
   - The specific treatment plan and delivery method can influence out-of-field dose. For example, using conformal beams and avoiding beams directed toward the trunk can reduce dose to organs at risk.

Understanding these sources and factors can help in optimizing treatment planning and delivery to minimize out-of-field dose and associated risks.

Question11 (TG176)

Let’s think step by step. According to AAPM TG reports, in radiotherapy, how much impact does couch and different kinds of patient immobilization devices have on patient dose in radiation therapy?

RTPhy-ChatBot:

Couch tops and various types of patient immobilization devices can significantly affect the delivered dose to patients in radiation therapy. The primary concerns are related to skin dose and attenuation effects.

Skin Dose: The buildup effect of the couchtop can increase the skin dose due to the photons that scatter from the surface of the couchtop. Additionally, attenuation by the couchtop and immobilization devices can also affect the delivered target dose. For example, attenuation through carbon fiber couchtops ranges from about 2% for normally incident beams to as much as 17% for more dense sections, and these values increase with the combination of the couch top and immobilization device.

Attenuation Effects: The attenuation effects can lead to a reduction in dose at depth while increasing skin dose. For instance, up to 11% attenuation has been reported from the combination of couch and various immobilization devices including body and head frames used for stereotactic radiosurgery.

Couch Top Modeling: To accurately compute the dosimetric effects for indexed patient treatments, it is recommended that treatment planning systems include accurate models of couch tops. This will help ensure that the physicist can account for the couch top (and rails if present) during the planning process.

Proton Beams: For proton beams, when they traverse the treatment couch or immobilization device prior to the patient, the distal range of the treatment beam is shifted toward the patient surface. This change in distal range must be taken into account in the treatment planning process to accurately predict dose in the patient. The literature on the dosimetric effect of the treatment couch or immobilization devices for heavy charged particles is sparse.

Clinical Recommendations: To mitigate the impact of couch and immobilization devices on patient dose, clinical physicists, treatment planning system vendors, and device vendors are encouraged to work together to provide accurate couch top models in planning systems. Accurate measurements of surface dose and attenuation should also be reported by vendors.

In summary, couch tops and various types of patient immobilization devices can significantly impact the delivered dose to patients in radiation therapy through skin dose effects and attenuation. It is essential to consider these factors during treatment planning and to ensure accurate modeling of couch tops in treatment planning systems.

Gemini-2.0-flash:

According to AAPM Task Group (TG) reports, particularly **TG-176 ("Dosimetric effects caused by couch tops and immobilization devices")**, the treatment couch and patient immobilization devices can have a **non-negligible impact on the patient dose** in radiation therapy. This impact is a complex combination of:

- **Increased Skin Dose:** Devices in the beam path, especially those made of denser materials, can cause backscatter of photons and electrons, leading to an increase in the dose received by the patient's skin and superficial tissues.
- **Reduced Tumor Dose:** The materials of the couch and immobilization devices can attenuate the primary radiation beam, resulting in a reduction of the dose delivered to the target volume, especially for beams passing through these devices.
- **Altered Dose Distribution:** The presence of these external devices can scatter and absorb photons, leading to a perturbation of the planned dose distribution within the patient.

**Specific Impacts:**

- **Treatment Couch:**
  - **Modern Carbon Fiber Couches:** While optimal for imaging due to low attenuation at kV energies, monolithic carbon fiber couch tops can cause more attenuation and increased skin dose compared to older "tennis racket" style couches, especially for beams incident from posterior angles (patient lying supine).
  - **Magnitude of Dose Change:** The attenuation by carbon fiber couches can range from a few percent to more significant values depending on the beam energy, gantry angle, and the specific couch design. The increase in skin dose can also be substantial in certain configurations.
- **Immobilization Devices:**
  - A wide variety of immobilization devices are used in radiotherapy, including masks, headrests, breast boards, vacuum bags, and body frames.
  - **Attenuation:** These devices, depending on their material (e.g., plastics, foams, carbon fiber components) and thickness, can attenuate the beam to varying degrees. This attenuation needs to be accounted for to ensure accurate dose delivery to the target.
  - **Skin Dose Increase:** Similar to the couch, immobilization devices in the beam path can contribute to an increased skin dose due to backscatter. This effect is often overlooked or underestimated.

**AAPM Recommendations and Considerations:**

- **Modeling in Treatment Planning Systems (TPS):** TG-176 strongly recommends that treatment planning systems should have the capability to model the attenuation of couch tops and immobilization devices. This allows physicists to accurately calculate the dose delivered to the patient, considering the presence of these devices.
- **Measurement of Attenuation and Surface Dose:** The report emphasizes the need for medical physicists to measure the attenuation properties of the specific couch tops and commonly used immobilization devices in their clinic for different beam energies. They should also quantify the increase in surface dose caused by these devices.
- **Reporting of Device Properties:** AAPM recommends that device vendors provide information on the attenuation and scatter properties of their products to facilitate accurate dose calculations.
- **Beam Avoidance:** When possible, treatment planning should aim to avoid directing high-energy beams through dense components of the couch or immobilization devices, especially if the attenuation is significant and not accurately modeled.
- **Clinical Significance:** The dosimetric impact of these devices can be clinically significant, particularly for high-dose treatments, superficial targets, or when the beam passes through a substantial thickness of the device. Ignoring these effects can lead to underdosing the tumor or unexpected skin reactions.

In summary, AAPM TG reports highlight that the treatment couch and patient immobilization devices are not dosimetrically transparent. They can cause measurable attenuation of the beam and an increase in skin dose. Accurate modeling of these effects in the TPS and careful consideration during treatment planning are essential to ensure accurate dose delivery and minimize unintended side effects.

GPT-4o:

Let's go through the issue of the **impact of couches and patient immobilization devices on dose** step by step, based on the AAPM Task Group (TG) reports, particularly **TG-148**, **TG-101**, and **TG-132**. These reports discuss the effects of immobilization devices and treatment couches on patient dose during radiotherapy, and how to manage these effects effectively.

**✅ Step 1: Understanding the Role of Treatment Couches and Immobilization Devices**

In radiotherapy, **treatment couches** and **patient immobilization devices** are critical for ensuring that patients are accurately positioned during treatment. However, these devices can interact with the radiation beam in ways that affect dose distribution. Their impact is particularly important for treatments that require high precision, like **IMRT**, **SBRT**, and **SRS**.

**❌ Step 2: Impact of Couches on Patient Dose**

**1. Radiation Attenuation**

- The **treatment couch** can attenuate or scatter radiation, leading to a **reduction in dose** reaching the patient, especially for **superficial tumors** or **treatment areas near the surface**. This is because the couch is made of materials (like **carbon fiber**, **plastic**, or **metal**) that can absorb or scatter the radiation.

**Impact on Dose:**

- - **High-Energy Beams (e.g., 6 MV or higher)**: The attenuation from a typical carbon fiber couch is relatively low but can still cause minor dose reductions (typically 1-3%).
  - **Low-Energy Beams (e.g., 3 MV or less)**: Couches tend to have a more pronounced effect, and the dose reduction could be more significant due to the higher attenuation of the materials at these energies.

**2. Scatter Radiation**

- **Scattered radiation** from the treatment couch can contribute to **out-of-field dose** and affect areas outside the treatment volume, which may lead to unwanted doses to adjacent tissues or organs.

**Impact on Dose:**

- - The **scatter contribution** varies depending on the type of couch, its material, and its position relative to the treatment field.
  - **Couch design and composition** play a major role here. For instance, **carbon fiber couches** generally contribute less scatter compared to **metal-based** couches.

**❓ Step 3: Impact of Immobilization Devices on Patient Dose**

**1. Immobilization Materials Interaction with Radiation**

- **Immobilization devices**, like **thermoplastic masks**, **spacers**, and **vacuum cushions**, are typically made from materials like **polypropylene**, **silicone**, or **foams**. These materials may absorb or scatter radiation, causing slight dose changes to the patient.

**Impact on Dose:**

- The **impact is generally small** and often negligible for high-energy photon beams. For example, thermoplastic masks may reduce dose by **1-2%**, and vacuum cushions may cause similar effects.
- In contrast, **electron beams** can be affected more significantly due to the high dose gradients near the surface. In such cases, materials like **bolus** (used in electron therapy to bring dose closer to the skin) can **increase the dose** in the skin area.

**2. Material Composition and Thickness**

- The **thickness** and **density** of the immobilization device materials directly impact dose perturbation. Thicker or denser materials result in higher attenuation of the beam.
- Some devices may cause **dose enhancement** or **dose reduction**, depending on how they interact with the radiation beam.

**3. Misalignment or Inaccurate Immobilization**

- **Poor alignment** or **shift in immobilization** can lead to **treatment inaccuracies**, which indirectly affect dose delivery, especially in precise treatments like **SBRT** or **IMRT**.

**Impact on Dose:**

- - A misalignment could result in the tumor receiving **less dose** (if it shifts out of the optimal treatment area), or **more dose** (if a nearby organ-at-risk is unintentionally irradiated).

**🔧 Step 4: Methods to Minimize Impact**

**1. Optimize Couch and Immobilization Device Design**

- **Use materials with low atomic number** (e.g., **carbon fiber** for couches) to minimize **beam attenuation** and **scatter**.
- **Minimize the thickness** of the devices as much as possible, especially in areas that are close to the treatment target. Thin, low-density materials help to **reduce dose perturbation**.

**2. Ensure Proper Positioning**

- Consistently verify that **patient immobilization** devices are placed correctly. Even small displacements can lead to large changes in dose delivery, especially in **high-precision treatments**.
- Perform regular **patient setup checks** (such as kV imaging or CBCT) to confirm that the patient is positioned exactly as planned.

**3. Use of Bolus and Compensators**

- For certain treatment techniques, like **electron therapy**, **bolus** material may be used to bring dose closer to the surface. However, bolus material can also introduce **additional dose perturbation**, so careful selection of the bolus material and thickness is necessary.

**4. Quality Assurance (QA)**

- Implement a robust **QA program** to monitor the **dose effects** of immobilization devices and couches. This could involve:
  - **Dosimetric measurements** with phantom setups to account for couch/immobilization perturbations.
  - Regular checks for **couch positioning accuracy** and **material integrity**.

**📚 Step 5: Key Takeaways**

From AAPM TG reports (TG-148, TG-101, TG-132):

- **Couches** can attenuate or scatter radiation, potentially reducing the dose delivered to the target and contributing to out-of-field dose. Carbon fiber is often preferred for minimizing attenuation and scatter.
- **Immobilization devices** can also cause slight dose changes depending on their material and thickness, although this is usually minimal for high-energy photon treatments.
- **Patient setup** and **immobilization accuracy** are crucial, as even small misalignments can significantly affect dose delivery.
- Minimizing the use of **high-density materials**, optimizing the **thickness of immobilization devices**, and using **effective QA procedures** can help mitigate these effects.

Moonshot-v1:

According to AAPM TG reports and related studies, the impact of couch tops and patient immobilization devices on patient dose in radiotherapy can be significant. Here are the key findings:

**Impact of Couch Tops and Immobilization Devices on Patient Dose**

1. **Couch Tops**:
   - **Dose Attenuation**: Couch tops can cause significant dose attenuation. For example, studies have shown that couch tops can reduce the dose to the isocenter by up to 5.8%. This can lead to clinically significant reductions in target volume coverage.
   - **Skin Dose Increase**: Couch tops can also increase skin dose due to interactions with the radiation beam. This can be particularly problematic in treatments like stereotactic body radiotherapy (SBRT) where high doses are delivered.
2. **Immobilization Devices**:
   - **Skin Dose Increase**: Immobilization devices such as vacuum bags and head-and-neck immobilization devices can significantly increase skin dose. For example, vacuum bags can increase skin dose by up to 43% for a 10 cm thick bag. The skin dose increase depends on the thickness and density of the device.
   - **Dose Attenuation**: Immobilization devices can attenuate the beam, reducing the dose to the target volume. For example, head-and-neck immobilization devices can reduce the dose coverage of the planning target volume (PTV) by up to 9.9%. This can impair tumor control rates and treatment efficacy.
   - **Dose Distribution Alteration**: The presence of immobilization devices can alter dose distribution, potentially reducing dose to organs at risk (OARs) and increasing dose to normal tissues.

**Recommendations to Mitigate the Impact**

1. **Incorporate Devices in Treatment Planning**:
   - AAPM TG-176 recommends contouring and including immobilization devices and couch tops in treatment planning to account for their dosimetric effects. This ensures that the treatment planning system (TPS) accurately models the dose perturbations caused by these devices.
2. **Regular QA and Calibration**:
   - Regular quality assurance and calibration of treatment planning systems are essential to ensure accurate dose calculations.
3. **Use of Advanced Segmentation Techniques**:
   - For 3D-printed immobilization devices, advanced segmentation techniques can help accurately include these devices in dose calculations.
4. **Patient-Specific Adjustments**:
   - Adjust treatment plans based on the specific materials and configurations of immobilization devices used for each patient.

By following these recommendations, the impact of couch tops and immobilization devices on patient dose can be minimized, ensuring more accurate dose delivery and improved treatment outcomes.

Question12 (TG185)

Let’s think step by step. According to AAPM TG reports, in proton therapy, how to reduce the impact of high-atomic number materials?

RTPhy-ChatBot:

In proton therapy, high-atomic number materials can significantly affect the dose delivered to the prescription point and to tissue shielded by the prosthesis, leading to dramatic differences in treatment outcomes. To reduce the impact of high-atomic number materials, several strategies have been suggested:

1. Use lower-energy beams where possible: Lower-energy beams deposit more energy in the entrance channel and less energy in the distal part of the beam, reducing the amount of energy deposited in high-Z materials.
2. Avoid or minimize the use of high-photon and neutron activation cross section materials in accelerator components, treatment couch, and accessories: High-Z materials can produce significant amounts of photons and neutrons during proton therapy, which can contribute to dose buildup and increase the risk of secondary malignancies. By avoiding or minimizing their use, the impact of high-atomic number materials on the dose distribution can be reduced.
3. Restrict the use of high-energy treatment to the latter part of the day: High-energy treatments produce more neutrons and photons than low-energy treatments. By restricting the use of high-energy treatment to the latter part of the day, the buildup of activity throughout the day can be reduced, minimizing the impact of high-atomic number materials on personnel doses.
4. Consider delayed entry of staff for a few seconds or minutes following high-energy beam treatments: Delaying staff entry into the treatment room for a few seconds or minutes after high-energy beam treatments can help reduce their exposure to neutrons and photons, reducing the impact of high-atomic number materials on personnel doses.
5. Use specialized immobilization devices: Specialized immobilization devices can help mitigate range uncertainty due to immobilization materials, which can also contribute to dose inaccuracies. By using such devices, the impact of high-Z materials on the accuracy and precision of proton therapy can be minimized.
6. Consider alternative treatment techniques: Alternative treatment techniques, such as intensity-modulated proton therapy (IMPT) or passive scattering proton therapy with active scanning, can help reduce the impact of high-atomic number materials by delivering more conformal doses and minimizing dose deposition in healthy tissue.
7. Commission image guidance systems for proton therapy: Properly commissioning image guidance systems for proton therapy can help ensure accurate target localization and minimize the impact of high-Z materials on treatment accuracy and precision.
8. Develop recommendations to minimize the effects of uncertainties associated with proton RBE: Uncertainties in proton RBE can contribute to dose inaccuracies and uncertainty in treatment outcomes. By developing recommendations to minimize these uncertainties, the impact of high-atomic number materials on treatment accuracy and precision can be reduced.
9. Continuously monitor and improve treatment planning systems: Continuous monitoring and improvement of treatment planning systems can help ensure accurate dose calculations and minimize the impact of high-atomic number materials on treatment outcomes.
10. Conduct research to better understand the relationship between in vitro, in vivo, and clinical RBE: By conducting research to better understand the relationship between in vitro, in vivo, and clinical RBE, the impact of high-atomic number materials on treatment outcomes can be minimized and more accurate dose calculations can be made.

Gemini-2.0-flash:

While AAPM Task Group (TG) reports may not have a single report dedicated solely to mitigating the impact of high-atomic number materials in proton therapy, the issue is discussed within the context of treatment planning, dose calculation accuracy, and potential artifacts in imaging used for proton therapy. Based on the general understanding in the field and principles outlined in various TG reports (such as those concerning proton therapy commissioning, QA, and treatment planning), here's a breakdown of how to reduce the impact:

**Understanding the Challenges:**

High-atomic number (high-Z) materials, such as metallic implants (e.g., hip prostheses, dental fillings), can cause several issues in proton therapy:

- **Range Uncertainty:** Protons experience different stopping powers in high-Z materials compared to tissue. This can lead to significant uncertainties in the predicted proton range within and beyond the implant.
- **Dose Perturbations:** High-Z materials can cause dose enhancement at interfaces due to increased energy deposition from secondary electrons and backscatter. They can also cause dose reduction in the shadow of the implant due to attenuation and scattering of the primary proton beam.
- **Imaging Artifacts:** These materials can create significant artifacts in CT images, which are the primary input for treatment planning and range calculations in proton therapy. These artifacts can obscure the target volume and surrounding critical structures, leading to inaccurate planning.

**Strategies to Reduce the Impact:**

According to AAPM TG reports and best practices, the following strategies can be employed to mitigate the impact of high-Z materials in proton therapy:

**1. Improved Imaging and Artifact Reduction:**

- **Metal Artifact Reduction (MAR) Techniques:** Utilize CT imaging protocols and reconstruction algorithms specifically designed to reduce metal artifacts. Many modern CT scanners offer MAR software that can significantly improve image quality in the presence of metallic objects.
- **Dual-Energy CT:** This technique can sometimes help in differentiating materials based on their energy-dependent attenuation, potentially aiding in more accurate stopping power estimation even near high-Z materials.
- **Alternative Imaging Modalities:** In some cases, MRI or PET/CT might provide complementary information that is less susceptible to artifacts from metallic implants, aiding in target delineation.

**2. More Accurate Treatment Planning and Dose Calculation:**

- **Sophisticated Treatment Planning Systems (TPS):** Utilize TPS that have advanced algorithms for handling heterogeneous materials, including high-Z implants. These algorithms should account for the altered stopping powers and potential scattering effects.
- **Monte Carlo Simulations:** For complex cases with significant high-Z materials, Monte Carlo simulations can provide a more accurate prediction of dose distributions and range uncertainties compared to analytical algorithms. AAPM TG reports often recommend Monte Carlo as a benchmark for validating other dose calculation methods in challenging scenarios.
- **Density Override/Correction:** In the TPS, manually assigning or correcting the densities of the voxels containing the high-Z material based on known properties can improve range estimation. However, this requires careful consideration and may not fully account for scatter effects.
- **Worst-Case Scenario Planning and Robust Optimization:** Consider treatment plans that are robust against potential range uncertainties caused by the high-Z material. This might involve expanding target volumes or adjusting beam angles to minimize passage through or near the implant.

**3. Beam Angle Optimization:**

- **Avoiding Direct Beam Path Through Implants:** When possible, optimize beam angles to avoid having the primary proton beam pass directly through large volumes of high-Z material, especially near the target volume or critical structures.
- **Considering Implant Shadowing:** If a beam must pass through a high-Z material, understand the potential for dose reduction and adjust the plan accordingly to ensure adequate target coverage.

**4. In-Vivo Range Verification:**

- **Prompt Gamma Imaging:** Emerging techniques like prompt gamma imaging have the potential to provide real-time feedback on the proton range within the patient during treatment. This could help detect and potentially correct for range deviations caused by high-Z materials.
- **PET/CT Range Verification:** Using PET/CT imaging immediately after proton irradiation can provide information about the deposited activity, which is related to the proton range. This can be used to verify the accuracy of the планирование range in the presence of high-Z materials.

**5. Clinical Experience and Caution:**

- **Awareness of Potential Issues:** Clinicians and physicists should be aware of the potential dosimetric and imaging challenges posed by high-Z materials in proton therapy.
- **Careful Patient Evaluation:** Thoroughly evaluate the presence, size, and location of any high-Z materials during the planning process.
- **Conservative Margins:** In cases with significant range uncertainty, consider using slightly larger safety margins around the target volume, while carefully considering the proximity of critical structures.
- **Fractionation Considerations:** The impact of range uncertainties might be more pronounced over multiple fractions if the patient's anatomy or the implant position changes relative to the beam path.

In summary, while AAPM TG reports may not offer a specific "checklist" for reducing the impact of high-Z materials in proton therapy, the general principles emphasize accurate imaging with artifact reduction, sophisticated treatment planning that accounts for material heterogeneities, beam angle optimization, consideration of in-vivo range verification techniques, and a cautious clinical approach based on a thorough understanding of the potential challenges. The overarching goal is to minimize range uncertainties and dose perturbations to ensure accurate and safe proton therapy delivery for patients with high-Z implants.

GPT-4o:

Let's break down the **reduction of the impact of high-atomic number materials in proton therapy** step by step, according to AAPM Task Group (TG) reports, particularly **TG-158** and **TG-101**. These reports address the potential challenges posed by high-atomic number (high-Z) materials in proton therapy and how they affect dose distribution.

**✅ Step 1: Understanding the Problem of High-Z Materials in Proton Therapy**

In **proton therapy**, high-atomic number (high-Z) materials—such as metals (e.g., **titanium**, **stainless steel**) or other materials with a high **Z value**—pose several problems that can negatively impact the dose delivery and overall treatment outcome. The main issues are:

1. **Neutron Production**:
   High-Z materials can increase the production of **secondary neutrons** when protons interact with them. These neutrons can scatter and deliver unintended doses to nearby tissues.
2. **Increased Dose at the Surface**:
   High-Z materials can cause **dose enhancement** due to **secondary radiation production** (like **photo-neutrons**). This effect is more pronounced in proton therapy, where the dose distribution is already sensitive to material density variations.
3. **Disruption of the Bragg Peak**:
   The **Bragg peak** is where protons deposit the majority of their energy. High-Z materials can cause **scattering** of protons, which may **shift** the Bragg peak position or create a **dose tail** beyond the target area.

**❌ Step 2: Sources of High-Z Materials in Proton Therapy**

High-Z materials can be found in several components during proton therapy treatment:

1. **Prostheses or Implants**:
   Prostheses or metal implants (e.g., **hip replacements**, **dental fillings**, **surgical implants**) inside the patient's body can interact with the proton beam, leading to unwanted scattering or neutron production.
2. **Treatment Equipment**:
   The treatment machine itself may have high-Z materials in **beam-shaping devices** or **collimators**, as well as parts of the **gantry**, which can contribute to secondary radiation.
3. **Patient Positioning Devices**:
   Some patient immobilization devices and positioning equipment, like **headrests**, **braces**, or **cushions**, may contain high-Z materials that can affect the proton beam.

**⚖️ Step 3: Strategies to Mitigate the Impact of High-Z Materials**

AAPM TG-158 and TG-101 provide several strategies to reduce the impact of high-Z materials in proton therapy:

**1. Use of Low-Z Materials for Patient Positioning and Immobilization**

- **Minimize the use of high-Z materials** in the **patient immobilization devices**, **positioning devices**, and **beam-shaping components**. Low-Z materials, such as **carbon fiber**, should be preferred, as they cause less attenuation and scatter in the proton beam.
- Devices made from **low-density plastic** or **foam** materials are also preferred because they have less impact on the proton dose distribution compared to dense, high-Z materials.

**2. Implement Proton-Resistant Materials**

- When **metal implants** or **prostheses** are necessary, use materials that have a lower atomic number (e.g., **titanium**), as they produce fewer secondary neutrons compared to high-Z metals like **gold** or **platinum**.
- **Custom bolus materials** can be used for specific regions where high-Z materials cause issues, with the goal of **compensating for dose perturbations**.

**3. Advanced Treatment Planning Systems**

- Use advanced **treatment planning systems (TPS)** that take into account the **heterogeneous composition** of the patient, including the presence of high-Z materials, in order to **optimize the dose distribution**.
- **Monte Carlo simulations** or **ray-tracing algorithms** can be employed to model the effects of high-Z materials on proton beams more accurately and reduce the impact on the Bragg peak.
- Ensure **accurate tissue segmentation** in the treatment planning system to avoid miscalculating dose near high-Z regions and to help in the design of more precise proton beam angles and energy choices.

**4. Beam Modulation and Energy Variation**

- **Energy modulation** can help adjust the proton beam to reduce dose perturbations caused by high-Z materials. For instance, proton therapy typically utilizes **spread-out Bragg peaks (SOBPs)**, and energy variation can optimize the energy distribution to minimize side effects from high-Z materials.
- By modulating the proton beam and utilizing **multiple proton energy levels**, it’s possible to reduce the effect of high-Z material interactions on the dose distribution.

**5. Use of Shielding Materials**

- **Specialized shielding** (e.g., **lead** or **tungsten shields**) can be used to block secondary radiation, including neutrons, produced by high-Z materials. While this can reduce scattered dose in surrounding tissues, care must be taken to avoid introducing other dose perturbations.
- **Collimators and bolus materials** should be designed with **low-Z materials** to ensure that proton scattering is minimized while still effectively shaping the beam.

**✔️ Step 4: Quality Assurance and Ongoing Monitoring**

To ensure that the mitigation strategies are effective, AAPM TG-158 and TG-101 recommend implementing a robust **quality assurance (QA)** process:

1. **Check for Implant/Prosthesis Placement**:
   Prior to treatment, ensure that **metal implants** or **prostheses** are correctly accounted for in the treatment plan, and adjustments are made to mitigate the effects of neutron production and Bragg peak shifts.
2. **Regular QA on Treatment Equipment**:
   Ensure that proton therapy equipment, including **collimators** and **beam-shaping devices**, are regularly checked for the impact of high-Z materials. This will ensure that no excess secondary radiation is being produced during treatment.
3. **Patient-Specific QA**:
   For patients with high-Z materials, implement **patient-specific QA** using **CT imaging** or **imaging-based simulations** to account for material interactions and optimize dose delivery.

**🔑 Key Takeaways**

From AAPM **TG-158** and **TG-101**, to reduce the impact of high-atomic number (high-Z) materials in proton therapy:

- **Low-Z materials** should be used for **patient positioning** and **immobilization devices** to minimize the scattering and attenuation of proton beams.
- **Energy modulation** and **advanced treatment planning systems** (e.g., **Monte Carlo simulations**) help optimize dose distribution and minimize the effect of high-Z materials.
- **Shielding materials** can be used to reduce secondary radiation effects, but care must be taken not to introduce additional perturbations.
- **Regular QA** is essential to ensure that high-Z materials and their effects are properly accounted for in each patient’s treatment plan.

By following these strategies, the dose perturbation and unwanted side effects caused by high-Z materials in proton therapy can be effectively managed.

Moonshot-v1:

According to AAPM TG reports and related studies, reducing the impact of high-atomic number materials in proton therapy involves several strategies:

**Strategies to Reduce the Impact of High-Atomic Number Materials**

1. **Accurate Range Estimation**:
   - **Water-Equivalent Thickness (WET)**: Use WET measurements to account for the range shift caused by high-Z materials. This involves comparing the proton beam range in water with and without the high-Z material to determine the equivalent water thickness.
2. **Material Selection and Design**:
   - **Low-Z Materials**: Choose materials with lower atomic numbers for immobilization devices and other components to minimize dose perturbations.
   - **Boron Additives**: Incorporate boron into shielding materials to reduce the buildup of high-energy photons through the 10B(*n*,*α*)7Li reaction, which has a high cross-section for thermal neutrons.
3. **Advanced Imaging Techniques**:
   - **Dual-Energy CT (DECT)**: Use DECT to better estimate tissue properties and reduce proton range uncertainty. DECT can directly calculate effective atomic number (*Z*eff​) and electron density, improving the accuracy of proton range predictions.
4. **Optimized Treatment Planning**:
   - **Monte Carlo Simulations**: Use Monte Carlo methods to accurately model the interaction of protons with high-Z materials and optimize dose delivery.
   - **Robust Optimization**: Implement robust optimization techniques to account for uncertainties in range and dose distribution caused by high-Z materials.
5. **Shielding and Design Considerations**:
   - **Neutron Shielding**: For high-energy proton beams, consider additional shielding to mitigate neutron contamination, which can be a significant issue when high-Z materials are present.

**Practical Considerations**

- **Regular QA and Calibration**: Regular quality assurance and calibration of treatment planning systems are essential to ensure accurate dose calculations.
- **Patient-Specific Adjustments**: Tailor treatment plans to account for the specific materials and configurations used for each patient, ensuring accurate dose delivery.

By implementing these strategies, the impact of high-atomic number materials on proton therapy can be significantly reduced, ensuring more accurate dose delivery and improved treatment outcomes.

Question13 (TG203)

Let’s think step by step. According to AAPM TG reports, during radiotherapy, what is the maximum dose allowed for a pacemaker? During the treatment process, what should be paid attention to?

RTPhy-ChatBot:

According to the report of AAPM Task Group No. 34, the maximum dose allowed for a pacemaker during radiotherapy is 2 gray. However, it's important to note that this recommendation is based on available experimental and theoretical data, which suggests that there is a high risk to an individual patient but a low overall population risk. Therefore, precautions are suggested to minimize the risk of malfunction, while controlling the disease remains the priority in almost all cases.

During the treatment process, the following points should be paid attention to:

1. The radiation oncologist should consult with and dose to the part of the CIED body closest to the radiation and inform the treating cardiologist about the indications for field.
2. Absorbed dose to the pacemaker should be evaluated by an MP (Medical Physicist) and documented.
3. The patient should be informed about the small risks involved and requested to move out of the direct radiation field if possible. If placement within the field is unavoidable, all cardiologic symptoms encountered during his/her course of RT should be reported, and a consultation between the physicist, radiation oncologist, and cardiology department should engage in a consultation. The patient should be associated with the High-Risk category.
4. The quality of the treatment plan should not generally be compromised for the sake of sparing the device, as controlling the disease is more important than reducing the comparatively low risk of device failure.
5. Studies have dealt with linear accelerators, betatrons, and cobalt irradiators only. Use of other radiation therapy machines should be evaluated on an individual basis and approached with caution.
6. The most prudent and reasonable approach is to assess the maximum dose to any point in the device, as some areas may not be obvious and will vary between different devices. CIED leads do not need to be considered as part of the CIED, since they are highly resistant to therapy doses.
7. The precision of any dose assessment should be sufficient to ensure that the patient is properly categorized and therefore properly managed.

Gemini-2.0-flash:

According to AAPM Task Group (TG) reports, particularly **TG-34** and the more recent **TG-203 ("Management of Radiotherapy Patients with Implanted Cardiac Pacemakers and Defibrillators")**, there isn't a single "maximum allowed dose" for pacemakers that guarantees no malfunction. Instead, the reports provide recommendations based on the risk of device failure and functional changes.

Here's a breakdown of the key points:

**Maximum Dose Considerations:**

- **Functional Changes at Low Doses:** Contemporary cardiac pacemakers can exhibit functional changes at doses as low as **2 Gy (200 cGy)**. These changes might be transient or require reprogramming of the device.
- **Failure at Higher Doses:** Pacemaker failure due to radiation damage has been reported at cumulative doses as low as **10 Gy (1000 cGy)**, although there is variability between devices.
- **TG-203 Recommendations:** This report categorizes patient risk based on the estimated dose to the Cardiac Implantable Electronic Device (CIED), pacing dependency, and the presence of neutrons (typically from high-energy photon beams >10 MV or proton therapy). The risk categories guide the level of monitoring and intervention needed.
  - **Low Risk:** Pacing-independent patients receiving < 2 Gy with <10 MV photons.
  - **Medium Risk:** Pacing-independent patients receiving 2-5 Gy with <10 MV photons, or pacing-dependent patients receiving < 5 Gy with <10 MV photons.
  - **High Risk:** Patients receiving ≥ 5 Gy with <10 MV photons, or any dose with neutrons present.

**During the treatment process, what should be paid attention to:**

1. **Multidisciplinary Team Approach:** Effective management requires collaboration between the radiation oncologist, medical physicist, cardiologist or electrophysiologist, and the patient.
2. **Pre-treatment Assessment:**
   - **Device Information:** Identify the type and model of the pacemaker and the patient's dependency on it (pacing-dependent vs. pacing-independent). This information is crucial for risk assessment.
   - **Cardiology Consultation:** Consult with the patient's cardiologist or electrophysiologist to understand the device's settings, lower rate limit, and any specific concerns.
   - **Dose Estimation:** The medical physicist must estimate the dose to the pacemaker based on the treatment plan, including primary beam, scatter, and leakage. Treatment Planning Systems (TPS) can be used for this, but out-of-field doses might need separate measurement or calculation.
3. **Treatment Planning and Delivery:**
   - **Keep the Pacemaker Out of the Direct Beam:** This is the most crucial step. Plan the treatment to avoid direct irradiation of the pacemaker whenever clinically feasible.
   - **Minimize Scatter Dose:** Use techniques that reduce scatter to the device, such as lower energy beams (≤10 MV photons are generally considered non-neutron producing), beam collimation, and shielding if necessary.
   - **Monitor the Patient:** Observe the patient during treatment for any signs of pacemaker malfunction (e.g., dizziness, palpitations).
4. **During Treatment Monitoring (If Dose Exceeds Thresholds):**
   - **Functionality Checks:** If the estimated dose to the pacemaker exceeds 2 Gy (as per older guidelines and a point for increased vigilance in TG-203), additional functionality checks by a cardiac device specialist should be performed during and after the course of radiotherapy. The frequency and timing of these checks should be determined in consultation with the cardiologist.
   - **ECG Monitoring:** Continuous or intermittent ECG monitoring during treatment might be considered for high-risk patients or when the pacemaker is expected to receive a significant dose.
5. **Post-treatment Follow-up:**
   - **Device Evaluation:** A follow-up evaluation by the cardiologist or electrophysiologist is recommended after the completion of radiotherapy, especially if the estimated dose to the device was significant.
   - **Long-term Monitoring:** Patients should be educated about the potential for late-onset device malfunction and the importance of regular follow-up with their cardiologist.

**Key Takeaways from AAPM TG Reports:**

- The risk of pacemaker malfunction increases with the cumulative dose received by the device.
- Lower energy photon beams (<10 MV) are generally preferred to minimize neutron contamination.
- Keeping the pacemaker outside the direct treatment beam is paramount.
- Careful dose estimation to the device is essential.
- Collaboration with cardiology is crucial for appropriate patient management and device monitoring.

It's important to consult the latest AAPM TG reports (especially TG-203) for the most up-to-date and comprehensive recommendations on managing radiotherapy patients with pacemakers.

GPT-4o:

Let's break down the **maximum dose allowed for a pacemaker** during radiotherapy and the **important considerations** for managing this sensitive issue, based on AAPM Task Group (TG) reports, particularly **TG-34** and **TG-40**, which address radiation safety for medical devices such as pacemakers during radiotherapy.

**✅ Step 1: Understanding the Risk of Radiation to Pacemakers**

A **pacemaker** is an electronic device used to regulate the heart's rhythm. It is **sensitive to electromagnetic interference**, and **radiation** from radiotherapy can pose a risk to the device's functionality. There are concerns regarding both the **direct effects** of the radiation on the pacemaker and the potential **indirect effects** on the surrounding tissue.

- **Pacemakers can be affected by radiation** due to the radiation-induced damage to the electronic components inside the device.
- **Radiation exposure** can potentially lead to **malfunction** of the pacemaker or cause **irreversible damage** to its circuits, particularly with **high doses**.

**❌ Step 2: Maximum Dose Limits for Pacemakers**

According to **AAPM TG-34** and **TG-40**, the **maximum dose** that can be delivered to a pacemaker during radiotherapy is generally set to **2 Gy**. This is the **recommended dose limit** for pacemakers, as higher doses could potentially damage the device or interfere with its functioning.

However, this limit may vary based on the **pacemaker type**, **device manufacturer**, and **specific patient considerations**. It is important to note that the **pacemaker location** relative to the treatment field can significantly influence the dose it receives.

**⚠️ Step 3: Key Considerations During Treatment**

To minimize the risk of pacemaker malfunction during radiotherapy, several considerations must be taken into account:

**1. Placement of the Pacemaker**

- **Location relative to the treatment field**: The position of the pacemaker relative to the radiation field is crucial. If the pacemaker is in the **direct path** of the radiation beam, it is more likely to receive a higher dose, increasing the risk of damage.
- If the pacemaker is within the treatment field, efforts should be made to **avoid direct irradiation** of the device whenever possible, such as by adjusting the **beam angles**.

**2. Patient Positioning**

- **Ensure correct patient alignment**: Proper positioning can help minimize the direct exposure of the pacemaker to the radiation. Using **imaging systems** to verify patient alignment before treatment is essential to ensure the pacemaker remains outside the treatment area.
- If possible, **displace the pacemaker** from the treatment area (for example, by using **bolus material** to displace tissue away from the pacemaker).

**3. Treatment Planning Adjustments**

- **Use treatment planning systems** to carefully plan the radiation delivery, ensuring that the **pacemaker** is not exposed to doses higher than the recommended limit.
- Employ **shielding** techniques, such as using **lead shielding** or **custom shields**, around the pacemaker to reduce the dose to this sensitive device.

**4. Beam Modulation and Energy Selection**

- **Adjust the beam energy**: Lower-energy beams (e.g., **6 MV**) should be used when possible, as they tend to have a more localized dose distribution, which may help reduce dose to the pacemaker.
- When using **high-energy beams** or **IMRT**, special care should be taken to avoid over-irradiation of the pacemaker. The use of **modulated beams** can help better control the dose distribution.

**5. Monitoring Pacemaker Function**

- **Continuous monitoring**: During radiotherapy, the pacemaker's function should be regularly monitored. In some cases, the patient’s pacemaker may need to be reprogrammed or temporarily disabled to avoid any interference from the radiation.
- It is important to **coordinate with the cardiologist** or the physician responsible for managing the pacemaker, to ensure that the pacemaker settings are compatible with the planned radiotherapy.

**🔒 Step 4: Post-Treatment Considerations**

After the radiation treatment is completed, the following steps should be taken to ensure that the pacemaker is still functioning correctly:

1. **Monitor Pacemaker Function**: After radiotherapy, the **pacemaker’s functionality** should be closely monitored for any signs of malfunction or altered settings. This can involve:
   - **Electrophysiological tests** to verify that the pacemaker is working as expected.
   - A follow-up consultation with a cardiologist to check the pacemaker’s settings and overall performance.
2. **Check for Device Malfunction**: Signs of malfunction, such as **changes in heart rhythm**, should be immediately addressed. If any issues are detected, the pacemaker may need to be **reprogrammed** or even **replaced** if it has been compromised by radiation exposure.

**✔️ Step 5: Key Takeaways**

Based on the AAPM **TG-34** and **TG-40** reports, the **maximum allowable dose** to a pacemaker during radiotherapy is **2 Gy**. Several measures should be taken to ensure that this limit is not exceeded and that the pacemaker is protected:

- The **pacemaker’s location** should be carefully considered, and **beam angles** should be adjusted to avoid direct exposure.
- Proper **patient positioning** and **treatment planning** are essential to minimize radiation exposure to the pacemaker.
- If necessary, **shielding techniques** can be employed, and the **pacemaker’s function** should be monitored during and after treatment.
- **Collaboration with the cardiology team** is crucial to ensure pacemaker safety during radiotherapy.

By taking these steps, the risk of pacemaker malfunction can be minimized, ensuring the patient receives effective treatment without compromising the function of their pacemaker.

Moonshot-v1:

**Maximum Dose Allowed for a Pacemaker in Radiotherapy**

According to AAPM Task Group No. 34, the maximum dose allowed for a cardiac pacemaker during radiotherapy should be limited to **2 Gy**. This recommendation is based on studies showing that pacemakers can fail from radiation damage at doses as low as 10 Gy and can exhibit functional changes at doses as low as 2 Gy.

**Precautions During Treatment**

1. **Avoid Direct Beam Exposure**:
   - Pacemakers should not be placed in the direct (unshielded) therapy beam, as some accelerator beams can cause transient malfunction.
2. **Estimate and Monitor Dose**:
   - The absorbed dose to the pacemaker should be estimated before treatment. If the total estimated dose to the pacemaker might exceed 2 Gy, the pacemaker function should be checked prior to therapy and possibly at the start of each following week of therapy.
3. **Programming Considerations**:
   - If the average dose rate at the device is >1 cGy/min, the pacemaker should be programmed into asynchronous pacing mode.
4. **Beam Energy**:
   - The beam energy should be <10 MV to minimize the risk of neutron contamination, which can damage the pacemaker.
5. **Regular Monitoring**:
   - Close monitoring of the pacemaker function during the first treatment and subsequent treatments is recommended. Continuous electrocardiogram (ECG) monitoring during treatment may be considered in patients with implantable cardioverter-defibrillators (ICDs) who have had prior treatments for ventricular arrhythmias.
6. **Multidisciplinary Collaboration**:
   - A multidisciplinary team should assess each patient individually, considering factors such as pacemaker dependency, planned energy of the RT, and the absorbed dose to the device.

By following these guidelines and precautions, the risk of pacemaker malfunction during radiotherapy can be significantly reduced, ensuring patient safety and effective treatment outcomes.

Question14 (TG264)

Let’s think step by step. According to AAPM TG reports, what are the limitations of MLC tracking?

RTPhy-ChatBot:

MLC tracking is a technique used to improve the agreement between the planned and delivered dose distributions to the treatment target(s) and OARs in radiotherapy. It consists of three major components: (a) a real-time position monitoring system, (b) an MLC tracking algorithm, and (c) the MLC itself. However, there are several limitations to MLC tracking that need to be considered when implementing this technology clinically. Some of these limitations include:

1. The need for additional gating thresholds: Since MLC tracking only controls the MLC motor positions, other motion management strategies such as gating may still be required to ensure accurate treatment delivery. This means that additional gating thresholds may be needed to account for target motion outside the tracking volume or faster than the MLC response time.
2. The need for periodic motion prediction: In some cases, there may be a periodic component to the target motion that needs to be accounted for in real-time. This requires the use of motion prediction algorithms to anticipate future target positions and adjust the MLC accordingly.
3. The choice of adjacent leaves: The number and arrangement of adjacent leaves used in MLC tracking can affect the accuracy and efficiency of the technique. Careful consideration is needed to determine the optimal number and arrangement of leaves for a given treatment plan.
4. The fallback strategy in case of MLC tracking failure: In the event of MLC tracking failure, there needs to be a fallback strategy in place to ensure that treatment delivery continues without interruption. This may involve switching to another motion management strategy or manually adjusting the MLC positions.
5. Constraints on plan complexity: The use of MLC tracking may impose constraints on the complexity of treatment plans, particularly for cases with large target volumes or complex anatomical structures. Careful planning and adaptation may be required to ensure that the benefits of MLC tracking outweigh the limitations.
6. Compatibility with other motion management strategies: MLC tracking is not the only motion management strategy used in radiotherapy. It is important to consider how MLC tracking will interact with other motion management techniques, such as gating or breath hold devices, to ensure accurate and efficient treatment delivery.
7. Safe implementation considerations: The safe clinical implementation of MLC tracking requires careful consideration of various factors, including the compatibility of the position monitoring system with MLC tracking, the accuracy and uncertainty of the position monitoring signal, and the potential for unanticipated events or errors during treatment delivery.
8. Limitations specific to MLC tracking: Some limitations of MLC tracking are specific to this technique, such as the leaf width limitation and the potential for residual dose errors due to variable anatomy or target motion. Mitigation strategies for these limitations include the use of PRV concepts, gating thresholds, trade-offs between target and critical structures, robust beam angles, real-time positioning, and MRI-Linacs or 3D fluoroscopy. It is important to note that the specific limitations of MLC tracking may vary depending on the clinical site and patient characteristics. Regular quality assurance (QA) checks and retrospective analysis of treatment data are essential to ensure accurate and safe implementation of MLC tracking in radiotherapy.

Gemini-2.0-flash:

According to AAPM Task Group (TG) reports and the broader literature on motion management in radiation therapy, MLC (Multi-Leaf Collimator) tracking, where the MLCs move dynamically to follow the motion of the target, has several limitations:

**1. Mechanical Limitations of MLCs:**

- **Leaf Speed and Acceleration:** MLC leaves have maximum speeds and accelerations. For rapid or large-amplitude motion, the leaves may not be able to keep up with the target's movement, leading to undershooting or overshooting of the target volume. This can result in geometric miss and dose inaccuracies.
- **Leaf Travel Range:** MLC leaves have a finite travel range. For targets with large motion amplitudes, especially in the superior-inferior direction, the leaves might reach their physical limits, making it impossible to fully encompass the moving target throughout the entire respiratory cycle.
- **Leaf Positioning Accuracy and Repeatability:** While modern MLCs are highly accurate, there is still a small inherent positioning uncertainty and potential for mechanical lag or hysteresis. These small errors can accumulate and become significant, especially during continuous tracking.
- **Interleaf Leakage and Transmission:** MLCs are not perfectly opaque to radiation. There is some interleaf leakage (radiation passing between the leaves) and transmission (radiation passing through the leaves). During dynamic tracking, the leaves are constantly moving and changing positions, which can make it more challenging to accurately model and account for this leakage and transmission, potentially affecting the delivered dose distribution.

**2. Imaging and Tracking System Limitations:**

- **Latency in Motion Detection and System Response:** There is a time delay between detecting the target's motion (e.g., using external surrogates or real-time imaging) and the MLCs responding and adjusting their positions. This latency can lead to a mismatch between the target's actual position and the MLC aperture, especially for rapid or unpredictable motion.
- **Accuracy and Resolution of Motion Monitoring:** The accuracy and spatial/temporal resolution of the motion monitoring system (e.g., optical tracking, electromagnetic tracking, real-time imaging) directly impact the effectiveness of MLC tracking. If the motion is not tracked accurately or with sufficient speed, the MLCs will not be able to follow it precisely.
- **Correlation Between Surrogate and Target Motion:** When external surrogates (e.g., skin markers) are used to infer internal target motion, the correlation between the two may not be perfect or constant throughout the treatment. Changes in this correlation (e.g., due to patient breathing pattern changes) can lead to inaccuracies in MLC tracking.
- **Imaging Dose and Frequency:** Real-time imaging to track the target directly (marker-based or markerless) can increase the overall radiation dose to the patient. The frequency of imaging needs to be balanced with the need for accurate tracking.

**3. Treatment Planning and Delivery Complexity:**

- **Complex Treatment Planning:** Planning for MLC tracking treatments is more complex than for static field treatments. The TPS needs to account for the dynamic movement of the MLCs and the interplay between the moving beam aperture and the moving target.
- **Increased Monitor Units (MU) and Delivery Time:** MLC tracking can sometimes require more monitor units or longer delivery times compared to other motion management techniques. This can increase the risk of intrafractional motion and patient discomfort.
- **Quality Assurance (QA) Challenges:** QA for dynamic MLC tracking is more challenging than for static treatments. Specialized phantoms and measurement techniques are needed to verify the accuracy of the tracking system and the delivered dose distribution to the moving target.

**4. Clinical Limitations and Patient Factors:**

- **Patient Compliance and Breathing Pattern:** MLC tracking relies on the patient's ability to maintain a relatively consistent and predictable breathing pattern (if respiratory motion is being tracked). Irregular or unpredictable breathing can reduce the effectiveness of the technique.
- **Target Motion Characteristics:** MLC tracking is generally more suitable for targets with relatively smooth and periodic motion. Highly irregular or large-amplitude motion can be challenging to track effectively.
- **Anatomical Constraints:** The proximity of critical structures to the moving target needs to be carefully considered when planning MLC tracking to ensure adequate sparing while accounting for potential tracking inaccuracies.

In summary, while MLC tracking offers the potential for highly conformal dose delivery to moving targets, its effectiveness is limited by the mechanical capabilities of the MLCs, the accuracy and latency of the motion monitoring system, the complexity of treatment planning and QA, and patient-related factors. Careful consideration of these limitations is crucial for determining the suitability of MLC tracking for a given clinical situation and for implementing it safely and effectively. AAPM TG reports on motion management provide guidance on assessing these limitations and implementing appropriate QA procedures.

GPT-4o:

Let's break down the **limitations of MLC (multileaf collimator) tracking** in radiotherapy step by step, according to AAPM Task Group (TG) reports, particularly **TG-142** and **TG-191**.

**✅ Step 1: Understanding MLC Tracking**

**MLC tracking** refers to the ability of the **multileaf collimator** to move and adapt in real time to the patient’s anatomy or tumor position, typically in response to **motion** (such as during **respiratory motion** in lung or breast cancer treatment). It is used in conjunction with **image-guided radiotherapy (IGRT)** to ensure that the radiation beam is delivered precisely to the target volume, even as the target moves.

The **MLC** consists of multiple leaves that can move independently to shape the radiation field. **MLC tracking** aims to adjust these leaves dynamically, compensating for motion of the tumor or organ during treatment.

**❌ Step 2: Limitations of MLC Tracking**

According to AAPM **TG-142** and **TG-191**, there are several **limitations** of MLC tracking that must be considered during treatment planning and delivery:

**1. Limited Tracking Speed**

- **MLC leaves may not track fast enough**: The **speed of MLC leaves** can be insufficient to precisely follow the rapid movements of the tumor or target. For example, **respiratory motion** can occur at rates that exceed the MLC tracking system's ability to adjust in real time.
- **Resulting effect**: If the MLC cannot track the motion fast enough, the beam may miss the target, leading to **under-dosing** of the tumor or **over-dosing** of healthy tissue.

**2. Limited Accuracy with Complex Motion**

- **Complex or irregular motion**: Tumors may move in a **non-linear or unpredictable manner**, such as **spiral** or **rotational motion**. MLC tracking systems are generally designed to track motion in **linear directions** (i.e., along the axis of the leaves). Thus, they may not be effective for **complex tumor motion** that deviates from a simple pattern.
- **Resulting effect**: If the tumor moves in complex patterns, **MLC tracking systems may fail** to provide accurate leaf positions, leading to misalignment and suboptimal dose delivery.

**3. Limited Field of View**

- **Restricted imaging or tracking range**: MLC tracking systems rely on **imaging systems** (e.g., **kV imaging**, **CBCT**) to monitor tumor movement. The **field of view (FOV)** of these imaging systems can be limited, making it difficult to track large or extended tumors. If the target moves outside the imaged volume, the MLC system may **lose track of the target**, resulting in misalignment and potential under-dose or over-dose to the target.
- **Resulting effect**: The MLC may **fail to adapt** to tumor motion if the tumor moves outside the tracking system's FOV, leading to inaccurate dose delivery.

**4. Beam and Target Alignment**

- **Potential misalignment** between the MLC and the actual tumor position can occur due to the **time delay** between detecting the tumor position and adjusting the MLC leaves. Even slight delays in **MLC repositioning** can result in the radiation beam not matching the target's location, especially if the tumor is moving rapidly.
- **Resulting effect**: The misalignment can lead to **under-dosing** of the tumor and **over-dosing** of normal tissues, especially if the tumor is in a critical location.

**5. MLC Leaf Interactions**

- In some advanced techniques such as **stereotactic radiotherapy** or **VMAT (volumetric modulated arc therapy)**, the **interaction between leaves** during dynamic movements of the MLC can result in **leaf positioning errors**. This can cause the treatment to be delivered incorrectly, and the dose distribution may not be as expected.
- **Resulting effect**: The issue of **leaf interplay** can degrade the dose delivery, particularly in treatments involving small fields or complex beam shapes, leading to less precise targeting of the tumor.

**6. Synchronization with Respiratory Motion**

- **Respiratory motion synchronization**: Many MLC tracking systems are designed to work in conjunction with **respiratory gating** or **breath-hold techniques**. However, the synchronization between the MLC and respiratory motion can sometimes be imperfect. For example, **phase shifts** between the respiratory cycle and MLC leaf motion can occur.
- **Resulting effect**: If synchronization is imperfect, **misalignment** of the radiation beam relative to the moving tumor can occur, leading to **incorrect dosing**.

**7. Setup and Calibration Errors**

- **Errors in initial setup and calibration** can compromise the accuracy of MLC tracking. If the MLC system is not calibrated correctly or if there is **set-up uncertainty** (e.g., **patient positioning errors**), the system may not track the target as precisely as needed.
- **Resulting effect**: These errors can lead to **deviations** in the MLC leaf positions and **incorrect dose delivery**.

**⚖️ Step 3: Mitigating Limitations of MLC Tracking**

AAPM **TG-142** and **TG-191** also provide strategies to **mitigate** the limitations of MLC tracking:

1. **Enhancing MLC Speed and Responsiveness**:
   - To address **speed limitations**, use **faster MLC systems** or **adaptive radiation therapy** techniques that can adjust to motion more effectively.
2. **Advanced Imaging Techniques**:
   - Integrating **real-time imaging systems** (e.g., **cine-MRI**, **4D-CT**, **kV-CBCT**) can help track motion more accurately, even for complex or irregular motion patterns.
3. **Improving Synchronization**:
   - Use advanced **gating systems** or **breath-hold techniques** in combination with MLC tracking to **synchronize the MLC leaves** with tumor motion, reducing the risk of misalignment due to motion.
4. **Patient Positioning**:
   - Optimize **patient positioning** and use **custom immobilization devices** to minimize motion and reduce the need for extensive MLC tracking during treatment.
5. **Incorporating Beam Modulation**:
   - Use **beam-modulated techniques** such as **VMAT** or **IMRT**, where MLC tracking is used in conjunction with dose optimization techniques to enhance the overall treatment precision.
6. **Regular Quality Assurance (QA)**:
   - Perform **regular QA** of the MLC tracking system to ensure that the system is functioning within the required tolerances. This includes testing the speed, accuracy, and responsiveness of the MLC system to motion.

**✔️ Step 4: Key Takeaways**

Based on **AAPM TG-142** and **TG-191**, the **limitations of MLC tracking** in radiotherapy include:

1. **Limited speed** of the MLC system to track fast-moving targets.
2. **Inaccuracy with complex or irregular motion** of the tumor.
3. **Limited field of view** for imaging and tracking, which can cause loss of target position.
4. **Misalignment** due to delays in synchronization and beam tracking.
5. **Potential errors due to leaf interplay** in dynamic treatments like VMAT or stereotactic radiotherapy.
6. **Synchronization issues** with respiratory motion.

To mitigate these limitations, **advanced imaging systems**, **improved synchronization techniques**, and **regular QA protocols** are recommended to enhance the precision and effectiveness of MLC tracking.

Moonshot-v1:

According to AAPM TG reports and related studies, the limitations of MLC (multileaf collimator) tracking in radiotherapy include the following:

**1. Limited Leaf Speed and Acceleration**

- **Conventional MLCs**: Traditional MLCs typically have a maximum leaf speed of 3–4 cm/s and a maximum leaf acceleration of 50–70 cm/s². These speeds are often inadequate to track fast target motion, especially when the target moves perpendicular to the leaf direction.
- **Impact on Tracking**: The inability to keep up with fast target motion can lead to beam hold-offs, which reduce treatment efficiency.

**2. Geometric and Dosimetric Accuracy**

- **Leaf Width**: The width of the MLC leaves affects the dosimetric properties and target conformity. Narrower leaves (e.g., 3 mm) can improve target conformity but may increase the total number of monitor units required for the treatment.
- **Penumbra and Leakage**: MLCs can produce wider effective penumbra and increased radiation leakage compared to conventional shielding methods. This can affect dose distribution accuracy.

**3. Mechanical and Control Limitations**

- **Mechanical Backlash**: Conventional MLCs using rotary motors can experience mechanical backlash, which affects tracking accuracy.
- **Control System Latency**: The control system used to drive the MLC leaves can introduce latency, impacting the real-time tracking capability.

**4. Complex Motion Patterns**

- **Irregular Breathing**: MLC tracking may struggle with irregular breathing patterns, which can lead to larger position errors and reduced dosimetric accuracy.
- **Multidimensional Motion**: Tracking complex, multidimensional motion (e.g., rotational or deformable motion) is more challenging and may require more advanced MLC systems.

**5. Software and Algorithm Limitations**

- **Leaf Sequencing Algorithms**: Current algorithms for optimal leaf sequencing and moving average calculations have limitations in handling fast target motion and may not fully eliminate beam hold-offs.

**Solutions and Future Directions**

- **High-Speed MLCs**: Development of high-speed MLCs with linear motors can significantly improve leaf speed and acceleration, enabling better tracking of fast target motion.
- **Advanced Control Systems**: Implementing advanced control systems with lower latency and higher precision can enhance tracking accuracy.
- **Optimized Treatment Planning**: Using more sophisticated treatment planning algorithms and techniques (e.g., intensity-modulated radiotherapy, IMRT) can mitigate some of the limitations of MLC tracking.

By addressing these limitations, the accuracy and efficiency of MLC tracking in radiotherapy can be improved, leading to better treatment outcomes.

Question15 (TG302)

Let’s think step by step. According to AAPM TG reports reports, for deep-inspiration breath hold (DIBH) for breast radiotherapy treatment with bolus, what should we pay attention to when performing surface guided radiotherapy?

RTPhy-ChatBot:

To perform DIBH for breast radiotherapy treatment with bolus using surface guided radiotherapy (SGRT), the following considerations are essential:

1. CT Simulation: Acquire scans at FB and DIBH, create SGRT-friendly immobilization, and ensure a reproducible correlation between the surface displacement and the breath hold pattern.
2. Planning: Create accurate FB & DIBH body contours (DICOM), export plan and body contours to the SGRT system, and select ROIs and settings including presholds.
3. Positioning for Treatment: Fine-tune positioning with SGRT at DIBH, acquire new reference surface with bolus at DIBH, and confirm DIBH position at least once per week using X-ray based target localization (e.g., kV/MV ports) or another IGRT modality like MV, fluoroscopy, CBCT, or MRI.
4. Treatment: Monitor the DICOM DIBH surface during non-bolus fields and the new reference surface during bolus fields using SGRT. If motion exceeds tolerance, coach the patient to DIBH. If motion continues to exceed tolerance, return to "Positioning for Treatment."
5. Alternatively, the patient's position may be compared to the non-bolus reference surface using SGRT prior to bolus placement. Nonbolus fields could be treated first, as patients are expected to reproduce their breath hold to ≤3 mm on any single day.
6. Experience treating non-bolus patients will aid the treatment team in gaining confidence with treatment requiring bolus. Assess the impact of the bolus on surface tracking prior to clinical use.
7. To minimize patient discomfort and reduce fatigue, initially position the patient utilizing a FB surface for postural alignment of anatomy that moves independently of the target volume such as the chin, shoulder, and elbow.
8. The patient should be able to understand and perform a reproducible breath hold, maintain it for at least 10 seconds, and simultaneously operate the hand-held switch (if applicable).
9. Stability of internal anatomy during breath hold is also crucial; some patients may have continuous diaphragm motion even while believing they are holding their breath. Evaluate under fluoroscopy on a conventional simulator and perform a breath-hold CT scan with segmented 10s acquisitions to assess this.

By following these steps, you can effectively implement DIBH for breast radiotherapy treatment with bolus using surface guided radiotherapy.

Gemini-2.0-flash:

According to AAPM TG reports and best practices in surface guided radiotherapy (SGRT) for deep-inspiration breath hold (DIBH) breast radiotherapy with bolus, several key aspects require careful attention:

**1. Baseline Setup and Reproducibility of DIBH:**

- **Establish a Stable and Reproducible DIBH:** AAPM guidelines emphasize the importance of a consistent and reproducible DIBH throughout the treatment fractions. SGRT systems are highly sensitive to surface changes, so a stable breath-hold is crucial. Train the patient thoroughly on the DIBH technique and monitor their ability to achieve and hold it consistently during the initial setup and each treatment fraction.
- **Define and Record the DIBH Surface:** During the simulation or first treatment fraction, carefully define and record the patient's surface in the DIBH state using the SGRT system. This will serve as the reference surface for subsequent treatments. Ensure the recorded surface encompasses the entire treatment area, including the bolus.
- **Account for Bolus Placement:** The bolus should be in place and consistent during the reference surface acquisition for DIBH. Any variation in bolus placement between the reference and treatment fractions will lead to setup errors detected by SGRT.

**2. Monitoring and Verification of DIBH with Bolus:**

- **Real-time Monitoring:** SGRT systems provide continuous, real-time monitoring of the patient's surface during each breath-hold. Ensure the system is set up to track the relevant surface points, including areas covered by the bolus if possible (though the bolus itself might obscure the skin surface).
- **Tolerance Levels:** Establish strict tolerance levels for deviations from the reference DIBH surface. These tolerances should be smaller than those typically used for free-breathing treatments due to the increased precision aimed for with DIBH and the potential impact of bolus position.
- **Bolus Stability:** Pay close attention to the stability of the bolus during the breath-hold. Any slippage or movement of the bolus relative to the skin surface will be detected by SGRT as a change in the overall surface and could indicate a compromised setup. Consider methods to ensure the bolus remains securely in place.
- **Triggering and Beam Hold:** Integrate the SGRT system with the linac to allow for beam-on only when the patient's surface is within the pre-defined DIBH tolerance. Implement beam-hold functionality if the surface deviates beyond the acceptable limits during treatment.

**3. Impact of Bolus on SGRT Accuracy:**

- **Surface Changes Due to Bolus:** The presence of the bolus will alter the patient's surface geometry compared to without the bolus. The reference surface *must* be acquired with the bolus in place.
- **Potential for Bolus Movement:** As mentioned earlier, any movement of the bolus between fractions or during a breath-hold will be detected as a surface change by SGRT. Ensure robust bolus fixation techniques.
- **SGRT Tracking Over Bolus:** The SGRT system tracks the external skin surface. If the bolus completely obscures the skin in the treatment area, direct tracking of that specific skin region becomes impossible. In such cases, rely on tracking the surrounding skin surface that is not covered by the bolus, ensuring a stable relationship between the bolus position and the tracked surface.

**4. Workflow and Training:**

- **Standardized Procedures:** Develop clear and standardized procedures for DIBH with bolus and SGRT, including patient setup, reference surface acquisition, monitoring parameters, and actions to be taken in case of deviations.
- **Staff Training:** Ensure all staff involved (radiation therapists, physicists) are thoroughly trained on the specific workflow and the intricacies of using SGRT with DIBH and bolus.

**5. Quality Assurance (QA):**

- **Regular System QA:** Perform regular QA of the SGRT system according to AAPM guidelines to ensure its accuracy and reliability in tracking surface changes.
- **End-to-End Testing:** Conduct end-to-end tests that simulate the entire treatment process with DIBH and bolus, including surface acquisition, breath-hold monitoring, and beam delivery, to verify the overall accuracy and identify potential issues.

**In summary, when using SGRT for DIBH breast radiotherapy with bolus, the key attention points are:**

- **Consistent and reproducible DIBH with the bolus in place during reference surface acquisition.**
- **Real-time monitoring of the surface, including areas influenced by the bolus.**
- **Strict tolerance levels for surface deviations.**
- **Ensuring bolus stability throughout the treatment.**
- **Thorough patient and staff training on the specific workflow.**
- **Robust QA procedures to verify the entire process.**

By carefully addressing these aspects, you can leverage the benefits of SGRT for accurate and safe delivery of DIBH breast radiotherapy with bolus. Remember to consult specific AAPM TG reports on SGRT and motion management for detailed recommendations and tolerance levels relevant to your clinical practice.

GPT-4o:

Let’s break this down **step by step**, based on the guidance from **AAPM TG reports**, especially **AAPM TG-302** and **related literature on DIBH and surface guided radiotherapy (SGRT)**.

**🧠 Step 1: Understanding the Context**

- **Deep-Inspiration Breath Hold (DIBH)** is often used in **left-sided breast radiotherapy** to reduce the heart dose by increasing the distance between the heart and the chest wall.
- **Bolus** is applied over the treatment site to bring the dose closer to the skin surface.
- **Surface Guided Radiotherapy (SGRT)** uses optical surface imaging (e.g., VisionRT) to track the patient’s surface and help monitor and gate the beam during DIBH.

So we’re combining **DIBH**, **bolus**, and **SGRT**—each of which introduces its own considerations.

**✅ Step 2: Key Considerations When Using SGRT for DIBH with Bolus**

**1. Bolus Placement Affects Surface Tracking**

- **SGRT systems track the patient’s surface**, but **bolus changes that surface**.
- If the bolus is placed **after** the reference surface is acquired, it may be seen as a **mismatch** or **error**.
- If bolus is placed **before** the reference image, any **movement or air gaps** under the bolus during treatment can affect surface matching.

👉 **Recommendation**:

- Either **include the bolus** in the reference surface if you always treat with it in place, **or** define a region of interest (ROI) for tracking that **excludes the bolus area**.
- Always ensure **reproducible bolus placement**, with attention to **no air gaps**.

**2. ROI Selection Is Critical**

- The **tracking ROI** should avoid regions with large motion or deformation (like the breast itself or bolus-covered areas).
- The ROI should typically include **stable anatomical areas**, such as the **sternum**, **chest wall**, or **upper thorax**.

👉 **Recommendation**:

- Carefully define the ROI to focus on **rigid, reproducible areas** of the chest to minimize false-positive gating events.

**3. Breath Hold Reproducibility**

- Patients need to be **trained and coached** to consistently achieve and hold the same DIBH position.
- Even small variations in inspiration depth can cause dose differences, especially near the **heart** or **lung**.

👉 **Recommendation**:

- Use **visual feedback systems** for patients during SGRT to help guide consistent breath-holds.
- Ensure SGRT is used in **gated mode** to verify that radiation is only delivered when the patient is within a pre-set threshold of their reference DIBH position.

**4. Latency and System Response Time**

- There is a small **latency** between motion detection and beam-on/off response in SGRT systems.
- In DIBH, this may affect the accuracy of beam gating, particularly if the breath-hold is not steady.

👉 **Recommendation**:

- Configure a **tight but realistic gating window**, allowing for small fluctuations but avoiding false triggering.
- Perform QA to verify **system latency and gating accuracy**.

**5. Skin Dose and Dosimetric Impact of Bolus**

- Bolus increases **skin dose**, which may impact **surface alignment** due to possible **swelling**, **inflammation**, or **patient discomfort** over the treatment course.
- Daily changes in skin condition can affect surface tracking accuracy.

👉 **Recommendation**:

- Monitor skin changes over the treatment course, and **adjust the ROI or surface reference as needed**.
- Consider periodic re-verification or **mid-treatment re-scanning** if significant anatomical changes are expected.

**🧪 Step 3: Quality Assurance (QA)**

- **AAPM TG-302** and **TG-147** recommend **routine QA** of the SGRT system including:
  - **Daily checks** of camera calibration.
  - **Monthly end-to-end testing** for DIBH workflows (phantom-based testing with breath-hold simulation).
  - **Verification of bolus reproducibility** (physical marking or templates).

**📝 Summary – What to Pay Attention to:**

1. **Bolus must be placed consistently**, and tracked surface should either include it from the start or avoid it entirely in ROI.
2. **Define the ROI** on **rigid anatomical structures**, avoiding soft or bolus-covered areas.
3. **Train patients** for consistent DIBH, use visual feedback, and verify with gating.
4. **Manage system latency** and define a proper **gating threshold**.
5. **Monitor skin changes**, as they can affect surface tracking over time.
6. Perform **QA regularly**, especially on bolus placement, SGRT calibration, and breath-hold reproducibility.

Moonshot-v1:

When performing surface-guided radiotherapy (SGRT) for deep-inspiration breath hold (DIBH) in breast radiotherapy treatment with bolus, several important considerations and precautions should be taken to ensure accurate treatment delivery and patient safety, as outlined in AAPM TG-302 and related studies:

**Key Considerations and Precautions**

1. **Patient Setup and Positioning**:
   - **Two-Step Setup Procedure**: Use a two-step setup procedure to align the patient first in free-breathing (FB) and then transition to DIBH. This ensures minimal deformation and accurate positioning of the breast and local lymph nodes.
   - **Arm and Chin Alignment**: Ensure proper alignment of the arm and chin during the FB setup to minimize breast deformation and accurately reproduce the positions of local lymph nodes.
2. **Region of Interest (ROI) Definition**:
   - **Uniform ROI Definition**: Define a large ROI that covers the ipsilateral breast and part of the contralateral breast to ensure robust surface matching and minimize ambiguity. This ROI should include stable areas like the sternum and provide sufficient visible surface area for real-time monitoring.
3. **Monitoring and Adjustments**:
   - **Vertical Shift Monitoring**: Closely monitor the vertical shift of the surface from FB to DIBH, as this directly correlates with chest wall separation from the heart. The goal is to achieve minimal residual shift in the vertical direction.
   - **Real-Time Motion Monitoring**: Use real-time surface imaging to monitor patient motion during treatment. If motion exceeds a set tolerance (typically ±3 mm), the beam should be held until the patient returns to the correct position.
4. **Bolus Application**:
   - **Bolus Placement**: If a bolus is used, it should be applied quickly within a DIBH and a new reference surface captured immediately. This ensures accurate setup and motion monitoring regardless of bolus application.
   - **Bolus Material and Imaging**: Use bolus materials that can be effectively imaged by the optical surface imaging system. For example, sticky bolus with an outer white-cloth surface can be used for better imaging.
5. **Safety and Patient Comfort**:
   - **Breath-Hold Duration**: Ensure that patients can comfortably hold their breath for the required duration (typically around 20 seconds) without significant discomfort.
   - **Safety Mechanisms**: Equip the patient with a safety button to terminate the treatment cycle if they experience breathing difficulties. The treating physician should also monitor the patient’s breathing from the console.
6. **Technical Considerations**:
   - **Surface Imaging System**: Use advanced surface imaging systems like AlignRT, which provide 3D/4D surface guidance for both patient setup and DIBH gating. These systems can communicate with the linear accelerator to apply SGRT shifts and gate the radiation beam.
   - **Daily Reference Capture**: Always capture a new on-site reference surface after patient setup, regardless of whether bolus is used. This resets any residual setup uncertainties and simplifies clinical operations.

**Summary**

By following these guidelines, the accuracy and reproducibility of DIBH treatment can be significantly improved, ensuring optimal sparing of critical organs like the heart while delivering the prescribed dose to the target volume. Proper patient setup, ROI definition, real-time monitoring, and careful bolus application are crucial for successful SGRT in breast radiotherapy.

Questions16 (TG284)

Let’s think step by step. According to AAPM TG reports, in a monthly quality assurance test, what is the tolerance of laser marking accuracy of an MRI simulator?

RTPhy-ChatBot:

According to AAPM TG reports, the recommended tolerance for laser marking accuracy of an MRI simulator is ±2 mm. However, it is mentioned that with high precision laser localizer systems commercially available, a tolerance of ±1 mm should be achievable for both tests. Therefore, while the minimum tolerance is ±2 mm, it's recommended to aim for a higher accuracy if possible.

Gemini-2.0-flash:

While there isn't a specific AAPM Task Group report dedicated solely to the quality assurance of MRI simulators used in radiation therapy, several reports address aspects relevant to geometric accuracy and laser systems used for patient positioning. Based on these and general quality assurance principles, we can infer a reasonable tolerance for the laser marking accuracy of an MRI simulator in a monthly QA test.

Here's a step-by-step breakdown:

1. **AAPM TG-66: Quality assurance for computed-tomography simulators and the computed-tomography-simulation process:** Although focused on CT simulators, TG-66 provides valuable insights into geometric accuracy requirements for simulation. It recommends a tolerance of **±2 mm** for the spacing of lateral wall lasers with respect to lateral gantry lasers and the scan plane in monthly QA. This highlights the importance of laser accuracy for patient localization.
2. **AAPM TG-142: Task Group 142 report: Quality assurance of medical accelerators:** While primarily focused on linear accelerators, TG-142 also addresses the QA of imaging devices integrated with the linac, including CT on rails or cone-beam CT. For these systems, it recommends a monthly tolerance of **±2 mm** for the alignment of lasers with the center of the imaging plane and the spacing of room lasers relative to the isocenter.
3. **Extrapolation to MRI Simulators:** MRI simulators, like CT simulators, rely on laser systems for accurate patient positioning and marking isocenter locations on the patient's skin or immobilization devices. The geometric accuracy requirements for MRI simulation are comparable to those for CT simulation to ensure accurate treatment planning and delivery.
4. **General Principles of Geometric Accuracy in Radiotherapy:** Across various AAPM reports (e.g., TG-40, TG-105, TG-142), a general principle of maintaining geometric accuracy within a few millimeters is emphasized to avoid significant dosimetric errors.

**Therefore, based on the recommendations in AAPM TG-66 and TG-142 for laser accuracy in CT simulators and imaging systems associated with linacs, and considering the general principles of geometric accuracy in radiation therapy, a reasonable monthly tolerance for the laser marking accuracy of an MRI simulator would be ±2 mm.**

**In summary, according to the principles outlined in AAPM TG reports concerning geometric accuracy in simulation and imaging systems, a monthly quality assurance test for the laser marking accuracy of an MRI simulator should aim for a tolerance of ±2 mm.**

It is important to note that specific institutional protocols might adopt more stringent tolerances based on the clinical applications and the capabilities of the MRI simulator system. Always refer to your institution's QA procedures and consider any specific guidelines provided by the MRI simulator vendor.

GPT-4o:

Sure, let's go step by step.

1. **We're focusing on MRI simulators** — used in radiation therapy for treatment planning.
2. The relevant AAPM Task Group report is **AAPM TG-284**, which provides guidelines for **quality assurance (QA) of MRI simulators**.
3. For **monthly QA**, TG-284 includes checks on the **laser system**, which is used for patient positioning and marking the isocenter.
4. The **laser marking accuracy** refers to how well the lasers align with the **MRI scanner's imaging isocenter**.
5. According to **AAPM TG-284**, the **tolerance for laser marking accuracy** during **monthly QA** is **±2 mm**

This means the lasers should align with the imaging isocenter within 2 millimeters.

Moonshot-v1:

According to AAPM TG-284, the tolerance for laser marking accuracy in an MRI simulator during a monthly quality assurance test is **±2.0 mm**. This tolerance applies to the accuracy of laser markings in the axial, sagittal, and coronal planes.

Questions17 (TG256)

Let’s think step by step. According to AAPM TG reports, what are disadvantages of using a constant RBE in proton therapy?

RTPhy-ChatBot:

The disadvantages of using a constant RBE in proton therapy include the following:

1. Lack of justification for using a constant clinical RBE = 1.1 that is independent of (a/b)c, fraction size, and LETd within tumor targets and OAR based on our current understanding of the RBE for cell survival.
2. It is premature to adopt and recommend a variable RBE model to use clinically due to uncertainties in quantifying and modeling RBE effects in various tumor and normal tissues for various endpoints.
3. An uncertainty analysis cannot take into account RBE in a comprehensive manner due to the lack of knowledge of RBE values in humans for any endpoint or tissue, as well as the lack of information on how RBE values vary between individuals, populations, and other host factors.
4. The increase in RBE is currently considered only by avoiding the end of range, but it is not quantitatively incorporated in planning and evaluation of proton treatments. For intensity-modulated proton therapy (IMPT), the spot intensities and LETd distributions in the target are highly heterogeneous, so RBE may deviate significantly from 1.1 and vary considerably within an organ or tumor.
5. The use of a constant RBE may introduce noise and overestimate the uncertainty when performing a single analysis for tumors with increased RBE. A more appropriate way to report uncertainty would be to consider the percentage difference in RBE-weighted dose assuming a constant RBE of 1.1 or a spatially variant RBE using whatever biological model.
6. The lack of knowledge of RBE values and their uncertainties can make it difficult to optimize treatment plans, especially for complex cases where accurate dosimetry is essential. This can lead to suboptimal treatments or increased risk to critical structures.
7. The use of a constant RBE may not account for the potential differences in RBE between different tumor types and normal tissues, which could impact treatment outcomes and patient safety.
8. The lack of consensus on the definition and measurement of RBE can make it challenging to compare data across studies and institutions, making it difficult to establish best practices and guidelines for clinical use.

Gemini-2.0-flash:

According to AAPM TG reports and the broader literature on proton therapy, using a constant Relative Biological Effectiveness (RBE) of 1.1 has several disadvantages:

Here's a step-by-step breakdown:

1. **RBE is not a constant:** The fundamental limitation is that the RBE of protons is not a fixed value but varies depending on several factors. AAPM TG-256 specifically addresses the RBE of proton beams and acknowledges this variability. These factors include:
   - **Linear Energy Transfer (LET):** RBE generally increases with increasing LET. LET is not constant throughout the proton beam path; it increases towards the distal edge (Bragg peak).
   - **Dose per fraction:** Experimental data suggests that RBE can increase with decreasing dose per fraction.
   - **Tissue type:** Different tissues and tumor types exhibit varying radiosensitivities, leading to different RBE values for the same physical dose and LET.
   - **Biological endpoint:** The RBE can vary depending on the biological effect being considered (e.g., cell killing, DNA damage, chromosomal aberrations).
   - **Oxygenation status:** Hypoxic tumor regions can exhibit different RBE compared to well-oxygenated tissues.
2. **Potential for under- or over-dosage:** Assuming a constant RBE of 1.1 can lead to inaccuracies in the delivered biological dose:
   - **Under-dosage:** In tumors or tissues where the actual RBE is higher than 1.1, using a constant 1.1 could result in delivering a biologically lower dose than intended, potentially compromising tumor control. This might be particularly relevant for certain tumor types with low α/β ratios.
   - **Over-dosage:** Conversely, in regions where the RBE is lower than 1.1, a constant value could lead to delivering a biologically higher dose, increasing the risk of normal tissue toxicities. This is a significant concern at the distal edge of the Bragg peak where LET increases, potentially leading to unexpected damage in adjacent critical structures.
3. **Ignoring LET variations:** Using a constant RBE neglects the well-established increase in LET towards the end of the proton range. This higher LET region is associated with increased biological effectiveness, and failing to account for this can lead to unpredictable biological effects in the target volume and surrounding normal tissues.
4. **Suboptimal treatment planning:** Treatment plans based on a constant RBE might not fully exploit the potential benefits of proton therapy, such as enhanced tumor control in specific regions of the target volume due to higher LET. It also hinders the ability to optimize plans to minimize the risk of complications related to increased RBE in critical normal tissues.
5. **Difficulties in comparing clinical outcomes:** Clinical trials comparing proton and photon therapy that rely on a constant RBE of 1.1 might be difficult to interpret accurately if the actual RBE varies significantly across patients and treatment sites. This can obscure the true clinical benefits or risks associated with proton therapy.
6. **Limitations in radiobiological modeling:** A constant RBE does not align with current radiobiological understanding, which emphasizes the complex interplay between radiation quality (LET), dose, and biological systems. This simplification limits the ability to develop and utilize more sophisticated and potentially more accurate treatment planning strategies based on variable RBE models.

**In conclusion, while the use of a constant RBE of 1.1 has been a pragmatic approach in the early days of clinical proton therapy, AAPM TG reports (like TG-256) and the evolving body of research highlight the significant disadvantages of this simplification. Accounting for RBE variability is crucial for improving the accuracy, safety, and efficacy of proton therapy.** The field is moving towards incorporating variable RBE models into treatment planning as our understanding of the underlying radiobiology and the tools for implementation advance.

GPT-4o:

According to AAPM Task Group reports such as TG-256 and TG-185, using a constant RBE of 1.1 in proton therapy has several disadvantages.

First, it oversimplifies the biological effect of proton radiation. In reality, RBE varies based on factors like linear energy transfer (LET), tissue type, dose per fraction, and the biological endpoint being considered. A constant value does not reflect this variability.

Second, it can lead to an underestimation of the dose to normal tissues. Near the end of the proton range, where LET is high, the actual RBE may be greater than 1.1. Assuming a lower, fixed RBE can result in underestimating the biological dose to nearby organs at risk, potentially increasing the risk of side effects.

Third, it may overestimate the dose to the tumor. In low-LET regions such as the entrance of the proton beam, the actual RBE might be less than 1.1. Using a fixed value could result in overestimating the effectiveness of the dose, possibly reducing tumor control.

Fourth, it limits the ability to use advanced treatment planning techniques. As proton therapy becomes more biologically guided, a constant RBE restricts the ability to use LET-based or variable RBE optimization, which could improve treatment accuracy.

Finally, it introduces uncertainty in clinical outcomes. Some unexpected side effects in patients may be related to biological dose variations that are not captured when using a fixed RBE value.

In summary, while a constant RBE of 1.1 is convenient for clinical use, it can lead to inaccuracies in both tumor dosing and protection of healthy tissue.

Moonshot-v1:

According to AAPM TG-256 and related studies, using a constant relative biological effectiveness (RBE) in proton therapy has several significant disadvantages:

1. **Suboptimal Treatment Plans**:
   - Using a constant RBE may lead to suboptimal treatment plans that do not fully exploit the potential of proton therapy. This can result in less effective dose delivery to the target volume and potentially higher doses to normal tissues.
2. **Inaccurate Dose Distribution**:
   - The assumption of a fixed RBE does not account for the spatial variations in RBE within the target volume and surrounding tissues. This can lead to inaccuracies in the dose distribution, potentially resulting in under- or overdosing of critical structures.
3. **Increased Toxicity and Reduced Efficacy**:
   - Ignoring the spatial and dosimetric variations in RBE can lead to increased toxicity in normal tissues and reduced efficacy in tumor control. This is particularly concerning for tissues with known low α/β ratios.
4. **Complexity in Treatment Planning**:
   - Modern proton therapy techniques, such as pencil beam scanning (PBS), produce more complex dose distributions compared to passive scattering. The constant RBE assumption may not accurately reflect the biological effect of these complex dose distributions.
5. **Misleading Interpretation of Outcome Data**:
   - When tissue- and spatially variant RBE variations are neglected, the interpretation of treatment outcome data can be misleading. This can affect the assessment of treatment efficacy and the incidence of side effects.

**Recommendations**

- **Variable RBE Models**: There is a need to develop and implement variable RBE models that account for dose, linear energy transfer (LET), and tissue-specific biological effects. This can improve the accuracy of dose planning and delivery.
- **Clinical Data Collection**: It is important to collect clinical data to reconstruct RBE doses and correlate them with clinical outcomes. This will help in understanding the true impact of RBE variations on treatment efficacy and toxicity.
- **Advanced Treatment Planning Systems**: Treatment planning systems should be updated to incorporate variable RBE models and LET distributions. This will allow for more accurate biological dose optimization.
- **Robust Planning Techniques**: Robust planning techniques should be used to account for uncertainties in RBE and LET. This can help in creating treatment plans that are less sensitive to these variations.

In summary, while the use of a constant RBE simplifies treatment planning, it may not fully reflect the biological effects of proton therapy. Implementing variable RBE models and collecting more clinical data are essential steps to improve the accuracy and efficacy of proton therapy.

Questions18 (TG238)

Let’s think step by step. According to AAPM TG reports, what is the difference between analytic calibration model and pose determination model for a C-arm CBCT system?

RTPhy-ChatBot:

The geometric calibration of a C-arm CBCT system is essential to ensure accurate image reconstruction and localization of anatomical structures within the acquired data. Two primary approaches are used for geometric calibration: analytic calibration model and pose determination model.

The analytic calibration model involves mathematically modeling all the system parameters required for image reconstruction and pose determination. This approach provides a precise description of the system's geometry, including intrinsic and extrinsic parameters. The analytic model can be derived from the physical design of the C-arm CBCT system and is often used in simulation studies or when the actual calibration phantom is not available.

On the other hand, the pose determination model relies on estimating the system parameters from projection matrices, which describe the relationship between the X-ray source and detector positions for each view. This approach can be less stable due to challenges in accurately determining the source-to-detector distance, especially when dealing with small perturbations or detector rotations around the piercing point. However, it is more commonly used in clinical settings because it does not require a specific calibration phantom and can be performed quickly during routine system checks.

The choice between analytic and pose determination models depends on the specific application and available resources. The analytic model offers greater precision but requires more complex calculations and access to detailed system information, while the pose determination model is faster and simpler but may have lower accuracy due to the challenges in estimating certain parameters from projection data alone.

Gemini-2.0-flash:

While AAPM Task Group (TG) reports may not explicitly define "analytic calibration model" and "pose determination model" as distinct, universally labeled categories for C-arm CBCT systems, we can infer the differences based on the principles and procedures discussed in reports like **TG-238: 3D C-arms with volumetric imaging capability** and general CBCT calibration practices.

Here's a step-by-step understanding of the likely differences:

**1. Analytic Calibration Model:**

- **Focus:** This type of model primarily deals with determining and correcting the **intrinsic geometric parameters** of the C-arm CBCT system. These parameters are inherent to the hardware and ideally should be constant over time, although they can drift due to mechanical instabilities or thermal effects.
- **Parameters Determined:** An analytic calibration model aims to accurately quantify parameters such as:
  - **Source-to-detector distance (SDD):** The distance between the X-ray source and the detector.
  - **Detector pixel size and geometry:** The physical dimensions and arrangement of the detector elements.
  - **Projection center (principal point):** The point where the central ray of the X-ray beam ideally intersects the detector.
  - **Detector skew and tilt:** Any deviations of the detector plane from the ideal orientation.
  - **X-ray source focal spot position:** The effective location of the X-ray source.
  - **System isocenter:** The theoretical point of intersection of the X-ray beam's central ray with the axis of rotation at a specific C-arm angle.
- **Methodology:** Analytic calibration typically involves scanning a **geometric calibration phantom** with precisely known marker positions (e.g., a grid of beads or pins). The projections of these markers are then analyzed using mathematical algorithms to solve for the intrinsic geometric parameters. These algorithms often rely on geometric relationships and optimization techniques.
- **Outcome:** The output of an analytic calibration model is a set of fixed parameters that describe the system's geometry. These parameters are then used in the reconstruction algorithms to produce accurate 3D images by correcting for geometric distortions introduced during the acquisition process.

**2. Pose Determination Model:**

- **Focus:** This type of model is concerned with determining the **extrinsic parameters** of the C-arm CBCT system, specifically its **position and orientation (pose)** *relative to a fixed coordinate system*, often the patient or a treatment isocenter. This is particularly crucial for image-guided radiation therapy (IGRT) applications where the CBCT image needs to be accurately registered with the treatment planning CT or the patient's current position.
- **Parameters Determined:** A pose determination model aims to quantify:
  - **Translation:** The displacement of the C-arm isocenter (or a reference point on the C-arm) relative to the fixed coordinate system (e.g., in x, y, and z directions).
  - **Rotation:** The angular orientation of the C-arm around its rotational axes relative to the fixed coordinate system (e.g., roll, pitch, and yaw).
- **Methodology:** Pose determination often involves:
  - **External Tracking Systems:** Using optical or electromagnetic tracking systems to monitor the position and orientation of markers attached to the C-arm.
  - **Image-Based Registration:** Analyzing the CBCT images themselves to align them with a reference image (e.g., a planning CT) or with known anatomical landmarks. This might involve manual or automated registration algorithms that optimize the transformation (translation and rotation) between the two datasets.
  - **Calibration Objects in the Field of View:** Scanning a phantom with known spatial relationships to the external coordinate system. Analyzing the phantom's projection in the CBCT images allows for the determination of the C-arm's pose.
- **Outcome:** The output of a pose determination model is a transformation matrix or a set of translation and rotation parameters that define the spatial relationship between the C-arm CBCT system and the external reference frame. This information is vital for accurately aligning the patient for treatment based on the CBCT image.

**In summary, the key differences are:**

- **Analytic Calibration Model:** Focuses on the **internal geometry** of the C-arm CBCT system itself and aims to determine fixed geometric parameters for accurate image reconstruction.
- **Pose Determination Model:** Focuses on the **external position and orientation** of the C-arm CBCT system relative to a fixed coordinate system, crucial for spatial registration and image guidance.

While these models address different aspects, they are often interconnected. An accurate analytic calibration is a prerequisite for reliable pose determination, especially when image-based registration techniques are employed. The geometric accuracy of the reconstructed CBCT image directly impacts the accuracy of its alignment with other datasets or the patient's physical position.

GPT-4o:

Let's go step by step to understand the difference between the **analytic calibration model** and the **pose determination model** for a C-arm CBCT system, based on AAPM Task Group reports such as TG-179.

Step 1: Why is geometric calibration needed?

Geometric calibration ensures that the CBCT system knows exactly where the X-ray source and detector are located at each angle during image acquisition. This is critical for reconstructing accurate 3D images.

Step 2: What is the analytic calibration model?

The analytic calibration model assumes that the motion of the X-ray source and detector follows an ideal or known geometric path, usually a circular or elliptical trajectory. It uses a small number of fixed parameters, such as the source-to-axis and source-to-detector distances. The system is calibrated by fitting these parameters to match expected geometry. This method is simple and fast but assumes that the system behaves ideally.

Step 3: What is the pose determination model?

The pose determination model does not assume an ideal path. Instead, it determines the actual position and orientation (or "pose") of the X-ray source and detector for each projection image by analyzing images of a calibration phantom with known marker positions. This model captures system imperfections, such as gantry flex or mechanical sag, by using image data to calculate the true geometry for every angle.

Step 4: What is the main difference?

The analytic model assumes ideal geometry and uses a fixed set of parameters to define system motion. The pose determination model measures the actual geometry directly from image data, allowing it to correct for non-ideal mechanical behavior.

In summary, the analytic model is faster and simpler but assumes ideal conditions, while the pose determination model is more accurate because it accounts for real-world deviations in system geometry.

Moonshot-v1:

According to AAPM TG-238, the differences between the analytic calibration model and the pose determination model for a C-arm CBCT system are as follows:

**Analytic Calibration Model**

- **Description**: This model aims to describe the geometry of the imaging system exactly by providing a numerical value for all defined system parameters. It involves complex geometric descriptions relating the 3D position of a physical phantom to the measured image of the phantom at the detector.
- **Advantages**:
  - Provides an exact description of the system geometry.
  - Can be used for precise calibration when all parameters are accurately estimated.
- **Challenges**:
  - The results can be unstable due to challenges in estimating certain parameters, such as the source-to-detector distance, from projection matrices.
  - Requires a detailed understanding of the system's geometric relationships and often involves complex mathematical formulations.
  - The calibration process can be time-consuming and may require specific calibration phantoms with precise marker configurations.

**Pose Determination Model**

- **Description**: This model is based on the pinhole camera model and describes the system parameters in a calibration matrix containing both extrinsic and intrinsic parameters. It focuses on determining the pose (position and orientation) of the C-arm system relative to a reference coordinate frame.
- **Advantages**:
  - Simplifies the calibration process by using a linear transformation of homogeneous coordinates to model the relationship between 3D and 2D coordinates.
  - Allows for efficient optimization of the projection matrix, which minimizes the error between known 3D calibration objects and acquired projection images.
  - Can be more robust in handling variations in the system's pose and is widely used in practice.
- **Challenges**:
  - May not provide as detailed a description of the system geometry as the analytic model.
  - The accuracy of the pose determination depends on the quality of the calibration data and the optimization process.
  - Requires a good initial estimate of the system's pose to achieve accurate results.

**Summary**

- The **analytic calibration model** provides a detailed and exact description of the system geometry but can be complex and challenging to implement accurately.
- The **pose determination model** offers a more simplified and robust approach to calibration, focusing on optimizing the system's pose relative to a reference frame.

Questions19 (TG191)

Let’s think step by step. According to AAPM TG reports, what should be paid attention to when using TLDs or OSLs in the measurement of skin dosimetry?

RTPhy-ChatBot:

When measuring skin dosimetry with Thermoluminescent Dosimeters (TLDs) or Optically Stimulated Luminescent Dosimeters (OSLDs), there are several considerations to keep in mind.

First, it is important to note that the accurate measurement of dose to the skin is a serious challenge due to the very thin depth of only 0.07 mm recommended for the most superficial skin layers. This greatly limits the detectors that can be used for these measurements.

Second, TLDs have been demonstrated to measure skin doses or surface doses, but they typically require extrapolation due to their relatively thick size compared to the dose gradient. The high density of TLDs means that the effective point of measurement is downstream from the middle of the detector, which can result in overestimation of the surface dose by 10-40%. One technique for overcoming this is to make measurements with detectors of different thicknesses and extrapolate to the desired depth. However, Dot OSLDs have an effective point of measurement that is 0.8 mm deep, which also substantially overestimates the surface/skin dose.

Third, when reporting TLD or OSLD results for skin dosimetry, it is recommended to include specific details such as the detector material and manufacturer, readout methodology, calibration information, and uncertainty estimates. This will ensure that the data are accurate and can be reliably compared to other measurements.

Fourth, caution must be used when measuring out-of-field doses with TLDs in high-energy photon beams due to the potential for significant errors. The TLD-700 is recommended for use in these cases as it has a very low thermal neutron cross section and will accurately measure only photon doses.

Fifth, when using TLDs or OSLDs for skin dosimetry, it is important to follow the guidelines outlined in AAPM TG reports such as TG-191, which provide detailed methodologies for practical and optimal luminescence dosimetry in a clinical setting. These reports also include consensus guidelines on the optimal use of luminescent dosimeters for clinical practice and special medically relevant uses such as mixed photon/neutron field dosimetry, particle beam dosimetry, and skin dosimetry.

Sixth, it is important to note that in vivo measurements with TLDs or OSLDs require caution due to the necessary high voltage. Additionally, when using TLDs for out-of-field measurements in high-energy photon beams, correction factors may be required for soft beams to account for the overresponse of less than 10%.

Overall, careful consideration and attention to detail are essential when using TLDs or OSLDs for skin dosimetry to ensure accurate and reliable measurements.

Gemini-2.0-flash:

Let's break down the key considerations from AAPM Task Group (TG) reports for using Thermoluminescent Dosimeters (TLDs) and Optically Stimulated Luminescence Dosimeters (OSLs) in skin dosimetry measurements, step by step. While **AAPM TG-191** remains the most directly relevant report ("Clinical Use of Luminescent Dosimeters: TLDs and OSLDs"), other TGs discussing surface dose measurements or detector characteristics might also offer valuable insights.

Here's a step-by-step breakdown of what these reports emphasize:

**1. Detector Selection and Characteristics:**

- **Thinness is Crucial:** AAPM reports consistently highlight the need for **thin detectors** (e.g., thin discs or chips) to accurately measure skin dose. This minimizes volume averaging effects and ensures the measurement reflects the dose at or very near the skin surface (typically within the first few hundred micrometers). Thicker detectors can average the dose over a larger volume, potentially underestimating the surface dose, especially in regions with steep dose gradients (like buildup regions).
- **Material and Sensitivity:** Choose TLD or OSL materials with appropriate sensitivity for the expected dose range in skin dosimetry. Consider the energy response characteristics of the chosen material and ensure proper energy correction factors are applied during analysis.
- **Reproducibility and Batch Homogeneity:** Select detectors from batches with well-characterized and consistent response. Calibrate each batch thoroughly to account for inter-detector variations in sensitivity.

**2. Calibration and Energy Dependence:**

- **Energy Calibration:** Skin dose measurements often involve low-energy scattered radiation. Therefore, it's critical to calibrate the TLDs/OSLs using radiation beams with energies relevant to the clinical scenario. Using calibration factors derived from high-energy photon beams might lead to significant errors. AAPM reports emphasize the importance of **energy dependence corrections**.
- **Geometry Dependence:** Calibration should ideally be performed in a geometry that closely mimics the measurement conditions on the patient's skin, including the presence of backing materials or bolus if used clinically.
- **Traceability:** Calibration should be traceable to a national or international standard dosimetry laboratory.

**3. Placement and Contact with the Skin:**

- **Intimate Skin Contact:** Ensure **firm and direct contact** between the detector and the skin surface. Any air gaps can significantly alter the measured dose due to changes in electron backscatter and attenuation.
- **Securement:** The dosimeter must be securely attached to the skin to prevent movement during the radiation delivery. Use hypoallergenic tape or dressings that do not significantly attenuate the radiation beam. Document the exact location of each dosimeter.
- **Contouring to Skin Surface:** On curved anatomical regions, use flexible dosimeters or multiple small detectors to conform to the skin surface and avoid air gaps or inaccurate readings due to detector tilt.

**4. Environmental Factors and Handling:**

- **Temperature and Humidity:** TLD and OSL materials can be sensitive to environmental conditions. Follow the manufacturer's recommendations for storage, handling, and exposure conditions to minimize fading (signal loss over time) or spurious signals.
- **Light Sensitivity (OSLs):** OSL dosimeters are particularly sensitive to light. Handle and store them in light-tight containers before and after irradiation to prevent unwanted stimulation and signal loss.
- **Pre- and Post-Irradiation Handling:** Implement consistent handling procedures to minimize variability and potential contamination of the detectors.

**5. Measurement and Analysis:**

- **Reader Calibration and QA:** Ensure the TLD or OSL reader is properly calibrated and maintained. Regular quality assurance checks of the reader are essential for reliable measurements.
- **Background Subtraction:** Account for any background signal or zero-dose reading of the dosimeters.
- **Annealing and Re-use (TLDs):** For TLDs that can be re-used, follow established annealing protocols to ensure the complete removal of residual signals from previous irradiations. Inconsistent annealing can lead to inaccurate readings.
- **Multiple Readings (OSLs):** If partial readout is performed for OSLs, be aware of potential signal depletion and follow manufacturer guidelines for accurate analysis.

**6. Documentation and Uncertainty:**

- **Detailed Records:** Maintain thorough records of detector identification, calibration factors, irradiation conditions (energy, location, date, time), readout values, and any applied correction factors.
- **Uncertainty Analysis:** Perform a comprehensive uncertainty analysis that considers all potential sources of error, including calibration uncertainty, energy dependence correction, reader precision, detector placement variability, and environmental factors. Report the measured dose with an associated uncertainty.

**In essence, AAPM TG reports emphasize meticulous attention to detail in all aspects of using TLDs and OSLs for skin dosimetry. This includes selecting appropriate thin detectors, performing accurate energy-dependent calibration, ensuring intimate skin contact during measurement, controlling environmental factors, using calibrated and well-maintained readers, and rigorously analyzing the data with a thorough assessment of uncertainties.** The goal is to obtain a reliable and accurate estimate of the radiation dose delivered to the patient's skin. Always consult the specific recommendations within the relevant AAPM TG reports and the guidelines provided by the dosimeter manufacturer.

GPT-4o:

Let’s go step by step to understand what should be paid attention to when using **TLDs (thermoluminescent dosimeters)** or **OSLs (optically stimulated luminescent dosimeters)** for **skin dosimetry**, based on guidance from AAPM Task Group reports such as **TG-190** and **TG-158**.

**Step 1: Purpose of skin dosimetry**
Skin dosimetry is used to measure the dose received by the surface or near-surface layers of the patient’s skin, which is especially important in radiation therapy where skin toxicity can be a concern.

**Step 2: Why use TLDs or OSLs?**
TLDs and OSLs are small, passive detectors that can be placed on the skin to measure the dose delivered during treatment. They are commonly used because of their small size and ability to measure dose without requiring active electronics during exposure.

**Step 3: What should be paid attention to?**

1. **Energy dependence**
   TLDs and OSLs can respond differently depending on the energy of the radiation. Since the energy spectrum near the skin includes low-energy scatter and contaminant electrons, calibration must be done carefully to match these conditions.
2. **Angular dependence**
   The response of these dosimeters can vary based on the angle at which radiation hits them. This is important for skin dosimetry, as beams often enter at oblique angles, especially in complex treatment setups.
3. **Buildup material and depth of measurement**
   TLDs and OSLs may have a small thickness or encapsulation that adds a layer of buildup, meaning they might not measure exactly at the skin surface but slightly deeper. This must be taken into account when interpreting the dose.
4. **Detector placement and reproducibility**
   Accurate placement on the skin is critical. Misalignment or movement can lead to measurement errors. It’s important to place them securely and consistently in the correct location.
5. **Spatial resolution**
   Because they have a finite size, TLDs and OSLs average the dose over a small area. In areas with steep dose gradients, like near field edges or the skin surface, this may lead to under- or over-estimation.
6. **Calibration and readout**
   The detectors must be calibrated under similar conditions to those used clinically. Proper handling and readout are essential to avoid introducing errors, such as signal fading or inconsistencies between readouts.
7. **Environmental sensitivity**
   TLDs are sensitive to heat, and OSLs to light. They must be protected before and after exposure to prevent signal loss or noise.

**Step 4: Final point**
When using TLDs or OSLs for skin dosimetry, careful attention to calibration, positioning, angular effects, and physical properties of the detectors is necessary to ensure accurate and reliable dose measurements.

Moonshot-v1:

According to AAPM TG-191, when using TLDs (Thermoluminescent Dosimeters) or OSLDs (Optically Stimulated Luminescent Dosimeters) for skin dosimetry, the following considerations should be taken into account:

**1. Material and Dosimeter Selection**

- **Common Materials**: TG-191 provides specific details for TLD-100 and nanoDotTM dosimeters due to their prevalence in clinical practice.
- **Special Applications**: Other types of TLDs and OSLDs are available, each with unique features and limitations. The choice of dosimeter should be based on the specific requirements of the measurement.

**2. Calibration and Handling**

- **Calibration Coefficient**: For TLDs, a calibration coefficient must be determined for every reading session. For OSLDs, it can be determined for each session or a calibration curve can be used.
- **Readout Reproducibility**: At least three readouts should be performed for each OSLD to ensure reproducibility.
- **Fading and Depletion**: OSLDs should be read within 10 minutes to minimize fading, while TLDs should be annealed for at least 12 hours.

**3. Measurement Accuracy**

- **Linearity and Correction Factors**: Correction factors for linearity (kL), beam quality (kQ), and angular sensitivity (kθ) should be applied if high precision is required.
- **Energy Dependence**: Special attention should be paid to energy dependence, especially in applications like brachytherapy and imaging.

**4. Special Considerations for Skin Dosimetry**

- **Surface Dosimetry**: Dosimeters do not measure at the surface directly and may overestimate doses by 10-40%. Using a bolus over the dosimeter can help mitigate this issue.
- **Out-of-Field Measurements**: For measurements outside the treatment field, especially with high-energy photons, select a dosimeter that is neutron-insensitive to avoid over-response.

**5. Safety and Reuse**

- **Handling**: TLDs and OSLDs should be handled with care to avoid damage. Good laboratory practices should be followed, including washing hands after handling.
- **Reuse**: TLDs can be reused indefinitely with a consistent annealing procedure. OSLDs, like nanoDot, should be used up to a cumulative dose of 10 Gy to avoid complications.

**6. General Recommendations**

- **Phantom and In Vivo Measurements**: When starting with a new dosimeter, compare it with the old one using phantom and in vivo measurements to ensure similar precision.
- **Consistent Procedures**: Use consistent procedures for annealing/bleaching, storage, and handling to maximize reproducibility.

By following these guidelines, TLDs and OSLDs can be effectively used for accurate skin dosimetry in clinical settings.

Questions20 (TG157)

Let’s think step by step. According to AAPM TG reports, what is the tolerance of output factors for a monte-carlo based treatment planning system?

RTPhy-ChatBot:

The AAPM TG reports do not provide specific tolerance values for output factors in the context of Monte Carlo-based treatment planning systems. However, they emphasize the importance of monitor unit verification programs to ensure accurate dose calculations and deliveries.

AAPM Task Group 71 provides a formalism for calculating monitor units, while AAPM Task Group 114 discusses methods and requirements for verifying data for collimator settings greater than 3.0 cm. These reports were likely used to configure treatment planning systems for use with broader beam sizes.

The need for monitor unit verification programs was identified early in the adoption of intensity-modulated radiation therapy (IMRT) treatment planning and delivery techniques due to the complexity of these treatments. Monte Carlo methods are often used for IMRT planning because they can more accurately model complex dose distributions, but they require extensive computational resources and longer calculation times compared to other methods.

Therefore, it is essential to ensure that the Monte Carlo-based treatment planning system is correctly configured and calibrated to deliver accurate doses. Regular verification of monitor units using phantom measurements or other methods can help maintain confidence in the system's accuracy. However, specific tolerance values for output factors are not mentioned in the provided AAPM TG reports.

Gemini-2.0-flash:

While I cannot provide a definitive tolerance for output factors specifically for Monte Carlo-based treatment planning systems according to *all* AAPM TG reports, here's a summary of relevant information and considerations based on available reports:

**General Tolerance for Output Factors:**

- AAPM TG reports generally recommend a tolerance of **±2% to ±3%** for the constancy of the monitor output for photon and electron beams under reference conditions (e.g., TG-40, TG-142).

**Considerations for Monte Carlo-Based TPS:**

- **TG-105:** This report discusses the clinical implementation of Monte Carlo (MC) based treatment planning systems. It highlights the unique aspects of MC calculations, such as statistical uncertainties and the need for careful beam modeling and commissioning. While it doesn't specify a direct tolerance for output factors calculated by MC, it emphasizes the importance of **validating the entire treatment planning system**, including the accuracy of its output factor calculations, through measurements.
- **TG-157:** This report focuses on beam modeling and commissioning for MC-based TPS. It underscores the need for **thorough commissioning of the beam model**, which includes verifying the accuracy of calculated output factors against measurements for various field sizes and conditions. The acceptable tolerance would likely align with the general recommendations for conventional TPS, but the emphasis is on validating the MC model's ability to predict these factors accurately.
- **TG-224:** While focused on proton therapy, this report on comprehensive proton therapy machine quality assurance highlights the importance of accurate beam position and energy measurements. The underlying principle of verifying the TPS output with independent measurements applies to photon and electron MC-based systems as well.

**Key Takeaways:**

- While specific AAPM TG reports might not isolate a tolerance solely for output factors calculated by Monte Carlo TPS, the general tolerance for output constancy in radiotherapy (±2% to ±3%) serves as a benchmark.
- The emphasis for MC-based TPS is on **comprehensive validation of the entire system**, ensuring that the MC algorithm, beam model, and calculations accurately predict output factors under various clinical conditions.
- **Direct measurements remain crucial** for verifying the accuracy of the MC-calculated output factors during commissioning and routine quality assurance. The tolerance for the agreement between calculated and measured output factors should align with the general recommendations (±2% to ±3%).

**In conclusion, while a specific tolerance solely for MC-calculated output factors might not be explicitly stated in all AAPM TG reports, the general tolerance for output constancy applies, and thorough validation against measurements is paramount to ensure the accuracy of Monte Carlo-based treatment planning systems.** You should consult specific TG reports like TG-105 and TG-157 for detailed guidance on commissioning and QA of MC-based TPS.

GPT-4o:

et’s go step by step to determine the tolerance of **output factors** for a **Monte Carlo-based treatment planning system (TPS)**, according to AAPM Task Group reports like **TG-105** and **TG-114**.

**Step 1: What are output factors?**
Output factors are the relative dose outputs for various field sizes compared to a reference field size, typically 10×10 cm². They are important for accurate dose calculations in a treatment planning system.

**Step 2: What is special about Monte Carlo-based TPS?**
Monte Carlo (MC) TPS simulates radiation transport using statistical methods to achieve high accuracy. It is especially useful for complex cases, small fields, and heterogeneous media. However, its accuracy depends on proper modeling and commissioning.

**Step 3: Why do we check output factor accuracy?**
Correct output factors are essential for dose accuracy across different field sizes. They must be validated against measurements during TPS commissioning.

**Step 4: What do AAPM TG reports recommend?**
AAPM TG-105 and TG-114 suggest that for a Monte Carlo TPS, the calculated output factors should agree with measured values within:

**±2% for standard field sizes**

This tolerance ensures the TPS is accurately modeling the field size dependence of output. For very small fields or complex geometries, a slightly higher tolerance (such as ±3%) may be acceptable if properly justified due to increased uncertainty in measurement and modeling.

**Step 5: Summary**
The recommended tolerance for output factors in a Monte Carlo-based treatment planning system is **±2%**, consistent with expectations for accurate clinical use.

Moonshot-v1:

According to AAPM TG-157, the tolerance for output factors in a Monte Carlo-based treatment planning system is **2%**. This tolerance applies to both photon and electron beams, and it is used to ensure that the calculated dose distributions are sufficiently accurate for clinical use.
